# Getting to 90-90-90 in paediatric HIV: What is needed?

**DOI:** 10.7448/IAS.18.7.20770

**Published:** 2015-12-02

**Authors:** Mary-Ann Davies, Jorge Pinto, Marlène Bras

Nearly a year ago, UNAIDS launched the ambitious “90–90–90” targets to help end the AIDS epidemic: by 2020, 90% of people living with HIV will be diagnosed, 90% of those diagnosed will be on sustained antiretroviral therapy (ART) with 90% viral suppression in those on ART [1]. A welcome feature of the targets is that they focus not just on expanding access to diagnosis and treatment but also on quality of care in terms of retention and suppression, which are key to optimal HIV outcomes. Perhaps the greatest challenge in achieving these targets will be ensuring that their reach is extended to all populations everywhere. It is therefore encouraging and appropriate that the 90–90–90 targets prioritize equity across populations, with specific focus on their achievement for children and adolescents [1]. The Collaborative Initiative for Pediatric HIV Education and Research has sponsored this supplement of the journal to highlight some of the challenges and ways forward towards attaining 90–90–90 for children and adolescents. Many of these are outlined in the opening paper by Abrams and Strasser [2].

## Why are these targets so important for children and adolescents?

### Children and adolescents have not gone away

Despite an impressive 40% reduction in mother-to-child HIV transmission (MTCT) in the last five years, there were still an estimated 220,000 new paediatric infections in 2014 [3]. Due to years of failure to prevent MTCT, as well as the success of ART programmes in keeping children alive, we are left with a legacy of an estimated 2.6 million children <15 years living with HIV worldwide currently, nearly 90% of them in sub-Saharan Africa (SSA) [4]. Every day, 410 children die from HIV across the world [3]. By 2020, it is estimated that there will still be well over one million children <15 years old needing ART [5]. However, the burden of paediatric HIV will shift from toddlers and young children not on ART, to older children and adolescents, a growing proportion of whom will have initiated ART [6]. There are currently 2.1 million HIV-infected adolescents aged 10–19 [7] and nearly one-sixth of all new HIV infections are in adolescents aged 15–19 [8]. Until we successfully reduce HIV incidence in adolescents, this population will continue to grow, being a mix of those recently infected together with long-term survivors of perinatal infection.

### Children lag in access to diagnosis and treatment

Infants, children and adolescents continue to have the largest gaps in HIV diagnosis and treatment [3,4,8]. Despite encouraging recent scale-up of early infant diagnostic (EID) services, only half of HIV-exposed infants received an EID test before two months of age in 22 Global Plan priority countries during 2014 [9]. In older children, although there is potential for HIV diagnosis within child survival programmes, integration of provider-initiated testing and counselling remains limited [10,11]. A large burden of undiagnosed perinatally acquired HIV-infection in adolescents has been identified in primary care clinics and other services [12–14]. Among older youth aged 15–19 in East and Southern Africa, only one in three girls and one in five boys had ever tested for HIV and received their results [15].

It is well-known that the treatment gap for children remains vast and substantially larger than that of adults, with less than a third of HIV-infected children <15 years receiving ART in 2014 [4]. Given the high pre-ART mortality in infants [16–18], the treatment gap for children would be even larger if the denominator for determining treatment access was all newly infected individuals, rather than just those surviving with HIV [19]. While global data on treatment access for adolescents is lacking, a South African survey suggests that the proportion of HIV-infected adolescents on ART is less than half of that in any other age group [20].

### Treatment of infants and children is life-saving and prevents later chronic morbidity

In the absence of treatment, perinatally HIV-infected infants experience extraordinarily high mortality, which can be reduced by 75% with immediate ART in children <3 months of age [16–18,21]. This is undoubtedly the strongest evidence for the urgency of paediatric ART and immediate treatment of infants must be a priority. However, the goals of any medical intervention including ART go beyond averting death and severe morbidity, and extend to optimizing wellness. For example, the CHER study demonstrated significantly better neurocognitive outcomes and less comorbidity with immediate compared to deferred ART [22,23]. There is no randomized controlled trial evidence of the benefit of starting ART within the first few weeks of life as addressed by Cotton *et al*. [24]. However, arguments in favour of diagnosing and treating paediatric HIV soon after birth include the rapid disease progression in early infancy and the potential to lower viral reservoirs with possible later treatment-sparing options [25–27].

In older children, a causal modelling study showed small but significantly reduced mortality with universal ART in children aged 5–10 years, and studies consistently show better height gain with immediate treatment in children [28–31]. Cohort studies suggest that once stunted, children may not be able to attain normal height after starting ART even if virologically suppressed [32,33]. Furthermore, puberty is delayed with ART initiation at older ages and more severe disease, so deferred ART may result in permanently reduced adult height [34,35]. Immune reconstitution may also be better with earlier ART [36,37]. Similarly, as outlined in the article by Vreeman *et al*. [38], increased access to ART has been associated with reductions in HIV-associated comorbidities in children, including HIV encephalopathy, HIV-associated nephropathy, anaemia and malignancy. Importantly, manuscripts in this issue by Chamla *et al*. [39] and Rabie *et al*. [40] highlight the reduced risk of tuberculosis in children on ART. This is a significant benefit given the exceptionally high risk of infection with both drug-sensitive and -resistant organisms from early infancy onwards in settings where most HIV-infected children live, the complexity of co-treatment especially in young children and the substantial risk of permanent sequelae, especially following tuberculous meningitis [40,41].

### Focusing on treatment success in children is critical as they require lifelong treatment

The second and third “90s”, namely retention on ART and achieving viral suppression on first-line therapy, are paramount for children who face lifelong treatment with access to a limited range of alternative drugs. These goals are important to prevent exhausting limited treatment options and to achieve optimal ART outcomes, as well as to prevent transmission of multi-drug resistant viruses when these children grow up with HIV and become sexually active. In addition, sustained virologic suppression, especially from early infancy, is associated with better neurocognitive and growth outcomes as well as reduced viral reservoirs [42–44].

While reports from individual research cohorts suggest that good retention and viral suppression are possible, more routine programmatic data reflects a less optimistic picture [45,46]. In an analysis of routine data of >13,000 children from SSA and Asia, loss to follow-up (LTFU) by 18 months after ART initiation was higher in SSA, ranging from 9.0% in Southern Africa to 21.8% in West Africa [47]. In addition to young age and disease severity, requirement to pay for drugs or services and larger clinic size were associated with higher LTFU [47,48]. Recent systematic reviews of mostly research cohorts suggest that viral failure is higher in children than adults, although comparisons are difficult due to study heterogeneity [49,50]. The same review noted that 90% of children who had failed therapy had at least one resistance mutation, with 76% of children developing resistance within a year of failure. Even in children on lopinavir-based first-line with a high genetic barrier to resistance, 11% had lopinavir mutations [51].

### Adolescents are an especially vulnerable group

Adolescents experience obstacles to accessing health services on their own, including stigma, lack of youth-friendly services and parental consent policies, making this a key group for targeting 90–90–90 [1,13]. Whether transitioning from paediatric services or initiating HIV care for the first time, adolescents also frequently struggle with the linked domains of adherence, retention, stigma, disclosure and negotiation of sexual relationships [52]. These difficulties are exacerbated in a context of social and structural deprivation described by Cluver *et al*. [53], and by the complexities of transitioning to adult services as outlined by Lee and Hazra [54]. Adolescents are the only age group in which AIDS-related deaths are increasing [15], with HIV being the leading cause of adolescent deaths in Africa and the second leading cause of death among adolescents globally [55,56]. There is limited experience with transition of adolescents to adult services in resource-limited settings; however, in the UK adolescents experienced a five-fold increased mortality risk after transition to adult health services [57]. Achieving 90–90–90 among adolescents is important not only for their own health but also to prevent transmission. Adolescents have a high lifetime potential of transmitting HIV as HIV risk behaviour tends to be highest at young ages and those adolescents who become horizontally infected earlier generally also engage in sexual and other risk behaviours [58].

### A focus on children means focusing on adults too

While the 90–90–90 targets make us think about those already HIV-infected, one of their most important benefits will be in the reduction of new HIV infections [59]. Ending paediatric HIV critically requires improving diagnosis and treatment in adults, both to directly prevent MTCT, but also to prevent incident infections in adults. There is increasing recognition that Option B+ will not achieve virtual elimination of paediatric HIV unless accompanied by reductions in adult HIV incidence, as incident infection in pregnant and breastfeeding women after a first negative antenatal test is one of the key drivers of ongoing mother-to-child transmission [60,61]. Bringing an end to paediatric AIDS therefore means achieving 90–90–90 for children and adults everywhere.

## Making the most of the 90–90–90 targets for children and adolescents

**Figure 1:**
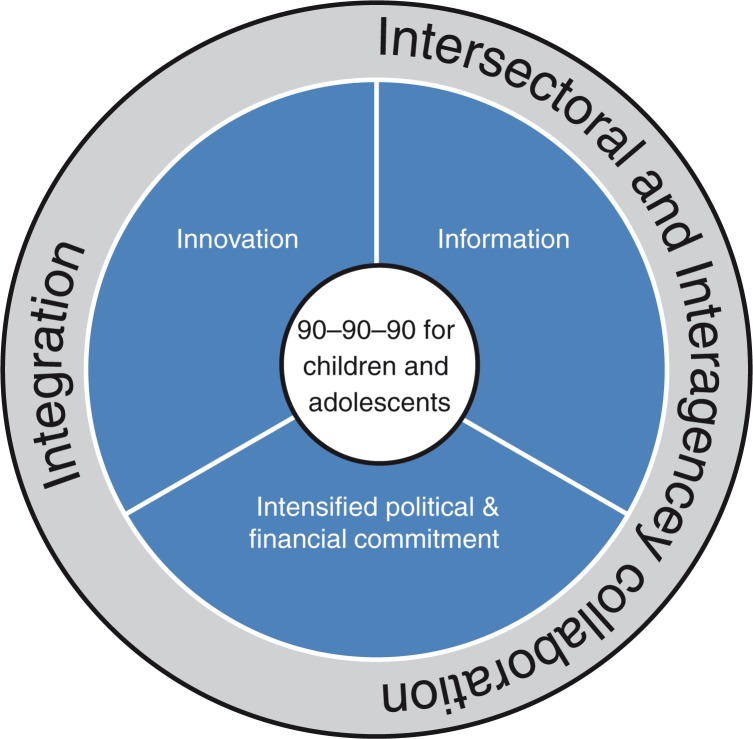
Strategies to achieve 90–90–90 for children and adolescents.

The specific focus on children and adolescents in the UNAIDS 2020 targets, together with alignment of political commitment and financial resources, provides a much-needed opportunity to address previous inequities both in research and service delivery for paediatric HIV. The articles in this issue describe a number of challenges and barriers to achieving the targets, but also important linked strategies for overcoming them, which are represented in the conceptual framework in Figure 1.

### Information

One of the major barriers to improving paediatric HIV care is the paucity of paediatric HIV research. There is frequently little or no high-quality evidence on which to base policies and guidelines. Many paediatric HIV care recommendations in WHO and national guidelines therefore remain conditional, rather than strong, with a risk of less commitment to their implementation [31,62,63]. The effects of limited paediatric research range from the inferior and limited HIV and tuberculosis drugs and formulations available for children in all age groups, highlighted in this supplement by Boerma *et al*. [64], Cotton *et al*. [24], Penazzato *et al*. [65] and Rabie *et al*. [40], to the lack of evidence-based transition models for adolescents moving to adult HIV care [54]. While paediatric HIV research is not easy for a number of reasons, including the developmental biology of children, decreased number of new paediatric infections, and complex but important regulatory and ethical requirements, it is essential if we are to achieve the 90–90–90 targets. Both clinical and implementation science research is needed to identify more effective and safer ways of treating children, especially newborns [24], adolescents [65] and those failing therapy [64], as well as how best to operationalize and deliver interventions at scale in a range of settings [66].

The need for information extends beyond academic research to monitoring and evaluation of routine programmes – we will not know whether we have met the 90–90–90 targets unless we measure them, and we are unlikely to achieve them unless we monitor our progress (or lack thereof) towards them, using the information to improve programmes. In this respect, the lack of access to routine viral load monitoring in many settings is a major obstacle both to achieving 90% suppression and knowing how close or far off we are.

### Innovation

We will not reach 90–90–90 for children with a “business as usual” approach. Many articles in this issue discuss innovations both within and outside the health system that show promise in improving paediatric and adolescent HIV care. For example, Essajee *et al*. [67] review four innovative approaches to EID, namely point-of-care testing, use of SMS printers to connect laboratories and peripheral facilities, alternative health system entry points for EID and birth testing. Lee and Hazra [54] emphasize the need for innovative transition models that use a public health approach and can be implemented at scale in resource-limited settings, and Abrams and Strasser [2] point out that service delivery innovations such as youth-friendly services and community-, school- and home-based ART are long overdue as we seek to achieve quality ART scale-up for children. Cluver *et al*. [53] argue that we need to combine biomedical solutions with social protection innovations beyond the health system, including cash transfers, parental and education support (“cash, care, classroom”), to increase uptake of prevention and treatment technologies in adolescents. In addition, Penazzato *et al*. [65] outline a role for innovative trial design to fast-track comparisons of new drugs in children.

### Intensified political and financial commitment

Abrams and Strasser [2] emphasize the need for political commitment and financial resources to chart a steady course to the 90–90–90 targets for children. In securing this commitment, it is helpful that the new Sustainable Development Goals support the UNAIDS targets, including the aim of ending the epidemics of AIDS and tuberculosis by 2030 [68]. UNAIDS has estimated the resource requirements to meet the goal of ending AIDS will increase incrementally, reaching US$18 billion by 2020, with modest declines through to 2030 [1]. While these costs may seem daunting in the context of diminishing global HIV funding, there will likely be severe cost implications for HIV programming beyond 2020 if the necessary investments to accelerate the end of AIDS are not made now [1]. Globally and at country level, we need to continue to scale up advocacy for funding from all sources. At the same time we need to improve and use information about cost-effectiveness and programme efficiency gains, innovative financing mechanisms and broader economic analysis so that finite resources are used in the most efficient way.

### Integration

Integration has been a “buzzword” in adult HIV for several years, with emerging promising practices for children and adolescents. The need for integration is highlighted by a number of articles in this supplement. As described by Chamla *et al*. [66], the rationale for integration includes the conventional goals of improving service delivery, health outcomes and efficiencies as demonstrated by improved outcomes following implementation of the Integrated Management of Childhood Illness (IMCI). Integration can also provide a platform for dissemination of innovations such as point-of-care diagnostics and viral load assays. The double dividend initiative launched in 2013 is one integrating approach intending to catalyse accelerated action towards both ending paediatric HIV and improving child survival [69]. It aims to identify service delivery platforms that provide better care for HIV-affected and - infected children through strategic investments from which all children can benefit. Integration is a promising strategy to address missed opportunities for HIV diagnosis, especially in infants missed or lost from PMTCT programmes, delayed ART initiation and poor retention, treatment of comorbidities and improved adolescent care. Rabie *et al*. [40] identify components of tuberculosis, HIV, antenatal and IMCI care where linkage and integration would facilitate optimal delivery of tuberculosis preventive and treatment services to HIV-infected children. In a previous CIPHER supplement in this journal, Bekker *et al*. [58] emphasized the role of integration in adolescent-centred rather than speciality-centred services, with comprehensive peer-guided youth-friendly one-stop shops in a diverse array of community-based settings. Critically, services need to link HIV-testing and diagnosis with prevention and treatment services, address other adolescent health needs, especially sexual and reproductive health, and, as outlined by Lee and Hazra [54], prepare adolescents for transition to adult services.

### Interagency and intersectoral collaboration

There is a huge diversity of role players and stakeholders in paediatric and adolescent HIV, both within and outside the health service. Stakeholders include funding agencies, policy makers, researchers, implementing partners, ministries of health, industry (pharmaceutical and diagnostic), health care workers, non-profit and community-based organizations, as well as, importantly, children, adolescents and caregivers themselves. Like previous targets, the 90–90–90 agenda provides an opportunity for these groups to work towards a common goal, facilitating collaboration. For example, Chamla *et al*. [66] highlight that integration has tended to focus at the level of service delivery, but needs to step up to full integration across numerous health system domains including governance, human resources, information and financing. The Pediatric HIV Treatment Initiative (PHTI) (discussed by Penazzato *et al*. [65]) is an important multi-stakeholder activity that aims to accelerate development of and access to WHO-recommended paediatric antiretroviral formulations by co-ordinating drug development and engaging industry to ensure sharing of intellectual property rights to facilitate formulation development. The Interagency Task Team on prevention and treatment of HIV infection in pregnant women mothers and children (IATT) is a collaboration that provides formulary guidance on optimal paediatric antiretrovirals [65]. Demand for different drugs is consolidated through endorsement of this formulary by major implementers and purchasers. Another example of intersectoral collaboration in drug development has been the recognition in 2010 of paediatric HIV as a “neglected disease” by the Drugs for Neglected Diseases initiative (DNDi) [70]. In consultation with experts from countries where HIV is endemic, major research institutions, and international and non-governmental organizations, DNDi has developed “ideal” and “acceptable” specifications for desired formulations/combinations of paediatric antiretrovirals and identified priorities for acceleration of clinical studies.

Intersectoral collaboration needs to extend beyond the health system and its traditional partners. Cluver *et al*. [53] point out that key HIV risk behaviours as well as treatment adherence or non-adherence do not happen in the clinic, but in social and family spaces where children and adolescents live. While we clearly need health system and clinical innovations to achieve 90–90–90, treatment and prevention interventions will be far more effective if they take into account the social and structural context that drives the decisions and behaviours of children, adolescents and their caregivers.

### Final comments

There can be no keener revelation of a society's soul than the way it treats its children.

– Nelson Mandela

There are many challenges to reaching the 90–90–90 targets for children and adolescents. They require a range of linked activities by multiple players working together with concerted effort at many levels within and beyond the health system. While targets can be criticized, they drive progress and help to consolidate and renew financial and political commitment to HIV prevention and treatment. These targets therefore offer the global community an opportunity to focus on children, and the very real and remarkable possibility of ending paediatric HIV.
