# Supplementary material for: Getting to 90-90-90 in paediatric HIV: What is needed?
Source: J Int AIDS Soc. 2015 Dec 2;18(7Suppl 6):20770. doi: 10.7448/IAS.18.7.20770 (PMC4813611; doi:10.7448/IAS.18.7.20770)
Supplement: Related texts

# Sub article: 90-90-90 – Charting a steady course to end the paediatric HIV epidemic

**DOI:** 10.7448/IAS.18.7.20296

**Published:** 2015-12-02

**Authors:** Elaine J Abrams, Susan Strasser

**Affiliations:** 1 ICAP, Mailman School of Public Health, Columbia University, New York, NY, USA; 2 Department of Pediatrics, College of Physicians & Surgeons, Columbia University, New York, NY, USA

**Keywords:** paediatric HIV, prevention of mother-to-child HIV transmission, antiretroviral treatment, antiretroviral adherence

## Abstract

**Introduction:**

The new “90-90-90” UNAIDS agenda proposes that 90% of all people living with HIV will know their HIV status, 90% of all people with diagnosed HIV infection will receive sustained antiretroviral therapy and 90% of all people receiving antiretroviral therapy will have viral suppression by 2020. By focusing on children, the global community is in the unique position of realizing an end to the paediatric HIV epidemic.

**Discussion:**

Despite vast scientific advances in the prevention and treatment of paediatric HIV infection over the last two decades, in 2014 there were an estimated 220,000 new paediatric infections attributed to mother-to-child HIV transmission (MTCT) and 150,000 HIV-related paediatric deaths. Furthermore, adolescents remain at particularly high risk for acquisition of new HIV infections, and HIV/AIDS remains the second leading cause of death in this age group. Among the estimated 2.6 million children less than 15 years of age living with HIV infection, only 32% were receiving life-saving antiretroviral treatment. After decades of languishing, good progress is now being made to prevent MTCT. Unfortunately, efforts to scale up HIV treatment services have been less robust for children and adolescents compared with adult populations. These discrepancies reflect substantial gaps in essential services and numerous missed opportunities to prevent HIV transmission and provide effective life-saving antiretroviral treatment to children, adolescents and families. The road to an AIDS-free generation will require bridging the gaps in HIV services and addressing the particular needs of children across the developmental spectrum from infancy through adolescence. To reach the ambitious new targets, innovations and service improvements will need to be rapidly escalated at each step along the prevention-treatment cascade.

**Conclusions:**

Charting a successful course to reach the 90-90-90 targets will require sustained political and financial commitment as well as the rapid implementation of a broad set of systematic improvements in service delivery. The prospect of a world where HIV no longer threatens the lives of infants, children and adolescents may finally be within reach.

## Introduction

In October 2014, in a bold effort to accelerate an end to the AIDS epidemic, UNAIDS proposed ambitious new targets to accelerate the HIV treatment scale-up in low- and middle-income countries [1]. The targets, described as “90-90-90,” propose that 90% of all people living with HIV will know their HIV status, 90% of all people with diagnosed HIV infection will receive sustained antiretroviral therapy and 90% of all people receiving antiretroviral therapy will have viral suppression by 2020 [1]. It is posited that achievement of these 90-90-90 targets in the next decade has the potential to transform and likely end the AIDS epidemic by 2030. Unimaginable a decade ago, the prospect of an AIDS-free world may finally be within reach. Charting a successful course to an AIDS-free world, however, will require sustained political and financial commitment as well as a series of systematic improvements in service delivery. This is particularly the case as we apply the 90-90-90 paradigm to paediatrics which would require identifying 3.7 million infants, children and adolescents with HIV infection, treating 3.3 million and achieving viral suppression among 3 million within the next four years.

Remarkable achievements connote the history of the antiretroviral treatment (ART) scale-up in low- and middle-income countries with more than 13.5 million individuals initiated on treatment and a slow but steady restoration of health and stability to many communities devastated by HIV infection [2]. Children have also benefited from global efforts to prevent and treat paediatric HIV infection but successes, particularly in efforts to scale up treatment, have been less robust compared with adult populations. Despite substantial scientific advances in the prevention and treatment of paediatric HIV infection, in 2014 there were 220,000 new paediatric infections attributed to mother-to-child HIV transmission (MTCT), and in 2013, close to a quarter of a million new HIV infections reported in adolescents 15–19 years of age [3, 4]. Among the estimated 2.6 million children less than 15 years of age living with HIV infection, only 32% were receiving life-saving ART. Furthermore, in 2013, 210,000 children and 120,000 adolescents died because of HIV/AIDS [4, 5]. HIV is now the second leading cause of death among adolescents, second only to road traffic injuries. It is estimated that 32% of adults living with HIV are virally suppressed but global estimates for children are not available [6]. Reported viral suppression rates among children on ART therapy in low- and middle-income countries range from 50 to 90%, varying by regimen, treatment duration, geographic location, age and developmental stage [7–10]. Overall, these statistics not only reflect notable accomplishments in comparison to years past, but also underscore the multiple gaps in essential services and numerous missed opportunities to prevent, treat and successfully manage HIV infection. The road to an AIDS-free generation will require bridging the gaps in HIV services and addressing the particular needs of children across the developmental spectrum from infancy through adolescence.

In well-resourced countries where scientific discoveries have been rapidly translated into policy and clinical practice, new paediatric HIV infections are increasingly rare, and most children living with HIV have aged into adolescence and adulthood. Consistent and widespread access to comprehensive antenatal care coupled with routine HIV testing and combination antiretroviral therapy for pregnant women has resulted in MTCT rates of less than one percent in many settings [11, 12]. At the same time, the availability of an ever-increasing and improving armamentarium of highly efficacious, virally suppressive ARTs has transformed perinatal HIV infection into a chronic, treatable disease of adolescence and adulthood. In the United Kingdom and Ireland, in 2011, the average age of 1131 children followed in the Collaborative HIV Paediatric Study was 13.2 years, with approximately 1/3 of children over 15 years of age [13]. Similarly, in 2011 in New York City, an epicentre of the paediatric HIV epidemic in the United States, among 2449 children with perinatal HIV, 11% were reported to be older than 24 years of age with 76% between the ages of 13 and 24 years [14].

While the population of children with perinatally acquired HIV is ageing in the global north, this is juxtaposed against persistently high transmission and mortality rates amongst affected paediatric populations in sub-Saharan Africa. What will it take to further accelerate prevention and treatment efforts in sub-Saharan Africa and other resourced constrained settings to achieve successes similar to those in the global north? Unfortunately, ART was late to arrive in sub-Saharan Africa where more than 90% of children with HIV infection live and, for many years, efforts to prevent perinatal infections were hampered by repeated attempts to find quick, easy and inexpensive approaches rather than building an enabling platform of HIV services to deliver efficacious antiretroviral therapy to pregnant women to prevent MTCT and treat maternal HIV infection [15, 16]. The road has been bumpy and mired by mixed messages on breastfeeding and frequent shifts in prevention of mother-to-child transmission (PMTCT) drug regimens and approaches. More recently, important incremental improvements and innovations across the paediatric prevention–treatment cascade have resulted in better outcomes – fewer new infections, and decreased morbidity and mortality for those with established disease. To reach the ambitious new targets, however, innovations and service improvements will need to be rapidly escalated at each step along the cascade.

## Discussion

### Preventing new paediatric HIV infections

First proposed as a solution to the poorly functioning PMTCT programme in Malawi, the Option B+ approach was hotly debated [17]. It was believed, on one hand, that Option B+ would pave the road to elimination of new paediatric infections by initiating lifelong treatment for all pregnant and breastfeeding women, maternal health would be protected and transmission would be prevented during current and future pregnancies, and to uninfected partners [18]. On the other hand, there was deep concern that Option B+ would result in costly, unnecessary treatment for millions of women and dangerous drug exposures for their babies. Four years later, the Option B+ approach has been endorsed by the majority of high HIV burden countries in sub-Saharan Africa, and on balance, early field experience suggests that it has leapfrogged over other approaches to increase ART uptake among pregnant and breastfeeding women and reduced loss along the first steps of the PMTCT cascade from HIV diagnosis to treatment initiation [19, 20]. This approach, though initially engendering scepticism, has charted a course for potential systematic improvements in care. Several countries have reported dramatic increases in the number of HIV-positive pregnant and breastfeeding women starting ART [20]. While this is excellent news, early reports also suggest that retention on treatment is less than optimal and substantial numbers of women on Option B+ do not remain engaged in care [20–23]. It appears that some of the early losses in the PMTCT cascade, between diagnosis and treatment initiation, may simply have been postponed rather than prevented [23].

A wide array of individual, structural and health systems factors contribute to whether a woman and her baby can and will stay in care [17–20,22–30]. Many of these factors overlap with those described by non-pregnant adults as reasons for non-retention including high out-of-pocket costs, long travel times, inconvenient clinic hours, poor treatment by health workers and self-transfer to other health facilities. Each of these factors, either individually or collectively, threatens achievement of elimination goals. Option B+ has been generally interposed onto an already fragile maternal-child health (MCH) structure ill-equipped to provide comprehensive treatment services during pregnancy and especially during the postpartum period. In addition, social and cultural influences affect health seeking behaviour and health service use during this time period. In many settings, pregnant women migrate to family homes for delivery and the early postpartum period, and some may leave their babies with families when they return to work. In some parts of the world, access to and uptake of antenatal care is low, and large numbers of women are unable to learn their HIV status or obtain HIV care in a timely way. Finally, despite an explosion of electronic health information systems and mobile health technologies globally, penetration in MCH services has been poor, and the paper-based medical record and register-based systems remain the norm [31]. Different service delivery models and nimble, responsive and adaptive health platforms are needed to expand access to care and improve service delivery and retention along the PMTCT cascade.

Attention urgently needs to shift downstream in the PMTCT cascade, from getting women on ART to engaging them in lifelong care, and to augmenting the health systems now responsible for providing these services. This can only be done by applying successful interventions already in place in services where non-pregnant adults traditionally receive ART, but also by understanding and addressing the particular needs of women and their children during this period of time [32]. In addition to reinforcing the MCH platform to ensure that PMTCT services can be delivered, innovations both small (organized appointment systems, SMS reminders, extended prescriptions, mother–baby registers) and large (integrated mother–child postnatal follow-up, community-based ART, engaging partners and communities, home-based HIV testing, multi-drug infant prophylaxis regimens) will be needed to identify, engage and retain all women and infants at risk and minimize loss and enhance efficiency across the PMTCT cascade. Without implementing improvements in how services are delivered, efforts to prevent new infections among infants will be compromised.

### Identifying and engaging children with HIV infection

Weaknesses in PMTCT programming have been particularly evident on the infant side of the cascade where early infant diagnosis (EID) and early treatment are critical to prevent rapid disease progression and reduce the high mortality rates well documented in untreated infants and young children [33]. In 2013, only 42% of HIV-exposed infants in the 21 priority countries received an early diagnostic test in the first two months of life to determine infection status [3]. Performance across subsequent steps in the cascade wanes dramatically with few HIV-exposed children remaining in care throughout the period of breastfeeding and MTCT risk. Furthermore, having a positive EID test has not directly translated into early ART initiation due to long delays from the time of taking the test to getting results to the family, preparing for and finally starting treatment [34]. Improvements in how PMTCT is delivered, as described above, are likely to have the greatest impact on infant outcomes first and foremost by reducing the number of babies acquiring HIV infection, but equally importantly, by shifting the HIV care paradigm to one that facilitates long-term, continuous tracking and engagement of mothers and their infants and by providing a platform on which to rapidly deliver diagnostic, prevention and treatment services. A variety of other innovations have been demonstrated to expedite various steps along the EID cascade such as: the use of SMS printers to return test results to health facilities quickly and systematically, engaging peer and community workers to help patients remain in care and reduce stigma, computer-based tracking systems, and expanding EID testing to additional access points beyond PMTCT programmes [35, 36]. Other approaches of great interest include the addition of an EID test at birth and introducing point-of-care (POC) diagnostics at the clinic level [37, 38]. While each has the potential to improve early identification and treatment of HIV-infected infants, it will be important to carefully consider the consequences of adding more demands onto the fragile PMTCT-EID infrastructure currently in place. Health workers juggle numerous competing demands, and facilities are often under-resourced and poorly staffed. It is of note that many country programmes are still challenged to provide the basics, such as an uninterrupted supply of rapid HIV tests to PMTCT and other clinical services.

Challenges in case finding have not been limited to infants [39]. While global estimates for the number of children living with HIV have recently been reduced from 3.2 million to 2.6 million, we find that fewer than half of all children living with HIV are diagnosed and actively engaged in care [6]. However, when concerted efforts are made to test children, not surprisingly, large numbers of HIV-infected infants, children and adolescents are identified. HIV programmes, however, have generally failed to implement routine HIV testing for children [40, 41]. Intentional and routine testing, even in high burden areas, is the exception rather than the norm. Through provider-initiated testing and counselling (PITC), inpatient wards and malnutrition, tuberculosis and urgent care clinics have been demonstrated to be high-yield venues to identify HIV-infected children, but resources are rarely available to offer routine testing [42, 43]. Testing the children of adults with HIV should also improve paediatric case finding, but adults are often unable or unwilling to bring their children and families to the clinic. Health workers are also often reluctant to initiate the conversations and counselling necessary to promote paediatric testing. Ahmed *et al*. trained community health workers in Malawi to provide clinic- and home-based testing of paediatric contacts of adult index patients, demonstrating the value of family/household testing and the importance of community-based approaches to find HIV-infected children [36]. Similarly, by introducing routine PITC in primary care clinics in Zimbabwe, Ferrand *et al*. successfully identified adolescents with HIV infection, many with symptomatic disease and presumed perinatal infection [40, 44].

If we are to reach the first target, to ensure that the status of 90% of all positive infants, children and adolescents is known, there will need to be proper resourcing for and a rapid scale-up of routine testing in these high-yield settings, both in health centres and communities. This will require making HIV testing a routine part of health services for paediatric populations particularly in high prevalence settings, training health workers on the importance of and proper approaches to testing children, improving the supply chain for HIV test kits, addressing issues around consent for paediatric and adolescent testing (e.g. age and authority to provide consent), developing effective systems to link those testing positive to HIV care and collecting data disaggregated by age to ensure better programme monitoring. Recent guidance from the United States President's Emergency Fund for AIDS Relief (PEPFAR) summarizes key strategies for identifying and linking infants, children and adolescents to HIV care and are included in Table 1 [45].

**Table 1:** Strategies to improve paediatric case finding and antiretroviral treatment^a^

	Area	Description
Cross-cutting strategies	Health work force	Increase number and capacity of health workers to provide paediatric and adolescent services; task shifting and sharing; engagement of community and lay worker cadres; health worker training; mentoring and continuing education
	Service delivery	Decentralization; integration of services including youth-friendly services and sexual and reproductive health; community, family and home-based testing, care and treatment; reduce out-of-pocket expenses; appointment systems with active tracking and follow-up including SMS and telephonic reminders
	Supply chain	Improve supply chain management of paediatric antiretroviral medications and essential commodities including HIV test kits and early infant diagnosis materials
	Monitoring and evaluation	Collect age-disaggregated testing and treatment data; document linkages between mothers and infants, testing and enrolment, transfers between facilities; improve data quality; develop approaches to routinely measure key outcomes including mother-to-child transmission rates, viral suppression, HIV drug resistance
Strategies to improve HIV testing	Early infant diagnosis	Point-of-care diagnostics; birth testing in addition to routine testing at six weeks of age; expand testing to access points outside of PMTCT programmes; centralized specimen transport schemes; SMS printers to return results to facilities
	Testing of older children and adolescents^a^	Test all children and adolescents of adults receiving HIV services through facility or home-based testing; admitted to inpatient paediatric wards; attending TB clinics, malnutrition and urgent care services; receiving orphan and vulnerable children (OVC) services
		Test mothers or infants attending immunization or under-5 clinics to identify HIV-exposed infants in high prevalence settings (>5%),
Strategies to improve HIV treatment		Universal ART for all infants, children and adolescents; remove CD4 and clinical criteria for ART initiation
		Improve drug regimens and formulations: fixed-dose formulations; dispersibles; scored adult tablets; weight band guided dosing
		Expedite development and early access for new drugs and drug combinations with improved efficacy and resistance profiles for children
		Integrated age-appropriate psychosocial and behavioural services including disclosure, adherence and sexual and reproductive health

a Adapted from “Strategies for Identifying and Linking HIV-Infected Infants, Children, and Adolescents to HIV Care and Treatment,” www.pepfar.gov/documents/organization/244347.pdf.

### Treatment for children with HIV infection

An estimated 832,000 children less than 15 years of age were reported to be receiving treatment in low- and middle-income countries in 2014 [6]. While increasing numbers of children are initiated on treatment annually, the number will need to be tripled to meet the 90-90-90 targets. Treatment coverage among children varies by geographic region, by country and, within countries, by region and district [32]. Some countries in sub-Saharan Africa, where the burden of HIV infection is greatest, have met with notable success, while others, particularly in West Africa, have seen more modest improvements in paediatric treatment. Additionally, children are still entering care late and initiating treatment at advanced stages of disease [46, 47]. Infants and adolescents have been particularly challenging to identify and initiate on treatment, contributing to high rates of mortality and suboptimal treatment outcomes in these very high risk groups [48–50].

The impediments to treatment in paediatric populations parallel those cited for testing including patient and system-level barriers. The complexity of paediatric treatment, including age and CD4-specific indications for initiation, weight-based dosing, and the need for different regimens and formulations across age groups, contrast distinctly with the simplicity of the uniform “one pill once daily” approach to adult ART. Limited health worker capacity and reported high levels of discomfort treating children have often been cited as contributing to the limited ART coverage among children.

Echoing the Option B+ debates, the proposal to treat all children, independent of age or severity of disease, has engendered heated discussion. Those in favour of universal treatment cite CD4 testing as a barrier to ART initiation and a step along the cascade with high risk for loss [51]. Retention among those not eligible for treatment (pre-ART) is substantially worse compared with children on therapy and rather than providing a venue for close monitoring, many children are lost to care, often returning late with more advanced disease [52, 53]. In addition, an emerging body of evidence suggests that some health outcomes such as immune restoration may be better when treatment is started earlier among healthier children [54, 55]. Several countries in sub-Saharan Africa, such as Zambia and Uganda, have already pushed ahead and endorsed treatment for all children under 15 years [56]. In light of findings from two adult trials demonstrating the benefit of ART in adults with high CD4 counts, the World Health Organization will recommend universal treatment for all individuals living with HIV [57, 58]. As these recommendations are taken up by national programmes, we can expect to see, similar to the B+ experience [19, 20], an uptick in the number of children on treatment in low- and middle-income countries.

In addition to simplifying ART eligibility, increasing health worker capacity to care for children through training, mentoring and sustained supervision and improving supply chain management of antiretrovirals will be essential to achieve the ambitious treatment targets. Furthermore, decentralization to primary care facilities and innovations in service delivery such as family-focused care; youth-friendly services; and community-, school- and home-based ART [59–61] are long overdue for paediatric populations. There is also a pressing need to engage with the behavioural and psychosocial issues affecting children, adolescents and families affected by HIV, particularly disclosure, mental health and, for adolescents, to ensure easy access to sexual and reproductive health services [62, 63].

## Achieving and maintaining viral suppression

Population-based estimates of viral suppression among paediatric populations are not available. In published reports, suppression rates vary from 40% to over 90%, by population, age, gender, drug regimen, calendar year, duration of ART, study design and frequency of measurement. Among 4803 children on ART in South Africa, in crude analyses at any time-point on treatment, 65.9% (95% CI 62.7–68.4%) of children receiving a community-based intervention and 55% (95% CI: 54.2–57%) in the standard of care arm were virally suppressed [7]. In a recent cross-sectional analysis of adults and children on ART in Swaziland, rates of suppression were reported at 71, 65, and 86% among <10, 10–19, and 20 years and older [8]. Achieving and maintaining viral suppression among adolescents has been particularly difficult across high- as well as low- and middle-income countries [64].

The most substantial threat to the third target has been, and continues to be, the inferior drugs and formulations available for treating children. Adult treatment has been revolutionized by new drug classes and formulations, with fixed-dose once-daily combinations of highly efficacious medications with improved toxicity profiles. Not yet widely available in resource-poor settings, many of these agents appear to be on track to enter the global market in the near future. These advances reflect consistent, aggressive efforts over the past decade to expand and improve treatment access for adults with HIV infection.

The efforts on the part of children have been less consistent and generally less successful. This is compounded by the biology of childhood, which implicitly makes paediatric drug development more complex and further limits the drug formulary for children, particularly in low- and middle-income countries. Physical growth and organ maturation directly impact drug metabolism, warranting study of dosing and safety across the age/weight spectrum of childhood and adolescence. Special formulations (granules, dispersibles and syrups) are needed to ensure proper dosing for infants and young children. Therefore, paediatric drug development is often decades behind that of adults, and an array of less optimal drugs remain the only options for children. Urgent measures are needed to expand and expedite paediatric drug development and to guarantee early access to the safest, most efficacious regimens. Long-acting formulations, currently in trial in adult populations, hold particular promise for adolescents, where the use of other long-acting formulations met with great success [65, 66]. In the interim, it remains necessary to assess innovative strategies to best employ currently available drugs and settle for less optimal and often more complex options to expand treatment access and prevent disease progression and mortality [67, 68]. Efforts to strengthen health systems as noted in the previous sections are also imperative not only to increase the number of children initiating treatment but also to improve the quality of care including providing effective adherence, monitoring and support, and maintaining an uninterrupted supply of essential medications.

Finally, achieving and maintaining viral suppression amongst 90% of children on treatment will require fully embracing the challenges of adherence to lifelong treatment as well as the complexity of paediatrics, recognizing and addressing the evolving emotional and psychosocial needs of children as they grow and develop. Children need to learn their HIV status when it is developmentally appropriate, and adolescents need to be equipped with the knowledge and skills to adhere and protect themselves and their partners as they age and mature. Emergency efforts to make these life-saving drugs available have, to date, left little room to consider the emotional wellbeing of these children and their families [69, 70]. Globally, HIV-infected children are often from vulnerable families affected by poverty, violence, limited health care and educational resources, and not infrequently, racism and discrimination. They often experience disruptions in caregiving due to a variety of factors including parental illness and death, and live in communities where HIV stigma is prevalent. Added to this list are the challenges of adhering to a daily medication regimen and grappling, when they are aware, with the knowledge of having a transmissible, potentially fatal infection. Adolescence is a particularly vulnerable time when normal neurologic and developmental changes increase the risk of acquisition of HIV infection and jeopardize retention and adherence among those with established disease [64, 71–73]. Expanding services and health worker capacity to address the psychosocial needs of children and adolescents living with HIV will be essential to fully realize the potential of the ART scale-up [74].

## Conclusions

In the history of the AIDS epidemic, targets have been enormously helpful to focus resources and attention on global inequities in treatment and other essential services. While they are oftentimes largely aspirational, the “3 by 5” initiative propelled the treatment scale-up in low- and middle-income countries [75]. For infants, children and adolescents with HIV, we find ourselves at a moment in time when political commitment and financial resources are aligned, opening a door to improving health outcomes for this special population [76]. The 90-90-90 campaign offers the prospect of a world where HIV no longer threatens the lives of infants, children and adolescents.

## References

[1] UNAIDS (2014). Ambitious treatment targets: writing the final chapter of the AIDS epidemic.

[2] World Health Organization (WHO) (2014). Global update on the health sector response to HIV, 2014.

[3] UNAIDS (2014). 2014 Progress Report on the Global Plan towards the elimination of new HIV infections among children by 2015 and keeping their mothers alive.

[4] UNAIDS (2015). All in global strategy consultation.

[5] UNAIDS (2013). Global report: UNAIDS report on the global AIDS epidemic 2013.

[6] UNAIDS (2015). How AIDS changed everything – MDG6: 15 years, 15 lessons of hope from the AIDS response.

[7] Fatti G, Shaikh N, Eley B, Grimwood A (2014). Improved virological suppression in children on antiretroviral treatment receiving community-based adherence support: a multicentre cohort study from South Africa. AIDS Care.

[8] Jobanputra K, Parker LA, Azih C, Okello V, Maphalala G, Kershberger B (2015). Factors associated with virological failure and suppression after enhanced adherence counselling, in children, adolescents and adults on antiretroviral therapy for HIV in Swaziland. PLoS One.

[9] Kekitiinwa A, Cook A, Nathoo K, Mugyenyi P, Nahirya-Ntege P, Bakeera-Kitaka S (2013). Routine versus clinically driven laboratory monitoring and first-line antiretroviral therapy strategies in African children with HIV (ARROW): a 5-year open-label randomised factorial trial. Lancet.

[10] Technau KG, Schomaker M, Kuhn L, Moultrie H, Coovadia A, Eley B (2014). Virologic response in children treated with abacavir-compared with stavudine-based antiretroviral treatment: a South African multi-cohort analysis. Pediatr Infect Dis J.

[11] Townsend CL, Byrne L, Cortina-Borja M, Thorne C, de Ruiter A, Lyall H (2014). Earlier initiation of ART and further decline in mother-to-child HIV transmission rates, 2000–2011. AIDS.

[12] Birkhead GS, Pulver WP, Warren BL, Klein SJ, Parker MM, Caggana M (2010). Progress in prevention of mother-to-child transmission of HIV in New York State: 1988–2008. J Public Health Manag Pract.

[13] Bamford A, Lyall H (2015). Paediatric HIV grows up: recent advances in perinatally acquired HIV. Arch Dis Child.

[14] New York City Department of Health and Mental Hygiene (2012). Annual report: pediatric HIV/AIDS surveillance update New York City.

[15] Cooper ER, Charurat M, Mofenson L, Hanson IC, Pitt J, Diaz C (2002). Combination antiretroviral strategies for the treatment of pregnant HIV-1-infected women and prevention of perinatal HIV-1 transmission. J Acquir Immune Defic Syndr.

[16] Abrams EJ, Myer L, Rosenfield A, El-Sadr WM (2007). Prevention of mother-to-child transmission services as a gateway to family-based human immunodeficiency virus care and treatment in resource-limited settings: rationale and international experiences. Am J Obstet Gynecol.

[17] Schouten EJ, Jahn A, Midiani D, Makombe SD, Mnthambala A, Chirwa Z (2011). Prevention of mother-to-child transmission of HIV and the health-related Millennium Development Goals: time for a public health approach. Lancet.

[18] Ahmed S, Kim MH, Abrams EJ (2013). Risks and benefits of lifelong antiretroviral treatment for pregnant and breastfeeding women: a review of the evidence for the Option B+ approach. Curr Opin HIV AIDS.

[19] Centers for Disease Control and Prevention (2013).

[20] Kieffer MP, Mattingly M, Giphart A, van de Ven R, Chouraya C, Walakira M (2014). Lessons learned from early implementation of option B+: the Elizabeth Glaser Pediatric AIDS Foundation experience in 11 African countries. J Acquir Immune Defic Syndr.

[21] Phillips T, McNairy M, Zerbe A, Myer L, Abrams EJ (2015). Postpartum transfer of care among HIV-infected women initiating antiretroviral therapy during pregnancy. J Acquir Immune Defic Syndr.

[22] Tenthani L, Haas AD, Tweya H, Jahn A, van Oosterhout JJ, Chimbwandira F (2014). Retention in care under universal antiretroviral therapy for HIV-infected pregnant and breastfeeding women (‘Option B+‘) in Malawi. AIDS.

[23] Kim MH, Ahmed S, Hosseinipour MC, Giordano TP, Chiao EY, Yu X (2015). Implementation and operational research: the impact of option B+ on the antenatal PMTCT cascade in Lilongwe, Malawi. J Acquir Immune Defic Syndr.

[24] Parker LA, Jobanputra K, Okello V, Nhlangamandla M, Mazibuko S, Kourline T (2015). Barriers and facilitators to combined ART initiation in pregnant women with HIV: lessons learnt from a PMTCT B+ pilot program in Swaziland. J Acquir Immune Defic Syndr.

[25] van Lettow M, Bedell R, Mayuni I, Mateyu G, Landes M, Chan AK (2014). Towards elimination of mother-to-child transmission of HIV: performance of different models of care for initiating lifelong antiretroviral therapy for pregnant women in Malawi (Option B+). J Int AIDS Soc.

[26] Tweya H, Gugsa S, Hosseinipour M, Speight C, Ng'ambi W, Bokosi M (2014). Understanding factors, outcomes and reasons for loss to follow-up among women in Option B+ PMTCT programme in Lilongwe, Malawi. Trop Med Int Health.

[27] Clouse K, Schwartz S, Van Rie A, Bassett J, Yende N, Pettifor A (2014). “What they wanted was to give birth; nothing else”: barriers to retention in option B+ HIV care among postpartum women in South Africa. J Acquir Immune Defic Syndr.

[28] Ebuy H, Yebyo H, Alemayehu M (2015). Level of adherence and predictors of adherence to the Option B+ PMTCT programme in Tigray, northern Ethiopia. Int J Infect Dis.

[29] Colvin CJ, Konopka S, Chalker JC, Jonas E, Albertini J, Amzel A (2014). A systematic review of health system barriers and enablers for antiretroviral therapy (ART) for HIV-infected pregnant and postpartum women. PLoS One.

[30] Hodgson I, Plummer ML, Konopka SN, Colvin CJ, Jonas E, Albertini J (2014). A systematic review of individual and contextual factors affecting ART initiation, adherence, and retention for HIV-infected pregnant and postpartum women. PLoS One.

[31] Gachuhi AB, Ndumiso D, Mudoni D, Mthethwa S, Okello V, Zerbe A Optimizing health information systems for option B+ in Swaziland.

[32] UNAIDS (2014). The gap report.

[33] Violari A, Cotton MF, Gibb DM, Babiker AG, Steyn J, Madhi SA (2008). Early antiretroviral therapy and mortality among HIV-infected infants. N Engl J Med.

[34] Motswere-Chirwa C, Voetsch A, Lu L, Letsholathebe V, Lekone P, Machakaire E (2014). Follow-up of infants diagnosed with HIV — early infant diagnosis program, Francistown, Botswana, 2005–2012.

[35] Noordam AC, George A, Sharkey AB, Jafarli A, Bakshi SS, Kim JC (2015). Assessing scale-up of mHealth innovations based on intervention complexity: two case studies of child health programs in Malawi and Zambia. J Health Commun.

[36] Ahmed S, Kim MH, Dave AC, Sabelli R, Kanjelo K, Preidis GA (2015). Improved identification and enrolment into care of HIV-exposed and -infected infants and children following a community health worker intervention in Lilongwe, Malawi. J Int AIDS Soc.

[37] Penazzato M, Revill P, Prendergast AJ, Collins IJ, Walker S, Elyanu PJ (2014). Early infant diagnosis of HIV infection in low-income and middle-income countries: does one size fit all?. Lancet Infect Dis.

[38] Finocchario-Kessler S, Gautney BJ, Khamadi S, Okoth V, Goggin K, Spinler JK (2014). If you text them, they will come: using the HIV infant tracking system to improve early infant diagnosis quality and retention in Kenya. AIDS.

[39] Ahmed S, Kim MH, Sugandhi N, Phelps BR, Sabelli R, Diallo MO (2013). Beyond early infant diagnosis: case finding strategies for identification of HIV-infected infants and children. AIDS.

[40] Kranzer K, Meghji J, Bandason T, Dauya E, Mungofa S, Busza J (2014). Barriers to provider-initiated testing and counselling for children in a high HIV prevalence setting: a mixed methods study. PLoS Med.

[41] Sibanda EL, Hatzold K, Mugurungi O, Ncube G, Dupwa B, Siraha P (2012). An assessment of the Zimbabwe ministry of health and child welfare provider initiated HIV testing and counselling programme. BMC Health Serv Res.

[42] Kankasa C, Carter RJ, Briggs N, Bulterys M, Chama E, Cooper ER (2009). Routine offering of HIV testing to hospitalized pediatric patients at university teaching hospital, Lusaka, Zambia: acceptability and feasibility. J Acquir Immune Defic Syndr.

[43] McCollum ED, Preidis GA, Golitko CL, Siwande LD, Mwansambo C, Kazembe PN (2011). Routine inpatient human immunodeficiency virus testing system increases access to pediatric human immunodeficiency virus care in sub-Saharan Africa. Pediatr Infect Dis J.

[44] Ferrand RA, Munaiwa L, Matsekete J, Bandason T, Nathoo K, Ndhlovu CE (2010). Undiagnosed HIV infection among adolescents seeking primary health care in Zimbabwe. Clin Infect Dis.

[45] PEPFAR, USAID, Centers for Disease Control and Prevention Strategies for identifying and linking HIV-infected infants, children, and adolescents to HIV care and treatment.

[46] Davies MA, Phiri S, Wood R, Wellington M, Cox V, Bolton-Moore C (2013). Temporal trends in the characteristics of children at antiretroviral therapy initiation in southern Africa: the IeDEA-SA Collaboration. PLoS One.

[47] Koller M, Patel K, Chi BH, Wools-Kaloustian K, Dicko F, Chokephaibulkit K (2015). Immunodeficiency in children starting antiretroviral therapy in low-, middle-, and high-income countries. J Acquir Immune Defic Syndr.

[48] Innes S, Lazarus E, Otwombe K, Liberty A, Germanus R, Van Rensburg AJ (2014). Early severe HIV disease precedes early antiretroviral therapy in infants: are we too late?. J Int AIDS Soc.

[49] Lowenthal ED, Bakeera-Kitaka S, Marukutira T, Chapman J, Goldrath K, Ferrand RA (2014). Perinatally acquired HIV infection in adolescents from sub-Saharan Africa: a review of emerging challenges. Lancet Infect Dis.

[50] Motswere-Chirwa C, Voetsch A, Lu L, Letsholathebe V, Lekone P, Machakaire E (2014). Follow-up of infants diagnosed with HIV – Early Infant Diagnosis Program, Francistown, Botswana, 2005–2012. MMWR Morb Mortal Wkly Rep.

[51] Fox MP, Rosen S (2015). Systematic review of retention of pediatric patients on HIV treatment in low and middle-income countries 2008–2013. AIDS.

[52] McNairy ML, Lamb MR, Carter RJ, Fayorsey R, Tene G, Mutabazi V (2013). Retention of HIV-infected children on antiretroviral treatment in HIV care and treatment programs in Kenya, Mozambique, Rwanda, and Tanzania. J Acquir Immune Defic Syndr.

[53] Nuwagaba-Biribonwoha H, Kilama B, Antelman G, Khatib A, Almeida A, Reidy W (2013). Reviewing progress: 7 year trends in characteristics of adults and children enrolled at HIV care and treatment clinics in the United Republic of Tanzania. BMC Public Health.

[54] Picat MQ, Lewis J, Musiime V, Prendergast A, Nathoo K, Kekitiinwa A (2013). Predicting patterns of long-term CD4 reconstitution in HIV-infected children starting antiretroviral therapy in sub-Saharan Africa: a cohort-based modelling study. PLoS Med.

[55] Szubert AJ, Musiime V, Bwakura-Dangarembizi M, Nahirya-Ntege P, Kekitiinwa A, Gibb DM (2015). Pubertal development in HIV-infected African children on first-line antiretroviral therapy. AIDS.

[56] E Elyanu, Magongo B, Asire B, Lukabwe I, Bitimwine H, Katureebe C Impact of implementing “Test and Treat” policy on paediatric ART enrolments and coverage in Uganda.

[57] TEMPRANO ANRS 12136 Study Group (2015). A trial of early antiretrovirals and isoniazid preventive therapy in Africa. N Engl J Med.

[58] Lundgren JD, Babiker AG, Gordin F, Emery S, Grund B, Sharma S (2015). Initiation of antiretroviral therapy in early asymptomatic HIV infection. N Engl J Med.

[59] Fayorsey RN, Saito S, Carter RJ, Gusmao E, Frederix K, Koech-Keter E (2013). Decentralization of pediatric HIV care and treatment in five sub-Saharan African countries. J Acquir Immune Defic Syndr.

[60] Grimsrud A, Sharp J, Kalombo C, Bekker LG, Myer L (2015). Implementation of community-based adherence clubs for stable antiretroviral therapy patients in Cape Town, South Africa. J Int AIDS Soc.

[61] Weidle PJ, Wamai N, Solberg P, Liechty C, Sendagala S, Were W (2006). Adherence to antiretroviral therapy in a home-based AIDS care programme in rural Uganda. Lancet.

[62] Brandt L, Beima-Sofie K, Hamunime N, Shepard M, Ferris L, Ingo P (2015). Growing-up just like everyone else: key components of a successful pediatric HIV disclosure intervention in Namibia. AIDS.

[63] Mellins CA, Nestadt D, Bhana A, Petersen I, Abrams EJ, Alicea S (2014). Adapting evidence-based interventions to meet the needs of adolescents growing up with HIV in South Africa: the VUKA case example. Glob Soc Welf.

[64] Kahana SY, Fernandez MI, Wilson PA, Bauermeister JA, Lee S, Wilson CM (2015). Rates and correlates of antiretroviral therapy use and virologic suppression among perinatally and behaviorally HIV-infected youth linked to care in the United States. J Acquir Immune Defic Syndr.

[65] Margolis DA, Brinson CC, Smith GH, de Vente J, Hagins DP, Eron JJ (2015). Cabotegravir plus rilpivirine, once a day, after induction with cabotegravir plus nucleoside reverse transcriptase inhibitors in antiretroviral-naive adults with HIV-1 infection (LATTE): a randomised, phase 2b, dose-ranging trial. Lancet Infect Dis.

[66] Tavernise S Colorado's effort against teenage pregnancies is a startling success.

[67] Kuhn L, Coovadia A, Strehlau R, Martens L, Hu CC, Meyers T (2012). Switching children previously exposed to nevirapine to nevirapine-based treatment after initial suppression with a protease-inhibitor-based regimen: long-term follow-up of a randomised, open-label trial. Lancet Infect Dis.

[68] Butler KM, on behalf of the BREATHER Trial Team ART with weekends off is noninferior to continuous ART in young people on EFV+2NRTI.

[69] Mellins CA, Malee KM (2013). Understanding the mental health of youth living with perinatal HIV infection: lessons learned and current challenges. J Int AIDS Soc.

[70] Small L, Mercado M, Gopalan P, Pardo G, Ann Mellins C, McKay MM (2014). Enhancing the emotional wellbeing of perinatally HIV infected youth across global contexts. Glob Soc Welf.

[71] Agwu AL, Fairlie L (2013). Antiretroviral treatment, management challenges and outcomes in perinatally HIV-infected adolescents. J Int AIDS Soc.

[72] Hudelson C, Cluver L (2015). Factors associated with adherence to antiretroviral therapy among adolescents living with HIV/AIDS in low- and middle-income countries: a systematic review. AIDS Care.

[73] Kim SH, Gerver SM, Fidler S, Ward H (2014). Adherence to antiretroviral therapy in adolescents living with HIV: systematic review and meta-analysis. AIDS.

[74] Strasser S, Gibbons S (2014). The development of HIV-related mental health and psychosocial services for children and adolescents in Zambia: the case for learning by doing. Child Youth Serv Rev.

[75] World Health Organization (WHO) (2015). The 3 by 5 initiative: treat three million people with HIV/AIDS by 2005.

[76] Office of the Global Health Coordinator, President's Emergency Fund for AIDS Relief (PEPFAR) (2014). Accelerating Children's HIV/AIDS Treatment (ACT) initiative [press release].

# Sub article: Reducing mortality in HIV-infected infants and achieving the 90–90–90 target through innovative diagnosis approaches

**DOI:** 10.7448/IAS.18.7.20299

**Published:** 2015-12-02

**Authors:** Shaffiq Essajee, Lara Vojnov, Martina Penazzato, Ilesh Jani, George K Siberry, Susan A Fiscus, Jessica Markby

**Affiliations:** 1 World Health Organization, Geneva, Switzerland; 2 Clinton Health Access Initiative, Boston, MA, USA; 3 Instituto Nacional de Saude, Maputo, Mozambique; 4 National Institutes of Health, Bethesda, MD, USA; 5 Department of Microbiology & Immunology, School of Medicine, University of North Carolina, Chapel Hill, NC, USA

**Keywords:** infant, HIV, diagnosis, treatment, point of care, SMS

## Abstract

**Introduction:**

Despite significant gains in access to early infant diagnosis (EID) over the past decade, most HIV-exposed infants still do not get tested for HIV in the first two months of life. For those who are tested, the long turnaround time between when the sample is drawn and when the results are returned leads to a high rate of loss to follow-up, which in turn means that few infected infants start antiretroviral treatment. Consequently, there continues to be high mortality from perinatally acquired HIV, and the ambitious goals of 90% of infected children identified, 90% of identified children treated and 90% of treated children with sustained virologic suppression by 2020 seem far beyond our reach. The objective of this commentary is to review recent advances in the field of HIV diagnosis in infants and describe how these advances may overcome long-standing barriers to access to testing and treatment.

**Discussion:**

Several innovative approaches to EID have recently been described. These include point-of-care testing, use of SMS printers to connect the central laboratory and the health facility through a mobile phone network, expanding paediatric testing to other entry points where children access the health system and testing HIV-exposed infants at birth as a rapid way to identify *in utero* infection. Each of these interventions is discussed here, together with the opportunities and challenges associated with scale-up. Point-of-care testing has the potential to provide immediate results but is less cost-effective in settings where test volumes are low. Virological testing at birth has been piloted in some countries to identify those infants who need urgent treatment, but a negative test at birth does not obviate the need for additional testing at six weeks. Routine testing of infants in child health settings is a useful strategy to identify exposed and infected children whose mothers were not enrolled in programmes for the prevention of mother-to-child transmission. Facility-based SMS printers speed up the return of laboratory results and may be of value for other testing services apart from HIV infant diagnosis.

**Conclusions:**

New tools and strategies for HIV infant diagnosis could have a significant positive impact on the identification and retention of HIV-infected infants. In order to be most effective, national programmes should carefully consider which ideas to implement and how best to integrate novel strategies into existing systems. There is no single solution that will work everywhere. Rather, a number of approaches need to be considered and should be linked in order to achieve the greatest impact on the continuum of care from testing to treatment.

## Introduction

The ambitious new global targets to identify 90% of all people living with HIV, treat 90% of those identified as infected and achieve virologic suppression in 90% of treated individuals by 2020 [1] represent a bold and timely call to identify new infections, promote sustained efforts to retain patients in care and reduce the morbidity and mortality associated with HIV infection. These so-called 90–90–90 targets have also been proposed for children in order to improve overall treatment coverage, which at 32% still lags behind the 40% for adults [2], and to reduce the high, early mortality of paediatric HIV, which peaks at age three to four months [3,4] and approaches 50% by two years of age [5].

Scale-up of services for early infant diagnosis (EID) is an essential step to address this problem, and some countries – especially those in Eastern and Southern Africa – have achieved remarkable success in this regard. In South Africa, for example, the decade between 2002 and 2012 saw a 100-fold increase in the number of EID tests performed. Data from the National Health Service Laboratory showed that in 2012 almost 75% of all HIV-exposed infants received their first EID test by two months of age [6]. The global statistics, however, are less encouraging. In the 22 Global Plan priority countries, only 50% of exposed infants received an EID test in the first two months of life during 2014 [7]. Moreover, it is estimated that of the infants identified as infected less than 35% are then referred for care and started on treatment [8], far from the goal of 90% tested and 90% treated.

There are inherent constraints associated with the current EID testing technologies that contribute to these poor performance statistics. The commercial platforms in use today require high-level facilities and highly trained technicians, which means that they can only be employed in well-resourced laboratories. Typically, a handful of such laboratories in each country serve many hundreds of service delivery sites, which are linked by specimen transport systems. Even in the best of circumstances, it can take four weeks for results to be returned to sites and in some settings it can take two months or more [9]. The purpose of this commentary is to review recent advances in EID that may help to overcome current constraints and to describe how these innovations may address some of the long-standing issues that have hampered access to testing and treatment.

## Discussion

Four innovations have recently emerged that may speed up identification and improve retention of infected infants to get us closer to 90% tested and 90% treated. Point-of-care (POC) EID tests and the use of SMS printers for result delivery are both examples of innovative tools that can decrease the turnaround time for results, potentially decreasing the time to initiation of antiretroviral therapy (ART) and reducing loss to follow-up. Expanding entry points for infant diagnosis beyond prevention of mother-to-child transmission (PMTCT) programmes could help to identify previously unrecognized HIV-exposed infants and “recapture” infants that have been lost to follow-up from the PMTCT continuum. In addition, virological testing at birth could detect *in utero* infected infants early and, with good linkage to care and prompt ART initiation, prevent mortality in such high-risk newborns. Figure 1 illustrates the testing and treatment cascade for HIV-infected infants and shows how these four innovative approaches could help to close the gap to achieve the 90% targets.

**Figure 1:** Estimated annual proportion of infected infants tested by two months of age in 2014 and estimated proportion of identified infected infants treated. Gap A represents the difference between infants currently receiving early infant diagnosis and the testing target of 90% of all positives, while Gap B represents the difference between infants treated and the treatment target of 90% of identified positives. Data sources: *Estimated number of new child infections in 2014 and estimated coverage of early infant diagnosis testing in 2014 taken from Global AIDS Response Progress Report, WHO, Geneva 2015 [7]. ^#^Proportion of infected infants receiving treatment from Chatterjee et al. *BMC Public Health*, 2011 [8].

### Point of care EID

Rapid HIV antibody assays have been used successfully to decentralize HIV testing and facilitate widespread access to ART for adults. The key to this success is ease of use by non-laboratory personnel, thermostability of test supplies, low cost, high sensitivity and results within minutes at the point of care. In children under 18 months of age, however, antibody testing cannot be used for a definitive diagnosis due to the presence of maternal antibodies. To address this issue, a number of molecular technologies are in the pipeline for POC infant diagnosis and recently two of these, the Alere™ q HIV-1/2 Detect (Alere q) and the Cepheid Xpert^®^ HIV-1 (Xpert) assays, were approved by European regulators as *in vitro* diagnostic assays (CE-IVD) [10,11]. Both are polymerase chain reaction (PCR)-based technologies that provide results within 50 minutes (Alere q) to 90 minutes (Xpert) using “closed system” proprietary cartridges that contain all the constituent components for the PCR. Both have the potential of being able to quantify HIV viral load (VL), although the currently available version of Alere q does not have VL capabilities. The Xpert instrument can also be used to detect *Mycobacterium tuberculosis* (MTb) and identify rifampin resistance in MTb.

The Alere q runs one specimen at a time and can be used at primary health care facilities. It may also offer potential for mobile testing due to its portability (7.8 kg), ease of use and flexible power source options, including on-board battery. Alere q also requires small blood volumes (25 µL) and the test cartridge can be loaded from an infant heel-prick. The Xpert platform is more suited to district and regional hospitals due to the requirement for a constant power supply; however, this assay can be used with whole blood as well as dried blood spots (DBS). Although DBS use requires trained staff and additional laboratory equipment such as a thermomixer, it does make it possible to use the Xpert platform in a laboratory that is receiving DBS samples from several sites. Unlike Alere q, the Xpert device is modular and available in different sizes, which offers a range of throughput options for simultaneous specimen testing. The most commonly used four-module version can process 16 tests a day (assuming four specimens at a time and four test runs per eight-hour shift). The Xpert platform has been in use for several years for tuberculosis (TB) testing, and field experience has confirmed its suitability for higher level facilities [12]. Importantly, both instruments have built-in wireless connectivity, which can be used to track instrument utilization, supply chain, quality assurance and operator performance.

In comparative analyses, both assays perform well against the gold-standard laboratory-based PCR. Independent field evaluations of a prototype of the Alere q machine in Mozambique [13] and South Africa [14] showed sensitivity/specificity of 98.5/99.9% and 94.1/100%, respectively. Manufacturer-led laboratory evaluations of a prototype of the Xpert test using whole blood and DBS showed sensitivity of 95.6 to 98.2% and specificity of 98 to 98.5%, respectively. Independent field evaluations using the CE-IVD marked versions of the assays are currently underway and should be completed by the end of 2015. A small number of other POC technologies for EID are in the pipeline, with expected commercial availability in 2016.

POC EID testing offers great promise but comes with some limitations. The cost per test is higher than conventional laboratory-based PCR, although this may be offset in part by the fact that infants are more likely to actually get their results when using POC so the cost per test received may be comparable to laboratory-based PCR. Although test throughput may change as technologies evolve, appropriate placement of POC instruments should carefully consider current limits. There are very few EID collection sites that require more than 8 to 16 tests per day (the maximum throughput of the Alere q and Xpert platforms, respectively), so POC EID devices could be prioritized for placement at the largest sites in order to maximize the use of each device. It is also important to note that in the future many POC EID instruments will also have the ability to measure VL. Since the number of VL tests required will far outweigh the number of EID tests, placement of POC devices may ultimately be determined by the need for VL testing. To date, there is very little programme experience with implementing POC EID testing within health services. Practical considerations, such as how to ensure quality control, how to confirm a positive test result, when to start ART after a positive POC test and how to ensure that POC tests are captured within the national EID database, will all have to be addressed through targeted research and best practice learning from programmes. Overall, POC will likely not replace laboratory-based EID testing, especially in the short-medium term. Rather, POC EID will complement and enhance conventional approaches by offering a flexible and rapid testing approach that could be implemented by non-technical staff.

### SMS printers

Current EID testing programmes are typically built on a hub-and-spoke model of service delivery, where a small number of tertiary laboratories serve a large network of testing sites. In some countries, such as Uganda, one national laboratory provides centralized EID testing for the whole country [15]. Specimens are collected in the periphery and sent by courier or through the national post to central laboratories. Once results are available and confirmed, the laboratory sends back a paper copy of the results to the site using the same specimen transportation system. In theory this process should work well, but in practice delays are common and it is not unusual for results to take more than two months, by which time some infected infants may have died and others may be lost to follow-up. SMS technology enables results to be transmitted electronically to a small printer device at the site as soon as they are available in the laboratory. This technology has been implemented nationally in several countries including Kenya, South Africa, Mozambique, Zimbabwe, Rwanda and Zambia. A recent systematic review comparing turnaround time in paper-based versus SMS systems showed that SMS printers shorten the time until results are available by an average of 17 days [16]. For SMS printers to be deployed, there must be a cellular network through which data can be transmitted; although cellular services are now available almost everywhere, this approach may not work for sites that are especially remote. In addition, it is necessary to make an initial investment in printers at the site and computers in the laboratory, but in the long term the same system could be used to transmit any type of laboratory data.

### Expanded entry points for testing

For a number of reasons – either because HIV-positive women were never tested during antenatal care, or because they were lost from the PMTCT programme before delivery, or because they did not return with their infants for post-partum testing – 50% of HIV-exposed infants do not currently receive any EID testing in the first two months of life. However, because these infants may not have received the full complement of PMTCT interventions, they are also the ones at higher risk of acquiring HIV. In order to identify these children and bring them into care, it is important to expand infant and young child testing beyond EID in PMTCT programmes, by testing at other time points when children access the health system. This is an especially valuable strategy in high-prevalence settings but could also be of benefit in lower-prevalence settings in some circumstances. A recent systematic review found that in studies of testing of hospitalized children in Eastern/Southern Africa an average of 20.7% of all children tested were HIV-infected. In Western/Central Africa, the proportion was 10.8%. By contrast, testing in malnutrition clinics had a higher yield in Western/Central Africa compared with Eastern/Southern – 22 versus 6% [17]. The WHO has recommended provider-initiated HIV testing and counselling (PITC) in paediatric care settings for many years; however, few countries have implemented this guidance at the national level. The evidence suggests that using a universal testing approach for children admitted to hospitals or malnutrition centres would be a very successful approach for identifying HIV-infected and -exposed children and should be adopted widely. At present, EID is often offered as part of a specialized package of care that is seen as distinct and separate from routine infant and child health services. Integration of EID as a service provided at immunization clinics and well-child clinics may help to increase both uptake and follow-up, especially in countries with a high burden of HIV.

### Virological testing at birth

The high mortality of HIV in infants is driven in large part by the especially high risk of death among infants infected *in utero* [18]. Virological testing at birth has high specificity (99.7%) but low overall sensitivity (67.8%) due in large part to the fact that viral nucleic acids may not be detected at birth in infants who have acquired HIV through intra-partum transmission [19]. However, virological testing at birth testing should identify most infants who are infected through *in utero* transmission (although the precise impact of maternal antiretrovirals [ARVs] on the ability to detect *in utero* infection at birth is not known). If one of the goals of EID is to prevent infant mortality, then identifying and treating *in utero* infection as early as possible (with virological testing at birth linked to early treatment) may be a useful clinical strategy and one where POC EID could serve as a valuable tool to ensure that rapid action is taken to refer infants for ART once a newborn is confirmed positive. At the same time, it is important to recognize that for such an approach to be effective, a number of other elements need to be in place. Rates of facility delivery would have to be high enough to capture a significant proportion of infected newborns. Alternatively, for infants delivered at home, there would have to be a robust mechanism to find and test exposed babies in the first days of life, either through community outreach or by testing at BCG immunization, which is often administered three days after birth. Providers would need to be trained not only in testing neonates, but also in treating them. National procurement would have to be modified to buy more syrup ARV formulations, as the currently available Fixed Dose Combinations (FDCs) do not contain the correct dosing ratios for neonatal treatment. Moreover, we need additional research on what regimens to use in newborns, especially preterm and low birth weight infants, as there is a paucity of data on the dosing and safety of ARVs in this population. It will be critically important to develop and evaluate a testing approach that defines which infants might benefit most from a virological test at birth and how to manage a positive birth test. For infants that test negative at birth, close follow-up, tracking and repeat testing will be essential to retest at six weeks or soon after.

## Conclusions

Innovations in the tools available to perform EID may have a positive impact on the identification, retention and survival of HIV-infected infants and children. At the same time, the practical challenges of implementing strategies such as virological testing at birth and POC EID may hamper wide-scale adoption.

Where virological testing at birth and POC EID *are* implemented, they should be integrated with complementary strategies to enable infants to be followed across the continuum of care from testing through to treatment. For example, while POC testing will enable immediate identification of infected infants, having a test result in hand does not solve the problem of ensuring linkage to care for infected infants – especially if paediatric ARVs and staff trained in prescribing ART are not available. Elements of an integrated approach include the following: task shifting of paediatric testing to enable more providers to test children (including training a cadre of staff for POC testing); mentoring of clinicians and improved registers to identify early loss to follow-up of infants; robust “leak-proof” mechanisms to refer infected infants and children for treatment; training and equipping clinicians with the knowledge, tools and commodities they need to treat infected infants; and systems to continuously monitor the quality of testing as well as retention and impact on child survival.

In order to achieve the 90–90–90 goal for children, we cannot rely on any one-size-fits-all solution. Rather, we must develop context-specific, integrated, multifaceted approaches that involve identification and treatment of pregnant HIV-positive women as early as possible, prevention of loss to follow-up for mother–infant pairs, provision of timely paediatric diagnosis using technologies that are best suited to the setting where they are deployed and robust linkage of infected infants and children to lifelong care and treatment. It is important to note that not all improvements need novel technological solutions. In many cases, simple interventions, such as strengthening formal record-keeping and introducing systems to track mothers and infants who fail to return for follow-up visits, may increase retention within existing infrastructures. Finally, it is vital to recognize that community engagement to ensure acceptability and uptake of testing is a key enabler to achieve the greatest impact [20]. Without buy-in from communities of pregnant women and mothers living with HIV, even the most sophisticated of tools will not address the high rate of loss to follow-up and mortality and poor access to treatment for children.

## References

[1] 90–90–90: an ambitious treatment target to help end the AIDS epidemic.

[2] How AIDS changed everything – MDG6: 15 years, 15 lessons of hope from the AIDS response.

[3] Bourne DE, Thompson M, Brody LL, Cotton M, Draper B, Laubscher R (2009). Emergence of a peak in early infant mortality due to HIV/AIDS in South Africa. AIDS.

[4] Innes S, Lazarus E, Otwombe K, Liberty A, Germanus R, Van Rensburg AJ (2014). Early severe HIV disease precedes early antiretroviral therapy in infants: are we too late?. J Int AIDS Soc.

[5] Newell ML, Coovadia H, Cortina-Borja M, Rollins N, Gaillard P, Dabis F (2004). Mortality of infected and uninfected infants born to HIV-infected mothers in Africa: a pooled analysis. Lancet.

[6] Sherman GG, Lilian RR, Bhardwaj S, Candy S, Barron P (2014). Laboratory information system data demonstrate successful implementation of the prevention of mother-to-child transmission programme in South Africa. S Afr Med J.

[7] World Health Organization (2014). Global update on the health sector response to HIV, 2014.

[8] Chatterjee A, Tripathi S, Gass R, Hamunime N, Panha S, Kiyaga C (2011). Implementing services for early infant diagnosis (EID) of HIV: a comparative descriptive analysis of national programs in four countries. BMC Public Health.

[9] Sutcliffe CG, van Dijk JH, Hamangaba F, Mayani F, Moss WJ (2014). Turnaround time for early infant HIV diagnosis in rural Zambia: a chart review. PLoS One.

[10] http://www.prnewswire.com/news-releases/alere-receives-ivd-ce-mark-for-alere-q-hiv-12-detect-assay-for-molecular-diagnosis-of-hiv-at-the-point-of-care-300042878.html.

[11] http://ir.cepheid.com/releasedetail.cfm?ReleaseID=906875.

[12] Piatek AS, Van Cleeff M, Alexander H, Coggin WL, Rehr M, Van Kampen S (2013). GeneXpert for TB diagnosis: planned and purposeful implementation. Glob Health Sci Pract.

[13] Jani IV, Meggi B, Mabunda N, Vubil A, Sitoe NE, Tobaiwa O (2014). Accurate early infant HIV diagnosis in primary health clinics using a point-of-care nucleic acid test. J Acquir Immune Defic Syndr.

[14] Hsiao N-Y, Kroon M, Dunning L, Myer L (2015). Evaluation of the Alere q for point-of-care early infant HIV diagnosis in South Africa [CROI Abstract 34]. In Special Issue: Abstracts From the 2015 Conference on Retroviruses and Opportunistic Infections. Top Antivir Med.

[15] Kiyaga C, Sendagire H, Joseph E, McConnell I, Grosz J, Narayan V (2013). Uganda's new national laboratory sample transport system: a successful model for improving access to diagnostic services for early infant HIV diagnosis and other programs. PLoS One.

[16] Vojnov Systematic review manuscript in preparation.

[17] Cohn Systematic review manuscript in preparation.

[18] Marston M, Becquet R, Zaba B, Moulton LH, Gray G, Coovadia H (2011). Net survival of perinatally and postnatally HIV-infected children: a pooled analysis of individual data from sub-Saharan Africa. Int J Epidemiol.

[19] Lilian RR, Kalk E, Bhowan K, Berrie L, Carmona S, Technau K (2012). Early diagnosis of in utero and intrapartum HIV infection in infants prior to 6 weeks of age. J Clin Microbiol.

[20] Penazzato M, Revill P, Prendergast AJ, Collins IJ, Walker S, Elyanu PJ (2014). Early infant diagnosis of HIV infection in low-income and middle-income countries: does one size fit all?. Lancet Infect Dis.

# Sub article: The last and first frontier – emerging challenges for HIV treatment and prevention in the first week of life with emphasis on premature and low birth weight infants

**DOI:** 10.7448/IAS.18.7.20271

**Published:** 2015-12-02

**Authors:** Mark F Cotton, Sandi Holgate, Aurelie Nelson, Helena Rabie, Catherine Wedderburn, Mark Mirochnick

**Affiliations:** 1 Division of Paediatric Infectious Diseases, Department of Paediatrics and Child Health, Faculty of Medicine and Health Sciences, Tygerberg Children's Hospital, Stellenbosch University, Stellenbosch, South Africa; 2 Division of Neonatology, Department of Paediatrics and Child Health, Faculty of Medicine and Health Sciences, Tygerberg Children's Hospital, Stellenbosch University, Stellenbosch, South Africa; 3 Médecins Sans Frontières, Khayelitsha, South Africa; 4 Department of Pediatrics, Boston University School of Medicine, Boston, MA, USA

**Keywords:** premature, low birth weight, small for gestation, antiretrovirals, management

## Abstract

**Introduction:**

There is new emphasis on identifying and treating HIV in the first days of life and also an appreciation that low birth weight (LBW) and preterm delivery (PTD) frequently accompany HIV-related pregnancy. Even in the absence of HIV, PTD and LBW contribute substantially to neonatal and infant mortality. HIV-exposed and -infected infants with these characteristics have received little attention thus far. As HIV programs expand to meet the 90-90-90 target for ending the HIV pandemic, attention should focus on newborn infants, including those delivered preterm or of LBW.

**Discussion:**

In high prevalence settings, infant diagnosis of HIV is usually undertaken after the neonatal period. However, as *in utero* infection may be diagnosed at birth, earlier initiation of therapy may limit viral replication and prevent early damage. Globally, there is growing awareness that preterm and LBW infants constitute a substantial proportion of births each year. Preterm infants are at high risk for vertical transmission. Feeding difficulties, apnoea of prematurity and vulnerability to sepsis occur commonly. Feeding intolerance, a frequent occurrence, may compromise oral administration of medications. Although there is growing experience with post-exposure prophylaxis for HIV-exposed term newborn infants, there is less experience with preterm and LBW infants. For treatment, there are even fewer options for preterm infants. Only zidovudine has adequate dosing recommendations for treating term and preterm infants and has an intravenous formulation, essential if feeding intolerance occurs. Nevirapine dosing for prevention, but not treatment, is well established for both term and preterm infants.

HIV diagnosis at birth is likely to be extremely stressful for new parents, more so if caring for preterm or LBW infants. Programs need to adapt to support the medical and emotional needs of young infants and their parents, where interventions may be lifesaving.

**Conclusions:**

New focus is required for the newborn baby, including those born preterm, with LBW or small for gestational age to consolidate gains already made in early diagnosis and treatment of young children.

## Introduction

The Joint United Nations Program on HIV/AIDS (UNAIDS) was established in 1996 to lead global efforts for universal access to prevention, treatment, care and support [1]. There has been much progress, with increased access to care, reduction in vertical transmission and reversal of mortality trends. In 2014, UNAIDS released the 90-90-90 policy document, which defines new global targets to consolidate gains and ultimately end the pandemic. The aims are as follows: identifying HIV in 90% of the infected population, of whom 90% should be placed on combination antiretroviral therapy (cART), of whom 90% should be virally suppressed [2].

Newborn infants are an important target group that includes the most neglected population of all – those with low birth weight (LBW) and/or preterm delivery (PTD). Collectively, these infants are the last frontier for HIV management in terms of knowledge and experience. Paradoxically, as they are developmentally the youngest and most fragile population, they also represent the first frontier to be encountered. We comment on the current state of knowledge, areas requiring increased attention under the present circumstances and research. We also seek to heighten awareness in healthcare workers of challenges and opportunities in addressing the needs of young newborn infants, including PTD and LBW infants.

## Discussion

### HIV infection and the newborn infant

Early infant diagnosis leading to prompt initiation of effective cART can prevent postnatal HIV disease progression. Until recently, infant diagnosis focused on those between four and six weeks of age after preliminary data from the Children with HIV Early Antiretroviral (CHER) trial results were released in 2007. This study showed that commencing antiretroviral therapy (ART) at a median of 7.4 weeks of age reduced mortality by 76% and HIV disease progression by 75% in infants with a baseline CD4% ≥25%. However, less well appreciated was data suggesting rapid disease progression preceding ART initiation in the trial. Over the three to four weeks between diagnosis and study entry, seven HIV-infected infants (1.25% of those identified) had already died, 16 (2.8%) had developed signs of advanced HIV disease and almost 20% had a CD4% <25% [3]. These observations, during this short period, suggest that seven weeks of age for ART initiation is too late for many. In a subsequent study from the two CHER trial sites between 2007 and 2010, 62% of 250 infants already had advanced HIV disease when ART was initiated by 12 weeks of age, again illustrating rapid disease progression in early infancy [4]. Impetus for a birth PCR (in addition to a PCR later on) is the awareness that most *in utero* infection can be detected on the first day of life, which could allow earlier linkage to care and less attrition [5].

Infants most at risk for acquiring HIV and who could benefit from both enhanced prophylaxis and early diagnosis are those whose mothers either seroconverted late in pregnancy or had not attained viral suppression on ART. Under these circumstances, there is both a need for early diagnosis to establish infection status as soon as possible after birth and to establish enhanced post-exposure prophylaxis for the infants. These numbers can be substantial. For example, in South Africa, where a rapid antibody test is repeated at week 32 and in labour in pregnant women who initially tested negative, 3.3% of mothers had acute seroconversion, accounting for 26% of vertically infected infants in a public program [6]. PTD and being small for gestational age (SGA) are risk factors for intrapartum HIV transmission, probably because of shorter exposure to antenatal cART and more immature mucosal barriers [7–9].

For infants whose mothers were diagnosed with HIV late in pregnancy, the best possible post-exposure prophylaxis is required, accompanied by caregiver counselling on prophylaxis administration to avoid medication errors. A prophylaxis regimen comprising zidovudine plus three doses of nevirapine is now recommended for infants at high risk for vertical transmission [10,11]. It is also essential to continue prophylaxis in infants whose mothers may be failing ART. In many settings, it is standard practice to introduce cART as post-exposure prophylaxis when there is a risk for vertical transmission [12,13]. Although there is no evidence of enhanced prevention, using cART for prevention will then continue, should baseline tests confirm infection.

**Table 1:** ARVs available for term infants in first four weeks of life and for preterm newborn infants

	Term	Preterm	Comments
Reverse transcriptase inhibitors			
Zidovudine	X	X	IV formulation available
Lamivudine	X		Dosage available for first month
Emtricitabine	X		Dosage from 0 to 3 months
Stavudine	X		
Non-nucleoside reverse transcriptase inhibitors			
Nevirapine	X	X	Dosages available for prevention only
Protease inhibitors			
Lopinavir-ritonavir	X (Recommended only after 2 weeks of age and 42 weeks post-conception)		If used earlier than 42 weeks post-conception age, therapeutic drug monitoring and clinical and cardiac monitoring recommended

From Ref. [11].

Early cART initiation within the first few hours may have benefits beyond preventing HIV disease complications. The report of the Mississippi baby who spent almost two years without detectable plasma viremia after starting cART at 31 hours of age suggests that early therapy could limit HIV reservoir size, which may have a dramatic impact on the lifelong course of HIV infection [14,15]. The experience with the Mississippi baby has increased motivation for a birth PCR and cART for both prophylaxis and treatment in newborn infants [15].

#### Low birth weight, premature and SGA infants

LBW is defined as a birth weight below 2500 g. Its global prevalence is 15.5%, accounting for about 20 million infants born each year, 96.5% of them in developing countries [16]. Contributing factors are prematurity, defined as birth before 37 completed weeks of gestation, and being SGA, defined as a birth weight below the 10th centile for gestational age. PTD accounted for 11.1% or 14.9 million babies in 2010 and is the second most common cause of mortality below five years of age, after pneumonia. Most PTDs occur after 32 weeks of gestation [17]. In a survey from East Africa comprising more than 5000 live births, 9.2% were LBW and 4% were PTD. Compared to term delivery, gestational age below 34 weeks gestation had a 58-fold higher death rate. Risk of death was threefold higher for gestational age between 34 and 36 weeks, but if also SGA mortality was 20-fold higher [18]. In a recent South African report of neonates requiring intensive care in Pretoria, 68% were LBW, with 8% weighing below 1000 g, 24% between 1000 and 1499 g, and 24% between 1500 and 2000 g [19]. In an audit of HIV-exposed preterm infants at a tertiary neonatal unit in Cape Town between 2010 and 2011, 3.3% had a birth weight below 1000 g and were significantly more HIV exposed than those with a higher birth weight. Of 51 HIV-exposed infants, mean birth weight was 834 g and mean gestational age was 28.3 weeks. Most were born by caesarean section before active labour, with a transmission rate of 2.7% by six weeks of age [20].

PTD and LBW rates are increasing every year [21]. In 2010, 59% of SGA newborn infants were born at term and 41% were preterm [22]. Infectious causes of SGA include maternal malaria, varicella and syphilis [23]. Maternal tuberculosis, prevalent in many settings where HIV is common, contributes substantially to prematurity and LBW [24,25]. Placental causes include abruption and infarcts; other maternal causes include hypertension, diabetes or exposure to tobacco, alcohol or recreational drugs [23]. HIV infection and ART contribute to LBW, PTD and SGA [26].

Infant consequences of PTD include hypothermia, hypoglycaemia, respiratory distress, apnoea, intraventricular haemorrhage and sepsis and may complicate HIV diagnosis and treatment. Very premature infants may require parenteral nutrition and tube feeding is necessary below around 34 weeks gestation. Gastro-oesophageal reflux occurs commonly putting the infant at risk for apnoea and aspiration [27]. Necrotizing enterocolitis (NEC), a potentially lethal complication often necessitating periods without enteral feeding, is more common in HIV-infected premature infants [28]. Breastfeeding is especially important to prevent NEC [29]. SGA infants have additional risks of polycythaemia and neurological dysfunction related to hypoxia [30].

#### Antiretrovirals for neonates

Neonates have significant differences in physiology that impact drug disposition, so that neonatal drug absorption, distribution, metabolism and elimination differ from that in older infants and children. Neonatal differences in drug disposition are even greater in premature infants [31]. Antiretrovirals (ARVs) cannot be safely and effectively used in neonates without being directly studied in these most vulnerable populations. Immaturity in gastrointestinal tract function, the unique diet of the young infant, developmental changes in drug transporter systems and the need for special formulations due to the inability to swallow pills all impact drug absorption. Maturational changes in body size and composition, as well as plasma protein concentrations, will change drug distribution with age [32]. Activity of enzymes critical for drug elimination such as CYP3A4 and UGT2B6 are lower in neonates than older infants and lower in preterm than term infants. The pattern of maturation in activity is specific for each enzyme and isoform, with adult values generally reached by later childhood or early adolescence [31,32].

Although cART regimens should be an essential component of neonatal post-exposure prophylaxis and treatment regimens, the paucity of relevant and appropriate neonatal pharmacokinetic and safety data makes their use in neonates and young infants difficult. Twenty-five ARVs have US Food and Drug Administration (FDA) approval for children. Only six are approved for newborn term infants, and one of these drugs (lopinavir) is not recommended until 42 weeks post-conception (See Table 1). Zidovudine, stavudine, lamivudine and emtricitabine have adequate pharmacokinetic data and oral formulations for the first two weeks of life in term infants [11,33,34]. Zidovudine is the only ARV with sufficient pharmacokinetic data from premature infants to allow development of dosing regimens to ensure effective yet not toxic plasma concentrations [35]. For infants unable to tolerate enteral feeding and requiring parenteral drug administration, zidovudine is also the only ARV with an intravenous formulation.

While there are several studies describing nevirapine pharmacokinetics in neonates and young infants, these studies were designed for prophylactic dosing regimens to maintain trough concentrations above 0.1 µg/mL [36]. Nevirapine regimens for successful treatment of HIV infection require trough concentrations above 3.0 µg/mL, but there are no pharmacokinetic studies of nevirapine in neonates with the goal of achieving the treatment concentrations. Nevirapine is metabolized mainly by CYP2B6 and CYP3A4, whose activity is low in term neonates. The nevirapine prophylaxis studies have shown that nevirapine clearance is low in neonates and even lower in preterm or growth-retarded infants [36,37]. In adults, nevirapine auto-induces its own clearance but the extent of auto-induction on immature enzyme systems is unknown. For infants co-infected with *Mycobacterium tuberculosis* and requiring rifampicin co-treatment, nevirapine clearance is also enhanced in African children [38]. Thus, there are fewer antiretroviral options for co-infected newborn infants.

Raltegravir, the first commercially available integrase inhibitor, illustrates many challenges and delays faced in establishing safe and effective ARV dosing regimens for neonates. The FDA approved raltegravir for adults in 2007, for children aged 2 to 12 years in 2011 and for infants four weeks to two years in 2013. However, safety and pharmacokinetic data for neonates are still lacking. Raltegravir is easily transported across the placenta and commonly included in antenatal cART regimens where mothers are on second-line therapy or have not attained virologic suppression towards delivery. Neonatal washout of transplacentally acquired raltegravir is variable and prolonged during the first days of life, as expected since raltegravir is eliminated by UGT1A1, the same enzyme that metabolizes bilirubin and whose activity is very low immediately after birth [39]. An *in vitro* study has shown that at extremely high concentrations (50 to 100 times greater than those seen in adults receiving usual treatment doses), raltegravir displaces bilirubin from albumin [40]. These data suggest that a hyperbilirubinaemic newborn, especially if PTD or LBW, receiving raltegravir could be at increased risk for kernicterus from the displacement of bilirubin from albumin, as was seen with sulfisoxazole [41,42]. Raltegravir dosing for neonates will have to be carefully designed to avoid accumulation to potentially dangerous plasma concentrations, especially when neonates are premature and have LBW. While raltegravir would be of great benefit for use in neonates for prevention or treatment, the rigorous safety and pharmacokinetic studies needed before it can be used safely in term and preterm newborns have yet to be conducted.

Lopinavir-ritonavir is available as a liquid formulation containing 42.4% alcohol by volume and 15.3% propylene glycol by weight and volume. The FDA received a report of 10 neonatal cases of severe renal, metabolic and cardiac toxicities with a single fatality after being given this lopinavir-ritonavir liquid formulation. After this report, a recommendation was issued to avoid lopinavir-ritonavir until two weeks of age and 42 weeks post-conception [43]. It is not known whether these toxicities were caused by excessive exposure to alcohol, propylene glycol and/or lopinavir. However, there are some data to guide dosage in term neonates and premature and LBW infants, should the potential benefit be thought to outweigh the risk [44,45]. The FDA recently approved a lopinavir-ritonavir pellet formulation for children below three years of age for procurement in high prevalence countries. Although it does not contain ethanol or propylene glycol and may be safer for infants, there is concern that newborn infants may not be able to safely swallow the pellets [46].

#### Programmatic and diagnostic challenges in newborn infants

Mothers identified as HIV positive late in pregnancy and at delivery face a very stressful period, learning about their own status and the real possibility that their newborn infants may be HIV infected all in a short space of time. Resources to facilitate counselling in this period are essential. There is growing emphasis on the benefits of breastfeeding HIV-exposed infants in low-income settings and ensuring that mothers continue ARVs in the postpartum period. Feeding and weight monitoring are particularly important in premature and LBW infants. In many hospital settings, flash heating or pasteurization may be used to inactivate HIV in breast milk, while retaining nutritional value [47].

In many settings where HIV is prevalent, newborn infants weighing above 1800 g are already sent home, if stable. Therefore, such LBW infants are seen in primary care facilities such as in Khayelitsha, South Africa, where 28% of HIV-exposed infants delivered were LBW or premature (internal data). Simplified guidelines on PCR testing of HIV-exposed infants (high risk infants versus universal testing for all HIV-exposed infants), indications for up-referral of clinically unwell premature HIV-exposed infants, clear weight-based algorithms on neonatal ART dosages and routine laboratory monitoring all require consideration in primary care settings.

Preliminary data from birth PCR testing of HIV-exposed infants in Khayelitsha indicated a high acceptance of mothers (99%) for testing at delivery. Standardized counselling sessions, including information on repeat testing for neonates negative at birth, were introduced. Counselling sessions were adapted for mother-infant pairs to support cART adherence and appropriate dosing, together with frequent clinical reviews during the first months of life [48]. Neonatal ART is feasible and promotes retention in care when accompanied by good counselling, complemented by disclosure to a treatment supporter [49].

## Conclusions

Neonates including LBW and PTD form a special risk group, requiring increased recognition in the efforts to reach the 90-90-90 targets. Early diagnosis shortly after birth requires planning and resources. There is an urgent need for safe and effective cART regimens. Use of these drugs in this population will require rigorous pharmacokinetic and safety studies and a supportive clinical environment. While several studies through the International Maternal Pediatric and Adolescent AIDS Clinical Trials (IMPAACT) network (P1097, P1106 and P1115) are beginning to address some of the therapeutic gaps in the first weeks of life and in premature infants, these efforts are insufficient. Delays between diagnosis and therapy while waiting for infants to reach a physiological maturity sufficient to allow dosing based on our current knowledge base cannot be justified in term infants and may be dangerous for premature infants.

## References

[1] UNAIDS (2008). The first 10 years.

[2] UNAIDS (2014). 90-90-90 – an ambitious treatment target to help end the AIDS epidemic.

[3] Violari A, Cotton MF, Gibb DM, Babiker AG, Steyn J, Madhi SA (2008). Early antiretroviral therapy and mortality among HIV-infected infants. N Engl J Med.

[4] Innes S, Lazarus E, Otwombe K, Liberty A, Germanus R, Van Rensburg AJ (2014). Early severe HIV disease precedes early antiretroviral therapy in infants: are we too late?. J Int AIDS Soc.

[5] Lilian RR, Kalk E, Technau KG, Sherman GG (2013). Birth diagnosis of HIV infection in infants to reduce infant mortality and monitor for elimination of mother-to-child transmission. Pediatr Infect Dis J.

[6] Dinh TH, Delaney KP, Goga A, Jackson D, Lombard C, Woldesenbet S (2015). Impact of maternal HIV seroconversion during pregnancy on early mother to child transmission of HIV (MTCT) measured at 4–8 weeks postpartum in South Africa 2011–2012: a national population-based evaluation. PLoS One.

[7] Kuhn L, Abrams EJ, Matheson PB, Thomas PA, Lambert G, Bamji M (1997). Timing of maternal-infant HIV transmission: associations between intrapartum factors and early polymerase chain reaction results. New York City Perinatal HIV Transmission Collaborative Study Group. AIDS.

[8] Kuhn L, Steketee RW, Weedon J, Abrams EJ, Lambert G, Bamji M (1999). Distinct risk factors for intrauterine and intrapartum human immunodeficiency virus transmission and consequences for disease progression in infected children. Perinatal AIDS Collaborative Transmission Study. J Infect Dis.

[9] Warszawski J, Tubiana R, Le Chenadec J, Blanche S, Teglas JP, Dollfus C (2008). Mother-to-child HIV transmission despite antiretroviral therapy in the ANRS French Perinatal Cohort. AIDS.

[10] Nielsen-Saines K, Watts DH, Veloso VG, Bryson YJ, Joao EC, Pilotto JH (2012). Three postpartum antiretroviral regimens to prevent intrapartum HIV infection. New Engl J Med.

[11] Guidelines for the use of antiretroviral agents in pediatric HIV infection.

[12] Chiappini E, Galli L, Giaquinto C, Ene L, Goetghebuer T, Judd A (2013). Use of combination neonatal prophylaxis for the prevention of mother-to-child transmission of HIV infection in European high-risk infants. AIDS.

[13] Bitnun A, Samson L, Chun TW, Kakkar F, Brophy J, Murray D (2014). Early initiation of combination antiretroviral therapy in HIV-1-infected newborn infants can achieve sustained virologic suppression with low frequency of CD4+ T-cells carrying HIV in peripheral blood. Clin Infect Dis.

[14] Persaud D, Gay H, Ziemniak C, Chen YH, Piatak M, Chun TW (2013). Absence of detectable HIV-1 viremia after treatment cessation in an infant. N Engl J Med.

[15] Luzuriaga K, Gay H, Ziemniak C, Sanborn KB, Somasundaran M, Rainwater-Lovett K (2015). Viremic relapse after HIV-1 remission in a perinatally infected child. N Engl J Med.

[16] Care of the preterm and/or low-birth-weight newborn.

[17] Blencowe H, Cousens S, Oestergaard MZ, Chou D, Moller A-B, Narwal R (2012). National, regional, and worldwide estimates of preterm birth rates in the year 2010 with time trends since 1990 for selected countries: a systematic analysis and implications. Lancet.

[18] Marchant T, Willey B, Katz J, Clarke S, Kariuki S, ter Kuile F (2012). Neonatal mortality risk associated with preterm birth in East Africa, adjusted by weight for gestational age: individual participant level meta-analysis. PLoS Med.

[19] Lloyd LG, de Witt TW (2013). Neonatal mortality in South Africa: how are we doing and can we do better?. S Afr Med J.

[20] Tooke L, Horn AR, Harrison MC (2013). HIV transmission to extremely low birth weight infants. Pediatr Infect Dis J.

[21] Blencowe H, Cousens S, Chou D, Oestergaard M, Say L, Moller AB (2013). Born too soon: the global epidemiology of 15 million preterm births. Reprod Health.

[22] Lee AC, Katz J, Blencowe H, Cousens S, Kozuki N, Vogel JP (2013). National and regional estimates of term and preterm babies born small for gestational age in 138 low-income and middle-income countries in 2010. Lancet Glob Health.

[23] Approach to the child with IUGR/SGA.

[24] Pillay T, Khan M, Moodley J, Adhikari M, Coovadia H (2004). Perinatal tuberculosis and HIV-1: considerations for resource-limited settings. Lancet Infect Dis.

[25] Bekker A, Du Preez K, Schaaf HS, Cotton MF, Hesseling AC (2012). High tuberculosis exposure among neonates in a high tuberculosis and human immunodeficiency virus burden setting. Int J Tuberc Lung Dis.

[26] Chen JY, Ribaudo HJ, Souda S, Parekh N, Ogwu A, Lockman S (2012). Highly active antiretroviral therapy and adverse birth outcomes among HIV-infected women in Botswana. J Infect Dis.

[27] Behrman RE, Butler AS (2007). Preterm birth: causes, consequences, and prevention.

[28] Desfrere L, de Oliveira I, Goffinet F, El Ayoubi M, Firtion G, Bavoux F (2005). Increased incidence of necrotizing enterocolitis in premature infants born to HIV-positive mothers. AIDS.

[29] Section on Breastfeeding (2012). Breastfeeding and the use of human milk. Pediatrics.

[30] Tenovuo A (1988). Neonatal complications in small-for-gestational age neonates. J Perinat Med.

[31] Edginton AN, Schmitt W, Voith B, Willmann S (2006). A mechanistic approach for the scaling of clearance in children. Clin Pharmacokinet.

[32] Ku LC, Smith PB (2015). Dosing in neonates: special considerations in physiology and trial design. Pediatr Res.

[33] Mirochnick M, Nielsen-Saines K, Pilotto JH, Pinto J, Veloso VG, Rossi S (2011). Nelfinavir and Lamivudine pharmacokinetics during the first two weeks of life. Pediatr Infect Dis J.

[34] Moodley D, Pillay K, Naidoo K, Moodley J, Johnson MA, Moore KH (2001). Pharmacokinetics of zidovudine and lamivudine in neonates following coadministration of oral doses every 12 hours. J Clin Pharmacol.

[35] Mirochnick M, Capparelli E, Dankner W, Sperling RS, van Dyke R, Spector SA (1998). Zidovudine pharmacokinetics in premature infants exposed to human immunodeficiency virus. Antimicrob Agents Chemother.

[36] de Waal R, Kroon SM, Holgate SL, Horn AR, Tooke LJ, Norman J (2014). Nevirapine concentrations in preterm and low birth weight HIV-exposed infants: implications for dosing recommendations. Pediatr Infect Dis J.

[37] Mugabo P, Els I, Smith J, Rabie H, Smith P, Mirochnick M (2011). Nevirapine plasma concentrations in premature infants exposed to single-dose nevirapine for prevention of mother-to-child transmission of HIV-1. S Afr Med J.

[38] Oudijk JM, McIlleron H, Mulenga V, Chintu C, Merry C, Walker AS (2012). Pharmacokinetics of nevirapine in HIV-infected children under 3 years on rifampicin-based antituberculosis treatment. AIDS.

[39] Bosma PJ, Seppen J, Goldhoorn B, Bakker C, Oude Elferink RP, Chowdhury JR (1994). Bilirubin UDP-glucuronosyltransferase 1 is the only relevant bilirubin glucuronidating isoform in man. J Biol Chem.

[40] Clarke DF, Wong RJ, Wenning L, Stevenson DK, Mirochnick M (2013). Raltegravir in vitro effect on bilirubin binding. Pediatr Infect Dis J.

[41] Silverman WA, Andersen DH, Blanc WA, Crozier DN (1956). A difference in mortality rate and incidence of kernicterus among premature infants allotted to two prophylactic antibacterial regimens. Pediatrics.

[42] Ahlfors CE (2000). Unbound bilirubin associated with kernicterus: a historical approach. J Pediatr.

[43] Boxwell D, Cao K, Lewis L, Marcus K, Nikhar B (2011). Neonatal toxicity of Kaletra oral solution – LPV, ethanol, or propylene glycol?.

[44] Holgate S, Rabie H, Smith P, Cotton MF (2012). Trough lopinavir concentrations in preterm human immunodeficiency virus-infected infants. Pediatr Infect Dis J.

[45] Urien S, Firtion G, Anderson ST, Hirt D, Solas C, Peytavin G (2011). Lopinavir/ritonavir population pharmacokinetics in neonates and infants. Br J Clin Pharmacol.

[46] Klein R (2015). Approved and tentatively approved antiretrovirals in association with the president's emergency plan.

[47] Israel-Ballard K, Chantry C, Dewey K, Lonnerdal B, Sheppard H, Donovan R (2005). Viral, nutritional, and bacterial safety of flash-heated and pretoria-pasteurized breast milk to prevent mother-to-child transmission of HIV in resource-poor countries: a pilot study. J Acquir Immune Defic Syndr.

[48] Nelson A, Maritz J, Giddy J, Frigati L, Rabie H, Van Cutsem G (2015). HIV testing and antiretroviral therapy initiation at birth: views from a primary care setting in Khayelitsha. S Afr J HIV Med.

[49] Gourlay A, Birdthistle I, Mburu G, Iorpenda K, Wringe A (2013). Barriers and facilitating factors to the uptake of antiretroviral drugs for prevention of mother-to-child transmission of HIV in sub-Saharan Africa: a systematic review. J Int AIDS Soc.

# Sub article: Sequencing paediatric antiretroviral therapy in the context of a public health approach

**DOI:** 10.7448/IAS.18.7.20265

**Published:** 2015-12-02

**Authors:** Ragna S Boerma, T Sonia Boender, Michael Boele van Hensbroek, Tobias F Rinke de Wit, Kim CE Sigaloff

**Affiliations:** 1 Amsterdam Institute for Global Health and Development and Department of Global Health, Academic Medical Center of the University of Amsterdam, The Netherlands; 2 Global Child Health Group, Emma Children's Hospital/Academic Medical Center of the University of Amsterdam, The Netherlands; 3 Division of Infectious Diseases, Department of Internal Medicine, Academic Medical Center, University of Amsterdam, The Netherlands

**Keywords:** paediatric HIV, antiretroviral therapy, HIV drug resistance, protease inhibitor, non-nucleoside reverse transcriptase inhibitor, low- and middle-income countries

## Abstract

**Introduction:**

As access to prevention of mother-to-child transmission (PMTCT) efforts has increased, the total number of children being born with HIV has significantly decreased. However, those children who do become infected after PMTCT failure are at particular risk of HIV drug resistance, selected by exposure to maternal or paediatric antiretroviral drugs used before, during or after birth. As a consequence, the response to antiretroviral therapy (ART) in these children may be compromised, particularly when non-nucleoside reverse transcriptase inhibitors (NNRTIs) are used as part of the first-line regimen. We review evidence guiding choices of first- and second-line ART.

**Discussion:**

Children generally respond relatively well to ART. Clinical trials show the superiority of protease inhibitor (PI)- over NNRTI-based treatment in young children, but observational reports of NNRTI-containing regimens are usually favourable as well. This is reassuring as national guidelines often still recommend the use of NNRTI-based treatment for PMTCT-unexposed young children, due to the higher costs of PIs. After failure of NNRTI-based, first-line treatment, the rate of acquired drug resistance is high, but HIV may well be suppressed by PIs in second-line ART. By contrast, there are currently no adequate alternatives in resource-limited settings (RLS) for children failing either first- or second-line, PI-containing regimens.

**Conclusions:**

Affordable salvage treatment options for children in RLS are urgently needed.

## Introduction

The treatment of HIV-1 in children is more challenging than treatment of adults and is associated with an increased risk of virological failure. Children are vulnerable to developing HIV drug resistance due to various reasons, such as variability in pharmacokinetics, limited paediatric treatment options and lack of adherence support [1]. Moreover, drug exposure as part of the prevention of mother-to-child transmission (PMTCT) can lead to pre-treatment drug resistance [2–4], thus diminishing the chance of treatment success.

Clinical trials have found that children under three years of age on protease inhibitor (PI)-based, antiretroviral therapy (ART) experience less virological failure and death than children on non-nucleoside reverse transcriptase inhibitor (NNRTI)-based regimens, both in PMTCT-exposed and -unexposed children [5–7]. The World Health Organization (WHO) therefore recommends all children below three years of age to receive a PI-based regimen [lopinavir/ritonavir (LPV/r)], regardless of history of PMTCT exposure [8]. Unfortunately, despite these recommendations, the use of PIs for young children in low- and middle-income countries (LMIC) in routine programmes is limited due to practical barriers. PIs are more costly than NNRTIs, and infant formulations were, until recently, only available as a liquid that requires refrigeration [7,9,10].

In this commentary, we will compare PI-based versus NNRTI-based, first-line ART for children, and also discuss feasible ART sequencing approaches in children.

## Discussion

More than half of HIV-infected children who do not receive treatment are estimated to die before the age of two years [11]. ART dramatically reduces morbidity and mortality in HIV-infected children of all ages. Findings of previous systematic reviews are encouraging as up to 70 to 80% of children achieve virological suppression after 12 months of first-line treatment [12,13]. In young children under three years of age, data from clinical trials and observational studies in resource-limited settings (RLS) show that, on average, the HIV suppression rate is sustained around 60 to 70% up to 24 months after treatment initiation (Figure 1, Table 1).

**Figure 1:** Summary estimates of virological suppression in children <3 years in LMIC, 6 to 24 months after first-line treatment initiation for NNRTI- and PI-treated children. Random effects meta-analysis was conducted using a Freeman–Tukey arcsine square root transformation to stabilize proportions. No virological suppression rates were available for PI-treated children after 18 and 24 months. NNRTI, non-nucleoside reverse transcriptase inhibitor; PI, protease inhibitor.

**Table 1:** Studies reporting virological suppression rates in children <3 years on first-line ART 6–24 months after treatment initiation

Study	Median year of treatment initiation	Regimen	Total number of patients	Number of patients with viral suppression	% children with virological suppression	Time after treatment initiation
Lockman 2007^a^ [2]	2001	NNRTI-based	12	11	91.7	6 months
Lockman 2007^b^	2001	NNRTI-based	11	1	9.1	6 months
Puthanakit 2009 [14]	2004	NNRTI-based	25	14	56.0	6 months
Germanaud 2010 [15]	2007	NNRTI-based	68	43	63.2	6 months
Van Dijk 2011 [16]	2008	NNRTI-based	96	85	88.5	6 months
Cotton 2013 [17]	2006	PI-based	230	192	83.5	6 months
Romano Mazzotti 2009 [18]	Not reported	PI-based	56	21	37.5	6 months
Technau 2014 [19]	2006	PI-based	2612	1763	67.5	6 months
Lindsey 2014^a^ [20]	2008	NNRTI-based	116	86	74.1	6 months
Lindsey 2014^a^	2008	PI-based	124	112	90.3	6 months
Lindsey 2014^b^	2008	NNRTI-based	68	55	80.1	6 months
Lindsey 2014^b^	2008	PI-based	71	67	94.4	6 months
Meyers 2011 [21]	2006	PI-based	617	323	52.4	6 months
Lockman 2007^b^	2001	NNRTI-based	11	10	90.9	12 months
Lockman 2007^a^	2001	NNRTI-based	10	1	10.0	12 months
Jaspan 2008 [22]	2004	PI-based	85	60	70.6	12 months
Jaspan 2008	2004	NNRTI-based	115	47	40.9	12 months
Prendergast 2008 [23]	2004	PI-based	49	44	89.8	12 months
Puthanakit 2009	2004	NNRTI-based	24	19	79.2	12 months
Van Dijk 2011	2008	NNRTI-based	77	68	88.3	12 months
Romano Mazzotti 2009	Not reported	PI-based	56	30	53.6	12 months
Soeters 2014 [24]	2011	PI-based	118	61	51.7	12 months
Technau 2014	2006	PI-based	2165	1595	73.7	12 months
Puthanakit 2009	2004	NNRTI-based	19	16	84.2	18 months
Van Dijk 2011	2008	NNRTI-based	53	46	86.8	18 months
Kay2012 [25]	2007	NNRTI-based	34	19	55.9	18 months
Lockman 2007^b^	2001	NNRTI-based	9	1	11.1	24 months
Lockman 2007^a^	2001	NNRTI-based	11	9	81.8	24 months
Puthanakit 2009	2004	NNRTI-based	15	14	93.3	24 months
Van Dijk 2011	2008	NNRTI-based	27	21	77.8	24 months
Musiime 2014 [26]	2011	NNRTI-based	349	294	84.2	24 months

a PMTCT-unexposed cohort;

b PMTCT-exposed cohort.

NNRTI, non-nucleoside reverse transcriptase inhibitor; PI, protease inhibitor.

### NNRTI- versus PI-based, first-line ART

Based on data from clinical trials [5,6,27], the WHO has moved to recommending PI-based, first-line ART for all children below three years, regardless of previous PMTCT exposure. Comparison of trials and observational data reveals higher rates of virological suppression among children receiving PI-based regimens (Figure 1). However, data on PI-based, first-line treatment in children are still scarce, compared to NNRTI-based treatment, and most available PI data are from clinical trials with a relatively short follow-up period. The P1060 trial, a multicentre trial conducted in South Africa, Zimbabwe, Zambia, Malawi, Uganda, Tanzania and India, compared 288 children up to three years of age treated with AZT and 3TC combined with either NVP or LPV/r using the primary end point of treatment failure or discontinuation after 24 weeks. Both among PMTCT-exposed and -unexposed children, significantly more children reached the primary end point in the NVP group compared to the LPV/r group: 40.8 versus 19.3% (*p*<0.001) [5,6]. By contrast, a study performed in South Africa by Teasdale *et al*. [28] reported 27% virological failure among children after 24 weeks of first-line, PI-based treatment. The higher failure rate in this cohort may be because children received either ritonavir-boosted lopinavir (LPV/r) or full-dose ritonavir, which is associated with diminished virological response and the emergence of major protease mutations [29]. Programmatic data, as have to date been reported mainly from South Africa [21,30], will be very valuable in assessing whether the favourable virological suppression rates reported by trials can be achieved in routine ART programmes.

Most data on the effectiveness of NNRTI-based, first-line treatment are from programmatic settings. A retrospective cohort of 202 children starting NNRTI-based, first-line treatment in Thailand reported that 33 (16%) children had virological failure in the first year of treatment [31]. Children on NVP-based treatment were 3.3 times more likely to develop failure compared to children on EFV-based treatment. This study found no difference between young children with and without previous PMTCT exposure: 1 out of 4 and 4 out of 16 children, respectively, developed virological failure during the study period [31]. Two studies from sub-Saharan Africa show concordant results. Lowenthal *et al*. [32] describe a cohort study in Botswana with five years of follow-up including 804 children starting on EFV- or NVP-based, first-line treatment. The virological failure rate was 6.7% after one year, 10.2% after two years and 12.8% after five years of follow-up on EFV-based treatment, and 12.8, 19.8 and 25.1%, respectively, for NVP-based treatment [32]. In a Zambian cohort, 198 ART-naive and mostly PMTCT-unexposed children started either NVP- or EFV-based treatment. Six to twenty-four months after treatment initiation, the virological failure rate increased from 11.5 to 22.2% [16].

Interpretation of the differences between PI- and NNRTI-treated children is limited by the heterogeneity of studies in terms of design, study participants and setting. It is difficult to draw firm conclusions on the benefits of PI over NNRTI treatment in programmatic settings, especially in PMTCT-unexposed young children. However, results from randomized controlled trials have convincingly shown the superiority of PI- over NNRTI-based treatment [5,6], and PI-based treatment should be implemented for all HIV-infected children under three years of age, as recommended by the WHO [8]. The outcomes of observational studies reporting on programmatic data remain relevant, because the dispensation of PIs may be influenced by financial and logistical issues. LPV/r, currently the only PI combination available for children, is at least five times more expensive than EFV or NVP [33]. Recently, the United States Food and Drug Administration approved LPV/r in pellet form for paediatric usage, which, in contrast to the up-to-now only available LPV/r syrup, does not require refrigeration [10]. This is an important step towards increased access to PI treatment for children in LMIC.

### HIV-TB coinfection

Tuberculosis (TB) is one of the most common co-infections affecting children with HIV, and cotreatment occurs in up to one-third of children [21]. Comedication for TB adds significant complexity to the treatment of children who also require or are already receiving ART. For children on LPV/r-based regimens, guidelines suggest to add ritonavir to achieve the full therapeutic dose [8]. An alternative is to change to a triple NRTI regimen [34] or to substitute NVP for LPV/r [8]. Children on NVP- or EFV-based ART can usually continue the same regimen (ensuring that NVP dose is 200 mg/m^2^) or can also be changed to a triple NRTI regimen. These changes in the ART regimen, as well as simultaneous use of TB drugs, put children at risk of developing drug toxicity, virological failure [21] and HIV drug resistance [35].

### Development of resistance on first-line therapy

Virological failure is defined by the WHO as two consecutive measurements of plasma viral load >1000 cps/mL after at least six months of treatment [8]. However, WHO definitions have changed over time and studies have reported different virological cut-offs to define failure. A systematic review of resistance data in children from resource-poor settings found that 90% of those failing first-line regimens had at least one HIV drug-resistance mutation, with mutations increasing in frequency with duration of treatment [36]. This review included mostly cross-sectional studies and included children who were treated with suboptimal regimens.

More recent studies also show high rates of HIV drug resistance among children with treatment failure. In a study conducted in the Central African Republic, 83 and 85% of children on first-line therapy with a detectable viral load after 18 months had NRTI and NNRTI mutations, respectively. The most prevalent NRTI mutations were M184V (73%), T69D/N/S (17%), L74I/V (8%), K65R (8%) and Q151M (2%), and the most prevalent NNRTI mutations were Y181C (44%), K103H/N/S (39%), K101E/P (39%), G190A (30%) and A98G/S (19%) [37].

In Thai children treated with NVP- or EFV-containing therapy, NRTI mutations were found in 89% of children at the time of virological failure, with M184V/I (85%), K65R (11%) and K219Q/E (8%) being the most prevalent. NNRTI mutations were detected in 97% of the children, of which Y181C/I (58%), K103N (34%), G190S/A (18%) and V108I (13%), were most common [31].

It is clear from these studies that children who fail NNRTI-based, first-line regimens, generally report similarly high rates of NNRTI- and NRTI-associated mutations, with the Y181C and M184V mutations being among the most prevalent mutations within the respective drug classes. Accumulated NRTI resistance can have consequences for the construction of an effective, second-line, PI-based regimen, in which NRTIs are used as the backbone. This implies that a timely switch to second-line ART after failure is warranted, to prevent clinical consequences as well as the accumulation of drug resistance. Timely switching is, however, challenged by lack of virological monitoring in RLS. Reluctance of clinicians to change therapy in children, for whom limited drug options are available, may be an additional barrier.

In a European study, the development of both PI and NRTI resistance among children failing first-line, PI-based regimens was negligible [38]. In RLS, there are few reports of acquired protease mutations on first-line treatment. A recent South African study found that 8 out of 75 (10.7%) children with virological failure on a first-line PI had LPV/r mutations [39]. Within the NRTI drug class, the M184V and thymidine analogue mutations were found in seven out of eight and two out of eight children, respectively. Data among adults have shown that with intensified adherence support, viral load resuppression on PI-based ART is possible, despite drug resistance [40]. In this study, performed in Khayelitsha, South Africa, two-third of participants resuppressed within three months while remaining on PI-based regimens. The consequences of this study obviously extend to children receiving PIs; intensive adherence counselling should be offered before switching.

### Second-line ART

As per WHO recommendation, failure of an NNRTI-based regimen is followed by switching to a boosted PI plus two NRTIs. There are limited data about the response to second-line ART in children [41]. A recent study from Thailand reported on 111 children among whom the risk of virological failure 24 months after second-line initiation was 41% [42]. Children with longer duration of first-line ART were at higher risk of second-line failure. The latter suggests that continued first-line failure may have led to the accumulation of NRTI mutations, diminishing the response to subsequent second-line therapy. However, in the study's multivariate analysis, resistance to NRTIs did not appear as a risk factor for failure.

For children for whom a PI-based, first-line regimen has failed, NNRTIs remain the only new drug class that can be introduced. However, potential re-emergence of archived NNRTI mutations may limit the effectiveness of this ART sequencing approach. Moreover, NNRTIs have a much lower genetic barrier for resistance [43], and without the protection of an effective NRTI backbone (due to acquired resistance), NNRTI resistance will rapidly emerge. Recently, the first reports on the outcome of second-line NNRTI in children have been published. One small study from South Africa found that six months after regimen change, the proportion with virological failure was 75% (6 out of 8) in children receiving NNRTI-based second-line versus 20% (13 out of 66) in children on PI-based second-line [44]. A second study, again from South Africa, reported on 12 children who were switched to NNRTI-based therapy. Of these, 8 out of 12 (67%) did not achieve virological suppression [39]. Although these findings are based on a small number of children, it is apparent that NNRTI-based, second-line ART is not an optimal choice and is expected to have limited durability.

### Salvage options

Constructing third-line regimens using novel, robust drugs such as darunavir, raltegravir or dolutegravir, may be possible for children. Studies have demonstrated the efficacy of darunavir in heavily ART-experienced patients [45]. In a UK cohort, even in children with prolonged PI exposure, resistance to darunavir was rare [46]. Darunavir could therefore be an option after failure of first-line, LPV/r-based treatment in children above three years of age. Raltegravir is the first integrase inhibitor approved for paediatric usage (>4 weeks of age) and has been evaluated in the IMPAACT P1066 trial, showing virological suppression (<400 cps/mL) in approximately 80% of participants after 48 weeks of follow-up [47]. In adults, co-administration of rifampicin decreases raltegravir concentrations, thereby potentially limiting the efficacy of this drug in children with HIV-TB coinfection [48]. Dolutegravir, an integrase strand transfer inhibitor with a very favourable resistance profile, has to date only been approved in children >12 years of age. Results of two cohorts of the IMPAACT 1093 trial have been presented in an abstract form and showed virological suppression in 17 out of 23 treatment-experienced adolescents (aged 12 to 18 years) after 48 weeks of treatment with dolutegravir, and in 9 out of 11 treatment-experienced children (aged 6 to 12 years) after 24 weeks of treatment [49,50]. These newer antiretroviral agents, however, are currently unavailable in RLS. Substantial cost-reduction and/or generic production of these drugs are vital to ensure salvage options for children failing PI-based regimens.

## Conclusions

Despite the challenges of paediatric antiretroviral treatment, especially in RLS, studies have shown relatively high rates of virological suppression in children on first-line treatment. For young children, randomized controlled trials have shown the superiority of PI- over NNRTI-based treatment. Observational studies, however, also report favourable results of NNRTI-based, first-line treatment. This has important implications for settings in which PI treatment is unavailable due to logistic and financial barriers. Unquestionably, early initiation of treatment is vital and should be prioritized even if NNRTIs are the only obtainable drugs.

After NNRTI-based, first-line treatment failure, the rates of acquired drug resistance among children are strikingly high. However, these children are likely to still benefit from PIs in second-line. By contrast, the development of resistance mutations after failure of PI-based first-line is limited. If children do have continued failure on first-line LPV/r, the chances of resuppression after switching to second-line NNRTI are very low. Suitable formulations of additional PIs are urgently needed for children who fail either first- or second-line LPV/r. Darunavir boosted with ritonavir would be a suitable candidate, but it is not widely available. Newer antiretroviral agents including second-generation NNRTIs and integrase inhibitors should also be evaluated. The future of an increasing number of children will depend on the availability of these salvage medications. To make these regimens accessible on a global scale, low-cost generic drugs or major price reductions of patented versions are necessary.

## References

[1] Havens PL, Gibb DM (2007). Increasing antiretroviral drug access for children with HIV infection. Pediatrics.

[2] Lockman S, Shapiro RL, Smeaton LM, Wester C, Thior I, Stevens L (2007). Response to antiretroviral therapy after a single, peripartum dose of nevirapine. N Engl J Med.

[3] Musoke PM, Barlow-Mosha L, Bagenda D, Mudiope P, Mubiru M, Ajuna P (2009). Response to antiretroviral therapy in HIV-infected Ugandan children exposed and not exposed to single-dose nevirapine at birth. J Acquir Immune Defic Syndr.

[4] Sigaloff KCE, Calis JC, Geelen SP, van Vugt M, de Wit TF (2011). HIV-1-resistance-associated mutations after failure of first-line antiretroviral treatment among children in resource-poor regions: a systematic review. Lancet Infect Dis.

[5] Violari A, Lindsey JC, Hughes MD, Mujuru HA, Barlow-Mosha L, Kamthunzi P (2012). Nevirapine versus ritonavir-boosted lopinavir for HIV-infected children. N Engl J Med.

[6] Palumbo P, Lindsey JC, Hughes MD, Cotton MF, Bobat R, Meyers T (2010). Antiretroviral treatment for children with peripartum nevirapine exposure. N Engl J Med.

[7] Penazzato M, Prendergast AJ, Muhe L, Tindyebwa D, Abrams E (2014). Optimisation of antiretroviral therapy in HIV-infected children under 3 years of age (Review). Cochrane Database Syst Rev.

[8] World Health Organization (WHO) (2013). Consolidated guidelines on the use of antiretroviral drugs for treating and preventing HIV infection.

[9] Fitzgerald F, Penazzato M, Gibb D (2013). Development of antiretroviral resistance in children with HIV in low- and middle-income countries. J Infect Dis.

[10] Cipla Cipla announces US FDA approval for the world's first paediatric lopinavir and ritonavir oral pellets for the treatment of AIDS in infants and young children [Internet]. Press release.

[11] Newell M-L, Coovadia H, Cortina-Borja M, Rollins N, Gaillard P, Dabis F (2004). Mortality of infected and uninfected infants born to HIV-infected mothers in Africa: a pooled analysis. Lancet.

[12] Sutcliffe CG, van Dijk JH, Bolton C, Persaud D, Moss WJ (2008). Effectiveness of antiretroviral therapy among HIV-infected children in sub-Saharan Africa. Lancet Infect Dis.

[13] Ciaranello AL, Chang Y, Margulis AV, Bernstein A, Bassett IV, Losina E (2009). Effectiveness of pediatric antiretroviral therapy in resource-limited settings: a systematic review and meta-analysis. Clin Infect Dis.

[14] Puthanakit T, Aurpibul L, Sirisanthana T, Sirisanthana V (2009). Efficacy of non-nucleoside reverse transcriptase inhibitor-based highly active antiretroviral therapy in Thai HIV-infected children aged two years or less. Pediatr Infect Dis. J.

[15] Germanaud D, Derache A, Traore M, Madec Y, Toure S, Dicko F (2010). Level of viral load and antiretroviral resistance after 6 months of non-nucleoside reverse transcriptase inhibitor first-line treatment in HIV-1-infected children in Mali. J Antimicrob Chemother.

[16] Van Dijk JH, Sutcliffe CG, Munsanje B, Sinywimaanzi P, Hamangaba F, Thuma PE (2011). HIV-infected children in rural Zambia achieve good immunologic and virologic outcomes two years after initiating antiretroviral therapy. PLoS One.

[17] Cotton MF, Violari A, Otwombe K, Panchia R, Dobbels E, Rabie H (2013). Early time-limited antiretroviral therapy versus deferred therapy in South African infants infected with HIV: results from the children with HIV early antiretroviral (CHER) randomised trial. Lancet.

[18] Romano-Mazzotti L, Sifuentes-Vela C, Villalobos-Acosta P, Santos-Preciado J, Pavia-Ruz N (2009). Respuesta discordante a terapia antirretroviral altamente activa en pacientes pediátricos mexicanos infectados con VIH/SIDA. Bol Med Hosp Infant Mex.

[19] Technau K-G, Schomaker M, Kuhn L, Moultrie H, Coovadia A (2014). Virologic response in children treated with abacavir compared with stavudine-based antiretroviral treatment – a South African multi-cohort analysis. Pediatr Infect Dis J.

[20] Lindsey JC, Hughes MD, Violari A, Eshleman SH, Abrams EJ, Bwakura-Dangarembizi M (2014). Predictors of virologic and clinical response to nevirapine versus lopinavir/ritonavir-based antiretroviral therapy in young children with and without prior nevirapine exposure for the prevention of mother-to-child HIV transmission. Pediatr Infect Dis J.

[21] Meyers TM, Yotebieng M, Kuhn L, Moultrie H (2011). Antiretroviral therapy responses among children attending a large public clinic in Soweto, South Africa. Pediatr Infect Dis J.

[22] Jaspan HB, Berrisford AE, Boulle AM (2008). Two-year outcomes of children on non-nucleoside reverse transcriptase inhibitor and protease inhibitor regimens in a South African pediatric antiretroviral program. Pediatr Infect Dis J.

[23] Prendergast A, Mphatswe W, Tudor-Williams G, Rakgotho M, Pillay V, Thobakgale C (2008). Early virological suppression with three-class antiretroviral therapy in HIV-infected African infants. AIDS.

[24] Soeters HM, Napravnik S, Patel MR, Eron JJ, Van Rie A (2014). The effect of tuberculosis treatment on virologic and CD4+cell count response to combination antiretroviral therapy: a systematic review. AIDS.

[25] Kay J, Wanzira H, Sandison T, Kakuru A, Bigira V, Kamya M (2012). Virologic suppression in nevirapine-exposed HIV-infected infants initiating antiretroviral therapy in rural Uganda. J Trop Pediatr.

[26] Musiime V, Kekitiinwa A, Mulenga V, Cook A, Abongomera G, Thomason M CHAPAS 3: A randomised trial comparing stavudine vs zidovudine vs abacavir as NRTI backbone in NNRTIbased first-line ART in 478 HIV-infected children in Uganda and Zambia.

[27] Penazzato M, Prendergast A, Tierney J, Cotton M, Gibb D (2012). Effectiveness of antiretroviral therapy in HIV-infected children under 2 years of age (Review). Cochrane Database Syst Rev.

[28] Teasdale CA, Abrams EJ, Coovadia A, Strehlau R, Martens L, Kuhn L (2013). Adherence and viral suppression among infants and young children initiating protease inhibitor-based antiretroviral therapy. Pediatr Infect Dis J.

[29] Van Zyl GU, van der Merwe L, Claassen M, Cotton MF, Rabie H, Prozesky HW (2009). Protease inhibitor resistance in South African children with virologic failure. Pediatr Infect Dis J.

[30] Davies M-A, Keiser O, Eley B, Rabie H, van Cutsem G, Giddy J (2009). Outcomes of the South Africa National Antiretroviral Treatment Programme for children: the IeDEA Southern Africa collaboration. South Afr Med J.

[31] Jittamala P, Puthanakit T, Chaiinseeard S, Sirisanthana V (2009). Predictors of virologic failure and genotypic resistance mutation patterns in thai children receiving non-nucleoside reverse transcriptase inhibitor-based antiretroviral therapy. Pediatr Infect Dis J.

[32] Lowenthal ED, Ellenberg JH, Machine E, Sagdeo A, Boiditswe S, Steenhoff AP (2013). Association between efavirenz-based compared with nevirapine-based antiretroviral regimens and virological failure in HIV-infected children. JAMA.

[33] Clinton Health Access Initiative Antiretroviral (ARV) ceiling price list [Internet]. Antiretrovir Ceil. PRICE List.

[34] Kekitiinwa A, Cook A, Nathoo K, Mugyenyi P, Nahirya-Ntege P, Bakeera-Kitaka S (2013). Routine versus clinically driven laboratory monitoring and first-line antiretroviral therapy strategies in African children with HIV (ARROW): a 5-year open-label randomised factorial trial. Lancet.

[35] Rossouw TM, Feucht UD, Melikian G, van Dyk G, Thomas W, du Plessis NM (2015). Factors associated with the development of drug-resistance mutations in HIV-1 infected children failing protease inhibitor-based antiretroviral therapy in South Africa. PLoS One.

[36] Sigaloff KCE, Calis JCJ, Geelen SP, van Vugt M, de Wit TFR (2011). HIV-1-resistance-associated mutations after failure of first-line antiretroviral treatment among children in resource-poor regions: a systematic review. Lancet Infect Dis.

[37] Charpentier C, Gody J, Mbitikon O, Moussa S, Matta M, Pere H (2012). Virological response and resistance profiles after treatment: a cross-sectional evaluation in HIV type 1-infected children living in the Central African Republic. AIDS Res Hum Retroviruses.

[38] Babiker A, Castro nee Green H, Compagnucci A, Fiscus S, Giaquinto C, Gibb DM (2011). First-line antiretroviral therapy with a protease inhibitor versus non-nucleoside reverse transcriptase inhibitor and switch at higher versus low viral load in HIV-infected children: an open-label, randomised phase 2/3 trial. Lancet Infect Dis.

[39] Meyers T, Sawry S, Wong JY, Moultrie H, Pinillos F, Fairlie L (2015). Virologic failure among children taking lopinavir/ritonavir-containing first-line antiretroviral therapy in South Africa. Pediatr Infect Dis J.

[40] Garone DB, Conradie K, Patten G, Cornell M, Frontières MS, Town C (2013). High rate of virological re-suppression among patients failing second-line antiretroviral therapy following enhanced adherence support?: a model of care in Khayelitsha, South Africa. South Afr Med J.

[41] Ajose O, Mookerjee S, Mills EJ, Boulle A, Ford N (2012). Treatment outcomes of patients on second-line antiretroviral therapy in resource-limited settings. AIDS.

[42] Suaysod R, Ngo-Giang-Huong N, Salvadori N, Cressey TR, Kanjanavanit S, Techakunakorn P (2015). Treatment failure in HIV-infected children on second-line protease inhibitor-based antiretroviral therapy. Clin Infect Dis.

[43] Van den Vijver D, Wensing A, Angarano G, Asjo B, Balotta C, Boeri E (2006). The calculated genetic barrier for antiretroviral drug resistance substitutions is largely similar for different HIV-1 subtypes. J Acquir Immune Defic Syndr.

[44] Zanoni BC, Sunpath H, Feeney ME (2012). Pediatric response to second-line antiretroviral therapy in South Africa. PLoS One.

[45] Violari A, Bologna R, Kumarasamy N, Pilotto JH, Hendrickx A, Kakuda TN (2015). Safety and efficacy of darunavir/ritonavir in treatment-experienced pediatric patients. Pediatr Infect Dis J.

[46] Donegan KL, Walker AS, Dunn D, Judd A, Pillay D, Menson E (2012). The prevalence of darunavir-associated mutations in HIV-1-infected children in the UK. Antivir Ther.

[47] Nachman S, Zheng N, Acosta EP, Teppler H, Homony B, Graham B (2014). Pharmacokinetics, safety, and 48-week efficacy of oral raltegravir in HIV-1-infected children aged 2 through 18 years. Clin Infect Dis.

[48] Eley BS, Meyers T (2011). Antiretroviral therapy for children in resource-limited settings. Pediatr Drugs.

[49] Viani RM, Alvero C, Fenton T, Acosta E, Hazra R, Gara EO Safety and efficacy of dolutegravir in HIV treatment-experienced adolescents: 48-week results.

[50] Viani RM, Alvero C, Fenton T, Acosta E, Hazra R, Gara EO Safety, pharmacokinetics and efficacy of dolutegravir in treatment-experienced HIV+children.

# Sub article: Optimizing drugs to reach treatment targets for children and adolescents living with HIV

**DOI:** 10.7448/IAS.18.7.20270

**Published:** 2015-12-02

**Authors:** Martina Penazzato, Janice Lee, Edmund Capparelli, Shaffiq Essajee, Nathan Ford, Atieno Ojoo, Fernando Pascual, Nandita Sugandhi, Marc Lallemant

**Affiliations:** 1 HIV Department, World Health Organization, Geneva, Switzerland; 2 Drugs for Neglected Diseases Initiative, Geneva, Switzerland; 3 Clinical pharmacology, University of California, San Diego, CA, USA; 4 UNICEF, Copenhagen, Denmark; 5 Medicine Patent Pool, Geneva, Switzerland; 6 Clinton Health Access Initiative, Boston, MA, USA

**Keywords:** antiretrovirals, treatment, children, ART, optimization, formulations, HIV

## Abstract

**Introduction:**

As the global community makes progress towards the 90-90-90 targets by 2020, a key challenge is ensuring that antiretroviral drugs for children and adolescents are suitable to the context of resource-limited settings. Drug optimization aims to support the expanded use of more simplified, less toxic drug regimens with high barriers to drug resistance that require minimal clinical monitoring while maintaining therapeutic efficacy. This manuscript summarizes the progress made and outlines further critical steps required to ensure that the right drugs are available to start children and adolescents on treatment and to keep them virologically suppressed.

**Discussion:**

Building upon previous work in drug optimization, several important steps were taken in 2014 to ensure alignment between WHO dosing recommendations and the requirements of regulatory bodies, to accelerate drug development, to reduce intellectual property barriers to generic production of combined formulations and rationalize drug selection in countries. The priority for the future is to improve access to antiretroviral therapy (ART) at the two ends of the paediatric age spectrum – infants and adolescents – where the treatment gap is greatest, and optimize drug sequencing with better use of available medicines for second- and third-line ART. Future efforts in this area will require continuous collaboration and coordination, and the promotion of innovative approaches to accelerate access to new drugs and formulations.

**Conclusions:**

While significant progress has been made, additional efforts are needed to ensure that treatment targets are reached by 2020.

## Introduction

The world has recently achieved the global treatment target of reaching 15 million people in antiretroviral therapy (ART) by 2015 and countries are now moving ahead towards reaching the new 90-90-90 targets [1]. However, improvements in access to treatment have been greater for adults than for children. There are many reasons for this, but a fundamental challenge is the enduring complexity of treatment regimens, particularly for young children. For adolescents, high rates of non-adherence to treatment and loss to care point to the need to prioritize simpler and more tolerable regimens.

Paediatric HIV is largely a disease affecting children in resource-limited settings [2], and as a result children have not benefited from the same level of engagement in drug development as adults. Research and development (R&D) of new drugs for children has historically been complex and time consuming, with drug approvals for children lagging years behind the approval for adults – up to nine years in the case of tenofovir (TDF), for example [3]. Without dedicated efforts, the situation can be expected to get worse. The reduction in new infections resulting from the rapid expansion of more effective interventions to prevent mother-to-child transmission with 220,000 new infections occurring in 2014 [4] has reduced the paediatric market and further dis-incentivized manufacturers to invest in paediatric antiretroviral (ARV) R&D.

Unfortunately, current estimates suggest that despite increasingly successful implementation of PMTCT interventions over 1 million children will still be in need of treatment in 2020 [5]. This paper summarizes some of the major milestones and advances in treatment optimization for children and adolescents, and aims to outline the critical steps needed to ensure that the right drugs are available so that children and adolescents can start on treatment promptly and maintain virological suppression.

## Discussion

### The path towards drug optimization

Early efforts to optimize treatment for patients in resource-limited settings focused on the development of nevirapine (NVP)-based fixed-dose combinations (FDCs) in adults. The development of these FDCs, with no pre-existing FDA or EMEA originator products, required an innovative regulatory framework to review these dossiers [6].

In the absence of suitable alternatives, adult FDCs were initially used to treat children, often with limited evidence regarding appropriate dosing. Building upon the early efforts by MSF and others in simplifying dosing and using weight-bands [7], WHO convened a technical meeting in 2005 to harmonize global paediatric dosing recommendations. This meeting led to the development of the WHO generic tool for assessing formulation dosing strategies using existing ARVs. Exposure targets were based primarily on the labelled dose and incorporating additional knowledge of maturational changes in drug metabolism [8]. This weight-band dosing approach for individual ARVs and FDCs was included in the WHO 2006 and 2010 guidelines for the treatment of infants and children with HIV within a public health approach [9].

The next revision of WHO treatment guidelines, released in 2013 [10], represented another milestone for drug optimization, rationalizing the number of treatment options to simplify procurement and prescribing, recommending more potent drugs for young children, and harmonizing recommendations with adult regimens down to 10 years of age. These guidelines recognized that different approaches to treat younger children were still required because of differences in the natural history of HIV infection, and differing pharmacokinetics (PK), efficacy and toxicity profiles of ARVs depending on age. However, while the WHO 2013 Guidelines have been widely adopted for adults, the implementation of paediatric drug recommendations has been slow and challenging in most countries due to the lack of age-appropriate formulations for key drugs such as LPV/r (which has an unpalatably high alcohol content and requires cold chain storage until dispensing) and TDF (which remains unaffordable for most settings and is currently only available in powder and multiple single-compound dosage forms from the originators).

Consequently, the most common ARV regimen used for children remains AZT/3TC/NVP [11]. Analysis of procurement patterns suggests that the main driver in product selection is the availability of dual or triple dispersible FDC rather than their cost or efficacy [12].

### Accelerating actions to optimize treatment for children

Building upon previous work in drug optimization (PADO 1; Figure 1), important further steps were taken in 2014 to ensure alignment between WHO dosing recommendations and the requirements of regulatory bodies, as well as to reduce intellectual property barriers and accelerate drug development and rationalize drug selection in countries [14].

**Figure 1:** Medium- and long-term priorities for drug sequencing in children [13].

#### Optimizing the use of ARVs for children

Experts from the WHO Paediatric ARV Working Group (PAWG) have provided evidence-based recommendations to guide age-appropriate dosing. The WHO generic tool was revised to reflect the non-linear relationship between weight and drug bioavailability due to organ maturation in young infants [15]. Population-based PK models incorporating the maturational changes in absorption, distribution, metabolism and clearance have been used together with simulations to assess ARV exposure (trough concentrations and AUCs) obtained when following WHO weight-band dosing recommendations. Concerns about potential under-dosing required a revision of the WHO weight-band dosing for DRV and DRV/r (Table 1). Also, a collaborative PK modelling was undertaken to establish the appropriate dosing for a new FDC including ABC/3TC/EFV. This additional modelling was undertaken in light of the high variability of EFV PK and the concerns raised about potential under-dosing observed in African trials [16] where WHO weight-band dosing was used. This has now resulted in more robust dosing recommendations that will guide the development of this FDC.

**Table 1:** PAWG-recommended weight-band dosing for DRV single compound and DRVr co-formulation to be used twice daily as part of second- or third-line regimens*

		Number of tablets/mL by weight-band morning and evening					
		
Drugs	Strength of paediatric tab (mg)	3–5.9 kg	6–9.9 kg	10–13.9 kg	14–19.9 kg	20–24.9 kg	25–29.9 kg
DRV^a^	100 mg/mL	NR	NR	2.5 (250 mg)	3.5 (350 mg)	–	–
DRV^a^	75 mg tablets	NR	NR	3 (225 mg)	5 (375 mg)	5 (375 mg)	5 (375 mg)
DRV/r	120/20*	NR	NR	2 (240 mg)	3 (360 mg)	3 (360 mg)	4 (480 mg)

a DRV single-strength dosing must be used with the appropriate dosing of RTV.

#### Developing priority medicines for children

The need to overcome intellectual property barriers that prevent individual drugs developed by different drug companies to be assembled in a potential FDC and to navigate the existing regulatory pathway for formulations which lack originator equivalent led to the establishment of the Paediatric HIV Treatment Initiative (PHTI) [17]. This initiative aims to accelerate the development of WHO-recommended paediatric ARV formulations by coordinating drug development and promoting increased access by engaging with industry to ensure sharing of intellectual property rights and know-how, and facilitate formulation development. Two drug development projects were launched: ABC/3TC/EFV and DRV/r.

#### Optimizing selection and procurement of existing paediatric products

In recent years, a number of improved ARV formulations have become available, such as dispersible FDCs in place of the traditional liquid formulations. These products have greatly simplified paediatric HIV care in low-income settings; however, the proliferation of these newer options, in addition to the availability of older sub-optimal products has resulted in a multiplicity of formulations across regimens and weight-bands. The fragmentation of demand across too many similar products has in some cases led to stock-outs as there was not enough demand for manufacturers to produce sufficient quantities of low-volume products.

For this reason, the Interagency Task Team on prevention and treatment of HIV infection in pregnant women, mothers and their children (IATT – convened by WHO and UNICEF in collaboration with multiple implementing partners) provided formulary guidance to programmes on selection of optimal paediatric ARVs. The formulary was first developed in 2011, revised in 2013 [18] using a more robust set of criteria (Table 2) and updated again in December 2014. Currently, the optimal formulary contains nine paediatric ARVs, plus a “limited use list” containing products that may be needed under special circumstances, or products that are transitioning in or out of the optimal formulary [19].

**Table 2:** Criteria for evaluation of paediatric ARV products included in IATT optimal formular [18]

Criterion	Definition	
Meets WHO requirements	•	Included in the latest WHO guidelines for paediatric treatment
Allows for widest range of dosing options	•	Allows for flexible dosing across multiple weight-bands and ages
Approved by SRA/WHO PQ	•	Availability of at least one SRA-approved product
User friendly	•	Easy for healthcare worker to prescribe
	•	Easy for caregivers to administer
	•	Supports adherence
Optimizes supply chain management	•	Easy to transport
	•	Easy to store
	•	Easy to distribute
Available for resource-limited settings	•	Product is licensed/registered for use in resource-limited settings
	•	Reliable supply of product
Comparative cost	•	Cost should not be a deciding factor; however comparative cost of formulations of the same drug/drug combination should be considered

SRA, stringent regulatory agencies; WHO PQ, WHO pre-qualification programme.

This list is endorsed by major implementers and purchasers of paediatric ARVs such as PEPFAR, UNICEF and the Global Fund, and enables consolidation of demand around optimal products. Procurement is coordinated through the Paediatric ARV procurement working group, a consortium (composed of the Global Fund, PEPFAR, CHAI and UNICEF) which pools orders from the various procurement entities (including some national governments) and schedules production with manufacturers.

### Remaining challenges and new directions

In late December 2014, WHO convened a second meeting on paediatric ARV drug optimization (PADO 2) with the goal of informing the WHO 2015 Guidelines development process, reviewing the list of mid- and long-term priorities for drug and formulations development as well as identifying additional research gaps in this area. Three main challenges were identified. First, as consideration is given to testing HIV-exposed infants at birth and more evidence is gathered on the risk and benefits of very early treatment to limit the viral reservoir [20], there is a need to identify safe and effective ARV options for newborns (less than four weeks, including premature children). A second challenge lies in identifying the most suitable regimen for adolescents who are generally at greater risk of poor adherence and consequent treatment failure and drug resistance development [21]. Finally, there is a need to improve second- and third-line options for children. The management of treatment failure is still very limited in resource-limited settings because of limited drug availability, limited access to viral load, and a general lack of guidance in national treatment guidelines, with consequent delays in switching from failing regimens [22] and accumulation of resistance mutations.

As a guiding principle, PADO 2 participants reiterated the importance of maintaining a public health approach focused on harmonization and simplification of regimens to minimize fragmentation and in turn support sustainability of supply.

To date, while there is robust, randomized, trial data to support the use of LPV/r over NVP in children less than three years, for neonates under two weeks of age NVP remains the preferred choice as there is no approved dose or formulation that can be used in this age group. Unfortunately, there is only sparse PK and safety data for other drugs in newborns (especially low birth weight or premature infants).

For adolescents, consideration was given to providing an integrase-inhibitor-based, first-line regimen to improve adherence, but it was recognized that drug optimization for adolescents needed to focus on maintaining simplification and harmonization with adult regimens. The treatment of adolescents infected horizontally may pose specific challenges, such as further reduction of adherence as a result of lack of support and poor access to health services as a result of stigma and discrimination. While perinatally infected adolescents face issues related to disclosure, treatment fatigue or stigma within their schools, homes and communities, those who acquire HIV through horizontal transmission may have specific challenges for adherence especially if they are infected as a result of sexual abuse or injection drug use.

Integrase inhibitors may also have a role in second-line regimens, particularly for children failing treatment after starting a PI-based regimen. Defining the optimal sequencing of this class of drugs and improving availability are key priorities.

The development of once-a-day FDC regimen continues to be a priority. For first-line regimen, the triple FDC ABC/3TC/EFV remains a critical product for children aged 3–10 years. Raltegravir (RAL) for infants could be of importance due to the time lag expected for full approval of dolutegravir (DTG), a potentially more potent integrase inhibitor with the advantage of a once-daily administration. For second- and third-line regimens, DRV/r or ATV/r co-formulations are key products still missing. DTG-based FDCs are of particular interest for adolescents, for whom DTG is already licensed [23]. Infants and small children (<3 years and 10 kg) currently have very limited ART options particularly in TB co-infection where drug–drug interactions may be difficult to manage. Finally, the improved toxicity profile of tenofovir alafenamide fumarate (TAF) [24] compared to TDF reported in trials among adults could make it an attractive option, given the concerns for bone toxicity and growth associated with the use of TDF in children. An accelerated paediatric development plan is needed for this drug.

### Pushing forward treatment optimization

Since the early 2000s when the only option for treating children was to split adult tablets, tremendous progress has been made to ensure access to better, simpler treatments for children. Yet more needs to be done.

Recent data on the immunological benefits of early therapy, including the elusive prospect of a cure, suggest that children should start treatment as soon as their infection status is confirmed. Innovative strategies have been developed to study the PK of new ARVs in newborn infants taking advantage of the fact that new regimens are being evaluated in pregnant women. Studies of drug clearance among infants who experience transplacental exposure to medications have provided essential data on their PK early in life. Originator companies have been willing to share their intellectual property rights for drugs for children, but there remains a need for companies to pursue the paediatric development of their newer drugs all the way down to infancy as individual drugs (rather than the sole development of FDCs of drugs owned by the same company). Advocacy is needed to address the lack of paediatric investigation plans for single entities that would enable development of generic FDCs and to ensure that those plans are completed as quickly as possible. While PK modelling can fill gaps and allow development of some of the needed FDCs identified at PADO 2, the availability of clinical data to verify weight-band dosing and promote harmonization of weight-bands across multiple products remains essential for the development of new products.

Clinical trials must be performed to determine the optimal sequence of new drugs from birth to adolescence. A good example is the integrase inhibitors. These potent new drugs appear to be very safe, yet they have to prove superiority or at least equivalence to the drugs currently recommended. Demonstrating the relative efficacy, safety and robustness of integrase-inhibitor-based regimens in comparison to protease inhibitors or NNRTI-based regimens will require two to three years. Adaptive trial designs could support the fast-tracking of head-to-head comparisons of new drugs as they become available [25].

Regulators and ethics committees are key actors in efforts to improve access to new, better therapies for children. They need to see their role as not only protecting patients, but also facilitating and expediting paediatric clinical research, without which children may still lag far behind adults. Efforts towards joint approval of drugs at the regional level represent an important step forward.

While adolescents are the first to benefit from the improvements of therapy for adults, there is a need to consider new formulations adapted to their needs. Very short interruptions, such as weekends off drugs [26], provided the drugs have a long half-life, could improve adherence in this group, and long-acting combinations that require bi-weekly or monthly dosing would be ideal during adolescent years.

## Conclusions

The priority for the future is to improve access to ART at the two ends of the age spectrum – infants and adolescents – where the treatment gap is greatest, and optimize drug sequencing with better use of available medicines for second and third line. More efforts are needed to develop FDCs that allow simplified weight-band dosing and minimize the number of products needed, which in turn will improve procurement and availability.

Future efforts will require continuous collaboration and coordination, and the promotion of innovative approaches to accelerate access to new drugs. While significant progress has been made, additional efforts are needed to ensure that treatment targets are reached by 2020. Originator pharmaceutical companies, generic producers and regulators all need to work together to meet with the needs of children in resource-limited settings.

## References

[1] UNAIDS (2014). 90-90-90 An ambitious treatment target to help end the AIDS epidemic.

[2] Lallemant M, Chang S, Cohen R, Pecoul B (2011). Pediatric HIV – a neglected disease?. N Engl J Med.

[3] iBase 2014 Pipeline report.

[4] UNAIDS (2015). 15 by 15 report.

[5] Penazzato M, Bendaud V, Nelson L, Stover J, Mahy M (2014). Estimating future trends in paediatric HIV. AIDS.

[6] European Medicines Agency Article 58 applications: regulatory and procedural guidance. European Medicines Agency.

[7] Ponnet M, Frederix K, Petdachai W, Wilson D, Eksaengsri A, Zachariah R (2005). A drug dosage table is a useful tool to facilitate prescriptions of antiretroviral drugs for children in Thailand. Int J STD AIDS.

[8] World Health Organization WHO generic tool for assessing paediatric ARV dosing.

[9] WHO (2010). Antiretroviral therapy for HIV infection in infants and children: recommendations for a public health approach. 2010 revision.

[10] World Health Organization (2013). Consolidated guidelines on the use of antiretroviral drugs for treating and preventing HIV infections: recommendations for a public health approach.

[11] World Health Organization The annual WHO surveys on the use of ART.

[12] Global pricing reduction mechanism WHO.

[13] World Health Organization (2014). Paediatric ARV drug optimization 2 meeting report.

[14] World Health Organization March 2014 supplement to the 2013 consolidated guidelines on the use of antiretroviral drugs for treating and preventing HIV infection: recommendations for a public health approach.

[15] Holford N, Heo Y, Anderson B (2013). A pharmacokinetic standard for babies and adults. J Pharm Sci.

[16] Fillekes Q, Natukunda E, Balungi J, Kendall L, Bwakura-Dangarembizi M, Keishanyu R (2011). Pediatric underdosing of efavirenz: a pharmacokinetic study in Uganda. J Acquir Immune Defic Syndr.

[17] UNITAID/Drugs for Neglected Diseases initiative (DNDi)/Medicines Patent Pool (MPP)/Clinton Health Access Initiative (CHAI) Paediatric HIV treatment initiative (PHTI).

[18] Meeting Report (2013). Update to the optimal list of paediatric ARV formulations.

[19] (2015). Interagency Task Team on Prevention and Treatment of HIV Infection in Pregnant Women, Mothers and Their Children (IATT).

[20] Rainwater-Lovett K, Luzuriaga K, Persaud D (2015). Very early combination antiretroviral therapy in infants: prospects for cure. Curr Opin HIV AIDS.

[21] Reisner SL, Mimiaga MJ, Skeer M, Perkovich B, Johnson CV, Safren SA (2009). A review of HIV antiretroviral adherence and intervention studies among HIV-infected youth. Top HIV Med.

[22] Davies MA, Moultrie H, Eley B, Rabie H, Van Cutsem G, Giddy J (2011). Virologic failure and second-line antiretroviral therapy in children in South Africa – the IeDEA Southern Africa collaboration. J Acquir Immune Defic Syndr.

[23] Viani RM, Alvero C, Fenton T, Acosta E, Hazra R, O’Gara E (2014). Safety and efficacy of dolutegravir in HIV treatment-experienced adolescents: 48-week results.

[24] Sax PE, Saag MS, Yin MT, Post FA, Oka S, Koenig E (2015). Renal and bone safety of tenofovir alafenamide vs tenofovir disoproxil fumarate.

[25] Wason JM, Trippa L (2014). A comparison of Bayesian adaptive randomization and multi-stage designs for multi-arm clinical trials. Stat Med.

[26] K Butler for BREATHER Trial Team (2015). ART with weekends off is non-inferior to continuous ART in young people on EFV + 2NRTI.

# Sub article: Tuberculosis: opportunities and challenges for the 90–90–90 targets in HIV-infected children

**DOI:** 10.7448/IAS.18.7.20236

**Published:** 2015-12-02

**Authors:** Helena Rabie, Lisa Frigati, Anneke C Hesseling, Anthony J Garcia-Prats

**Affiliations:** 1 Division of Infectious Diseases, Department of Paediatrics and Child Health, Faculty of Medicine and Health Sciences, Stellenbosch University and Tygerberg Hospital, Tygerberg, South Africa; 2 Desmond Tutu TB Centre, Department of Paediatrics and Child Health, Faculty of Medicine and Health Sciences, Stellenbosch University, Tygerberg, South Africa

**Keywords:** tuberculosis, HIV, AIDS, children, infant, treatment, epidemiology, 90-90-90 target

## Abstract

**Introduction:**

In 2014 the Joint United Nations Programme on HIV/AIDS defined the ambitious 90–90–90 targets for 2020, in which 90% of people living with HIV must be diagnosed, 90% of those diagnosed should be on sustained therapy and 90% of those on therapy should have an undetectable viral load. Children are considered to be a key focus population for these targets. This review will highlight key components of the epidemiology, prevention and treatment of tuberculosis (TB) in HIV-infected children in the era of increasing access to antiretroviral therapy (ART) and their relation to the 90–90–90 targets.

**Discussion:**

The majority of HIV-infected children live in countries with a high burden of TB. In settings with a high burden of both diseases such as in sub-Saharan Africa, up to 57% of children diagnosed with and treated for TB are HIV-infected. TB results in substantial morbidity and mortality in HIV-infected children, so preventing TB and optimizing its treatment in HIV-infected children will be important to ensuring good long-term outcomes. Prevention of TB can be achieved by increasing access to ART to both children and adults, and appropriate provision of isoniazid preventative therapy. Co-treatment of HIV and TB is complicated by drug-drug interactions particularly due to the use of rifampicin; these may compromise virologic outcomes if appropriate corrective actions are not taken. There remain substantial operational challenges, and improved integration of paediatric TB and HIV services, including with antenatal and routine under-five care, is an important priority.

**Conclusions:**

TB may be an important barrier to achievement of the 90–90–90 targets, but specific attention to TB care in HIV-infected children may provide important opportunities to enhance the care of both TB and HIV in children.

## Introduction

An estimated 240,000 children were newly infected with HIV in 2013 despite scale-up of prevention of mother-to-child transmission (PMTCT), which resulted in a 40% reduction in perinatal transmission of HIV in 2011 compared to 2009 [1]. The majority of HIV-infected children (630,300) live in Africa, followed by Asia (54,100) and Latin America (26,400) [1]. Although 13.6 million HIV-infected people accessed combination antiretroviral therapy (ART) in 2014, this represents only 38% of adults and 24% of children with HIV. The regions with the lowest access to therapy are also in Africa (20 to 24%) and Asia (22 to 32%) [1]. In order to address these ongoing critical gaps in HIV care, the Joint United Nations Programme on HIV/AIDS defined the ambitious 90–90–90 targets to be achieved by 2020: 90% of persons living with HIV must be diagnosed, 90% of those diagnosed should be on sustained therapy and 90% of those on therapy should have an undetectable viral load. These targets were designed to stimulate rapid scale-up of sustainable and high quality HIV care, in order to prevent new HIV infections and to reduce HIV-associated morbidity and mortality. Adolescents and children were identified as key focus populations [2].

Tuberculosis (TB) remains a major cause of disease globally, with 9 million incident cases; 13% of these cases were HIV-infected. There were 1.5 million TB deaths in 2013, of which 360,000 were in HIV-infected persons. Although Asian countries (particularly China and India) account for the highest numbers of TB cases, all the countries with a TB incidence of more than 500 cases per 100,000 population are in Africa [3]. Models estimate that in 2010 there were 650,000 cases of TB disease in children and many more with latent infection [4]. In settings with a high burden of both diseases up to 56% of children treated for TB had HIV [5].

Given the close overlap of the TB and HIV epidemics, successful achievement of the 90–90–90 targets must specifically consider TB and its impact on HIV diagnosis, retention in care and attaining virologic suppression.

In this paper we discuss key aspects of the care of TB in HIV-infected children, highlighting threats and opportunities on the road to 90–90–90. We will discuss the epidemiology of TB-HIV co-infection, calling attention to the ongoing close interactions of these two diseases, as well as recent advances in the prevention of TB in HIV-infected children. We will describe developments in the treatment of TB and HIV and the implications of these for the reduction of morbidity and mortality in TB-HIV co-infected children. We will also focus on the emerging threat of drug-resistant TB.

## The epidemiology of TB-HIV co-infection

As described above, the TB and HIV epidemics have substantial overlap in their epidemiology. It is clear that Africa (particularly sub-Saharan Africa), South East Asia (Thailand and India) and Latin America carry the biggest burden of TB and HIV [1,6]. This has impacted children directly and through its impact on women of childbearing age. The HIV epidemic disproportionately increased the burden of TB in women of childbearing age [7]. One-third of TB deaths in women are in HIV-infected women [6]. Data from India confirms that where TB occurs in HIV-infected pregnant women, the infant is not only at risk for TB, but has a threefold higher risk of HIV acquisition and a fourfold increase in mortality [8–10].

### The risk of TB in HIV-infected children

HIV-infected and HIV-exposed infants are highly TB exposed in their households and local communities. In a South African trial of isoniazid preventive therapy (IPT), 10% of HIV-exposed infants had contact with a potential case of TB by 14 weeks of age [11].

In addition to frequent TB exposure, other conditions, including advanced immune suppression, poor nutritional status (stunting, wasting and reduced mid-upper arm circumference) and anaemia are associated with increased risk of TB in HIV-infected children [12]. The proportion of children with TB that are HIV co-infected varies from 5.8 to 56% depending on the setting [5].

Among South African infants with limited access to ART, up to 1595 cases per 100,000 population have been reported; this is more than 24 times the rate reported at the same time for HIV-uninfected South African infants [13]. The risk increases after six months of age in the absence of ART, with incidence rates of 6.2 to 18.1 cases per 100 person-years (age <6 months) and 9.4 to 46.3 cases per 100 person-years (age ≥6 months) reported in East Africa [14]. In other HIV-infected African children with poor access to ART, high risk of TB is also reported (more than 17 TB cases per 100 person-years) [15–18]. A review study of African post-mortem studies reports pulmonary TB in 8.3% of HIV-infected children [19].

In low-burden settings, the risk of TB in HIV-infected children is much less; however TB still occurs. Among children attending HIV services in the United Kingdom (between 1991 and 2006) and New York City (between 1989 and 1995) 3 to 5.5% had a diagnosis of TB at some point [20,21].

## TB prevention in HIV-infected children

TB co-infection negatively affects outcomes for HIV-infected patients, and there are challenges with TB diagnosis and co-treatment in HIV-infected children. These factors may all impact negatively on achieving 90% retention on ART and 90% virologic suppression; hence strategies to prevent TB in HIV-infected children are a priority for achieving the 90–90–90 targets.

### Antiretroviral therapy for HIV-infected children and adults

Increasing access to ART in paediatric cohorts decreases confirmed and probable TB. In the Children with HIV Early Antiretroviral Therapy (CHER) study, South African infants with deferred ART had 20 cases of TB per 100 person-years, compared to 8.3 cases per 100 person years in children receiving early ART [17]. Up to 33% of infants starting ART at a median age of eight months are already on TB therapy, indicating early infancy as a particular risk period and highlighting the importance of early initiation of ART [22]. A similar reduction in risk of TB in older children receiving ART has also been demonstrated in diverse settings in Africa, Asia and Latin America [15,16,18,22–28].

The first three months after initiation of ART represent a very high-risk period for hospitalization and death [29]. In addition, there is a substantial initial increase in the risk of TB, likely representing undetected TB, TB exposure at ART initiation or unmasking TB immune reconstitution inflammatory syndrome (IRIS) [27,30–33].

With increasing access to ART in adults, the risk of TB declines in adults as well as in HIV-infected and -uninfected children. In Johannesburg, South Africa, ART access in adults increased from 21.5% in 2005 to 68.2% in 2009 of those requiring ART. During the same period, TB in HIV-infected children declined from 1566.3 to 460.7 per 100,000 and in HIV-uninfected children from 18.7 to 11.0 per 100,000 [34]. If ART expands to cover 80% of adults living with HIV, TB incidence among all adults is projected to decline by 28 to 37% [35]. A reduction of adult TB cases, particularly among HIV-infected adults, would likely have an important impact on the risk of TB exposure, infection and disease among HIV-infected children.

Despite these clear benefits of ART, the risk of TB remains high in HIV-infected infants and children even when on ART. In South African infants with a 98% uptake of ART, those that were HIV-infected had a burden of 121 cases per 1000 years compared to 41 cases per 1000 child-years in HIV-exposed uninfected infants [36]. This highlights the importance of a multi-pronged strategy to address TB in HIV-infected children.

### BCG vaccination

Although Bacille Calmette Guérin (BCG), a live attenuated *Mycobacterium bovis* strain, is protective against disseminated TB, including meningitis in young children, it is less effective at preventing pulmonary TB [37]. BCG itself poses a risk to HIV-infected children, particularly those with severe immunosuppression and delayed initiation of ART. Disseminated BCG, a serious and potentially life-threatening complication of BCG vaccination, occurs in an estimated 992 (95% CI: 567 to 1495) per 100,000 HIV-infected infants [38]. In addition, infants initiating ART may develop IRIS related to BCG [39]. Given these concerns, the WHO recommended that BCG not be administered to persons who are confirmed HIV-infection [40]. As BCG is given at birth to healthy infants, this recommendation is problematic in settings where birth HIV DNA PCR is not routine, particularly as these are often the same settings with a high TB burden. Early infant diagnosis of HIV and early ART mitigates the risk of disseminated BCG and IRIS [41]. In a prospective study of 451 HIV-infected infants receiving early ART, none developed disseminated BCG disease; although BCG IRIS remained a problem, the risk was reduced threefold with early access to ART [39]. Rather than altering BCG vaccine delivery or managing IRIS, most countries are focusing on prevention of HIV transmission to children and access to ART [42].

### Isoniazid preventative therapy

Young age is an important risk factor for the development of TB disease after infection [43]. Treating latent *Mycobacterium tuberculosis* infection with isoniazid (INH) prevents disease [44,45]. Therefore the WHO recommends the provision of IPT for a minimum of six months to all children <5 years of age, and HIV-infected children of any age, with a documented infectious TB source case; this is an integral component of TB prevention in HIV-infected children [6].

Studies of pre-exposure and longer-term IPT in HIV-infected children have yielded conflicting evidence. In the only true pre-exposure prevention trial (IMPAACT P1041), 548 HIV-infected and 804 HIV-exposed uninfected infants between three and four months of age without TB exposure were randomized to INH versus placebo for 96 weeks with follow-up for another 96 weeks. The pre-exposure prevention did not reduce the TB incidence in either HIV-exposed-uninfected or HIV-infected infants [36]. An earlier placebo controlled study of IPT for all HIV-infected children enrolled older children with delayed access to ART, including those with prior TB or TB exposure. This study showed a significant reduction in TB and all-cause mortality (9.9 and 16% in the placebo arm vs. 3.8 and 8% in the IPT arm). Subsequent analysis of this cohort illustrated that combining IPT with ART was more effective than either strategy alone to prevent TB [16,46]. In 2014, the WHO recommended routine pre-exposure IPT for six months in all HIV-infected children older than 12 months without evidence of TB disease [6]. Children with suggestive symptoms (poor weight gain, fever and current cough) or with a history of TB exposure should be investigated for TB prior to IPT initiation. Diagnostic challenges in HIV-infected children remain a reality, especially if there is no TB contact but suggestive symptoms. The WHO guidelines also recommend that children treated for TB with a good response should receive six months of INH post–TB treatment completion; however, there is little evidence for this recommendation.

There are substantial challenges in IPT implementation, including health system weakness and lack of availability of child-friendly INH formulations. Missed opportunities for chemoprophylaxis are well documented. A community-based study from South Africa found that only 21% of eligible children received IPT and that the lack of specific tools to ensure implementation possibly contributed to low uptake [47]. Poor adherence to IPT remains a challenge [48]. Health care worker training and introduction of specific documentation such as cards and registers, as well as contact tracing, increased uptake of IPT from 16 to 61% [49]. Integration of TB and HIV services may also increase IPT completion [50].

In HIV-uninfected children, adherence is improved by using rifamycins, which require shorter courses [51]. This is not an option for HIV-infected children on ART or HIV-exposed infants on extended nevirapine (NVP) prophylaxis for HIV prevention, as rifamycins have important drug-drug interactions with a number of antiretrovirals. Twelve doses of rifapentine-INH, recently shown to be effective in HIV-uninfected children, was not studied in HIV-infected children [52].

There is no published data in HIV-infected children on the benefit of tuberculin skin testing or other tests of infection, such as the interferon gamma release assays, to help target pre-exposure IPT. Data in HIV-infected adults suggests that TST-positive adults benefit the most from IPT [53]. No TB prevention studies have specifically targeted adolescents. However, the risk of TB increases during adolescence [43], and HIV-infected adolescents with TB exposure or infection would potentially benefit considerably from IPT. Drug resistance in TB source cases may be a major factor in children failing IPT. In the IMPAACT P1041 trial, children developing culture-confirmed TB on INH did not have INH mono-resistance, but rather multidrug-resistant (MDR) TB [54]. Obtaining a thorough history of the potential contacts prior to IPT initiation is important; if a child on IPT develops TB, the contacts should be reviewed again for resistance and clinically relevant specimens should be taken for culture and susceptibility testing prior to starting TB treatment.

### Other TB prevention opportunities

There are a number of other additional opportunities for preventing TB in HIV-infected children. Temporal associations between TB, influenza and pneumococcal disease in children suggest a complex interaction between these infections. An increase in TB diagnoses following approximately three months after an influenza epidemic in a high burden setting suggests a potential role for the influenza vaccine in reducing the TB burden [55]. A study of TB after introduction of conjugated pneumococcal vaccine showed that culture-confirmed TB was 47% less in vaccinated compared to unvaccinated HIV-infected children [56]. Cotrimoxazole appears to have anti-mycobacterial activity [57] and conflicting evidence suggests cotrimoxazole prophylaxis may reduce incident TB in HIV-infected adults [58,59]. In a trial of 758 HIV-infected children on ART randomized to prolonged cotrimoxazole despite immune reconstitution, there were fewer incident cases of TB, although numbers were small [60]. These strategies, in addition to reducing their targeted disease, may have the benefit of reducing TB, adding to the urgency of their implementation in HIV-infected children. In the absence of a highly effective TB vaccine, a multi-pronged approach to TB prevention is likely to be the most successful and is important in enabling achievement of the 90–90–90 targets.

## Outcomes among TB-HIV co-infected children

Prior to wide access to ART, high mortality of co-infected children was reported. A study from South Africa documented a 10 times higher mortality in TB-HIV co-infected children than in HIV-uninfected children with TB [12]. Children with TB-HIV co-infection who died often had additional pulmonary infections on post-mortem [61]. With access to ART, better outcomes are reported, particularly for children who develop TB while on ART for more than six months; however, mortality does occur especially in the first two months of ART. A retrospective observational study from South Africa reported 10% mortality in TB-HIV co-infected children less than two years of age [18,22]. Deaths may be due to TB, other HIV complications or IRIS. The effect of TB co-infection on mortality thus affects the achievement of 90% retention on ART.

### Virologic outcomes in children co-treated with rifampicin

Concomitant TB therapy may be a significant risk factor for virologic failure in children who are co-treated, impacting the target of 90% of children on ART being virologically suppressed. Viral load is not available in all settings where there is a high TB burden. In settings where the protease inhibitor (PI) lopinavir/ritonavir (4:1) LPV/r is the preferred initial therapy in children<3 years and super-boosting is used, high levels of viral suppression are reported for children, regardless of co-treatment. However the rates of suppression at 6 and 12 months are lower than those of children not requiring co-treatment [62–64]. Studies of children failing LPV-based regimens have shown accumulation of PI mutations in 10% of children tested a median of 21 months after initiation of therapy [65]. Although concurrent TB therapy may be a risk factor for virologic failure, it was not necessarily a risk factor for LPV resistance [67]. A likely explanation is the robust resistance profile of LPV; however great care should be taken to ensure dosing accuracy and appropriate addition of ritonavir (RTV). RTV at full dose should not be used as therapy, as it is associated with the highest risk of failure and PI resistance [62,66].

No difference in virologic suppression has been seen with efavirenz (EFV)-based therapies at month 12 [64]. There is limited data on the routine virologic outcomes of children treated with NVP. However, poorer virologic outcomes are reported in several cohort studies when compared to EFV, regardless of TB therapy [67,68].

## Treatment

Optimizing the co-treatment of TB and HIV is important to ensuring the targeted 90% treatment success in HIV-infected children.

In low resource settings, TB treatment initiation is as frequent a cause of ART regimen changes as adverse effects [69]. When choosing an ART regimen, apart from drug interactions, clinicians should also consider prior ART, available drugs and formulations, age and weight. For children on ART, clinicians must also consider duration on therapy, adherence to therapy and the probability of therapeutic failure.

### First-line TB medications and drug-susceptible TB

In 2010, the WHO recommended higher doses of the first-line anti-TB drugs in all children [70]. This recommendation was based on an extensive review of evidence demonstrating low exposure with the previously recommended doses. These changes introduced practical challenges, as the ratio of RMP, INH and pyrazinamide (PZA) in the existing paediatric fixed-dose combination (FDC) formulations does not allow for easy dosing within the new recommendations [71].

Among 20 children younger than two years receiving the newly recommended doses of INH, RMP and PZA, nearly all children had maximum serum concentrations of these medications above target levels. HIV-infected children in this study (*n=*5) had a significantly lower C_max_ and T_max_ on PZA 35 mg/kg, but no difference in total exposure; the small sample size may have limited the ability to detect other differences [72]. In 31 South African children younger than 10 years, the majority receiving the new WHO-recommended doses of the first-line TB medications, two-hour target concentrations were attained for RMP in only 2/31 (6%), for INH in 20/31 (65%), for PZA in 17/31 (55%) and for ethambutol (EMB) in 2/13 (15%); HIV infection (*n=*7 children) was associated with a low two-hour INH concentration [73]. It is not clear whether the reduced TB drug concentrations reported in these studies are related to interactions with ART or the direct effects of HIV infection; neither study clearly describes the ART regimens in the included HIV-infected children. Studies of the first-line TB medications in children are ongoing (NCT01637558 and NCT01687504).

Despite limited evidence, the WHO recommends the addition of EMB to the intensive phase of TB treatment in children with HIV, extensive disease or in settings with a high prevalence of INH resistance [6]. The rationale for this is that EMB may protect against the acquisition of RMP resistance in children with existing INH resistance; acquired RMP resistance in children has been described, although the actual risk is not known [74]. Additional data on the benefit, risk and programmatic implications of this recommendation for HIV-infected children is needed. An extensive review of the literature identified very few reports of EMB-associated ophthalmologic toxicity in children [75]. Recently in a study of a small number of children treated with EMB, newer technology identified reversible ophthalmological complications attributed to EMB. EMB should continue to be used when indicated, but this risk deserves further evaluation [76].

### Second-line TB medications

Despite common use there is little data on the pharmacokinetics of second-line TB medications in children [77]. The currently recommended doses of ofloxacin, levofloxacin and moxifloxacin result in drug exposures considerably lower than in adults, and moxifloxacin exposure is significantly lower in HIV-infected compared to HIV-uninfected children [78,79]. It is not clear if this is due to poor absorption or a potential drug interaction with RTV [79,80]. In HIV-infected adults, co-treatment with EFV results in a more than 30% reduction in para-aminosalicylic acid exposure [81]. There are no recommendations to alter doses of any of the second-line TB medications or ART in co-treated children, but additional data is urgently needed.

HIV infection may be a risk factor for adverse events among children treated for MDR-TB. A higher risk of ethionamide-induced hypothyroidism has been shown compared to HIV-uninfected children [82]. Linezolid, used in children with extensively drug-resistant TB, may have additive adverse effects with nucleoside reverse transcriptase inhibitors (NRTIs) due to mitochondrial protein synthesis inhibition and should be used with caution in HIV-infected children [83].

### Novel TB medications

Two novel TB medications, the ATP-synthase inhibitor bedaquiline and the nitroimidazole delamanid are increasingly used in adults [84,85]. Phase 1 and 2 studies of delamanid in children (NCT01856634, NCT01859923) have begun enrolment, and studies of bedaquiline in children are planned. Current studies exclude HIV-infected children but future studies will include them. Metabolism of delamanid is partially mediated by the cytochrome p450 (CYP) 3A4 enzyme, but healthy volunteer studies indicated no clinically significant drug-drug interactions with ART [85]. Bedaquiline is metabolized by CYP3A4 isoenzyme; co-administration with the CYP3A4 inducer EFV reduces exposures of bedaquiline and its main metabolite, M2, by roughly 50%. Co-administration with lopinavir, an inhibitor of CYP3A4, reduces clearance by 35% for bedaquiline and by 58% for M2, resulting in two- to threefold higher exposures of bedaquiline and M2. There is no clinically significant interaction between bedaquiline and NVP [86–88]. Data on the pharmacokinetics and safety of bedaquiline and delamanid in HIV-infected children will be important to ensure these medications are accessible and able to be safely used in these children.

### Deciding on the appropriate ART in children with TB

Drug-drug interactions between TB medication, especially rifamycins, and ART are a major concern, as they may influence the virologic outcomes of co-treated patients, leading to HIV treatment failure and increasing the risk of ART resistance, potentially limiting future therapy. The drug interactions between NRTIs and RMP are not considered clinically significant; however there are significant interactions with non-nucleoside reverse transcriptase inhibitors (NNRTIs), PI and integrase inhibitors. For some of these interactions, there is no paediatric data.

### Protease inhibitors

LPV/r is superior to NVP in young children regardless of previous NVP or EFV exposure through PMTCT [89,90]. LPV/r is now recommended as first line in most children <3 years of age [91]. Given its more prominent role, drug-drug interactions with TB medications are important. LPV/r concentrations are reduced by RMP CYP3A4 induction and through changes in the p-glycoprotein expression [92]. Adding RTV to alter the ratio from 1:4 to 1:1 (so called super-boosting) was shown to overcome this RMP effect in a study of 15 children (median age 16 months, median weight 8.6 kg). The median C_max_ and AUC_0–12_ were lower than in controls, but the C_min_ (the target for efficacy) was greater than the minimum recommended. Children tolerated the strategy and two cases had mild alanine transaminase elevation that did not require therapy interruption [93]. Preliminary data from an ongoing study of a large cohort of young TB-HIV co-treated South Africans receiving super-boosting (NCT02348177) confirms this finding [94].

In adults, giving double the dose of LPV/r was found to have acceptable pharmacokinetic and less toxicity [95]. However, of 20 children (median weight 9.1 kg, median age 1.2 years) receiving double-dose LPV/r (460/115 mg/m^2^ twice), 80% did not achieve a target morning trough of 1 mg/L [96]. Explanations include characteristics of the formulation as well as drug absorption and metabolism. A modelling study suggests that overcoming the interaction with RMP using the LPV/r 4:1 solution with twice daily administration will require such high doses that there may be adverse events, whereas an eight-hourly dosing regimen may overcome the drug interaction; a study of this is ongoing [97]. The individual RTV formulation requires refrigeration for storage and has a short shelf life, complicating its use in resource-limited settings, and when only used for super-boosting may be a challenge for supply chains to maintain continuous widespread availability. Additionally, it is poorly palatable and may be problematic to administer to children. In settings increasingly utilizing task-shifting and relying on nurses for ART provision, these complicated drug-drug interactions between LPV/r and TB medication may be a barrier to a super-boosting strategy. The optimal approach to ART in TB-HIV co-infected children may need to be considered by each high-burden country depending on these contextual issues, and improved options are needed. In order to facilitate access to LPV/r, mini-tablets were developed and are now licensed by FDA.

Rifabutin, which does not affect PI concentrations, is also problematic. Rifabutin is metabolized by CYP3A4 and dose adjustments of rifabutin are needed if co-treating with PIs; however pharmacokinetic data in children is limited. In six children treated with rifabutin 5 mg/kg three times per week and with LPV/r, severe transient neutropenia and insufficient rifabutin exposure was observed [98]. Using rifabutin in a public health programme is currently not possible due to lack of data in co-treated children, complex two-way interactions, cost and lack of an FDC.

There is a lack of paediatric data for boosted atazanavir (ATV) and darunavir (DRV), which also both interact with RMP. In adult volunteers, double doses of ATV with RMP did not correct the ATV exposure but did not cause substantial toxicity [99]. For DRV, modelling of adult data suggests that dose increases of DRV and RTV (800/100 mg and 1200/150 mg twice a day) both may overcome the RMP induction; the usual adult dose is 800/100 mg daily for naïve patients older than 12 years of age [100]. This dose increase may, however, cause adverse effects and there are no published data studying this approach. The interaction and optimal PI treatment strategy in children with TB is a critical area for future research given the importance of these medications in ART regimens.

### Non-nucleoside reverse transcriptase inhibitors

EFV is used in older children and is superior to NVP [67]. Recent data suggests that no adjustment of EFV dose is required when RMP is used [101,102]. Previously a lack of data in dosing prevented EFV use in children younger than three years. Though it is now licensed for this age, experts recommend CYP2B6 genotype prior to EFV initiation in this age group [103]. Using EFV in this age group with prior NNRTI exposure has not been studied and data on co-treatment with RMP-containing TB regimens are not available.

NVP is a commonly used NNRTI in low resource settings, where it is incorporated in easy-to-use and well-tolerated FDCs. There are reports of adequate exposure in RMP co-treatment but larger co-treatment studies found significant under-dosing even if NVP was given at more than the standard recommended dose [104–106]. The period of initiating NVP-containing ART using daily NVP for 14 days may be particularly risky in children who are also on RMP, as NVP concentrations may be low; avoiding the induction dosing in all children younger than two years of age has been suggested [107]. Where NVP use cannot be avoided in TB co-treated children, a dose of 200 mg/m^2^/dose twice daily should be used [91]. Switching children with TB from NVP to EFV should be considered wherever possible. Lastly, children taking NVP as PMTCT, where mothers are on RMP or where the infants require RMP-TB therapy, may have low NVP concentrations [108]. These low concentrations may reduce the efficacy of NVP in preventing HIV.

Switching suppressed children from LPV/r to EFV is a strategy studied in children without TB [109]. Although prospective studies have not been performed, a switch to EFV can be considered if children develop TB. If at all possible, viral load monitoring should be done to detect early failure, in which case further action must be taken.

Etravirine is not typically available in low resource settings and is not approved for use in children less than six years of age. There are no adult studies assessing the interaction of RMP and etravirine, with the exception of case reports, which confirm a significant reduction in plasma levels, which did not impact virologic suppression [110].

Based on results of the Antiretroviral Research for Watoto (ARROW) study, the WHO recommends triple NRTIs as a preferred strategy in children age <3 years requiring RMP co-treatment. In ARROW, three NRTIs were studied as an intensive induction and a maintenance strategy when combined with NNRTIs. After 48 weeks of initial therapy that contained a NNRTI children were randomized to a three-drug NRTI maintenance regimen or to remain on conventional therapy. Fifty-nine of the 1143 children required a drug alteration for TB that included stopping or replacing NVP. Children on EFV did not require drug switch. Triple NRTI was effective at 36 weeks, but not at 144 weeks. The advantage of this strategy is that, although viral suppression is inferior, the risk of progressive NNRTI and PI resistance was avoided and complicated drug-drug interactions and logistic complications were easier to manage [69]. Where PIs are available, limited NRTI resistance may not compromise future suppression. However, this strategy could be problematic in very ill children where viral control and immune recovery is essential to improve the health of the child.

### Integrase inhibitors and other issues

Integrase inhibitors are an increasingly important class of ART, particularly as a key component of third-line regimens. Raltegravir and dolutegravir are not metabolized by the CYP P450 enzymes but are metabolized through the liver by uridine 5-diphospho (UDP)-glucuro-nosyltransferase 1A1, an enzyme that is also induced by RMP. Healthy volunteer studies showed a significant reduction in raltegravir exposure with RMP co-treatment [111]. In a prospective study comparing standard and double-dose raltegravir with EFV in ART-naïve adults requiring RMP, no significant difference was found in virologic suppression at 24 weeks between the standard and double-dose raltegravir groups, with both groups similar to the EFV group [111]. There is no published data in children; however an ongoing study is assessing the effective dosing, safety and tolerance of children co-treated with raltegravir and RMP (NCT01751568).

Adult healthy volunteer studies assessing the effect of RMP on dolutegravir suggest that doubling the dolutegravir dose is needed if there is co-treatment with RMP [112].

Children needing third-line therapy and co-treatment with RMP-containing TB therapy may benefit from a holding triple NRTI strategy. This may be a good option if the children have a preserved CD4 count and are clinically stable. Where children require a suppressive regimen urgently, consideration should be given to changing to a non-rifamycin-containing TB regimen. Rifabutin also has less substantial interactions, but dose adjustment of rifabutin and ART may still be needed. Rifapentine is not recommended, as there may be a risk of RMP-monoresistant TB developing [113]. Fluoroquinolone-based TB regimens could be considered, but have not been prospectively studied in this context

### Approach to timing of ART initiation

The timing of ART initiation in adults with TB has been extensively studied. Delaying therapy in adults with a CD4 count of <200 cells/mL has been associated with poor virologic and clinical outcomes [112]. The timing of ART initiation in children with TB has not been studied prospectively. In observational paediatric cohorts, delaying therapy for up to two months was not associated with an increased risk of mortality or poorer virologic response [114]. Whether longer delay in older children and adolescents with good CD4 counts and minimal TB disease is acceptable is not known.

TB meningitis (TBM) is an exception regardless of CD4 count, since clinical deterioration due to paradoxical IRIS can be devastating. In the adult literature, IRIS is associated with more disseminated forms of the disease and positive culture in the cerebrospinal fluid. It is common practice to delay ART four to six weeks in TBM [115]. Although there are fewer data in children, case series confirm the high morbidity associated with paradoxical IRIS [116]. There are no prospective data in children and it is important to keep in mind that the outcomes in patients with TBM are also determined by the severity of meningitis. Only 20% of HIV-uninfected children are neurologically normal after full TBM treatment [117].

If children are already on ART, appropriate anti-TB therapy should be started as soon as the appropriate diagnostic testing has been performed. ART should be adapted and the possibility of virologic and or immunological failure must be considered and appropriately investigated.

## Drug-resistant TB

MDR-TB (resistant to INH and RMP) is a growing health threat with an estimated 480,000 cases occurring in 2013; 9% of these cases also had additional resistance to a fluoroquinolone and/or a second-line injectable medication [3]. Despite limited data on the burden of MDR-TB in children, a 2010 model estimates a burden of 32,000 cases. Children typically have transmitted or primary MDR-TB. Among childhood MDR-TB cases in South Africa, HIV infection was reported in 53.6, 77 and 22% in Johannesburg, Kwa-Zulu Natal and Cape Town [118–120]. HIV infection was independently associated with prevalent TB disease among child household contacts of adult MDR-TB cases and is associated with poorer outcome in child MDR-TB household contacts on MDR preventive therapy [121] HIV-infected children were also older and had more severe disease [122]. A recent review of adults and children with HIV and MDR-TB showed 83.4% treatment success in children [123]. It is becoming increasingly important to ensure that diagnostic, prevention and treatment strategies for MDR-TB are developed for HIV-infected children.

## Operational issues

Initially TB and HIV services were introduced as vertical programmes, but now strategies to integrate these services and strengthen the linkage to antenatal care are essential to improving diagnosis and access to care and treatment. HIV testing remains the entry point for HIV care. Ensuring that children diagnosed with TB are tested for HIV is absolutely essential. Given the high risk of HIV co-infection in children with TB in many settings, routine HIV testing of all children receiving TB treatment is an important opportunity to increase HIV diagnosis and thus achievement of 90% of HIV-infected persons knowing their status.

The diagnosis of TB in all children remains challenging. A study of paediatric ART programmes in diverse resource-limited settings showed that, although sputum microscopy and CXR were available in all programmes, they were only used in 86 and 52% of TB diagnoses. Xpert MTB/RIF was only used in 8% of TB diagnoses and mycobacterial culture in 17%. Eighty-six percent of sites provided access to TB treatment, but 30% never provided IPT to children [124]. Adult contact tracing and access to IPT remains a vital component of care in HIV-infected children regardless of the ART therapy. This undertaking starts with taking the appropriate history at each health care contact. HIV-infected children who have defaulted care may be picked up and re-enter care at TB services.

Screening of pregnant women for both HIV and TB may improve access not only to PMTCT, but also to IPT. Integrating TB services into antenatal care of HIV-infected women may also be key to preventing TB in young infants [125]. In Table 1 we highlight the components of care that require linkage and integration. Health care providers in all these settings need competency in all these aspects. Health system strengthening aimed at meeting the 90–90–90 targets will need to consider TB services and may have beneficial impacts for TB care in children as well.

**Table 1:** Components of care that require linkage and integration to deliver tuberculosis preventative and treatment services to HIV-infected children

	Tuberculosis services	HIV services	Antenatal care	General healthcare/IMCI
Screen for TB contact and link to IPT		✓		✓
Trace contacts	✓	✓	✓	
Screen for TB		✓	✓	✓
Diagnose TB	✓	✓	✓	✓
Diagnose HIV	✓	✓	✓	✓
Treat TB/link to TB care	✓	✓	✓	✓
Provide ART link to ART care	✓		✓	✓
Adapt drug choices to accommodate rifamycin	✓	✓		✓
Ensure adherence to all therapies	✓	✓		✓

IMCI: Integrated Management of Childhood Illness; TB: tuberculosis; IPT: isoniazid preventative therapy; ART: antiretroviral therapy.

## Conclusions

The TB and HIV epidemics remain closely interlinked and TB is still an important opportunistic infection in HIV-infected children, with substantial mortality and morbidity and with co-treatment possibly affecting HIV outcomes. The growing burden of drug-resistant TB poses new challenges. Increasing ART access has the potential to greatly impact the TB epidemic in settings with high dual burden. The reduction in adult cases will reduce TB infection to both HIV-infected and -uninfected infants and children. Early ART in HIV-infected children will further reduce the burden of TB in these children. The provision of IPT should be strengthened, and innovative prevention strategies such as influenza and pneumococcal vaccination and continuing co-trimoxazole should be explored. Treating these diseases simultaneously presents challenges with regards to choosing the most appropriate regimens and ensuring that medications are available in all settings and easy for children to adhere to. There is a synergy between working towards the 90–90–90 targets and improving TB diagnostic and treatment programmes. Efforts to meet the 90–90–90 targets for both adults and children may well have a profound impact on the burden of childhood TB, while improved prevention, diagnosis and treatment of TB in co-infected children, as well as strengthening and integration of TB-HIV programmes, will be important if the 90–90–90 targets are to be achieved.

## References

[1] World Health Organization Global update on the health sector response to HIV.

[2] UNAIDS 90–90–90 An ambitious treatment target to help end the AIDS epidemic.

[3] World Health Organization Global tuberculosis report.

[4] Dodd PJ, Gardiner E, Coghlan R, Seddon JA (2014). Burden of childhood tuberculosis in 22 high-burden countries: a mathematical modelling study. Lancet Glob Health.

[5] Venturini E, Turkova A, Chiappini E, Galli L, de Martino M, Thorne C (2014). Tuberculosis and HIV co-infection in children. BMC Infect Dis.

[6] World Health Organization Guidance for national tuberculosis programmes on the management of tuberculosis in children. Second Edition.

[7] Lawn SD, Bekker LG, Middelkoop K, Myer L, Wood R (2006). Impact of HIV infection on the epidemiology of tuberculosis in a peri-urban community in South Africa: the need for age-specific interventions. Clin Infect Dis.

[8] Gupta A, Nayak U, Ram M, Bhosale R, Patil S, Basavraj A (2007). Postpartum tuberculosis incidence and mortality among HIV-infected women and their infants in Pune, India, 2002–2005. Clin Infect Dis.

[9] Gupta A, Chandrasekhar A, Gupte N, Patil S, Bhosale R, Sambarey P (2011). Symptom screening among HIV-infected pregnant women is acceptable and has high negative predictive value for active tuberculosis. Clin Infect Dis.

[10] Gupta A, Bhosale R, Kinikar A, Gupte N, Bharadwaj R, Kagal A (2011). Maternal tuberculosis: a risk factor for mother-to-child transmission of human immunodeficiency virus. J Infect Dis.

[11] Cotton MF, Schaaf HS, Lottering G, Weber HL, Coetzee J, Nachman S (2008). Tuberculosis exposure in HIV-exposed infants in a high-prevalence setting. Int J Tuberc Lung Dis.

[12] Madhi SA, Huebner RE, Doedens L, Aduc T, Wesley D, Cooper PA (2000). HIV-1 co-infection in children hospitalised with tuberculosis in South Africa. Int J Tuberc Lung Dis.

[13] Hesseling AC, Cotton MF, Jennings T, Whitelaw A, Johnson LF, Eley B (2009). High incidence of tuberculosis among HIV-infected infants: evidence from a South African population-based study highlights the need for improved tuberculosis control strategies. Clin Infect Dis.

[14] Ciaranello A, Lu Z, Ayaya S, Losina E, Musick B, Vreeman R (2014). Incidence of World Health Organization stage 3 and 4 events, tuberculosis and mortality in untreated, HIV-infected children enrolling in care before 1 year of age: an IeDEA (International Epidemiologic Databases To Evaluate AIDS) East Africa regional analysis. Pediatr Infect Dis J.

[15] Braitstein P, Nyandiko W, Vreeman R, Wools-Kaloustian K, Sang E, Musick B (2009). The clinical burden of tuberculosis among human immunodeficiency virus-infected children in Western Kenya and the impact of combination antiretroviral treatment. Pediatr Infect Dis J.

[16] Frigati LJ, Kranzer K, Cotton MF, Schaaf HS, Lombard CJ, Zar HJ (2011). The impact of isoniazid preventive therapy and antiretroviral therapy on tuberculosis in children infected with HIV in a high tuberculosis incidence setting. Thorax.

[17] Violari A, Cotton MF, Gibb DM, Babiker AG, Steyn J, Madhi SA (2008). Early antiretroviral therapy and mortality among HIV-infected infants. N Engl J Med.

[18] Walters E, Cotton MF, Rabie H, Schaaf HS, Walters LO, Marais BJ (2008). Clinical presentation and outcome of tuberculosis in human immunodeficiency virus infected children on antiretroviral therapy. BMC Pediatr.

[19] Bates M, Mudenda V, Mwaba P, Zumla A (2013). Deaths due to respiratory tract infections in Africa: a review of autopsy studies. Curr Opin Pulm Med.

[20] Cohen JM, Whittaker E, Walters S, Lyall H, Tudor-Williams G, Kampmann B (2008). Presentation, diagnosis and management of tuberculosis in HIV-infected children in the UK. HIV Med.

[21] Thomas P, Bornschlegel K, Singh TP, Abrams EJ, Cervia J, Fikrig S (2000). Tuberculosis in human immunodeficiency virus-infected and human immunodeficiency virus-exposed children in New York City. The New York City Pediatric Spectrum of HIV Disease Consortium. Pediatr Infect Dis J.

[22] Walters E, Duvenhage J, Draper HR, Hesseling AC, Van Wyk SS, Cotton MF (2014). Severe manifestations of extrapulmonary tuberculosis in HIV-infected children initiating antiretroviral therapy before 2 years of age. Arch Dis Child.

[23] Bakeera-Kitaka S, Conesa-Botella A, Dhabangi A, Maganda A, Kekitiinwa A, Colebunders R (2011). Tuberculosis in human immunodeficiency virus infected Ugandan children starting on antiretroviral therapy. Int J Tuberc Lung Dis.

[24] Edmonds A, Lusiama J, Napravnik S, Kitetele F, Van Rie A, Behets F (2009). Anti-retroviral therapy reduces incident tuberculosis in HIV-infected children. Int J Epidemiol.

[25] Krauss MR, Harris DR, Abreu T, Ferreira FG, Ruz NP, Worrell C (2015). Tuberculosis in HIV-infected infants, children, and adolescents in Latin America. Braz J Infect Dis.

[26] Martinson NA, Moultrie H, van Niekerk R, Barry G, Coovadia A, Cotton M (2009). HAART and risk of tuberculosis in HIV-infected South African children: a multi-site retrospective cohort. Int J Tuberc Lung Dis.

[27] Mu W, Zhao Y, Sun X, Ma Y, Yu L, Liu X (2014). Incidence and associated factors of pulmonary tuberculosis in HIV-infected children after highly active antiretroviral therapy (HAART) in China: a retrospective study. AIDS Care.

[28] Prasitsuebsai W, Kariminia A, Puthanakit T, Lumbiganon P, Hansudewechakul R, Siew Moy F (2014). Impact of antiretroviral therapy on opportunistic infections of HIV-infected children in the therapeutic research, education and AIDS training Asia pediatric HIV observational database. Pediatr Infect Dis J.

[29] Bolton-Moore C, Mubiana-Mbewe M, Cantrell RA, Chintu N, Stringer EM, Chi BH (2007). Clinical outcomes and CD4 cell response in children receiving antiretroviral therapy at primary health care facilities in Zambia. JAMA.

[30] Abuogi LL, Mwachari C, Leslie HH, Shade SB, Otieno J, Yienya N (2013). Impact of expanded antiretroviral use on incidence and prevalence of tuberculosis in children with HIV in Kenya. Int J Tuberc Lung Dis.

[31] Auld AF, Tuho MZ, Ekra KA, Kouakou J, Shiraishi RW, Adjorlolo-Johnson G (2014). Tuberculosis in human immunodeficiency virus-infected children starting antiretroviral therapy in Cote d'Ivoire. Int J Tuberc Lung Dis.

[32] Li N, Manji KP, Spiegelman D, Muya A, Mwiru RS, Liu E (2013). Incident tuberculosis and risk factors among HIV-infected children in Tanzania. AIDS.

[33] Orikiiriza J, Bakeera-Kitaka S, Musiime V, Mworozi EA, Mugyenyi P, Boulware DR (2010). The clinical pattern, prevalence, and factors associated with immune reconstitution inflammatory syndrome in Ugandan children. AIDS.

[34] Dangor Z, Izu A, Hillier K, Solomon F, Beylis N, Moore DP (2013). Impact of the antiretroviral treatment program on the burden of hospitalization for culture-confirmed tuberculosis in South African children: a time-series analysis. Pediatr Infect Dis J.

[35] Pretorius C, Menzies NA, Chindelevitch L, Cohen T, Cori A, Eaton JW (2014). The potential effects of changing HIV treatment policy on tuberculosis outcomes in South Africa: results from three tuberculosis-HIV transmission models. AIDS.

[36] Madhi SA, Nachman S, Violari A, Kim S, Cotton MF, Bobat R (2011). Effect of primary isoniazid prophylaxis against tuberculosis in HIV-exposed children. N Eng J Med.

[37] Colditz GA, Brewer TF, Berkey CS, Wilson ME, Burdick E, Fineberg HV (1994). Efficacy of BCG vaccine in the prevention of tuberculosis. Meta-analysis of the published literature. JAMA.

[38] Hesseling AC, Johnson LF, Jaspan H, Cotton MF, Whitelaw A, Schaaf HS (2009). Disseminated bacille Calmette-Guerin disease in HIV-infected South African infants. Bull World Health Organ.

[39] Rabie H, Violari A, Duong T, Madhi SA, Josipovic D, Innes S (2011). Early antiretroviral treatment reduces risk of bacille Calmette-Guérin immune reconstitution adenitis. Int J Tuberc Lung Dis.

[40] World Health Organization (2007). Revised BCG vaccination guidelines for infants at risk for HIV infection. Wkly Epidemiol Rec.

[41] Hesseling AC, Cotton MF, Fordham van Reyn C, Graham SM, Gie RP, Hussey GD (2008). Consensus statement on the revised World Health Organization recommendations for BCG vaccination in HIV-infected infants. Int J Tuberc Lung Dis.

[42] Hesseling AC, Caldwell J, Cotton MF, Eley BS, Jaspan HB, Jennings K (2009). BCG vaccination in South African HIV-exposed infants–risks and benefits. S Afr Med J.

[43] Marais BJ, Gie RP, Schaaf HS, Hesseling AC, Obihara CC, Starke JJ (2004). The natural history of childhood intra-thoracic tuberculosis: a critical review of literature from the pre-chemotherapy era. Int J Tuberc Lung Dis.

[44] Curry FJ (1967). Prophylactic effect of isoniazid in young tuberculin reactors. N Engl J Med.

[45] Hsu KH (1974). Isoniazid in the prevention and treatment of tuberculosis. A 20-year study of the effectiveness in children. JAMA.

[46] Zar HJ, Cotton MF, Strauss S, Karpakis J, Hussey G, Schaaf HS (2007). Effect of isoniazid prophylaxis on mortality and incidence of tuberculosis in children with HIV: randomised controlled trial. BMJ.

[47] Van Wyk SS, Hamade H, Hesseling AC, Beyers N, Enarson DA, Mandalakas AM (2010). Recording isoniazid preventive therapy delivery to children: operational challenges. Int J Tuberc Lung Dis.

[48] Rutherford ME, Ruslami R, Maharani W, Yulita I, Lovell S, Van Crevel R (2012). Adherence to isoniazid preventive therapy in Indonesian children: a quantitative and qualitative investigation. BMC Res Notes.

[49] van Soelen N, du Preez K, van Wyk SS, Mandalakas AM, Enarson DA, Reid AJ (2013). Does an isoniazid prophylaxis register improve tuberculosis contact management in South African children?. PLoS One.

[50] Adams LV, Talbot EA, Odato K, Blunt H, Steingart KR (2014). Interventions to improve delivery of isoniazid preventive therapy: an overview of systematic reviews. BMC Infect Dis.

[51] Sharma SK, Sharma A, Kadhiravan T, Tharyan P (2014). Rifamycins (rifampicin, rifabutin and rifapentine) compared to isoniazid for preventing tuberculosis in HIV-negative people at risk of active TB. Evid Based Child Health.

[52] Villarino ME, Scott NA, Weis SE, Weiner M, Conde MB, Jones B (2015). Treatment for preventing tuberculosis in children and adolescents: a randomized clinical trial of a 3-month, 12-dose regimen of a combination of rifapentine and isoniazid. JAMA Pediatr.

[53] Samandari T, Agizew TB, Nyirenda S, Tedla Z, Sibanda T, Shang N (2011). 6-month versus 36-month isoniazid preventive treatment for tuberculosis in adults with HIV infection in Botswana: a randomised, double-blind, placebo-controlled trial. Lancet.

[54] Hesseling AC, Kim S, Madhi S, Nachman S, Schaaf HS, Violari A (2012). High prevalence of drug resistance amongst HIV-exposed and -infected children in a tuberculosis prevention trial. Int J Tuberc Lung Dis.

[55] Dangor Z, Izu A, Moore DP, Nunes MC, Solomon F, Beylis N (2014). Temporal association in hospitalizations for tuberculosis, invasive pneumococcal disease and influenza virus illness in South African children. PloS One.

[56] Moore DP, Klugman KP, Madhi SA (2010). Role of Streptococcus pneumoniae in hospitalization for acute community-acquired pneumonia associated with culture-confirmed Mycobacterium tuberculosis in children: a pneumococcal conjugate vaccine probe study. Pediatr Infect Dis J.

[57] Forgacs P, Wengenack NL, Hall L, Zimmerman SK, Silverman ML, Roberts GD (2009). Tuberculosis and trimethoprim-sulfamethoxazole. Antimicrob Agents Chemother.

[58] Hasse B, Walker AS, Fehr J, Furrer H, Hoffmann M, Battegay M (2014). Co-trimoxazole prophylaxis is associated with reduced risk of incident tuberculosis in participants in the Swiss HIV Cohort Study. Antimicrob Agents Chemother.

[59] Hoffmann CJ, Chaisson RE, Martinson NA (2014). Cotrimoxazole prophylaxis and tuberculosis risk among people living with HIV. PloS One.

[60] Bwakura-Dangarembizi M, Kendall L, Bakeera-Kitaka S, Nahirya-Ntege P, Keishanyu R, Nathoo K (2014). A randomized trial of prolonged co-trimoxazole in HIV-infected children in Africa. N Engl J Med.

[61] Rennert WP, Kilner D, Hale M, Stevens G, Stevens W, Crewe-Brown H (2002). Tuberculosis in children dying with HIV-related lung disease: clinical- pathological correlations. Int J Tuberc Lung Dis.

[62] Frohoff C, Moodley M, Fairlie L, Coovadia A, Moultrie H, Kuhn L (2011). Antiretroviral therapy outcomes in HIV-infected children after adjusting protease inhibitor dosing during tuberculosis treatment. PloS One.

[63] Reitz C, Coovadia A, Ko S, Meyers T, Strehlau R, Sherman G (2010). Initial response to protease-inhibitor-based antiretroviral therapy among children less than 2 years of age in South Africa: effect of cotreatment for tuberculosis. J Infect Dis.

[64] Zanoni BC, Phungula T, Zanoni HM, France H, Feeney ME (2011). Impact of tuberculosis cotreatment on viral suppression rates among HIV-positive children initiating HAART. AIDS.

[65] Meyers T, Sawry S, Wong JY, Moultrie H, Pinillos F, Fairlie L (2015). Virologic failure among children taking lopinavir/ritonavir-containing first-line antiretroviral therapy in South Africa. Pediatr Infect Dis J.

[66] Walters E, Reichmuth K, Dramowski A, Marais BJ, Cotton MF, Rabie H (2013). Antiretroviral regimens containing a single protease inhibitor increase risk of virologic failure in young HIV-infected children. Pediatr Infect Dis J.

[67] Lowenthal ED, Ellenberg JH, Machine E, Sagdeo A, Boiditswe S, Steenhoff AP (2013). Association between efavirenz-based compared with nevirapine-based antiretroviral regimens and virological failure in HIV-infected children. JAMA.

[68] van Dijk JH, Sutcliffe CG, Hamangaba F, Bositis C, Watson DC, Moss WJ (2013). Effectiveness of efavirenz-based regimens in young HIV-infected children treated for tuberculosis: a treatment option for resource-limited settings. PloS One.

[69] Arrow Trial Team (2013). Routine versus clinically driven laboratory monitoring and first-line antiretroviral therapy strategies in African children with HIV (ARROW): a 5-year open-label randomised factorial trial. Lancet.

[70] World Health Organization Rapid advice. Treatment of tuberculosis in children.

[71] Hill S, Regondi I, Grzemska M, Matiru R (2008). Children and tuberculosis medicines: bridging the research gap. Bull World Health Organ.

[72] Thee S, Seddon JA, Donald PR, Seifart HI, Werely CJ, Hesseling AC (2011). Pharmacokinetics of isoniazid, rifampin, and pyrazinamide in children younger than two years of age with tuberculosis: evidence for implementation of revised World Health Organization recommendations. Antimicrob Agents Chemother.

[73] Hiruy H, Rogers Z, Mbowane C, Adamson J, Ngotho L, Karim F (2015). Subtherapeutic concentrations of first-line anti-TB drugs in South African children treated according to current guidelines: the PHATISA study. J Antimicrob Chemother.

[74] Garcia-Prats AJ, Willemse M, Seifart HI, Jordaan AM, Werely CJ, Donald PR (2014). Acquired drug resistance during inadequate therapy in a young child with tuberculosis. Pediatr Infect Dis J.

[75] Donald PR, Maher D, Maritz JS, Qazi S (2006). Ethambutol dosage for the treatment of children: literature review and recommendations. Int J Tuberc Lung Dis.

[76] Levy M, Rigaudiere F, de Lauzanne A, Koehl B, Melki I, Lorrot M (2015). Ethambutol-related impaired visual function in children less than 5 years of age treated for a mycobacterial infection: diagnosis and evolution. Pediatr Infect Dis J.

[77] Seddon JA, Hesseling AC, Marais BJ, McIlleron H, Peloquin CA, Donald PR (2012). Paediatric use of second-line anti-tuberculosis agents: a review. Tuberculosis.

[78] Thee S, Garcia-Prats AJ, McIlleron HM, Wiesner L, Castel S, Norman J (2014). Pharmacokinetics of ofloxacin and levofloxacin for prevention and treatment of multidrug-resistant tuberculosis in children. Antimicrob Agents Chemother.

[79] Thee S, Garcia-Prats AJ, Draper HR, McIlleron HM, Wiesner L, Castel S (2015). Pharmacokinetics and safety of moxifloxacin in children with multidrug-resistant tuberculosis. Clin Infect Dis.

[80] Coyne KM, Pozniak AL, Lamorde M, Boffito M (2009). Pharmacology of second-line antituberculosis drugs and potential for interactions with antiretroviral agents. AIDS.

[81] de Kock L, Sy SK, Rosenkranz B, Diacon AH, Prescott K, Hernandez KR (2014). Pharmacokinetics of para-aminosalicylic acid in HIV-uninfected and HIV-coinfected tuberculosis patients receiving antiretroviral therapy, managed for multidrug-resistant and extensively drug-resistant tuberculosis. Antimicrob Agents Chemother.

[82] Thee S, Zollner EW, Willemse M, Hesseling AC, Magdorf K, Schaaf HS (2011). Abnormal thyroid function tests in children on ethionamide treatment. Int J Tuberc Lung Dis.

[83] Rose PC, Hallbauer UM, Seddon JA, Hesseling AC, Schaaf HS (2012). Linezolid-containing regimens for the treatment of drug-resistant tuberculosis in South African children. Int J Tuberc Lung Dis.

[84] World Health Organization The use of bedaquiline in the treatment of multidrug resistant tuberculosis. Interim policy guidance.

[85] World Health Organization The use of delamanid in the treatment of multidrug-resistant tuberculosis: interim policy guidance.

[86] Svensson EM, Murray S, Karlsson MO, Dooley KE (2015). Rifampicin and rifapentine significantly reduce concentrations of bedaquiline, a new anti-TB drug. J Antimicrob Chemother.

[87] Svensson EM, Dooley KE, Karlsson MO (2014). Impact of lopinavir-ritonavir or nevirapine on bedaquiline exposures and potential implications for patients with tuberculosis-HIV coinfection. Antimicrob Agents Chemother.

[88] Svensson EM, Aweeka F, Park JG, Marzan F, Dooley KE, Karlsson MO (2013). Model-based estimates of the effects of efavirenz on bedaquiline pharmacokinetics and suggested dose adjustments for patients coinfected with HIV and tuberculosis. Antimicrob Agents Chemother.

[89] Violari A, Lindsey JC, Hughes MD, Mujuru HA, Barlow-Mosha L, Kamthunzi P (2012). Nevirapine versus ritonavir-boosted lopinavir for HIV-infected children. N Engl J Med.

[90] Palumbo P, Lindsey J, Hughes MD, Cotton MF, Bobat R, Meyers T (2010). Antiretroviral Treatment for Children with Peripartum Nevirapine Exposure. N Engl J Med.

[91] World Health Organization Consolidated guidelines on the use of antiretroviral drugs for treating and preventing HIV infection: recommendations for a public health approach.

[92] Maartens G, Decloedt E, Cohen K (2009). Effectiveness and safety of antiretrovirals with rifampicin: crucial issues for high-burden countries. Antivir Ther.

[93] Ren Y, Nuttall JJ, Egbers C, Eley BS, Meyers TM, Smith PJ (2008). Effect of rifampicin on lopinavir pharmacokinetics in HIV-infected children with tuberculosis. J Acquir Immune Defic Syndr.

[94] Rabie HDP, Lee J, Kindra G, Coovadia A, Pillay S, Liberty A (2015). Lopinavir ritonavir (1:1 ratio) in the presence of rifampicin is not inferior to lopinavir ritonavir (4:1 ratio) in the absence of rifampicin in Human Immune Deficiency Virus (HIV) children.

[95] Decloedt EH, McIlleron H, Smith P, Merry C, Orrell C, Maartens G (2011). Pharmacokinetics of lopinavir in HIV-infected adults receiving rifampin with adjusted doses of lopinavir-ritonavir tablets. Antimicrob Agents Chemother.

[96] McIlleron H, Ren Y, Nuttall J, Fairlie L, Rabie H, Cotton M (2011). Lopinavir exposure is insufficient in children given double doses of lopinavir/ritonavir during rifampicin-based treatment for tuberculosis. Antivir Ther.

[97] Zhang C, Denti P, Decloedt EH, Ren Y, Karlsson MO, McIlleron H (2013). Model-based evaluation of the pharmacokinetic differences between adults and children for lopinavir and ritonavir in combination with rifampicin. Br J Clin Pharmacol.

[98] Moultrie H, McIlleron H, Sawry S, Kellermann T, Wiesner L, Kindra G (2015). Pharmacokinetics and safety of rifabutin in young HIV-infected children receiving rifabutin and lopinavir/ritonavir. J Antimicrob Chemother.

[99] Burger DM, Agarwala S, Child M, Been-Tiktak A, Wang Y, Bertz R (2006). Effect of rifampin on steady-state pharmacokinetics of atazanavir with ritonavir in healthy volunteers. Antimicrob Agents Chemother.

[100] Dickinson L, Winston A, Boffito M, Khoo S, Back D, Siccardi M (2014). Simulation of the impact of rifampicin on darunavir/ritonavir PK and dose adjustment strategies in HIV-infected patients: a population PK approach. J Int AIDS Soci.

[101] Ren Y, Nuttall JJ, Eley BS, Meyers TM, Smith PJ, Maartens G (2009). Effect of rifampicin on efavirenz pharmacokinetics in HIV-infected children with tuberculosis. J Acquir Immune Defic Syndr.

[102] McIlleron HM, Schomaker M, Ren Y, Sinxadi P, Nuttall JJ, Gous H (2013). Effects of rifampin-based antituberculosis therapy on plasma efavirenz concentrations in children vary by CYP2B6 genotype. AIDS.

[103] Panel on Antiretroviral Guidelines for Adults and Adolescents Guidelines for the use of antiretroviral agents in HIV-1-infected adults and adolescents.

[104] Prasitsuebsai W, Cressey TR, Capparelli E, Vanprapar N, Lapphra K, Chokephaibulkit K (2012). Pharmacokinetics of nevirapine in HIV and tuberculosis-coinfected children receiving antiretroviral fixed-dose combination tablets while receiving rifampicin-containing tuberculosis treatment and after rifampicin discontinuation. Pediatr Infect Dis J.

[105] Oudijk JM, McIlleron H, Mulenga V, Chintu C, Merry C, Walker AS (2012). Pharmacokinetics of nevirapine in HIV-infected children under 3 years on rifampicin-based antituberculosis treatment. AIDS.

[106] Barlow-Mosha L, Musoke P, Parsons T, Ajuna P, Lutajjumwa M, Musoke B (2009). Nevirapine concentrations in HIV infected Ugandan children on adult fixed dose combination tablet (Triomune) antiretroviral treatment; with and without Rifampicin based treatment for active M. Tuberculosis Infection.

[107] Fillekes Q, Mulenga V, Kabamba D, Kankasa C, Thomason MJ, Cook A (2013). Is nevirapine dose escalation appropriate in young, African, HIV-infected children?. AIDS.

[108] McIlleron HDP, Mashabela F, Hunt J, Shembe S, Msandiwa R, Velaphi S (2013). Effects of maternal tuberculosis treatment on nevirapine concentrations among neonates receiving nevirapine for prevention of mother to-child transmission of HIV international AIDS Society Conference.

[109] Coovadia A, Abrams E, Strehlau R, Shiau S, Pinillos F, Martens L (2014). NEVEREST Study Team. Virologic efficacy of efavirenz maintenance therapy in nevirapine prophylaxis-exposed children.

[110] Gagliardini R, Fabbiani M, Fortuna S, Visconti E, Navarra P, Cauda R (2014). Pharmacokinetics of etravirine in HIV-infected patients concomitantly treated with rifampin for tuberculosis. Infection.

[111] Wenning LA, Hanley WD, Brainard DM, Petry AS, Ghosh K, Jin B (2009). Effect of rifampin, a potent inducer of drug-metabolizing enzymes, on the pharmacokinetics of raltegravir. Antimicrob Agents Chemother.

[112] Vernon A, Burman W, Benator D, Khan A, Bozeman L (1999). Acquired rifamycin monoresistance in patients with HIV-related tuberculosis treated with once-weekly rifapentine and isoniazid, Tuberculosis Trials Consortium. Lancet.

[113] Abdool Karim SS, Naidoo K, Grobler A, Padayatchi N, Baxter C, Gray A (2010). Timing of initiation of antiretroviral drugs during tuberculosis therapy. N Engl J Med.

[114] Yotebieng M, Van Rie A, Moultrie H, Cole SR, Adimora A, Behets F (2010). Effect on mortality and virological response of delaying antiretroviral therapy initiation in children receiving tuberculosis treatment. AIDS.

[115] Bahr N, Boulware DR, Marais S, Scriven J, Wilkinson RJ, Meintjes G (2013). Central nervous system immune reconstitution inflammatory syndrome. Curr Infect Dis Rep.

[116] van Toorn R, Rabie H, Dramowski A, Schoeman JF (2012). Neurological manifestations of TB-IRIS: a report of 4 children. Eur J Paediatr Neurol.

[117] van Well GT, Paes BF, Terwee CB, Springer P, Roord JJ, Donald PR (2009). Twenty years of pediatric tuberculous meningitis: a retrospective cohort study in the western cape of South Africa. Pediatrics.

[118] Seddon JA, Hesseling AC, Willemse M, Donald PR, Schaaf HS (2012). Culture-confirmed multidrug-resistant tuberculosis in children: clinical features, treatment, and outcome. Clin Infect Dis.

[119] Hicks RM, Padayatchi N, Shah NS, Wolf A, Werner L, Sunkari VB (2014). Malnutrition associated with unfavorable outcome and death among South African MDR-TB and HIV co-infected children. Int J Tuberc Lung Dis.

[120] Fairlie L, Beylis NC, Reubenson G, Moore DP, Madhi SA (2011). High prevalence of childhood multi-drug resistant tuberculosis in Johannesburg, South Africa: a cross sectional study. BMC Infect Dis.

[121] Seddon JA, Hesseling AC, Finlayson H, Fielding K, Cox H, Hughes J (2013). Preventive therapy for child contacts of multidrug-resistant tuberculosis: a prospective cohort study. Clin Infect Dis.

[122] Seddon JA, Hesseling AC, Godfrey-Faussett P, Schaaf HS (2014). High treatment success in children treated for multidrug-resistant tuberculosis: an observational cohort study. Thorax.

[123] Isaakidis P, Casas EC, Das M, Tseretopoulou X, Ntzani EE, Ford N (2015). Treatment outcomes for HIV and MDR-TB co-infected adults and children: systematic review and meta-analysis. Int J Tuberc Lung Dis.

[124] Ballif M, Nhandu V, Wood R, Dusingize JC, Carter EJ, Cortes CP (2014). Detection and management of drug-resistant tuberculosis in HIV-infected patients in lower-income countries. Int J Tuberc Lung Dis.

[125] Getahun H, Sculier D, Sismanidis C, Grzemska M, Raviglione M (2012). Prevention, diagnosis, and treatment of tuberculosis in children and mothers: evidence for action for maternal, neonatal, and child health services. J Infect Dis.

# Sub article: Patching the gaps towards the 90–90–90 targets: outcomes of Nigerian children receiving antiretroviral treatment who are co-infected with tuberculosis

**DOI:** 10.7448/IAS.18.7.20251

**Published:** 2015-12-02

**Authors:** Dick D Chamla, Chukwuemeka Asadu, Abiola Davies, Arjan de Wagt, Oluwafunke Ilesanmi, Daniel Adeyinka, Ebun Adejuyigbe

**Affiliations:** 1 Health Section, UNICEF, New York, NY, USA; 2 Federal Ministry of Health, NASCP, Abuja, Nigeria; 3 Country Office, UNICEF, Abuja, Nigeria; 4 World Health Organization, Abuja, Nigeria; 5 Obafemi Awolowo University, Ile-Ife, Nigeria

**Keywords:** children, tuberculosis, HIV, ART

## Abstract

**Introduction:**

Nigeria has a high burden of children living with HIV and tuberculosis (TB). This article examines the magnitude of TB among children receiving antiretroviral treatment (ART), compares their ART outcomes with their non-TB counterparts and argues that addressing TB among children on ART is critical for achieving the 90–90–90 targets.

**Methods:**

This was a facility-based, retrospective analysis of medical records of children aged <15 years who were newly initiated on ART between 2011 and 2012. Structured tools were used to collect data. STATA software was used to perform descriptive, survival and multivariate analyses.

**Results:**

A total of 1142 children with a median age of 3.5 years from 20 selected facilities were followed for 24 months. Of these, 95.8% were assessed for TB at ART initiation and 14.7% had TB. Children on ART were more likely to have TB if they were aged 5 years or older (*p<*0.01) and had delayed ART initiation (*p<*0.05). The cotrimoxazole and isoniazid prophylaxes were provided to 87.9 and 0.8% of children, respectively. The rate of new TB cases was 3 (2.2–4.0) per 100 person-years at six months and declined to 0.2 (0.06–1.4) per 100 person-years at 24 months. TB infection [adjusted hazard ratio (aHR): 4.3; 2.3–7.9], malnutrition (aHR: 5.1; 2.6–9.8), delayed ART initiation (aHR: 3.2; 1.5–6.7) and age less than 1 year at ART initiation (aHR: 4.0; 1.4–12.0) were associated with death. Additionally, patients with TB (aHR: 1.3; 1.1–1.6) and children below the age of 1 at ART initiation (aHR: 2.9; 1.7–5.2) were more likely to be lost to follow-up (LFU).

**Conclusions:**

Children on ART with TB are less likely to survive and more likely to be LFU. These risks, along with low isoniazid uptake and delayed ART initiation, present a serious challenge to achieving the 90–90–90 targets and underscore an urgent need for inclusion of childhood TB/HIV in global plans and reporting mechanisms.

## Introduction

Tuberculosis (TB) is common in countries that have a high burden of children living with HIV and high under-five mortality [1]. Yet, TB/HIV co-morbidity among children has been largely overlooked globally and grossly under-reported. In most countries, the proportion of children with TB/HIV co-morbidity remains unknown. The most recent global WHO TB report could not disaggregate data on the burden or trends of TB/HIV among children [2]. Despite advances in early infant diagnosis of HIV infections, there are still challenges in diagnosing TB in children [3]. These diagnostic challenges are further exacerbated in children with HIV because clinical and radiological manifestations of TB are modified [4]. Additionally, opportunities for identifying HIV-infected children in child survival programs, including TB clinics, continue to be missed [5]. TB co-treatment in children with HIV presents a significant challenge for children aged less than three years, due to interaction of rifampicin with lopinavir/ritonavir and nevirapine [6,7]. Despite these obstacles, there is reason for optimism in tackling childhood TB/HIV supported by the presence of the global Roadmap for Childhood Tuberculosis with a goal of zero TB deaths [8], a Double Dividend initiative with the dual goal of improving paediatric HIV and child survival rates [9] and an ambitious post-2015 WHO TB strategy [10] that attempts to engage broader maternal and child health communities.

In Nigeria, the proportion of notified TB cases with HIV-positive test results was 22% in 2013 [2], although large variations among states have been reported [11]. High mortality among populations with TB/HIV has been well documented [12,13]. The coverage of paediatric antiretroviral treatment (ART) remains low [14], while high lost to follow-up (LFU) rates among children on ART has also been reported [15]. With high rates of mother-to-child transmission of HIV [14], the number of children living with HIV and in need of ART will continue to rise, yet data on the burden and outcomes of TB-infected children receiving ART remain limited. This article aims to examine the magnitude of TB among children starting ART and to compare their ART outcomes with non-TB-infected children. We argue that addressing TB is critical for accelerating progress towards the new global targets of 90–90–90 for the diagnosis, treatment and viral suppression needed to improve survival of children living with HIV.

## Methods

This was a facility-based, retrospective analysis of medical records of a cohort of children from selected health facilities who were newly initiated on ART between 1 January 2011 and 31 December 2012. This analysis was conducted as part of larger paediatric HIV assessment supported by United Nations Children Fund and the World Health Organization, in collaboration with the Nigeria Federal Ministry of Health and members of academic institutions and partners from the national paediatric ART technical working group. The eligibility criteria were strictly limited to children aged <15 years who had initiated ART between 1 January 2011 and 31 December 2012. The maximum follow-up period was 24 months after ART initiation. At the time of the assessment, Nigeria ART guidelines were based on the 2010 WHO guidelines that recommended early infant diagnosis at six weeks for all HIV-exposed infants and initiation of ART for all HIV-infected children under the age of 2 years, at CD4 count thresholds of ≤750 cells/mm^3^ or %CD4+≤25 for children aged 24 to 59 months and ≤350 cells/mm^3^ for children aged 5 years and older [16].

### TB diagnosis

TB screening and diagnosis in facilities followed the revised *National Tuberculosis and Leprosy Control Programme Workers’ Manual* (5th edition). A symptomatic checklist established TB suspects using the following symptoms: cough for two weeks or more; unexplained weight loss; failure to thrive and/or malnutrition; and history of contact with a TB case. A TB diagnosis was determined by a clinician based on sputum results for children who produced sputum radiological examination or culture. The study only collected data on TB assessment and confirmed TB diagnoses as recorded in ART registers. Data on TB suspects and diagnostic methods used were not routinely recorded in ART registers and were not included in the analysis.

### Sampling and data sources

Purposive sampling was used to select a total of 20 public and private facilities offering paediatric ART services from five states (Anambra, Bayelsa, Benue, Kano and Lagos). These represented a mixture of high and low HIV burden in five geopolitical zones. The list of facilities were drawn from the Nigerian health facility directory and sorted by key domains for wide representation. These included urban/rural location and ownership of facility (public/private/faith-based). Facility level was not included as a criterion, as only tertiary and general hospitals formally initiate paediatric ART in Nigeria. All eligible children who had newly initiated ART between January 2011 and December 2012 were selected for analysis.

The primary sources of paediatric ART data were paper-based or electronic HIV care/ART patients’ cards, patient charts and ART registers that contained data elements for TB assessments and diagnoses. Patient-level medical records were extracted into a structured data collection tool in English. Collected data included demographic characteristics, clinical and laboratory reports, TB status, uptake of isoniazid (INH) and cotrimoxazole, ART regimens used and ART outcomes.

### Outcome measures

The primary outcome measures were retention, LFU and death recorded at 6, 12 and 24 months following initiation of ART, comparing patients on ART with TB to those without TB at baseline. The definition of *LFU* followed the criteria articulated in the national ART guidelines, which is missed clinic appointments or pharmacy antiretroviral (ARV) refills for 90 days following last scheduled appointment as shown in the patient's records. Secondary outcome measures included new TB occurrence during HIV treatment at 6, 12 and 24 months after initiation of ART and uptake of INH and cotrimoxazole.

Individual level factors that often influence retention, LFU and deaths based on various literature were selected for analysis [17]. CD4 percentage or counts were measured at ART initiation, 6, 12 and 24 months to determine the degree of immunosuppression. Based on the 2010 WHO guidelines, *severe immunosuppression* was defined as an initial CD4 count less than 750 cells/mm^3^ or a percentage less than 15% for patients less than two years of age, less than 500 cells/mm^3^ or a percentage less than 15% for patients between two and five years of age and less than 200 cells/mm^3^ or a percentage less than 15% for patients five years or older. Other categories of immunosuppression, *moderate* and *no immunosuppression* as articulated in the WHO guidelines, were also analyzed. For malnutrition we calculated weight-for-age *z*-scores and categorized children as *z*-score less than or greater than −2 standard deviation [18].

Time to ART initiation was estimated by subtracting the dates of HIV diagnosis from ART initiation. We followed Kim *et al*.'s definition of *prompt initiation of ART* as those children initiating ART within 21 days [19]. Other individual factors included for analysis were age, referral sources (entry points) to ART, age and ARV regimens at ART initiation, sex and facility ownership (public/private).

### Data analysis

Kaplan–Meier survival and Nelson–Aalen cumulative hazard analyses were used to estimate survival and LFU probabilities, respectively, through 24 months after ART initiations comparing children on ART who had TB and non-TB counterparts. Bivariate and multivariate Cox proportion hazard models were used to establish the relationships between the individual factors mentioned above and primary outcome measures. Using the survival analysis function, the rates and their corresponding 95% confidence intervals (CI) of new TB cases, LFU and deaths were estimated and expressed per 100 person-years. The statistical associations between dependent and independent variables were estimated using hazard ratios (HR) for survival analysis, while odds ratios (OR) with 95% CI were used to establish correlates of TB at ART initiation. The proportional hazards assumption were assessed by graphical methods and Harrell's C measure of concordance probability. Statistical significance was determined at *α<*0.05. All statistical analyses were completed using STATA version IC 11 (StataCorp LP, College Station, TX, USA).

### Ethical considerations

Ethical clearance for the study was granted by National Health Research Ethics Committee of Nigeria (NHREC). Formal requests and approvals to visit facilities and abstract data were also granted by the Federal and State Ministries of Health. Confidentiality of patient records was assured by the use of unique number allocation and removal of personal identifiers.

## Results

The records from 1142 children aged less than 15 years who were newly initiated on ART between January 2011 and December 2012 were analyzed from 20 selected facilities. The majority of records, 984 (86.4%), were from public/government facilities, and less than 1.1% were from private for-profit facilities. Most (62.6%) initiated ART at tertiary facilities, with a median age (at ART initiation) of 3.5 years [interquartile ratio (IQR): 1.6–7.2] and 618 (54.7%) being males. Approximately 16.4% of those initiating ART were less than one year of age, 44.9% were between one and five years of age, 25.4% were aged between five and ten years and 13.2% were aged 10 years and above. At baseline, 50.1% (507/1002) of children with CD4 results were severely immunocompromised and 12.1% were malnourished. The most-used regimen at ART initiation was AZT/3TC/NVP (75.5%) followed by AZT/3TC/EFV (13.1%). Only 1.1% of patients were initiated with ritonavir-boosted lopinavir-based regimens.

### TB burden

The TB status was assessed and determined in 1094 (95.8%) patients. Of these, 161 (14.7%) were diagnosed as having active TB. In multivariate regression adjusting for other factors, children on ART were more likely to be diagnosed with TB if they were five years old or above at ART initiation (*p<*0.01) and had delayed ART initiation (*p<*0.05). There was no significant statistical difference in severe immunosuppression between children with and without TB in the final multivariate regression model (*p>*0.05) despite its independent association with TB following bivariate analysis (Table 1).

**Table 1:** Correlates of TB among children on ART

Factor	Co-infected with TB	No TB	OR (95% CI)	*p*	aOR (95% CI)	*p*
Age at ART initiation (*n*=1094)	161	933				
<1 year (reference)	16 (9.9%)	154 (16.5%)				
2 to <5 years	57 (35.4%)	419 (44.9%)	1.43 (0.78–2.60)	0.25	1.74 (0.65–1.81)	0.28
5 to 9 years	63 (39.1%)	237 (25.4%)	3.18 (1.75–5.81)	0.001	4.41 (1.61–12.07)	0.004
10 to 14 years	25 (15.5%)	123 (13.2%)	2.36 (1.18–4.72)	0.02	3.53 (1.20–10.35)	0.022
Sex (*n*=1081)	160	921				
Female (reference)	69 (43.1%)	414 (45%)				
Male	91 (56.9%)	507 (55%)	1.08 (0.77–1.51)	0.67		
Facility ownership (*n*=1091)	160	931				
Public/government (reference)	148 (92.5%)	810 (87%)				
Private not for profit	11 (6.9%)	110 (11.8%)	0.55 (0.29–1.04)	0.07		
Private for profit	1 (0.6%)	11 (1.2%)	0.49 (0.06–3.88)	0.51		
CD4 at ART initiation (*n*=1002)	131	871				
Severe immunosuppression	76 (58%)	431 (49.5%)	3.49 (1.74–6.79)	0.001	1.70 (0.80–3.59)	0.16
Nutrition status (*n*=988)	160	828				
Normal (reference)	137 (85.6%)	731 (88.3%)				
Malnourished	23 (14.4%)	97 (11.7%)	1.31 (0.79–2.19)	0.29		
Time to ART initiation (*n*=1052)	156	896				
Prompt initiation (reference)	19 (12.2%)	256 (28.6%)				
Delayed initiation	137 (87.8%)	640 (71.4%)	2.88 (1.75–4.76)	0.001	2.38 (1.23–4.62)	0.01
Referral sources to ART initiation (*n*=1094)	161	933				
HIV counselling and testing clinic (reference)	127 (78.9%)	633 (67.8%)				
Paediatric outpatient clinic	29 (18%)	207 (22.2%)	0.69 (0.45–1.08)	0.10	1.08 (0.65–1.81)	0.76
PMTCT clinic	1 (0.6%)	52 (5.6%)	0.10 (0.01–0.70)	0.02	0.28 (0.04–2.18)	0.23
Others	4 (2.5%)	41 (4.4%)				
ARV regimens at ART initiation (*n*=1092)	160	932				
AZT/3TC/NVP	30 (18.8%)	793 (85.1%)				
AZT/3TC/EFV	91 (56.9%)	52 (5.6%)				
AZT/3TC/ABC (triple nuke)	31 (19.4%)	10 (1.1%)				
D4T/3TC/NVP	2 (1.3%)	45 (4.8%)				
Others	6 (3.8%)	32 (3.4%)				

TB, tuberculosis; ART, antiretroviral therapy; OR, odds ratio; aOR, adjusted OR; CI, confidence interval; PMTCT, prevention of mother-to-child transfer; ARV, antiretroviral.

The main ART regimen for children with TB was AZT/3TC/EFV (56.9%) followed by AZT/3TC/ABC (19.4%) and AZT/3TC/NVP (18.8%). The cotrimoxazole prophylaxis was provided to 87.9% at ART initiation, including 95.7% (154/161) of those with TB. The median age at cotrimoxazole initiation was 4.7 years (IQR: 1.6–7.8) and 2.8 years (IQR: 1.3–6.4) for those with and without TB, respectively. Only 0.8% (7/933) of non-TB children were reported as receiving INH prophylaxis.

At the 24 month follow-up, 7.3% (68/933) of non-TB children developed TB, with 50 (73.5%) of these new TB cases occurring in the first six months. The rate of new TB cases was 3 per 100 person-years (95% CI: 2.2–4.0) at six months which declined to 0.9 (0.5–1.5) and 0.2 (0.06–1.4) per 100 person-years at 12 and 24 months, respectively.

### Retention, LFU and deaths among TB co-infected and non-infected

The differences in retention, LFU and deaths at the 6, 12 and 24 month follow-up periods among children on ART with and without TB is shown in Table 2. Both mortality (*p<*0.01) and LFU (*p<*0.05) were higher among children on ART who had been diagnosed with TB compared to non-TB children. The rates of LFU and death at 24 months among TB co-infected children were 12.8 (95% CI: 8.8–18.6) and 8 (5.1–12.8) compared to 8.7 (7.3–10.6) and 2.3 (1.6–3.2) per 100 person-years for non-TB children.

**Table 2:** Outcomes among children on ART by TB status

	Children with TB	Non-TB children	OR (95% CI)	*p*
6 months after ART initiation	161	933		
Retained (reference)	123 (76.4%)	844 (90.5%)		
Died	18 (11.2%)	22 (2.4%)	5.6 (2.9–10.6)	0.00
Lost to follow-up	20 (12.4%)	67 (7.2%)	2.1 (1.2–3.5)	0.01
12 months after ART initiation^a^	160	932		
Retained (reference)	114 (71.3%)	803 (86.1%)		
Died	19 (11.9%)	24 (2.6%)	5.2 (2.7–9.9)	0.00
Lost to follow-up	27 (16.9%)	105 (11.3%)	1.8 (1.1–2.9)	0.01
24 months after ART initiation^a^	129	805		
Retained (reference)	78 (60.5%)	615 (76.4%)		
Died	18 (14.0%)	35 (4.3%)	4.2 (2.3–7.8)	0.00
Lost to follow-up	33 (25.5%)	155 (19.3%)	1.7 (1.1–2.6)	0.03

TB, tuberculosis; ART, antiretroviral therapy; OR, odds ratio.

a Data analysis excludes participants who transferred out.

Following the Kaplan–Meier survival and Nelson–Aalen cumulative hazard analyses, children on ART who had TB were less likely to survive (*p=*0.001) and at increased risk to being LFU compared to those without TB (Figures 1 and 2). In a multivariate Cox proportional hazards model (Table 3), children with TB (aHR: 4.3; 95% CI: 2.3–7.9), malnutrition (aHR: 5.1; 95% CI: 2.6–9.8), those who delayed ART initiation (aHR: 3.2; 95% CI: 1.5–6.7) and those who were less than one-year old at ART initiation (aHR: 4.0; 95% CI: 1.4–12.0) were more likely to die. Additionally, children less than one year of age at ART initiation (aHR: 2.9; 95% CI: 1.7–5.2) and those with TB (aHR: 1.3; 95% CI: 1.1–1.6) were more likely to be LFU.

**Figure 1:** Kaplan–Meier survival by tuberculosis status among children on antiretroviral therapy.

**Figure 2:** Nelson–Aalen loss to follow-up by tuberculosis status.

**Table 3:** Factors associated with mortality and loss to follow-up

	Mortality				Loss to follow-up			
		
	HR (95% CI)	*p*	aHR (95% CI)	*p*	HR (95% CI)	*p*	aHR (95% CI)	*p*
Age at ART initiation								
<1 year	6.6 (2.4–17.7)	0.001	6.4 (1.9–12.0)	0.002	2.8 (1.6–4.9)	0.0001	3.2 (1.6–6.3)	0.001
2 to <5 years	2.9 (1.1–7.9)	0.03	2.8 (1.0–7.5)	0.04	1.8 (1.07–2.9)	0.025	1.8 (1.0–3.3)	0.05
5 to 9 years (reference)								
10 to 14 years	2.5 (0.8–8.3)	0.13 (NS)	1.8 (0.5–6.8)	0.37	1.7 (0.9–3.3)	0.098	1.5 (0.7–3.4)	0.31
Sex								
Female (reference)								
Male	1.4 (0.8–2.3)	0.26 (NS)			1.0 (0.8–1.4)	0.82		
Facility ownership								
Public/government (reference)								
Private not for profit	0.6 (0.3–1.6)	0.33 (NS)			0.9 (0.9–1.5)	0.85		
Private for profit	1.8 (0.2–12.8)							
CD4 at ART initiation								
Severe immunosuppression by age	1.3 (0.5–3.2)	0.58 (NS)			1.1 (0.7–1.8)	0.72		
Time to ART initiation								
Prompt initiation (reference)								
Delayed initiation	5.1 (2.1–9.1)	0.026	3.2 (1.5–6.7)	0.002	6.4 (4.6–8.8)	0.007	1.7 (0.4–1.3)	0.39
TB	3.4 (1.9–6.1)	0.0001	6.4 (3.3–12.4)	0.001	1.3 (1.06–1.5)	0.009	2.5 (1.5–4.2)	0.001
Nutrition status								
Normal (reference)								
Malnourished	6.8 (6.8–12.0)	0.0001	5.1 (2.6–9.8)	0.001	1.6 (0.9–2.6)	0.09		
Referral sources to ART initiation								
HIV counselling and testing clinic (reference)								
Paediatric outpatient clinic	1.3 (0.7–2.5)	0.32 (NS)			1.7 (1.2–2.4)	0.002	1.3 (0.7–2.2)	0.39
PMTCT clinic	0.4 (0.1–2.7)	0.33 (NS)			0.9 (0.4–2.0)	0.87 (NS)	0.6 (0.4–3.9)	0.84

ART, antiretroviral therapy; HR, hazard ratio; aHR, adjusted HR; CI, confidence interval; OR, odds ratio; TB, tuberculosis; PMTCT, prevention of mother-to-child transmission.

## Discussion

Few studies have reported the burden and outcomes of children on ART who had TB, and this analysis provides critical evidence that children on ART with TB are more likely to die and be lost to follow up than their non-TB counterparts. This result is consistent with other findings [13,20]. The study further reaffirms published literature of the association of mortality during ART with younger age at ART initiation and malnutrition [17,21].

The high TB screening rate among children on ART in this study is supported by previous studies in Nigeria [15]. The finding that TB increased with age is likely due to late initiation of ART depicted in this study and is similar with other findings in the Africa region [22,23]. This finding may reflect the difficulties in diagnosing TB in younger age groups due to limitations in the utility and interpretation of widely used diagnostic approaches such as chest radiography, sputum microbiology and tuberculin test in HIV/TB co-infected children [24–26]. In contrast, our study did not find significant differences in severe immunosuppression between TB-co-infected and non-infected children. The association between low CD4 count and TB among adult ART patients has also shown mixed results, with some studies showing stronger association and others weaker [22,27].

Higher incident TB infections, particularly within the first six months after ART initiation, is consistent with findings among adult populations where high probability of TB infection in the first three months of ART initiation was discussed [22,27]. Significant differences have been reported between TB before and after ART initiation with the probability of having TB within 30 months for pre-ART individuals as high as 22% compared to 18% among the ART population [27]. The reduction of TB risk following ART has been confirmed in most settings [28] and underscores the need for increased ART coverage and early ART initiation.

The choice of which ARV regimen to use among children and its challenges, particularly in TB/HIV co-infected children, has been amply discussed [7]. Over 18% of children with TB in this study were initiated on a nevirapine-based regimen, which has been found to interact with the TB agent rifampicin [6]. Though anti-TB regimens used were not examined, the Nigeria national guidelines recommend a rifampicin-based regimen as the first line for paediatric TB. Data on switching regimens or adverse reactions to determine any negative consequences for their co-administration were beyond the scope of this paper. These results will likely inform the selection and the optimal list of ARV formulations for paediatric TB/HIV co-morbidity.

The finding of high uptake of cotrimoxazole among children on ART in Nigeria is similar with other research [15], reflecting a concordance to the WHO guidelines and Stop TB policy on TB/HIV collaboration [29]. The significant gap was in the uptake of INH prophylaxis. This corresponds to other African countries [2] and needs urgent attention as improved outcomes have been documented following INH prophylaxis among individuals on ART [30,31]. There are various studies that have proposed measures for improving uptake of and adherence to INH [32–37]. Some of these strategies can be adapted in the Nigerian setting and will likely have a wider impact if implemented in lower-level facilities. The impact of decentralization on the scale-up of services including paediatric ART services has already be ascertained [38] and could well be adapted in Nigeria to increase the uptake of INH prophylaxis.

In 2014, UNAIDS and the international community endorsed new global targets (90–90–90) that seek to ensure that 90% of children living with HIV know their HIV status, 90% of children infected with HIV are receiving ART and 90% of those on treatment are virally suppressed [39]. From the findings of this study, these targets will not be actualized if TB among children living with HIV continues to be excluded from global priorities. Higher rates of mortality and LFU among TB co-infected children, as identified by this study, are detrimental to the progress towards these targets. Other studies have also established an independent association between TB and virological failure, particularly in children co-treated with protease inhibitors [40].

TB and delayed ART initiation have been previously found to be associated with poorer virological response and increased mortality [40–42], supporting the relationship between TB, delayed ART initiation and higher mortality observed in this study. Fewer infants initiated ART in this study, and fewer children were referred from prevention of mother-to-child transfer, which may explain the delay in ART initiation. This highlights the need for improved linkages between entry points for HIV testing and paediatric ART, as well as the adaptation of recent WHO ART guidelines that recommend HIV treatment for all children under the age of 5 and higher CD4 eligibility [43] in order to promote early initiation of ART.

Malnutrition was also identified as a factor associated with mortality. Other studies have established the association between food insecurity and incomplete viral suppression among children on ART [44], emphasizing the importance of improving nutrition among children on ART for survival and progress towards the 90–90–90 targets. Viral load testing, though not a major part of national recommendation at the time of this study, is critical as it is one of the major requirements for tracking progress towards the 90–90–90 targets. The data further underscore the need for improved retention and additional focus on younger age groups with the highest mortality.

The results of this study are limited by several factors. Random selection of facilities was not done in order to ensure that a wide and diverse representation of facilities were included. Data gaps due to incomplete medical records and poor linkage between ART and TB registers were common, though efforts were made to fill in data gaps from alternative sources of data in the facilities, such as clinical charts or laboratory registers. Due to diagnostic challenges, TB diagnosis was likely underestimated in some facilities. Despite these limitations, the findings have been consistent with other reports and have provided preliminary proof of high burden and poorer outcomes for children on ART with TB compared to non-TB children on ART, which may hamper progress towards the 90–90–90 targets.

## Conclusions

TB among children on ART presents a major challenge to achieving the global targets of 90–90–90 requiring global attention. Despite diagnostic challenges, Nigeria has demonstrated that childhood TB screening is effective in identifying prevalent and incident TB cases at different intervals of HIV treatment. However, uptake of INH prophylaxis continues to remain low. Higher levels of mortality, higher rates of LFU and delayed initiation of ART among children with TB need to be addressed to assist progress towards the 90–90–90 targets. These findings underscore the urgent need for inclusion of TB/HIV co-morbidity among children in global plans and reporting mechanisms.

## References

[1] Graham SM, Sismanidis C, Menzies HJ, Marais BJ, Detjen AK, Black RE (2014). Importance of tuberculosis control to address child survival. Lancet.

[2] World Health Organization (2014). Global tuberculosis report 2014.

[3] Pearce EC, Woodward JF, Nyandiko WM, Vreeman RC, Ayaya SO (2012). A systematic review of clinical diagnostic systems used in the diagnosis of tuberculosis in children. AIDS Res Treat.

[4] Pefura Yone EW, Evouna Mbarga A, Kuaban C (2012). The impact of HIV infection on childhood tuberculosis in Yaounde, Cameroon. Rev Mal Respir.

[5] Chamla D, Mbori-Ngacha D, Newman M, Kellerman S, Sugandhi N, Rwebembera A (2013). Evidence from the field: missed opportunities for identifying and linking HIV-infected children for early initiation of ART. AIDS.

[6] Ribera E, Pou L, Lopez RM, Crespo M, Falco V, Ocana I (2001). Pharmacokinetic interaction between nevirapine and rifampicin in HIV-infected patients with tuberculosis. J Acquir Immune Defic Syndr.

[7] Ren Y, Nuttall JJ, Egbers C, Eley BS, Meyers TM, Smith PJ (2008). Effect of rifampicin on lopinavir pharmacokinetics in HIV-infected children with tuberculosis. J Acquir Immune Defic Syndr.

[8] Marais BJ, Graham SM (2014). Childhood tuberculosis: a roadmap towards zero deaths. J Paediatr Child Health.

[9] United Nations Children's Fund (2013). Double dividend.

[10] World Health Organization (2014). Global strategy and targets for tuberculosis prevention, care and control after 2015.

[11] Pennap G, Makpa S, Ogbu S (2010). Sero-prevalence of HIV infection among tuberculosis patients in a rural tuberculosis referral clinic in northern Nigeria. Pan Afr Med J.

[12] Ige OM, Akindele MO (2011). Five year review of treatment outcome of directly observed therapy (DOT) for re-treatment pulmonary tuberculosis patients in UCH, Ibadan, Nigeria. Afr J Med Med Sci.

[13] Ifebunandu NA, Ukwaja KN, Obi SN (2012). Treatment outcome of HIV-associated tuberculosis in a resource-poor setting. Trop Doct.

[14] United Nations AIDS Programme (2013). 2013 progress report on the global plan: towards the elimination of new HIV infection among children by 2015 and keeping their mothers alive.

[15] Ojikutu B, Higgins-Biddle M, Greeson D, Phelps BR, Amzel A, Okechukwu E (2014). The association between quality of HIV care, loss to follow-up and mortality in pediatric and adolescent patients receiving antiretroviral therapy in Nigeria. PLoS One.

[16] World Health Organization (2010). Antiretroviral therapy of HIV infection in infants and children: towards universal access: recommendations for a public health approach – 2010 revision.

[17] McNairy ML, Lamb MR, Carter RJ, Fayorsey R, Tene G, Mutabazi V (2013). Retention of HIV-infected children on antiretroviral treatment in HIV care and treatment programs in Kenya, Mozambique, Rwanda and Tanzania. J Acquir Immune Defic Syndr.

[18] Kuczmarski RJ, Ogden CL, Grummer-Strawn LM, Flegal KM, Guo SS, Wei R (2000). CDC growth charts: United States. Adv Data.

[19] Kim MH, Cox C, Dave A, Draper HR, Kabue M, Schutze GE (2012). Prompt initiation of ART With therapeutic food is associated with improved outcomes in HIV-infected Malawian children with malnutrition. J Acquir Immune Defic Syndr.

[20] Buck WC, Olson D, Kabue MM, Ahmed S, Nchama LK, Munthali A (2013). Risk factors for mortality in Malawian children with human immunodeficiency virus and tuberculosis co-infection. Int J Tuberc Lung Dis.

[21] Kabue MM, Buck WC, Wanless SR, Cox CM, McCollum ED, Caviness AC (2012). Mortality and clinical outcomes in HIV-infected children on antiretroviral therapy in Malawi, Lesotho, and Swaziland. Pediatrics.

[22] Lawn SD, Myer L, Bekker LG, Wood R (2006). Burden of tuberculosis in an antiretroviral treatment programme in sub-Saharan Africa: impact on treatment outcomes and implications for tuberculosis control. AIDS.

[23] Okechukwu AA, Okechukwu OI (2011). Clinical correlate of tuberculosis in HIV co-infected children at the University of Abuja Teaching Hospital, Gwagwalada, Nigeria. Niger J Clin Pract.

[24] Marais BJ, Graham SM, Cotton MF, Beyers N (2007). Diagnostic and management challenges for childhood tuberculosis in the era of HIV. J Infect Dis.

[25] Graham SM (2014). Chest radiography for diagnosis of tuberculosis in children: a problem of interpretation. Int J Tuberc Lung Dis.

[26] Graham SM (2011). The use of diagnostic systems for tuberculosis in children. Indian J Pediatr.

[27] Alvarez-Uria G, Pakam R, Midde M, Naik PK (2014). Incidence and mortality of tuberculosis before and after initiation of antiretroviral therapy: an HIV cohort study in India. J Int AIDS Soc.

[28] Abuogi LL, Mwachari C, Leslie HH, Shade SB, Otieno J, Yienya N (2013). Impact of expanded antiretroviral use on incidence and prevalence of tuberculosis in children with HIV in Kenya. Int J Tuberc Lung Dis.

[29] World Health Organization (2012). WHO policy on collaborative TB/HIV activities: guidelines for national programmes and other stakeholders.

[30] Mosimaneotsile B, Mathoma A, Chengeta B, Nyirenda S, Agizew TB, Tedla Z (2010). Isoniazid tuberculosis preventive therapy in HIV-infected adults accessing antiretroviral therapy: a Botswana Experience, 2004–2006. J Acquir Immune Defic Syndr.

[31] Suthar AB, Lawn SD, del Amo J, Getahun H, Dye C, Sculier D (2012). Antiretroviral therapy for prevention of tuberculosis in adults with HIV: a systematic review and meta-analysis. PLoS Med.

[32] van Wyk SS, Reid AJ, Mandalakas AM, Enarson DA, Beyers N, Morrison J (2011). Operational challenges in managing Isoniazid Preventive Therapy in child contacts: a high-burden setting perspective. BMC Public Health.

[33] Shivaramakrishna HR, Frederick A, Shazia A, Murali L, Satyanarayana S, Nair SA (2014). Isoniazid preventive treatment in children in two districts of South India: does practice follow policy?. Int J Tuberc Lung Dis.

[34] Adams LV, Talbot EA, Odato K, Blunt H, Steingart KR (2014). Interventions to improve delivery of isoniazid preventive therapy: an overview of systematic reviews. BMC Infect Dis.

[35] Lewin S, Munabi-Babigumira S, Glenton C, Daniels K, Bosch-Capblanch X, van Wyk BE (2010). Lay health workers in primary and community health care for maternal and child health and the management of infectious diseases. Cochrane Database Syst Rev.

[36] M'Imunya JM, Kredo T, Volmink J (2012). Patient education and counselling for promoting adherence to treatment for tuberculosis. Cochrane Database Syst Rev.

[37] Lutge EE, Wiysonge CS, Knight SE, Volmink J (2012). Material incentives and enablers in the management of tuberculosis. Cochrane Database Syst Rev.

[38] Fayorsey RN, Saito S, Carter RJ, Gusmao E, Frederix K, Koech-Keter E (2013). Decentralization of pediatric HIV care and treatment in five sub-Saharan African countries. J Acquir Immune Defic Syndr.

[39] United Nations AIDS Programme (2014). 90–90–90: an ambitious treatment target to help end the AIDS epidemic.

[40] Zanoni BC, Phungula T, Zanoni HM, France H, Feeney ME (2011). Impact of tuberculosis cotreatment on viral suppression rates among HIV-positive children initiating HAART. AIDS.

[41] Yotebieng M, Van Rie A, Moultrie H, Cole SR, Adimora A, Behets F (2010). Effect on mortality and virological response of delaying antiretroviral therapy initiation in children receiving tuberculosis treatment. AIDS.

[42] Manosuthi W, Chottanapand S, Thongyen S, Chaovavanich A, Sungkanuparph S (2006). Survival rate and risk factors of mortality among HIV/tuberculosis-coinfected patients with and without antiretroviral therapy. J Acquir Immune Defic Syndr.

[43] World Health Organization (2013). Consolidated guidelines on the use of antiretroviral drugs for treating and preventing HIV infections.

[44] Mendoza JA, Paul ME, Schwarzwald H, Liu Y, Martinez R, Nicklas TA (2013). Food insecurity, CD4 counts, and incomplete viral suppression among HIV+ patients from Texas Children's Hospital: a pilot study. AIDS Behav.

# Sub article: The physical and psychological effects of HIV infection and its treatment on perinatally HIV-infected children

**DOI:** 10.7448/IAS.18.7.20258

**Published:** 2015-12-02

**Authors:** Rachel C Vreeman, Michael L Scanlon, Megan S McHenry, Winstone M Nyandiko

**Affiliations:** 1 Children's Health Services Research, Department of Pediatrics, Indiana University School of Medicine, Indianapolis, IN, USA; 2 Academic Model Providing Access to Healthcare (AMPATH), Eldoret, Kenya; 3 Department of Child Health and Paediatrics, School of Medicine, College of Health Sciences, Moi University, Eldoret, Kenya

**Keywords:** children, adolescents, perinatal HIV infection, development, HIV comorbidities, psychological complications

## Abstract

**Introduction:**

As highly active antiretroviral therapy (HAART) transforms human immunodeficiency virus (HIV) into a manageable chronic disease, new challenges are emerging in treating children born with HIV, including a number of risks to their physical and psychological health due to HIV infection and its lifelong treatment.

**Methods:**

We conducted a literature review to evaluate the evidence on the physical and psychological effects of perinatal HIV (PHIV+) infection and its treatment in the era of HAART, including major chronic comorbidities.

**Results and discussion:**

Perinatally infected children face concerning levels of treatment failure and drug resistance, which may hamper their long-term treatment and result in more significant comorbidities. Physical complications from PHIV+ infection and treatment potentially affect all major organ systems. Although treatment with antiretroviral (ARV) therapy has reduced incidence of severe neurocognitive diseases like HIV encephalopathy, perinatally infected children may experience less severe neurocognitive complications related to HIV disease and ARV neurotoxicity. Major metabolic complications include dyslipidaemia and insulin resistance, complications that are associated with both HIV infection and several ARV agents and may significantly affect cardiovascular disease risk with age. Bone abnormalities, particularly amongst children treated with tenofovir, are a concern for perinatally infected children who may be at higher risk for bone fractures and osteoporosis. In many studies, rates of anaemia are significantly higher for HIV-infected children. Renal failure is a significant complication and cause of death amongst perinatally infected children, while new data on sexual and reproductive health suggest that sexually transmitted infections and birth complications may be additional concerns for perinatally infected children in adolescence. Finally, perinatally infected children may face psychological challenges, including higher rates of mental health and behavioural disorders. Existing studies have significant methodological limitations, including small sample sizes, inappropriate control groups and heterogeneous definitions, to name a few.

**Conclusions:**

Success in treating perinatally HIV-infected children and better understanding of the physical and psychological implications of lifelong HIV infection require that we address a new set of challenges for children. A better understanding of these challenges will guide care providers, researchers and policymakers towards more effective HIV care management for perinatally infected children and their transition to adulthood.

## Introduction

An estimated 3.2 million children currently live with human immunodeficiency virus (HIV) [1]. Children with perinatal HIV infection (PHIV+) in the era of highly active antiretroviral therapy (HAART) have significantly improved odds of survival compared to the pre-HAART era [2] and are living into adolescence and adulthood in unprecedented numbers [3,4]. In the United States, mortality amongst HIV-infected children declined from 7.2 per 100 child-years in 1994 to 0.6 per 100 child-years in 2006, a more than 90% reduction [5]. Similar achievements have been made in Europe [6]. In these resource-rich settings, PHIV+ live longer, and fewer die of opportunistic infections [7], but PHIV+ still face significantly higher odds of morbidity and mortality compared to their uninfected peers [8,9]. Overall, it is estimated that HIV-infected children have mortality rates 30 times higher than uninfected children of similar age in the United States [10]. Although deaths from opportunistic infections have decreased significantly, deaths from end-stage, acquired immunodeficiency syndrome (AIDS), sepsis and renal failure are now more common [10]. And as HIV infection transforms from a terminal illness to a chronic disease, new comorbidities emerge, including metabolic disorders and cardiovascular and kidney diseases [11]. These comorbidities represent significant challenges to the long-term treatment and survival of PHIV+.

More than 90% of the world's PHIV+ live in resource-limited settings (RLS) such as sub-Saharan Africa (SSA), where less than a quarter of infected children currently receive HAART [12] and HIV/AIDS is still a leading cause of childhood and adolescent mortality [13]. In 2014, UNAIDS and partner stakeholders released ambitious new targets for the scale up of HIV testing and treatment, known as “90-90-90.” The aim is that by 2020, 90% of HIV-infected individuals will know their HIV status, 90% of diagnosed HIV-infected individuals will receive HAART, and 90% of HIV-infected individuals on HAART will be virally suppressed [14]. In addition, the 2013 World Health Organization (WHO) Consolidated ARV Guidelines recommend HAART for all PHIV+ under five years of age regardless of clinical or immunological stage [15]. The push towards these targets means that hundreds of thousands of PHIV+ will initiate life-long HAART over the next decade.

Although the majority of these children will be living in RLS, the experiences of PHIV+ in resource-rich settings, who have been treated with HAART for many more years, offer an important window into the long-term impact of perinatal infection and lifelong treatment across all settings. The objective of this review is to summarize and evaluate current evidence on the physical and psychological effects of PHIV+ infection and its treatment in the HAART era, including major chronic comorbidities. Insights will be offered on persistent and emerging challenges in the field of PHIV+ and future directions for research. Although much of the literature in this field comes from resource-rich settings, we will attempt to give appropriate attention to issues affecting PHIV+ in SSA.

## Methods

We conducted an extensive, literature review on the physical and psychological effects of PHIV+ infection and its treatment in the HAART era, including major chronic comorbidities. Our methodology for this review was guided by Grant and Booth [16] and their definition of a “literature review.” We searched major scientific literature databases (e.g. PubMed, EMBASE and MEDLINE) between 1 February 2015 and 1 June 2015, and we used relevant keywords to guide searches for appropriate sources. In this review, we aim to present findings from major, landmark studies, as well as from meta-analyses, systematic reviews and expert commentaries, to evaluate the current state of knowledge and to identify research gaps on the physical and psychological effects of PHIV+ infection. Where appropriate, we include statistics such as odds ratios (OR), hazard ratios (HR) and confidence intervals (CI), amongst other statistics. To facilitate our objective, we organized the Results and Discussion section in the following way: first, we review the general virologic and treatment outcomes of perinatally infected children, then discuss physical effects using an organ systems perspective (birth and growth outcomes, neurological, cardiovascular, gastrointestinal, renal, haematological, metabolic, musculoskeletal and reproductive), and end with a review of psychological effects.

## Results and discussion

### Viral and treatment outcomes

Studies from North America and Europe suggest that PHIV+ have lower probability of achieving both viral suppression and long-term treatment success compared to adults [17,18]. Data from RLS are limited but suggest more similar outcomes for children and adults. A recent, systematic review compiled data from 30 studies on treatment failure amongst 3241 children<18 years of age on first-line HAART in RLS [19]. In seven studies using a definition of treatment failure as a viral load (VL) >400 copies/mL, a median of 36% of children (range 13 to 71%) had failed first-line HAART [19]. Slightly better outcomes were reported in a systematic review of 89 studies including 9794 adult patients from SSA, in which a median of 28% had failed after one year [20]. Authors of both systematic reviews cited major challenges in comparing data across studies due to heterogeneous study designs, definitions, methods and length of follow-up.

Inadequate viral suppression can lead to the development of drug resistance in adults [21–24] and in children [25,26]. Drug resistance amongst PHIV+, who need lifelong therapy beginning at birth, is particularly concerning for their long-term outcomes. The challenges of lack of viral suppression and developing drug resistance are magnified for PHIV+ in RLS, where they often have limited access to adequate viral monitoring and to second- and third-line HAART regimens. Aggregated data reveal concerning rates of ARV resistance amongst PHIV+. Sigaloff *et al*. [19] reviewed data from 30 studies including 2258 HIV-infected children who had failed their first-line HAART regimen and calculated that a pooled proportion of 90% of children had a resistance-associated mutation (95% CI 88 to 93%). In subgroup analyses of children who failed treatment less than one year after HAART initiation, 76% (95% CI 69 to 83%) had at least one resistance-associated mutation [19]. The authors did not investigate differences in treatment failure or drug resistance by route of infection (perinatal versus other), presumably because few studies reported this information.

PHIV+ may be at higher risk for treatment failure and drug resistance for several reasons. First, PHIV+ may be exposed to HAART *in utero* or during breastfeeding for (unsuccessful) prevention of mother-to-child transmission (PMTCT) [27]. In the United States, the widespread use of HAART for PMTCT has reduced mother-to-child transmission to around 2% [28]. Despite its effectiveness, access, retention and adherence to PMTCT are not adequate and may be especially difficult in resource-poor settings [29,30]. Second, the risk of drug resistance is higher for many older PHIV+ with complicated treatment histories of monotherapy or dual therapy before the advent of HAART [31]. Third, studies show that significant proportions of PHIV+ have less than optimal therapeutic drug levels [32–35]. Subtherapeutic drug levels may be too low for viral suppression, yet sufficient to exert selection pressure that facilitates resistance mutation evolution [36–40]. Although non-adherence is a significant cause of subtherapeutic drug levels (see next section), even PHIV+ with high levels of adherence can fail treatment and develop drug resistance, particularly with longer treatment durations [41]. Conversely, supratherapeutic drug concentrations can also be problematic and can lead to drug toxicities, more severe side effects and non-adherence. Limited paediatric formulations, variable pharmacokinetics (PK) and dosing data, and frequent changes in dosing across the developmental course likely contribute to inadequate treatment of PHIV+, but more data are needed to address these challenges [42,43]. Finally, PHIV+ are a unique cohort; they acquire HIV before the maturation of their immune and organ systems and grow up with early and lifelong exposure to HAART [44].

Closely linked to the treatment outcomes for PHIV+ is the issue of medication adherence. Adherence to HAART is the critical behaviour underlying many of the long-term outcomes for children and adolescents with HIV. Systematic reviews of PHIV+ adherence to HAART have reported widely variable adherence estimates, likely due to heterogeneous measurement techniques [43,45]. Three studies (one in the United States and two in SSA) using electronic dose monitors, typically considered the gold standard measure [46–48], showed high rates of median adherence amongst PHIV+ (81, 95 and 96% of doses taken) [46,49,50]. Although high median rates of adherence are encouraging, they may not tell the whole story; patterns of adherence behaviour may critically shape outcomes. In the study from Kenya, only 68% of 191 PHIV+ achieved at least 90% adherence and they had a median of three treatment interruptions of greater than 48 hours over six months [50]. Another study in Kenya amongst a cohort of 21 PHIV+, established PK parameters for nevirapine (NVP) and used electronic dose monitors to calculate that almost half of the children spent more than 10% of time below the minimum effective drug concentration over four months of follow-up [51]. Although we know that non-adherence and clinical outcomes such as treatment failure are strongly associated [52,53], we lack data to characterize fully the relationship between adherence and drug resistance, particularly for PHIV+ in SSA. The risks of resistance resulting from non-adherence may vary based on regimen class, specific adherence patterns (e.g. treatment interruptions versus sporadic missed doses) and host genomics, in addition to the paediatric-specific factors just described [54]. Moreover, there are few data to inform strategies to improve and sustain high levels of adherence and prevent drug resistance in PHIV+ [43,55]. Studies do suggest that adherence and viral suppression decrease as PHIV+ transition into adolescence, highlighting the need for adherence interventions during this time [56–58].

It is in the context of these unique viral and treatment challenges that we must consider the impact of the physical and psychological effects of lifelong HIV infection, HAART treatment and comorbidities over the developmental course. Emerging data in these areas will be the focus of the remainder of this review.

### Birth outcomes

Fostering the growth of PHIV+ remains a major challenge throughout the developmental spectrum, from the prenatal to the adolescent phase. Although stillbirth, preterm birth and low birthweight have declined significantly in the era of PMTCT and HAART in resource-rich settings [59], several studies found increased risk of preterm delivery with maternal HAART use (versus mono or dual therapy for PMTCT), especially with protease inhibitor (PI)-containing regimens [60–64]. Data from SSA in this area are sparse [65]. A meta-analysis of studies conducted before 2007 found no overall link between HAART use during pregnancy and increased risk for preterm birth, but reported a significant association between PI-containing regimens and preterm birth versus non-PI containing regimens (OR 1.4, 95% CI 1.1 to 1.7), as well as maternal HAART started before pregnancy or in the first trimester versus later in pregnancy (OR 1.7, 95% CI 1.1 to 2.7) [66]. More recent results from a study amongst a large, well defined national cohort, the French Perinatal Cohort, found that ritonavir-boosted PI regimens were associated with an increased probability of preterm birth compared with non-boosted PI regimens (adjusted HR 2.0) when controlling for factors known to be associated with preterm delivery [67]. Preterm infants are at a significantly higher risk for a range of complications, including respiratory problems, infections, disability and mortality, and these risks may be compounded for PHIV+ [68]. More data are needed, particularly from SSA where the vast majority of HIV-positive women reside, health systems are less capable of caring for preterm infants, and where preterm delivery rates are the highest in the world [69].

### Growth and development

Throughout childhood, PHIV+ tend to have shorter stature, lower bodyweight and delayed entrance into puberty compared to uninfected children, even in the absence of overt AIDS or wasting [70]. This abnormal growth is associated with a wide set of factors, including viraemia, symptomatic HIV infection, malabsorption, inflammation, mitochondrial toxicity, psychosocial factors, micronutrient deficiency, abnormal nitrogen balance, and impaired growth hormone secretion or action [70]. A study using two large US longitudinal cohorts between 2000 and 2012 found that the timing of pubertal onset (Tanner stage≥2) was significantly later for 2086 PHIV+ compared to 453 HIV-exposed uninfected children [71]. Amongst PHIV+, the study also found that higher VL and lower CD4% were associated with more delayed pubertal onset, and that increased duration on HAART was associated with slightly more normal pubertal onset [71]. These data suggest that early access to HAART leads to more normal growth patterns for PHIV+, but there are few data from SSA where children are more likely to be malnourished and suffer from other diseases that are associated with poor growth [72].

### Neurological outcomes

The incidence of severe, AIDS-defining neurocognitive diseases, such as HIV encephalopathy, has significantly declined in the HAART era [73,74]. Amongst 2398 PHIV+ in the US-based Pediatric AIDS Clinical Trials Group (PACTG) 219/219C cohorts followed from 1993 to 2007, incidence of HIV encephalopathy decreased 10-fold beginning in 1996 (corresponding to the rollout of HAART), with stable incidence rates since 2002 at around two cases per 1000 person-years [75].

Nonetheless, PHIV+ on HAART may experience less severe neurocognitive complications, including deficits in general cognition, language and speech, gross motor, and fine motor functioning, which can substantially affect their quality of life, social relationships, school achievements and risk behaviours [76–81]. Several potential mechanisms for cognitive impairment in PHIV+ on HAART have been proposed, including (1) irreversible pre-HAART neuronal injury; (2) neuronal injury from inflammatory responses and neurotoxic viral proteins; (3) poor central nervous system (CNS) penetration of ARVs resulting in ongoing CNS viral replication and (4) neurotoxic effects of ARVs [82–86].

A number of studies reported that HIV-infected children treated with HAART had lower neurocognitive scores, high rates of motor deficits and delayed development compared to uninfected peers [87–95]; however, many of these studies were hampered by small sample sizes, lack of adequate control groups and lack of control for disease severity [86]. Several recent studies provided evidence for the protective effects of early HAART and viral suppression on neurodevelopment in PHIV+ [96,97]. A prospective study amongst 90 PHIV+ South African children randomized to receive early versus deferred HAART found significantly better neurodevelopment outcomes at 11 months of age as measured by the Griffiths Mental Development Scales in the early treatment group, who had a mean age of ART initiation at 8.4 weeks versus 31.4 weeks for the deferred treatment group [96]. Mental development scores from PHIV+ in the early treatment group were comparable with scores from HIV-uninfected (both exposed and unexposed) controls [96]. A study amongst 396 PHIV+ (mean age 9.6 years) in the United States found that early viral suppression (at five years of age or younger) was associated with higher cognitive scores on the Wechsler intelligence scales [97]. The study also found stronger associations in later birth cohorts when HAART use was more widespread, suggesting that HAART may improve neurocognitive outcomes independent of viral suppression [97]. In contrast, a study of 284 Thai and Cambodian PHIV+ aged 1 to 12 years in the PREDICT trial who were randomized to early or deferred HAART initiation reported no differences in neurodevelopmental scores between the two groups after 144 weeks, and found both early and deferred groups performed worse than uninfected controls [98,99]. However, the median age of HAART initiation was 6.4 years for the early group – significantly later than HAART initiation in the South Africa and US studies – which supports that neurological protective effects may be most significant in infancy and early childhood [98].

The potential effects of ARV-related neurotoxicity on neurodevelopment amongst PHIV+ are not clear and must be weighed against the benefits of CNS viral control. Cognitive impairment and decline in PHIV+ on HAART with good immunologic and systemic viral control [85] may point to a lack of CNS viral suppression due to poor drug CNS penetration, CNS-resistant virus, persistent immune activation or unknown factors [86]. Complicating this issue, ARVs that have high CNS penetration may better control HIV replication in cerebral spinal fluid that is associated with progressive HIV disease and neurocognitive deficits [100,101]. At the same time, ARVs with high CNS penetration may also increase the risks of ARV-related neurotoxicity. There are too few data amongst PHIV+ to assess the relationship between the level of CNS penetration by HAART agents and neurocognitive outcomes [74,75,97], and data amongst adults are conflicting [102–106].

A better understanding of the role of HIV infection and HAART on neurocognitive outcomes is needed to optimize treatment and to support the development of PHIV+ through adolescence and into adulthood. The field particularly lacks data from PHIV+ in SSA and data on how HIV infection and HAART exposure *in utero* affect neurocognitive development, as well as how these effects might be mitigated. Although studies advocate for the protective effects of HAART early in infancy [96,97], which is consistent with current WHO guidelines [15], whether these benefits will persist through adolescence and adulthood remains to be seen. More data from well-designed, longitudinal studies are warranted, in combination with measurement instruments adapted for children in SSA settings. This should be a high-priority research area as access to PMTCT and HAART expands, particularly in SSA.

### Cardiovascular system

The cardiovascular complications of HIV infection were noted early in the epidemic [107]. Current evidence suggests that PHIV+ may be at increased cardiac risk due to viral mechanisms [108], exposure to HAART for treatment or *in utero* for PMTCT or some combination [109]. Potential mechanisms of HIV-associated cardiomyopathy include direct infection of cardiac myocytes [107,110,111], increased production of certain cytokines within the myocardium [112–114] and nucleoside reverse transcriptase inhibitor (NRTI)-induced mitochondrial toxicity [115–117] that is most associated with zalcitabine, didanosine (ddI), stavudine (d4T) and zidovudine (AZT) [118–122]. The P2C2 study was the first major cohort study of PHIV+ to investigate cardiac complications and found high five-year cumulative incidence rates of depressed shortening fraction (28%), left ventricular end-diastolic dilation (22%), and heart failure or need for cardiac medications (29%); however, PHIV+ in this cohort had little exposure to modern HAART regimens [123,124].

More recent data suggest largely protective cardiac effects from HAART, especially early in life. In the largest prospective cohort study to date, 3035 PHIV+ enrolled in PACTG 219/219C were followed from 1993 to 2007 and showed that the use of HAART (versus no HAART) dramatically lowered incidence rates (by an average of 50%) of cardiomyopathy [125]. In subanalyses amongst HAART users, older age at HAART initiation, nadir CD4% below 15%, and initiating an AZT-containing regimen were independently associated with an increased risk of cardiomyopathy [125]. In another well-designed study, 70 PHIV+ from the earlier P2C2 study who had little exposure to HAART had significantly lower (i.e. worse) *z*-scores for left ventricular fractional shortening at around age 10 compared to 325 PHIV+ from the AMP cohort who had widespread use of HAART (*p*<0.05) [126]. Longer ARV exposure and lower nadir CD4% were associated with lower mean left ventricular fractional shortening *z*-scores in PHIV+ from the P2C2 cohort but not from the AMP cohort, suggesting that cardiac damage was more significant for PHIV+ early in life who did not receive effective HAART [126].

The effects of HAART exposure *in utero* are unclear, as two major studies amongst ART- exposed uninfected infants revealed conflicting results [127,128]. The CHAART-1 study found evidence that foetal exposure to HAART was associated with reduced left ventricular mass, left ventricular dimension and septal wall thickness [127], whereas the larger SMARTT study found low risk of overall cardiac anomalies and no specific increase in anomalies with any individual ARV [128]. Studies in SSA are lacking and were mostly conducted before widespread use of HAART [129–131]. The long-term effects of HAART exposure on PHIV+ in adolescence and young adulthood are also unknown. A small study of 28 PHIV+ with a mean age of 18.0 years who had been on HAART for a mean of 14.6 years found that although standard echocardiographic measures were normal, there was evidence of high impaired strain and strain rate (which have been proposed as prognostic factors for long-term myocardial dysfunction [132]) compared to age-matched, uninfected, unexposed controls [133]. The clinical impact of these changes over time is not known.

Although cardiovascular complications have significantly declined in the HAART era, PHIV+ still may be at higher risk for cardiomyopathies. Further research is needed on the long-term myocardial function of PHIV+ and on optimizing HAART regimens to protect cardiac health and to effectively suppress HIV replication. Close monitoring of cardiovascular function for this population is warranted, as is surveillance for HIV-uninfected children exposed to HAART *in utero* – a population that will increase significantly in size with more access to PMTCT under the 90-90-90 target.

### Gastrointestinal system

A prominent area of concern is the impact of HIV infection and its treatment on liver function for PHIV+, though there are few paediatric-specific data and long-term outcomes are mostly extrapolated from adult data [134]. PHIV+ tend to have HIV-associated elevated aspartate aminotransferase-to-platelet ratio indices, which may indicate liver fibrosis [135,136]. There are more data on the impact of HAART on liver function, which must be closely monitored in PHIV+. Almost all ARVs – in particular, atazanavir, ddI, indinavir and NVP – have the potential to cause hepatitis, hyperbilirubinaemia, liver toxicity and liver dysfunction [137]. In a small study of 26 PHIV+ on HAART aged 8 to 18 years, more than half had biological and/or radiological signs of hepatic impact, which was associated with older age, advanced disease stage and treatment with NRTI- and non-nucleoside analogue reverse transcriptase inhibitor (NNRTI)-containing regimens [138]. Regular monitoring of the liver function of PHIV+ is critical, which is now possible using various non-invasive procedures [138].

Co-infections can complicate treatment and increase the risks for liver damage, as is seen amongst HIV-infected adults with Hepatitis B virus (HBV) and Hepatitis C virus (HCV) co-infection [139]. There are comparatively few data on HBV and HCV co-infection amongst PHIV+, but a small number of studies suggest significant levels of chronic HBV and HCV co-infection [140–143]. Hepatitis co-infection requires specific treatment to reduce the risks of liver disease, hepatic fibrosis and cirrhosis in PHIV+ [144,145].

### Renal system

Renal disease is a growing concern for PHIV+ [146], and now accounts for 5% of mortality amongst PHIV+ in the HAART era [10]. The risk of HIV-associated nephropathy (HIVAN) has declined significantly with the rollout of HAART [147], but there are chronic renal complications predicted by renal abnormalities that may be caused by long-term HIV infection [148,149] and exacerbated by nephrotoxic ARVs [150]. This may be a particular concern with tenofovir disoproxil fumarate (TDF)-containing regimens in PHIV+, but study findings are not consistent [151–154]. The most common histopathological abnormality identified in renal biopsies in children with HIVAN is focal segmental glomerulosclerosis [155]. Host genomics play a significant role in the development of HIVAN, resulting in a fourfold increased risk for end-stage, kidney disease in African Americans in the United States [156,157]. High rates of renal disease amongst HIV-infected adults have been reported in SSA, along with evidence of a broader spectrum of histopathological lesions, but there are few data amongst PHIV+ [158]. A retrospective analysis of PHIV+ in the US-based PACTG 219/219C cohorts identified a variety of immune complex-mediated glomerulonephritides and HIVAN, with an incidence rate of chronic kidney disease of 2.79 events per 1000 person years in the 2003 to 2006 HAART era [159]. Other longitudinal cohort studies in the United States have found prevalence rates of nephrotic-range proteinuria between 8 and 11% amongst PHIV+ on HAART, with common risk factors of renal dysfunction including older age, uncontrolled viraemia, African–American race and use of TDF-containing regimens [160,161].

There are few data on survival outcomes amongst PHIV+ with end-stage kidney disease on maintenance dialysis in the HAART era. Two small studies amongst HIV-infected children with end-stage kidney disease on maintenance haemodialysis in the United States reported high mortality associated with cardiovascular disease, leading the authors to recommend routine echocardiography amongst HIV-infected children on dialysis [162,163]. Studies on kidney transplantation for HIV-infected individuals on HAART with end-stage kidney disease, once contraindicated in this population, show comparable three to five years survival rates to transplantation in uninfected individuals [164–166], but there are few data amongst children. More longitudinal data are needed to guide screening, prevention and treatment of renal diseases amongst an aging PHIV+ cohort, particularly as kidney diseases evolve from more acute conditions associated with advanced HIV disease to chronic diseases associated with long-term HIV infection and HAART exposure, as well as potential metabolic disorders. For PHIV+ in SSA and other RLS, interventions to prevent end-stage kidney disease are especially urgent as access to dialysis and transplantation are limited.

### Haematological issues

A frequent complication of HIV infection is anaemia. HIV infection may alter cytokine and erythropoietin responses by decreasing erythropoiesis through apoptosis of erythroid precursors and infection of auxiliary cells [167,168]. A number of common co-infections in PHIV+, such as neoplasms [169] and bacterial and fungal infections [170–172] including mycobacterial infections (e.g. *Mycobacterium tuberculosis*) [173,174], also increase risks for anaemia. In addition, children with HIV are at greater risk for more severe malaria-related anaemia compared to uninfected children [175,176]. Treatment with HAART, especially with AZT-containing regimens, is associated with increased risk for anaemia in children [177–179], including at birth for PHIV+ whose mothers were taking AZT for PMTCT [180,181]. A systematic review of 36 studies found that PHIV+ compared to uninfected children were at significantly higher odds of anaemia, with a calculated pooled random-effects OR of mild (haemoglobin <11 g/dL) and moderate (haemoglobin <9 g/dL) anaemia of 4.5 (95% CI 2.5 to 8.3) and 4.5 (95% CI 2.0 to 10.3), respectively, but there were few data on severe anaemia (haemoglobin <7 g/dL) [182]. Anaemia was commonly associated with HIV disease progression and mortality, and the use of HAART and treatment of secondary infections were protective, suggesting that as more PHIV+ access HAART and are able to switch regimens when AZT-associated anaemia is suspected, severe anaemia-related complications may be reduced in this population [182]. There is little evidence to support additional therapeutic interventions for anaemia amongst PHIV+, including interventions such as recombinant human erythropoietin and micronutrient supplementation, and more data are needed to evaluate these potential treatment options [183].

### Metabolic impact

PHIV+ on HAART are at an increased risk for metabolic disorders [184–186]. Lipodystrophy syndrome (or “fat redistribution”) comprises both lipoatrophy (loss of subcutaneous fat from the face, limbs and buttocks) and lipohypertrophy (increase of central fat) and can occur independent of weight change and dyslipidaemia [187]. HAART-associated lipoatrophy and lipohypertrophy have different and multifactorial pathogeneses that may involve changes in genetic polymorphisms, lipid metabolism, and adipocyte and mitochondrial cell function [188,189]. Studies reported a prevalence of lipodystrophy for PHIV+ on HAART between 10 and 33% [190–196], but rates as high as 57% have been found in Europe [197,198] and 65% in Thailand [199]. Common risk factors for lipodystrophy include longer duration on HAART, older age (especially during puberty), more severe HIV disease, and regimens containing NRTIs (particularly d4T and AZT) and to a lesser degree PIs, which may act synergistically when used together [200,201].

Body fat changes may ultimately affect adherence to therapy amongst older children and adolescents because they affect body image and the social desire to fit in is a strong motivator of behaviour during adolescent development [202]. In addition, characteristic body fat changes associated with HIV treatment may be recognizable and elicit HIV-related stigma, leading to reduced quality of life and adherence to medications, particularly in settings like SSA where HIV/AIDS stigma is pervasive [203–205]. Proper management of HAART-associated fat redistribution is critical in supporting the long-term treatment of PHIV+, particularly in SSA where fewer regimen options are combined with higher risks of stigma.

Dyslipidaemia, including hypertriglyceridaemia (elevated triglyceride levels) and hypercholesterolaemia (elevated LDL-cholesterol levels), is another potential metabolic complication for PHIV+ on HAART [206]. Elucidating potential mechanisms of dyslipidaemia in the HAART era have focused on the impact of PIs on the inhibition of low-density lipoprotein receptor-related proteins [207]. Data are limited to mostly small studies from the United States and Europe, where prevalence of hypertriglyceridaemia in PHIV+ on HAART ranges from 13 to 67% [186,190,192,208–211] and hypercholesterolaemia from 10 to 68% [186,190,192,208–212]. In the largest study to date amongst 2122 PHIV+ in the PACTG 219C cohort, the incidence rate for hypercholesterolaemia (total cholesterol ≥220 mg/dL at two consecutive time points) was 3.4 cases per 100 person-years (95% CI 3.0 to 3.9) [213]. The most significant factors associated with the development of hypercholesterolaemia after adjusting for age were the use of PI-containing regimens and lower VL [213]. In a follow-up study of the 240 PHIV+ who developed hypercholesterolaemia, only 34% experienced resolution to normal cholesterol levels after two years [214]. Smaller studies have shown potential benefits from switching from a PI-based to NNRTI-based regimen but more data are needed to guide optimal treatment in PHIV+ [215–218]. Furthermore, although the first-line treatment for dyslipidaemia in children is to change the diet and increase physical exercise, there are not data on the efficacy of these strategies amongst PHIV+ on HAART, and there are not clear guidelines on lipid thresholds for the use of statins and other lipid-lowering medications in this population [219]. As more PHIV+ initiate lifelong HAART, access to lipid monitoring needs to be supported to reduce the potential for drug toxicity and long-term lipid disorders.

Insulin resistance is characterized by reduced insulin stimulation of glucose use by adipose tissue and muscles resulting in increased pancreatic insulin production, and is associated with obesity, dyslipidaemia, hypertension and type 2 diabetes in children [220]. Both PIs and NRTIs are associated with insulin resistance through inhibition of GLUT-4 transporters in myocytes and adipocytes, causing decreased uptake of glucose by these tissues [221,222]. HAART-associated body composition changes such as central obesity, resulting in fat deposition in muscle cells, may also contribute to insulin resistance [223], but data are inconclusive [224,225]. The prevalence of insulin resistance in PHIV+ varies widely, ranging from 7 to 52% [190,208,209,211,226,227]. In 2011, a study amongst a large cohort of 402 PHIV+ in the United States found insulin resistance in 15% of the cohort, which was most strongly associated with obesity but also with low CD4% and exposure to PIs [228]. The prevalence of other glucose metabolism disorders such as abnormal fasting glucose and impaired glucose tolerance amongst PHIV+ is typically much lower (below 7%), but studies are few and generally small [186,190,208,209,211,227]. As in the case of dyslipidaemia, treatment for insulin resistance includes diet and exercise, and in the case of suspected PI-caused insulin resistance, studies suggest improved insulin sensitivity with switches from a PI- to NNRTI-based regimen in PHIV+ [229–231].

PHIV+ are at an increased risk for a variety of metabolic complications, but there are few longitudinal data to assess incidence, risk factors and long-term outcomes of these metabolic disorders [232]. Existing studies are further complicated by small sample sizes, heterogeneous definitions and complex interrelations in aetiology and risk factors. Metabolic disorders are significant risk factors for the acceleration of cardiovascular disease amongst HIV-infected adults [233], but whether these complications lead to the same cardiovascular risks in the ageing PHIV+ cohort remains to be seen [234]. As PHIV+ enter adolescence and young adulthood, optimizing treatment and reducing HIV-related and non-HIV-related risk factors for metabolic disorders are critical. Further data to guide prevention, monitoring and management of complications are urgently needed. PHIV+ in SSA may be at increased risk of poor diagnosis and management of complications due to lack of access to cardiac and metabolic monitoring tools.

### Musculoskeletal system

PHIV+ may be at greater risk for lower bone mineral density (BMD) due to viral infection – by affecting BMD-related growth factors [235,236] – and due to HAART exposure [237,238], particularly TDF [239]. Recent studies amongst PHIV+ revealed conflicting results. Two studies reported significant BMD loss amongst children treated with TDF-containing salvage regimens, with one-third losing more than 6% BMD [240,241]. It is unclear how these findings were confounded by the children's uncontrolled HIV infection, although one study reported that two children who discontinued TDF had significant recovery of BMD at 96 weeks [240]. Other studies found no association between TDF-containing regimens and greater risk for lower BMD [218,242,243]. In the longest study to date, 26 PHIV+ on TDF-containing regimens were followed for 132 months and showed good viraemic control and no significant increase in serum creatinine [244].

The effects of TDF may vary by developmental stage; studies have reported that TDF-associated BMD loss is less significant in adolescents and adults compared to PHIV+ in pre- or early pubertal stages [245,246]. Younger children's BMD may be more affected because their skeletal growth requires higher bone turnover; however, the aetiology, long-term risks and consequences of lower BMD for PHIV+ are not known [247]. More data are needed to guide optimal treatment regimens and to identify potential long-term complications of decreased BMD amongst PHIV+, who may consequently be at higher risk for bone fractures and osteoporosis later in life.

### Sexual and reproductive health

Studies show that having a sexually transmitted infection (STI) increases the risks for transmission and acquisition of HIV [248,249], but whether HIV-infected individuals on HAART with well-controlled HIV infection are at greater risk for STIs is less clear [250,251]. Several studies reported high rates of STIs amongst adolescents and young adults behaviourally infected with HIV [252–256], but there are fewer studies amongst PHIV+. A study of 638 PHIV+ adolescent women in the PACTG 219C cohort reported higher rates of condylomata acuminata, trichomoniasis and cervical abnormalities, including atypical cells, low-grade, squamous intraepithelial lesions and high-grade squamous intraepithelial lesions compared to matched, uninfected controls [257]. A cohort study of PHIV+ and behaviourally infected women in the United States found higher rates of pregnancy and premature births in both groups compared to the general population, with PHIV+ women significantly more likely to electively terminate the pregnancy compared to behaviourally infected women [258]. A recent retrospective cohort study of 152 pregnancies in the United States reported that uninfected infants born to PHIV+ mothers were significantly shorter throughout the first year of life (after adjusting for confounding) compared to uninfected infants born to non-perinatally HIV-infected mothers [259], but the significance of these findings is unclear.

There is limited evidence to inform sexual and reproductive interventions amongst PHIV+. A recent systematic review found evidence that linking sexual and reproductive services with HIV/AIDS services resulted in improved outcomes for women, including HIV and STI incidence, condom use, contraceptive use and retention in care [260]. Effective service delivery methods for sexual and reproductive health care, in combination with adolescent care transitions [168,261,262], must be investigated further as more PHIV+ enter adolescence and adulthood. In addition, the seldom-discussed issue of reproductive and sexual health for PHIV+ men will need to be addressed.

### Psychological health

A number of studies reported high rates of mental and behavioural disorders amongst PHIV+ [92,263–265]. Studies in the United States found higher than expected rates of anxiety, depression, hyperactivity, learning, other behavioural problems amongst PHIV+ [92,263], as well as rates of psychiatric hospitalizations three times higher compared to the general paediatric population, most commonly for depression and behaviour disorders [264]. A review of eight studies on the prevalence of psychiatric disorders amongst HIV-infected children and youth (aged 4 to 21 years) using the *Diagnostic and Statistical Manual* (Fourth Edition; DSM-IV) found high rates of attention deficit/hyperactivity disorders (29%), anxiety (24%) and depression (25%); however, the authors noted the lack of control groups and small sample sizes as significant limitations [265]. Several reviews have similarly pointed to the methodological weaknesses in existing studies on psychosocial outcomes amongst PHIV+ and the complex interactions of genetic, social and environmental factors [266,267]. Indeed, in larger, more well-designed trials with appropriate control groups, studies reported less evidence for increased mental and behavioural disorders amongst PHIV+ in childhood or adolescence [263,268–270]. In a US study, the authors found that PHIV+ were no more likely to have psychiatric symptoms than age-matched HIV-exposed uninfected controls, but were significantly more likely to be diagnosed and to have received treatment for a psychiatric disorder, including receiving psychotropic medication [268].

There is scarce evidence in the existing literature on the specific role of HIV or HAART in affecting psychological outcomes [267]. Several studies reported that low cognitive functioning scores were associated with mental health disorders amongst PHIV+ [92,98,271], but the significance of this finding is not clear. Clinical disease markers such as AIDS defining illness [272,273], higher VL [272,274] and low CD4 [272,275] were associated with mental and behavioural disorders amongst PHIV+ in some studies, but not in others [263,276,277]. There are also few data on psychological outcomes amongst PHIV+ in RLS [278–280]. High rates of orphanhood amongst PHIV+, particularly in SSA, may be an additional non-disease-related factor affecting mental and behavioural health [281–284]. Rigorous studies in settings such as SSA are hampered by a lack of rigorously evaluated and validated tools for assessing mental and behavioural disorders in paediatric populations [285]. As hundreds of thousands of PHIV+ transition into adolescence and adulthood, data are needed on the psychological challenges and targeted mental and behavioural interventions for PHIV+, which are critical to support their long-term chronic disease management [267].

## Conclusions

The advent of HAART drastically changed the course of HIV and significantly decreased HIV-associated morbidity and mortality. Although HAART allows PHIV+ to survive and prosper well into adolescence and adulthood, PHIV+ still face many challenges related to their perinatal infection and lifelong treatment. This review identified major chronic complications and comorbidities affecting all major organ systems. As more PHIV+ access HAART and live longer over the next decades, data that improve the management and ultimately prevention of these complications over the course of the developmental spectrum are critical. For PHIV+ with HIV-related disabilities, rehabilitation interventions are necessary to support their development with a chronic condition, but there are few data in this area [286,287]. The transition of PHIV+ from paediatric into adolescent and adult care settings is another under-researched area with significant implications for the treatment of long-term complications and comorbidities in PHIV+.

Many studies included in this review were conducted in the United States and Europe. PHIV+ in resource-rich settings represent an important population for study as they are mostly older, have been treated with HAART for many more years, and often initiated treatment at earlier ages compared to PHIV+ in RLS. For these reasons, PHIV+ in resource-rich settings offer critical insight into long-term complications and comorbidities and interventions to improve treatment and clinical outcomes. In addition, well-established cohorts such as the PACTG 219/219C in the United States provide opportunities to conduct studies with relatively large sample sizes and close monitoring and follow-up of patients. These cohorts have been particularly useful in investigating complex effects on different organ systems from HIV infection, HAART treatment or a combination of both. On the other hand, we did identify a number of recent studies from SSA, which is encouraging given that the region is home to over 90% of PHIV+ in the world. Studies across regions, both resource-rich and resource-poor, had significant limitations, most often issues of sample size, appropriate control groups, length of follow-up, heterogeneous definitions and assessment methods, and unknown and complex interrelations in aetiology and risk factors. Across all areas of research identified in this review, more research is needed that uses improved methodologies to address limitations. Establishing well-characterized and closely followed cohorts of PHIV+ in SSA and other RLS (as well as HIV-uninfected exposed children) will be essential to answer important questions around lifelong infection and treatment [288].

The combined evidence from this review supports the protective effects of early HAART for PHIV+ against many infection-related complications across organ systems; however, there are unanswered questions around HAART toxicity and management of potential drug side effects, as well as persisting infectious impacts. As the WHO guidelines advocate for the treatment of all PHIV+ with HAART and we work towards the goals of “90-90-90,” hundreds of thousands of PHIV+ will be initiating early and lifelong HAART in the coming years. HIV care programmes, particularly those in SSA, will be tasked with huge challenges in expanding HAART treatment and managing chronic complications and comorbidities of PHIV+. Furthermore, persistent challenges in areas such as infant diagnosis [289,290] and retention in care [291], to name just a few, are additional barriers to effectively treating PHIV+ in these settings. Finally, HIV cannot be viewed as a problem only for the realm of infectious disease; effective healthcare systems for the millions of youth needing HIV treatment will incorporate a holistic approach to the management of children and families’ physical, mental, emotional, behavioural and social needs across the developmental course.

## References

[1] World Health Organization (2014). Global update on the health sector response to HIV, 2014.

[2] Lee GM, Gortmaker SL, McIntosh K, Hughes MD, Oleske JM, Pediatric AIDS Clinical Trials Group Protocol 219C Team (2006). Quality of life for children and adolescents: impact of HIV infection and antiretroviral treatment. Pediatrics.

[3] Delaney M (2006). History of HAART – the true story of how effective multi-drug therapy was developed for treatment of HIV disease. Retrovirology.

[4] McConnell MS, Byers RH, Frederick T, Peters VB, Dominguez KL, Sukalac T (2005). Trends in antiretroviral therapy use and survival rates for a large cohort of HIV-infected children and adolescents in the United States, 1989–2001. J Acquir Immune Defic Syndr.

[5] Mofenson LM, Williams P, Brady MT, van Dyke RB, Dankner WM, Oleske JM (2008). Trends in mortality in HIV-infected children in the United States – 1994–2006.

[6] Judd A, Doerholt K, Tookey PA, Sharland M, Riordan A, Menson E (2007). Morbidity, mortality, and response to treatment by children in the United Kingdom and Ireland with perinatally acquired HIV infection during 1996–2006: planning for teenage and adult care. Clin Infect Dis.

[7] Gona P, Van Dyke RB, Williams PL, Dankner WM, Chernoff MC, Nachman SA (2006). Incidence of opportunistic and other infections in HIV-infected children in the HAART era. JAMA.

[8] Newell ML, Brahmbhatt H, Ghys PD (2004). Child mortality and HIV infection in Africa: a review. AIDS.

[9] Newell ML, Coovadia H, Cortina-Borja M, Rollins N, Gaillard P, Dabis F (2004). Mortality of infected and uninfected infants born to HIV-infected mothers in Africa: a pooled analysis. Lancet.

[10] Brady MT, Oleske JM, Williams PL, Elgie C, Mofenson LM, Dankner WM (2010). Declines in mortality rates and changes in causes of death in HIV-1-infected children during the HAART era. J Acquir Immune Defic Syndr.

[11] Hazra R, Siberry GK, Mofenson LM (2010). Growing up with HIV: children, adolescents, and young adults with perinatally acquired HIV infection. Ann Rev Med.

[12] World Health Organization (2011). Global HIV/AIDS response: epidemic update and health sector progress towards universal access – progress report 2011.

[13] Patton GC, Coffey C, Sawyer SM, Viner RM, Haller DM, Bose K (2009). Global patterns of mortality in young people: a systematic analysis of population health data. Lancet.

[14] UNAIDS (2014). 90-90-90 An ambitious treatment target to help end the AIDS epidemic.

[15] World Health Organization (2013). Consolidated guidelines on the use of antiretroviral drugs for treatment and preventing HIV infection.

[16] Grant MJ, Booth A (2009). A typology of reviews: an analysis of 14 review types and associated methodologies. Health Info Libr J.

[17] van Rossum AM, Fraaij PL, de Groot R (2002). Efficacy of highly active antiretroviral therapy in HIV-1 infected children. Lancet Infect Dis.

[18] Sabin CA, Smith CJ, d'Arminio Monforte A, Battegay M, Gabiano C, Galli L (2008). Response to combination antiretroviral therapy: variation by age. AIDS.

[19] Sigaloff KC, Calis JC, Geelen SP, van Vugt M, de Wit TF (2011). HIV-1-resistance-associated mutations after failure of first-line antiretroviral treatment among children in resource-poor regions: a systematic review. Lancet Infect Dis.

[20] Barth RE, van der Loeff MF, Schuurman R, Hoepelman AI, Wensing AM (2010). Virological follow-up of adult patients in antiretroviral treatment programmes in sub-Saharan Africa: a systematic review. Lancet Infect Dis.

[21] D'Aquila RT, Johnson VA, Welles SL, Japour AJ, Kuritzkes DR, DeGruttola V (1995). Zidovudine resistance and HIV-1 disease progression during antiretroviral therapy. AIDS Clinical Trials Group Protocol 116B/117 Team and the Virology Committee Resistance Working Group. Ann Intern Med.

[22] Lorenzi P, Opravil M, Hirschel B, Chave JP, Furrer HJ, Sax H (1999). Impact of drug resistance mutations on virologic response to salvage therapy. Swiss HIV Cohort Study. AIDS.

[23] Zolopa AR, Shafer RW, Warford A, Montoya JG, Hsu P, Katzenstein D (1999). HIV-1 genotypic resistance patterns predict response to saquinavir-ritonavir therapy in patients in whom previous protease inhibitor therapy had failed. Ann Intern Med.

[24] Tang MW, Shafer RW (2012). HIV-1 antiretroviral resistance: scientific principles and clinical applications. Drugs.

[25] Adje-Toure C, Hanson DL, Talla-Nzussouo N, Borget MY, Kouadio LY, Tossou O (2008). Virologic and immunologic response to antiretroviral therapy and predictors of HIV type 1 drug resistance in children receiving treatment in Abidjan, Cote d'Ivoire. AIDS Res Hum Retrovir.

[26] Rojas Sanchez P, Holguin A (2014). Drug resistance in the HIV-1-infected paediatric population worldwide: a systematic review. J Antimicrob Chemother.

[27] Moorthy A, Gupta A, Bhosale R, Tripathy S, Sastry J, Kulkarni S (2009). Nevirapine resistance and breast-milk HIV transmission: effects of single and extended-dose nevirapine prophylaxis in subtype C HIV-infected infants. PLoS One.

[28] Whitmore SK, Taylor AW, Espinoza L, Shouse RL, Lampe MA, Nesheim S (2012). Correlates of mother-to-child transmission of HIV in the United States and Puerto Rico. Pediatrics.

[29] Ferguson L, Grant AD, Watson-Jones D, Kahawita T, Ong'ech JO, Ross DA (2012). Linking women who test HIV-positive in pregnancy-related services to long-term HIV care and treatment services: a systematic review. Trop Med Int Health.

[30] Nachega JB, Uthman OA, Anderson J, Peltzer K, Wampold S, Cotton MF (2012). Adherence to antiretroviral therapy during and after pregnancy in low-income, middle-income, and high-income countries: a systematic review and meta-analysis. AIDS.

[31] de Mulder M, Yebra G, Navas A, de Jose MI, Gurbindo MD, Gonzalez-Tome MI (2012). High drug resistance prevalence among vertically HIV-infected patients transferred from pediatric care to adult units in Spain. PLoS One.

[32] Fraaij PL, van Kampen JJ, Burger DM, de Groot R (2005). Pharmacokinetics of antiretroviral therapy in HIV-1-infected children. Clin Pharmacokinet.

[33] Fraaij PL, Rakhmanina N, Burger DM, de Groot R (2004). Therapeutic drug monitoring in children with HIV/AIDS. Ther Drug Monit.

[34] Ren Y, Nuttall JJ, Egbers C, Eley BS, Meyers TM, Smith PJ (2007). High prevalence of subtherapeutic plasma concentrations of efavirenz in children. J Acquir Immune Defic Syndr.

[35] Viljoen M, Gous H, Kruger HS, Riddick A, Meyers TM, Rheeders M (2010). Efavirenz plasma concentrations at 1, 3, and 6 months post-antiretroviral therapy initiation in HIV type 1-infected South African children. AIDS Res Hum Retrovir.

[36] Veldkamp AI, Weverling GJ, Lange JM, Montaner JS, Reiss P, Cooper DA (2001). High exposure to nevirapine in plasma is associated with an improved virological response in HIV-1-infected individuals. AIDS.

[37] de Vries-Sluijs TE, Dieleman JP, Arts D, Huitema AD, Beijnen JH, Schutten M (2003). Low nevirapine plasma concentrations predict virological failure in an unselected HIV-1-infected population. Clin Pharmacokinet.

[38] Marzolini C, Telenti A, Decosterd LA, Greub G, Biollaz J, Buclin T (2001). Efavirenz plasma levels can predict treatment failure and central nervous system side effects in HIV-1-infected patients. AIDS.

[39] Csajka C, Marzolini C, Fattinger K, Decosterd LA, Fellay J, Telenti A (2003). Population pharmacokinetics and effects of efavirenz in patients with human immunodeficiency virus infection. Clin Pharmacol Ther.

[40] Stahle L, Moberg L, Svensson JO, Sonnerborg A (2004). Efavirenz plasma concentrations in HIV-infected patients: inter- and intraindividual variability and clinical effects. Ther Drug Monit.

[41] Mullen J, Leech S, O'Shea S, Chrystie IL, Du Mont G, Ball C (2002). Antiretroviral drug resistance among HIV-1 infected children failing treatment. J Med Virol.

[42] Menson EN, Walker AS, Sharland M, Wells C, Tudor-Williams G, Riordan FA (2006). Underdosing of antiretrovirals in UK and Irish children with HIV as an example of problems in prescribing medicines to children, 1997–2005: cohort study. BMJ.

[43] Simoni JM, Montgomery A, Martin E, New M, Demas PA, Rana S (2007). Adherence to antiretroviral therapy for pediatric HIV infection: a qualitative systematic review with recommendations for research and clinical management. Pediatrics.

[44] Abuzaitoun OR, Hanson IC (2000). Organ-specific manifestations of HIV disease in children. Pediatr Clin North Am.

[45] Vreeman RC, Wiehe SE, Pearce EC, Nyandiko WM (2008). A systematic review of pediatric adherence to antiretroviral therapy in low- and middle-income countries. Pediatr Infect Dis J.

[46] Farley J, Hines S, Musk A, Ferrus S, Tepper V (2003). Assessment of adherence to antiviral therapy in HIV-infected children using the Medication Event Monitoring System, pharmacy refill, provider assessment, caregiver self-report, and appointment keeping. J Acquir Immune Defic Syndr.

[47] Liu H, Golin CE, Miller LG, Hays RD, Beck CK, Sanandaji S (2001). A comparison study of multiple measures of adherence to HIV protease inhibitors. Ann Intern Med.

[48] Deschamps AE, De Geest S, Vandamme AM, Bobbaers H, Peetermans WE, Van Wijngaerden E (2008). Diagnostic value of different adherence measures using electronic monitoring and virologic failure as reference standards. AIDS Patient Care STDs.

[49] Haberer JE, Cook A, Walker AS, Ngambi M, Ferrier A, Mulenga V (2011). Excellent adherence to antiretrovirals in HIV+ Zambian children is compromised by disrupted routine, HIV nondisclosure, and paradoxical income effects. PLoS One.

[50] Vreeman RC, Nyandiko WM, Liu H, Tu W, Scanlon ML, Slaven JE (2014). Measuring adherence to antiretroviral therapy in children and adolescents in western Kenya. J Int AIDS Soc.

[51] Vreeman RC, Nyandiko WM, Liechty EA, Busakhala N, Bartelink IH, Savic RM (2014). Impact of adherence and anthropometric characteristics on nevirapine pharmacokinetics and exposure among HIV-infected Kenyan children. J Acquir Immune Defic Syndr.

[52] Vignoles M, Barboni G, Agosti MR, Garcia M, Ferrari I, Mahler E (2008). High frequency of antiretroviral drug resistance and low percentage of drug detection in plasma in HIV-infected children with low virological failure AIDS 2008.

[53] Gody JC, Charpentier C, Mbitikon O, Si-Mohamed A, LeGoff J, Gresenguet G (2008). High prevalence of antiretroviral drug resistance mutations in HIV-1 non-B subtype strains from African children receiving antiretroviral therapy regimen according to the 2006 revised WHO recommendations. J Acquir Immune Defic Syndr.

[54] Bangsberg DR, Kroetz DL, Deeks SG (2007). Adherence–resistance relationships to combination HIV antiretroviral therapy. Curr HIV/AIDS Rep.

[55] Reisner SL, Mimiaga MJ, Skeer M, Perkovich B, Johnson CV, Safren SA (2009). A review of HIV antiretroviral adherence and intervention studies among HIV-infected youth. Top HIV Med.

[56] Murphy DA, Belzer M, Durako SJ, Sarr M, Wilson CM, Muenz LR (2005). Longitudinal antiretroviral adherence among adolescents infected with human immunodeficiency virus. Arch Pediatr Adolesc Med.

[57] Nachega JB, Hislop M, Nguyen H, Dowdy DW, Chaisson RE, Regensberg L (2009). Antiretroviral therapy adherence, virologic and immunologic outcomes in adolescents compared with adults in southern Africa. J Acquir Immune Defic Syndromes.

[58] Williams PL, Storm D, Montepiedra G, Nichols S, Kammerer B, Sirois PA (2006). Predictors of adherence to antiretroviral medications in children and adolescents with HIV infection. Pediatrics.

[59] Schulte J, Dominguez K, Sukalac T, Bohannon B, Fowler MG (2007). Pediatric Spectrum of HIVDC. Declines in low birthweight and preterm birth among infants who were born to HIV-infected women during an era of increased use of maternal antiretroviral drugs: pediatric spectrum of HIV disease, 1989–2004. Pediatrics.

[60] Cotter AM, Garcia AG, Duthely ML, Luke B, O'Sullivan MJ (2006). Is antiretroviral therapy during pregnancy associated with an increased risk of preterm delivery, low birth weight, or stillbirth?. J Infect Dis.

[61] Ravizza M, Martinelli P, Bucceri A, Fiore S, Alberico S, Tamburrini E (2007). Treatment with protease inhibitors and coinfection with hepatitis C virus are independent predictors of preterm delivery in HIV-infected pregnant women. J Infect Dis.

[62] Thorne C, Patel D, Newell ML (2004). Increased risk of adverse pregnancy outcomes in HIV-infected women treated with highly active antiretroviral therapy in Europe. AIDS.

[63] Townsend C, Schulte J, Thorne C, Dominguez KI, Tookey PA, Cortina-Borja M (2010). Antiretroviral therapy and preterm delivery – a pooled analysis of data from the United States and Europe. BJOG.

[64] Townsend CL, Tookey PA, Newell ML, Cortina-Borja M (2010). Antiretroviral therapy in pregnancy: balancing the risk of preterm delivery with prevention of mother-to-child HIV transmission. Antivir Ther.

[65] Powis KM, Kitch D, Ogwu A, Hughes MD, Lockman S, Leidner J (2011). Increased risk of preterm delivery among HIV-infected women randomized to protease versus nucleoside reverse transcriptase inhibitor-based HAART during pregnancy. J Infect Dis.

[66] Kourtis AP, Schmid CH, Jamieson DJ, Lau J (2007). Use of antiretroviral therapy in pregnant HIV-infected women and the risk of premature delivery: a meta-analysis. AIDS.

[67] Sibiude J, Warszawski J, Tubiana R, Dollfus C, Faye A, Rouzioux C (2012). Premature delivery in HIV-infected women starting protease inhibitor therapy during pregnancy: role of the ritonavir boost?. Clin Infect Dis.

[68] Thorne C, Townsend CL (2012). A new piece in the puzzle of antiretroviral therapy in pregnancy and preterm delivery risk. Clin Infect Dis.

[69] Beck S, Wojdyla D, Say L, Betran AP, Merialdi M, Requejo JH (2010). The worldwide incidence of preterm birth: a systematic review of maternal mortality and morbidity. Bull World Health Organ.

[70] Kim RJ, Rutstein RM (2010). Impact of antiretroviral therapy on growth, body composition and metabolism in pediatric HIV patients. Paediatr Drugs.

[71] Williams PL, Abzug MJ, Jacobson DL, Wang J, Van Dyke RB, Hazra R (2013). Pubertal onset in children with perinatal HIV infection in the era of combination antiretroviral treatment. AIDS.

[72] Grantham-McGregor S, Cheung YB, Cueto S, Glewwe P, Richter L, Strupp B (2007). Developmental potential in the first 5 years for children in developing countries. Lancet.

[73] Chiriboga CA, Fleishman S, Champion S, Gaye-Robinson L, Abrams EJ (2005). Incidence and prevalence of HIV encephalopathy in children with HIV infection receiving highly active anti-retroviral therapy (HAART). J Pediatr.

[74] Shanbhag MC, Rutstein RM, Zaoutis T, Zhao H, Chao D, Radcliffe J (2005). Neurocognitive functioning in pediatric human immunodeficiency virus infection: effects of combined therapy. Arch Pediatr Adolesc Med.

[75] Patel K, Ming X, Williams PL, Robertson KR, Oleske JM, Seage GR (2009). Impact of HAART and CNS-penetrating antiretroviral regimens on HIV encephalopathy among perinatally infected children and adolescents. AIDS.

[76] Ettenhofer ML, Foley J, Castellon SA, Hinkin CH (2010). Reciprocal prediction of medication adherence and neurocognition in HIV/AIDS. Neurology.

[77] Malee K, Williams PL, Montepiedra G, Nichols S, Sirois PA, Storm D (2009). The role of cognitive functioning in medication adherence of children and adolescents with HIV infection. J Pediatr Psychol.

[78] Paramesparan Y, Garvey LJ, Ashby J, Foster CJ, Fidler S, Winston A (2010). High rates of asymptomatic neurocognitive impairment in vertically acquired HIV-1-infected adolescents surviving to adulthood. J Acquir Immune Defic Syndr.

[79] Ruel TD, Boivin MJ, Boal HE, Bangirana P, Charlebois E, Havlir DV (2012). Neurocognitive and motor deficits in HIV-infected Ugandan children with high CD4 cell counts. Clin Infect Dis.

[80] Bagenda D, Nassali A, Kalyesubula I, Sherman B, Drotar D, Boivin MJ (2006). Health, neurologic, and cognitive status of HIV-infected, long-surviving, and antiretroviral-naive Ugandan children. Pediatrics.

[81] Laughton B, Cornell M, Boivin M, Van Rie A (2013). Neurodevelopment in perinatally HIV-infected children: a concern for adolescence. J Int AIDS Soc.

[82] Cysique LA, Waters EK, Brew BJ (2011). Central nervous system antiretroviral efficacy in HIV infection: a qualitative and quantitative review and implications for future research. BMC Neurol.

[83] Eisfeld C, Reichelt D, Evers S, Husstedt I (2013). CSF penetration by antiretroviral drugs. CNS Drugs.

[84] Heaton RK, Franklin DR, Ellis RJ, McCutchan JA, Letendre SL, Leblanc S (2011). HIV-associated neurocognitive disorders before and during the era of combination antiretroviral therapy: differences in rates, nature, and predictors. J Neurovirol.

[85] Tamula MA, Wolters PL, Walsek C, Zeichner S, Civitello L (2003). Cognitive decline with immunologic and virologic stability in four children with human immunodeficiency virus disease. Pediatrics.

[86] Crowell CS, Malee KM, Yogev R, Muller WJ (2014). Neurologic disease in HIV-infected children and the impact of combination antiretroviral therapy. Rev Med Virol.

[87] Blanchette N, Smith ML, King S, Fernandes-Penney A, Read S (2002). Cognitive development in school-age children with vertically transmitted HIV infection. Dev Neuropsychol.

[88] Foster CJ, Biggs RL, Melvin D, Walters MD, Tudor-Williams G, Lyall EG (2006). Neurodevelopmental outcomes in children with HIV infection under 3 years of age. Dev Med Child Neurol.

[89] Jeremy RJ, Kim S, Nozyce M, Nachman S, McIntosh K, Pelton SI (2005). Neuropsychological functioning and viral load in stable antiretroviral therapy-experienced HIV-infected children. Pediatrics.

[90] Koekkoek S, de Sonneville LM, Wolfs TF, Licht R, Geelen SP (2008). Neurocognitive function profile in HIV-infected school-age children. Eur J Paediatr Neurol.

[91] Lindsey JC, Malee KM, Brouwers P, Hughes MD, PACTG 219C Study Team (2007). Neurodevelopmental functioning in HIV-infected infants and young children before and after the introduction of protease inhibitor-based highly active antiretroviral therapy. Pediatrics.

[92] Nozyce ML, Lee SS, Wiznia A, Nachman S, Mofenson LM, Smith ME (2006). A behavioral and cognitive profile of clinically stable HIV-infected children. Pediatrics.

[93] van Arnhem LA, Bunders MJ, Scherpbier HJ, Majoie CB, Reneman L, Frinking O (2013). Neurologic abnormalities in HIV-1 infected children in the era of combination antiretroviral therapy. PLoS One.

[94] Walker SY, Pierre RB, Christie CD, Chang SM (2013). Neurocognitive function in HIV-positive children in a developing country. Int J Infect Dis.

[95] Cohen S, Ter Stege JA, Geurtsen GJ, Scherpbier HJ, Kuijpers TW, Reiss P (2015). Poorer cognitive performance in perinatally HIV-infected children versus healthy socioeconomically matched controls. Clin Infect Dis.

[96] Laughton B, Cornell M, Grove D, Kidd M, Springer PE, Dobbels E (2012). Early antiretroviral therapy improves neurodevelopmental outcomes in infants. AIDS.

[97] Crowell CS, Huo Y, Tassiopoulos K, Malee KM, Yogev R, Hazra R (2015). Early viral suppression improves neurocognitive outcomes in HIV-infected children. AIDS.

[98] Puthanakit T, Ananworanich J, Vonthanak S, Kosalaraksa P, Hansudewechakul R, van der Lugt J (2013). Cognitive function and neurodevelopmental outcomes in HIV-infected children older than 1 year of age randomized to early versus deferred antiretroviral therapy: the PREDICT neurodevelopmental study. Pediatr Infect Dis J.

[99] Puthanakit T, Saphonn V, Ananworanich J, Kosalaraksa P, Hansudewechakul R, Vibol U (2012). Early versus deferred antiretroviral therapy for children older than 1 year infected with HIV (PREDICT): a multicentre, randomised, open-label trial. Lancet Infect Dis.

[100] Pratt RD, Nichols S, McKinney N, Kwok S, Dankner WM, Spector SA (1996). Virologic markers of human immunodeficiency virus type 1 in cerebrospinal fluid of infected children. J Infect Dis.

[101] Sei S, Stewart SK, Farley M, Mueller BU, Lane JR, Robb ML (1996). Evaluation of human immunodeficiency virus (HIV) type 1 RNA levels in cerebrospinal fluid and viral resistance to zidovudine in children with HIV encephalopathy. J Infect Dis.

[102] Cysique LA, Vaida F, Letendre S, Gibson S, Cherner M, Woods SP (2009). Dynamics of cognitive change in impaired HIV-positive patients initiating antiretroviral therapy. Neurology.

[103] Smurzynski M, Wu K, Letendre S, Robertson K, Bosch RJ, Clifford DB (2011). Effects of central nervous system antiretroviral penetration on cognitive functioning in the ALLRT cohort. AIDS.

[104] Tozzi V, Balestra P, Salvatori MF, Vlassi C, Liuzzi G, Giancola ML (2009). Changes in cognition during antiretroviral therapy: comparison of 2 different ranking systems to measure antiretroviral drug efficacy on HIV-associated neurocognitive disorders. J Acquir Immune Defic Syndr.

[105] Garvey L, Surendrakumar V, Winston A (2011). Low rates of neurocognitive impairment are observed in neuro-asymptomatic HIV-infected subjects on effective antiretroviral therapy. HIV Clin Trials.

[106] Marra CM, Zhao Y, Clifford DB, Letendre S, Evans S, Henry K (2009). Impact of combination antiretroviral therapy on cerebrospinal fluid HIV RNA and neurocognitive performance. AIDS.

[107] Grody WW, Cheng L, Lewis W (1990). Infection of the heart by the human immunodeficiency virus. Am J Cardiol.

[108] Lipshultz SE, Chanock S, Sanders SP, Colan SD, Perez-Atayde A, McIntosh K (1989). Cardiovascular manifestations of human immunodeficiency virus infection in infants and children. Am J Cardiol.

[109] McDonald CL, Kaltman JR (2009). Cardiovascular disease in adult and pediatric HIV/AIDS. J Am Coll Cardiol.

[110] Calabrese LH, Proffitt MR, Yen-Lieberman B, Hobbs RE, Ratliff NB (1987). Congestive cardiomyopathy and illness related to the acquired immunodeficiency syndrome (AIDS) associated with isolation of retrovirus from myocardium. Ann Intern Med.

[111] Lipshultz SE, Fox CH, Perez-Atayde AR, Sanders SP, Colan SD, McIntosh K (1990). Identification of human immunodeficiency virus-1 RNA and DNA in the heart of a child with cardiovascular abnormalities and congenital acquired immune deficiency syndrome. Am J Cardiol.

[112] Fiala M, Popik W, Qiao JH, Lossinsky AS, Alce T, Tran K (2004). HIV-1 induces cardiomyopathy by cardiomyocyte invasion and gp120, Tat, and cytokine apoptotic signaling. Cardiovasc Toxicol.

[113] Monsuez JJ, Escaut L, Teicher E, Charniot JC, Vittecoq D (2007). Cytokines in HIV-associated cardiomyopathy. Int J Cardiol.

[114] Satoh M, Minami Y, Takahashi Y, Nakamura M (2008). Immune modulation: role of the inflammatory cytokine cascade in the failing human heart. Curr Heart Fail Rep.

[115] Lewis W (2000). Cardiomyopathy in AIDS: a pathophysiological perspective. Prog Cardiovasc Dis.

[116] Lewis W (2003). Mitochondrial DNA replication, nucleoside reverse-transcriptase inhibitors, and AIDS cardiomyopathy. Prog Cardiovasc Dis.

[117] Lewis W, Papoian T, Gonzalez B, Louie H, Kelly DP, Payne RM (1991). Mitochondrial ultrastructural and molecular changes induced by zidovudine in rat hearts. Lab Investig.

[118] Benbrik E, Chariot P, Bonavaud S, Ammi-Said M, Frisdal E, Rey C (1997). Cellular and mitochondrial toxicity of zidovudine (AZT), didanosine (ddI) and zalcitabine (ddC) on cultured human muscle cells. J Neurol Sci.

[119] Birkus G, Hitchcock MJ, Cihlar T (2002). Assessment of mitochondrial toxicity in human cells treated with tenofovir: comparison with other nucleoside reverse transcriptase inhibitors. Antimicrob Agents Chemother.

[120] Brinkman K, ter Hofstede HJ, Burger DM, Smeitink JA, Koopmans PP (1998). Adverse effects of reverse transcriptase inhibitors: mitochondrial toxicity as common pathway. AIDS.

[121] Medina DJ, Tsai CH, Hsiung GD, Cheng YC (1994). Comparison of mitochondrial morphology, mitochondrial DNA content, and cell viability in cultured cells treated with three anti-human immunodeficiency virus dideoxynucleosides. Antimicrob Agents Chemother.

[122] Saitoh A, Fenton T, Alvero C, Fletcher CV, Spector SA (2007). Impact of nucleoside reverse transcriptase inhibitors on mitochondria in human immunodeficiency virus type 1-infected children receiving highly active antiretroviral therapy. Antimicrob Agents Chemother.

[123] The pediatric pulmonary and cardiovascular complications of vertically transmitted human immunodeficiency virus (P2C2 HIV) infection study: design and methods (1996). The P2C2 HIV Study Group. J Clin Epidemiol.

[124] Starc TJ, Lipshultz SE, Easley KA, Kaplan S, Bricker JT, Colan SD (2002). Incidence of cardiac abnormalities in children with human immunodeficiency virus infection: the prospective P2C2 HIV study. J Pediatr.

[125] Patel K, Van Dyke RB, Mittleman MA, Colan SD, Oleske JM, Seage GR (2012). The impact of HAART on cardiomyopathy among children and adolescents perinatally infected with HIV-1. AIDS.

[126] Lipshultz SE, Williams PL, Wilkinson JD, Leister EC, Van Dyke RB, Shearer WT (2013). Cardiac status of children infected with human immunodeficiency virus who are receiving long-term combination antiretroviral therapy: results from the Adolescent Master Protocol of the Multicenter Pediatric HIV/AIDS Cohort Study. JAMA Pediatr.

[127] Lipshultz SE, Shearer WT, Thompson B, Rich KC, Cheng I, Orav EJ (2011). Cardiac effects of antiretroviral therapy in HIV-negative infants born to HIV-positive mothers: NHLBI CHAART-1 (National Heart, Lung, and Blood Institute Cardiovascular Status of HAART Therapy in HIV-Exposed Infants and Children cohort study). J Am Coll Cardiol.

[128] Williams PL, Crain MJ, Yildirim C, Hazra R, Van Dyke RB, Rich K (2015). Congenital anomalies and in utero antiretroviral exposure in human immunodeficiency virus-exposed uninfected infants. JAMA Pediatr.

[129] Brown SC, Schoeman CJ, Bester CJ (2005). Cardiac findings in children admitted to a hospital general ward in South Africa: a comparison of HIV-infected and uninfected children. Cardiovasc J S Afr.

[130] Lubega S, Zirembuzi GW, Lwabi P (2005). Heart disease among children with HIV/AIDS attending the paediatric infectious disease clinic at Mulago Hospital. Afr Health Sci.

[131] Okoromah CA, Ojo OO, Ogunkunle OO (2012). Cardiovascular dysfunction in HIV-infected children in a sub-Saharan African country: comparative cross-sectional observational study. J Trop Pediatr.

[132] Lai W, Mertens L, Cohen M, Geva T (2009). Echocardiography in pediatric and congential heart disease.

[133] Sims A, Frank L, Cross R, Clauss S, Dimock D, Purdy J (2012). Abnormal cardiac strain in children and young adults with HIV acquired in early life. J Am Soc Echocardiogr.

[134] Price JC, Thio CL (2010). Liver disease in the HIV-infected individual. Clin Gastroenterol Hepatol.

[135] Siberry GK, Cohen RA, Harris DR, Cruz ML, Oliveira R, Peixoto MF (2014). Prevalence and predictors of elevated aspartate aminotransferase-to-platelet ratio index in Latin American perinatally HIV-infected children. Pediatr Infect Dis J.

[136] Siberry GK, Patel K, Pinto JA, Puga A, Mirza A, Miller TL (2014). Elevated aspartate aminotransferase-to-platelet ratio index in perinatally HIV-infected children in the United States. Pediatr Infect Dis J.

[137] Bamford A, Turkova A, Lyall H, Foster C, Klein N, Bastiaans D (2015). Paediatric European Network for Treatment of AIDS (PENTA) guidelines for treatment of paediatric HIV-1 infection 2015: optimizing health in preparation for adult life. HIV Med.

[138] Rubio A, Monpoux F, Huguon E, Truchi R, Triolo V, Rosenthal-Allieri MA (2009). Noninvasive procedures to evaluate liver involvement in HIV-1 vertically infected children. J Pediatr Gastroenterol Nutr.

[139] Martinez-Sierra C, Arizcorreta A, Diaz F, Roldan R, Martin-Herrera L, Perez-Guzman E (2003). Progression of chronic hepatitis C to liver fibrosis and cirrhosis in patients coinfected with hepatitis C virus and human immunodeficiency virus. Clin Infect Dis.

[140] England K, Thorne C, Newell ML (2006). Vertically acquired paediatric coinfection with HIV and hepatitis C virus. Lancet Infect Dis.

[141] Abera B, Zenebe Y, Mulu W, Kibret M, Kahsu G (2014). Seroprevalence of hepatitis B and C viruses and risk factors in HIV infected children at the Felgehiwot referral hospital, Ethiopia. BMC Res Notes.

[142] Toussi SS, Abadi J, Rosenberg M, Levanon D (2007). Prevalence of hepatitis B and C virus infections in children infected with HIV. Clin Infect Dis.

[143] Zhou S, Zhao Y, He Y, Li H, Bulterys M, Sun X (2010). Hepatitis B and hepatitis C seroprevalence in children receiving antiretroviral therapy for human immunodeficiency virus-1 infection in China, 2005–2009. J Acquir Immune Defic Syndr.

[144] Healy SA, Gupta S, Melvin AJ (2013). HIV/HBV coinfection in children and antiviral therapy. Expert Rev Anti Infect Ther.

[145] Siberry GK, Abzug MJ, Nachman S, Brady MT, Dominguez KL, Handelsman E (2013). Guidelines for the prevention and treatment of opportunistic infections in HIV-exposed and HIV-infected children: recommendations from the National Institutes of Health, Centers for Disease Control and Prevention, the HIV Medicine Association of the Infectious Diseases Society of America, the Pediatric Infectious Diseases Society, and the American Academy of Pediatrics. Pediatr Infect Dis J.

[146] Bhimma R, Purswani MU, Kala U (2013). Kidney disease in children and adolescents with perinatal HIV-1 infection. J Int AIDS Soc.

[147] Lescure FX, Flateau C, Pacanowski J, Brocheriou I, Rondeau E, Girard PM (2012). HIV-associated kidney glomerular diseases: changes with time and HAART. Nephrol Dial Transplant.

[148] Ray PE (2009). Taking a hard look at the pathogenesis of childhood HIV-associated nephropathy. Pediatr Nephrol.

[149] Wyatt CM, Meliambro K, Klotman PE (2012). Recent progress in HIV-associated nephropathy. Ann Rev Med.

[150] Mitchell CD, Chernoff MC, Seage GR, Purswani MU, Spiegel HM, Zilleruelo G (2015). Predictors of resolution and persistence of renal laboratory abnormalities in pediatric HIV infection. Pediatr Nephrol.

[151] Della Negra M, de Carvalho AP, de Aquino MZ, da Silva MT, Pinto J, White K (2012). A randomized study of tenofovir disoproxil fumarate in treatment-experienced HIV-1 infected adolescents. Pediatr Infect Dis J.

[152] Pontrelli G, Cotugno N, Amodio D, Zangari P, Tchidjou HK, Baldassari S (2012). Renal function in HIV-infected children and adolescents treated with tenofovir disoproxil fumarate and protease inhibitors. BMC Infect Dis.

[153] Purswani M, Patel K, Kopp JB, Seage GR, Chernoff MC, Hazra R (2013). Tenofovir treatment duration predicts proteinuria in a multiethnic United States Cohort of children and adolescents with perinatal HIV-1 infection. Pediatr Infect Dis J.

[154] Vigano A, Bedogni G, Manfredini V, Giacomet V, Cerini C, di Nello F (2011). Long-term renal safety of tenofovir disoproxil fumarate in vertically HIV-infected children, adolescents and young adults: a 60-month follow-up study. Clin Drug Investig.

[155] Ramsuran D, Bhimma R, Ramdial PK, Naicker E, Adhikari M, Deonarain J (2012). The spectrum of HIV-related nephropathy in children. Pediatr Nephrol.

[156] Bostrom MA, Freedman BI (2010). The spectrum of MYH9-associated nephropathy. Clin J Am Soc Nephrol.

[157] Kopp JB, Nelson GW, Sampath K, Johnson RC, Genovese G, An P (2011). APOL1 genetic variants in focal segmental glomerulosclerosis and HIV-associated nephropathy. J Am Soc Nephrol.

[158] Fabian J, Naicker S (2009). HIV and kidney disease in sub-Saharan Africa. Nat Rev Nephrol.

[159] Purswani MU, Chernoff MC, Mitchell CD, Seage GR, Zilleruelo G, Abitbol C (2012). Chronic kidney disease associated with perinatal HIV infection in children and adolescents. Pediatr Nephrol.

[160] Chaparro AI, Mitchell CD, Abitbol CL, Wilkinson JD, Baldarrago G, Lopez E (2008). Proteinuria in children infected with the human immunodeficiency virus. J Pediatr.

[161] Andiman WA, Chernoff MC, Mitchell C, Purswani M, Oleske J, Williams PL (2009). Incidence of persistent renal dysfunction in human immunodeficiency virus-infected children: associations with the use of antiretrovirals, and other nephrotoxic medications and risk factors. Pediatr Infect Dis J.

[162] Tourret J, Tostivint I, du Montcel ST, Bragg-Gresham J, Karie S, Vigneau C (2006). Outcome and prognosis factors in HIV-infected hemodialysis patients. Clin J Am Soc Nephrol.

[163] Gordillo R, Del Rio M, Woroniecki RP (2011). Dialysis-associated morbidity, ultrafiltration, and cardiovascular variables in children with HIV infection. Clin Nephrol.

[164] Gruber SA, Doshi MD, Cincotta E, Brown KL, Singh A, Morawski K (2008). Preliminary experience with renal transplantation in HIV+ recipients: low acute rejection and infection rates. Transplantation.

[165] Kumar MS, Sierka DR, Damask AM, Fyfe B, McAlack RF, Heifets M (2005). Safety and success of kidney transplantation and concomitant immunosuppression in HIV-positive patients. Kidney Int.

[166] Roland ME, Stock PG (2003). Review of solid-organ transplantation in HIV-infected patients. Transplantation.

[167] Kreuzer KA, Rockstroh JK (1997). Pathogenesis and pathophysiology of anemia in HIV infection. Ann Hematol.

[168] Wiener LS, Kohrt BA, Battles HB, Pao M (2011). The HIV experience: youth identified barriers for transitioning from pediatric to adult care. J Pediatr Psychol.

[169] Biggar RJ, Rabkin CS (1996). The epidemiology of AIDS – related neoplasms. Hematol Oncol Clin North Am.

[170] Brent AJ, Oundo JO, Mwangi I, Ochola L, Lowe B, Berkley JA (2006). Salmonella bacteremia in Kenyan children. Pediatr Infect Dis J.

[171] Graham SM, Walsh AL, Molyneux EM, Phiri AJ, Molyneux ME (2000). Clinical presentation of non-typhoidal Salmonella bacteraemia in Malawian children. Trans R Soc Trop Med Hyg.

[172] Sirisanthana V, Sirisanthana T (1995). Disseminated *Penicillium marneffei* infection in human immunodeficiency virus-infected children. Pediatr Infect Dis J.

[173] Clark TD, Mmiro F, Ndugwa C, Perry RT, Jackson JB, Melikian G (2002). Risk factors and cumulative incidence of anaemia among human immunodeficiency virus-infected children in Uganda. Ann Trop Paediatr.

[174] Hoyt L, Oleske J, Holland B, Connor E (1992). Nontuberculous mycobacteria in children with acquired immunodeficiency syndrome. Pediatr Infect Dis J.

[175] Otieno RO, Ouma C, Ong'echa JM, Keller CC, Were T, Waindi EN (2006). Increased severe anemia in HIV-1-exposed and HIV-1-positive infants and children during acute malaria. AIDS.

[176] van Eijk AM, Ayisi JG, Ter Kuile FO, Misore AO, Otieno JA, Kolczak MS (2002). Malaria and human immunodeficiency virus infection as risk factors for anemia in infants in Kisumu, western Kenya. Am J Trop Med Hyg.

[177] Capparelli EV, Englund JA, Connor JD, Spector SA, McKinney RE, Palumbo P (2003). Population pharmacokinetics and pharmacodynamics of zidovudine in HIV-infected infants and children. J Clin Pharmacol.

[178] Englund JA, Baker CJ, Raskino C, McKinney RE, Petrie B, Fowler MG (1997). Zidovudine, didanosine, or both as the initial treatment for symptomatic HIV-infected children. AIDS Clinical Trials Group (ACTG) Study 152 Team. N Engl J Med.

[179] Sperling RS, Shapiro DE, McSherry GD, Britto P, Cunningham BE, Culnane M (1998). Safety of the maternal-infant zidovudine regimen utilized in the Pediatric AIDS Clinical Trial Group 076 Study. AIDS.

[180] Connor EM, Sperling RS, Gelber R, Kiselev P, Scott G, O'Sullivan MJ (1994). Reduction of maternal-infant transmission of human immunodeficiency virus type 1 with zidovudine treatment. Pediatric AIDS Clinical Trials Group Protocol 076 Study Group. N Engl J Med.

[181] Dryden-Peterson S, Shapiro RL, Hughes MD, Powis K, Ogwu A, Moffat C (2011). Increased risk of severe infant anemia after exposure to maternal HAART, Botswana. J Acquir Immune Defic Syndr.

[182] Calis JC, van Hensbroek MB, de Haan RJ, Moons P, Brabin BJ, Bates I (2008). HIV-associated anemia in children: a systematic review from a global perspective. AIDS.

[183] Marti-Carvajal AJ, Sola I, Pena-Marti GE, Comunian-Carrasco G (2011). Treatment for anemia in people with AIDS. Cochrane Database Syst Rev.

[184] Leonard EG, McComsey GA (2005). Antiretroviral therapy in HIV-infected children: the metabolic cost of improved survival. Infect Dis Clin North Am.

[185] McComsey GA, Leonard E (2004). Metabolic complications of HIV therapy in children. AIDS.

[186] Aldrovandi GM, Lindsey JC, Jacobson DL, Zadzilka A, Sheeran E, Moye J (2009). Morphologic and metabolic abnormalities in vertically HIV-infected children and youth. AIDS.

[187] Wohl DA, Brown TT (2008). Management of morphologic changes associated with antiretroviral use in HIV-infected patients. J Acquir Immune Defic Syndr.

[188] Barbaro G (2007). Visceral fat as target of highly active antiretroviral therapy-associated metabolic syndrome. Curr Pharm Des.

[189] Zanone Poma B, Riva A, Nasi M, Cicconi P, Broggini V, Lepri AC (2008). Genetic polymorphisms differently influencing the emergence of atrophy and fat accumulation in HIV-related lipodystrophy. AIDS.

[190] Amaya RA, Kozinetz CA, McMeans A, Schwarzwald H, Kline MW (2002). Lipodystrophy syndrome in human immunodeficiency virus-infected children. Pediatr Infect Dis J.

[191] Babl FE, Regan AM, Pelton SI (1999). Abnormal body-fat distribution in HIV-1-infected children on antiretrovirals. Lancet.

[192] European Paediatric Lipodystrophy Group (2004). Antiretroviral therapy, fat redistribution and hyperlipidaemia in HIV-infected children in Europe. AIDS.

[193] Kinabo GD, Sprengers M, Msuya LJ, Shayo AM, van Asten H, Dolmans WM (2013). Prevalence of lipodystrophy in HIV-infected children in Tanzania on highly active antiretroviral therapy. Pediatr Infect Dis J.

[194] Piloya T, Bakeera-Kitaka S, Kekitiinwa A, Kamya MR (2012). Lipodystrophy among HIV-infected children and adolescents on highly active antiretroviral therapy in Uganda: a cross sectional study. J Int AIDS Soc.

[195] Sanchez Torres AM, Munoz Muniz R, Madero R, Borque C, Garcia-Miguel MJ, De Jose Gomez MI (2005). Prevalence of fat redistribution and metabolic disorders in human immunodeficiency virus-infected children. Eur J Pediatr.

[196] Taylor P, Worrell C, Steinberg SM, Hazra R, Jankelevich S, Wood LV (2004). Natural history of lipid abnormalities and fat redistribution among human immunodeficiency virus-infected children receiving long-term, protease inhibitor-containing, highly active antiretroviral therapy regimens. Pediatrics.

[197] Alam N, Cortina-Borja M, Goetghebuer T, Marczynska M, Vigano A, Thorne C (2012). Body fat abnormality in HIV-infected children and adolescents living in Europe: prevalence and risk factors. J Acquir Immune Defic Syndr.

[198] Dapena M, Jimenez B, Noguera-Julian A, Soler-Palacin P, Fortuny C, Lahoz R (2012). Metabolic disorders in vertically HIV-infected children: future adults at risk for cardiovascular disease. J Pediatr Endocrinol Metab.

[199] Aurpibul L, Puthanakit T, Lee B, Mangklabruks A, Sirisanthana T, Sirisanthana V (2007). Lipodystrophy and metabolic changes in HIV-infected children on non-nucleoside reverse transcriptase inhibitor-based antiretroviral therapy. Antivir Ther.

[200] Lainka E, Oezbek S, Falck M, Ndagijimana J, Niehues T (2002). Marked dyslipidemia in human immunodeficiency virus-infected children on protease inhibitor-containing antiretroviral therapy. Pediatrics.

[201] Leonard EG, McComsey GA (2003). Metabolic complications of antiretroviral therapy in children. Pediatr Infect Dis J.

[202] Ammassari A, Antinori A, Cozzi-Lepri A, Trotta MP, Nasti G, Ridolfo AL (2002). Relationship between HAART adherence and adipose tissue alterations. J Acquir Immune Defic Syndr.

[203] Holzemer WL, Uys L, Makoae L, Stewart A, Phetlhu R, Dlamini PS (2007). A conceptual model of HIV/AIDS stigma from five African countries. J Adv Nurs.

[204] Makoae LN, Portillo CJ, Uys LR, Dlamini PS, Greeff M, Chirwa M (2009). The impact of taking or not taking ARVs on HIV stigma as reported by persons living with HIV infection in five African countries. AIDS Care.

[205] Mutimura E, Stewart A, Crowther NJ (2007). Assessment of quality of life in HAART-treated HIV-positive subjects with body fat redistribution in Rwanda. AIDS Res Ther.

[206] Miller TL, Orav EJ, Lipshultz SE, Arheart KL, Duggan C, Weinberg GA (2008). Risk factors for cardiovascular disease in children infected with human immunodeficiency virus-1. J Pediatr.

[207] Carr A, Samaras K, Burton S, Law M, Freund J, Chisholm DJ (1998). A syndrome of peripheral lipodystrophy, hyperlipidaemia and insulin resistance in patients receiving HIV protease inhibitors. AIDS.

[208] Beregszaszi M, Dollfus C, Levine M, Faye A, Deghmoun S, Bellal N (2005). Longitudinal evaluation and risk factors of lipodystrophy and associated metabolic changes in HIV-infected children. J Acquir Immune Defic Syndr.

[209] Rosso R, Parodi A, d'Annunzio G, Ginocchio F, Nicolini L, Torrisi C (2007). Evaluation of insulin resistance in a cohort of HIV-infected youth. Eur J Endocrinol.

[210] Carter RJ, Wiener J, Abrams EJ, Farley J, Nesheim S, Palumbo P (2006). Dyslipidemia among perinatally HIV-infected children enrolled in the PACTS-HOPE cohort, 1999–2004: a longitudinal analysis. J Acquir Immune Defic Syndr.

[211] Chantry CJ, Hughes MD, Alvero C, Cervia JS, Meyer WA, Hodge J (2008). Lipid and glucose alterations in HIV-infected children beginning or changing antiretroviral therapy. Pediatrics.

[212] Kim JY, Zaoutis T, Chu J, Zhao H, Rutstein R (2009). Effects of highly active antiretroviral therapy (HAART) on cholesterol in HIV-1 infected children: a retrospective cohort study. Pharmacoepidemiol Drug Saf.

[213] Tassiopoulos K, Williams PL, Seage GR, Crain M, Oleske J, Farley J (2008). Association of hypercholesterolemia incidence with antiretroviral treatment, including protease inhibitors, among perinatally HIV-infected children. J Acquir Immune Defic Syndr.

[214] Jacobson DL, Williams P, Tassiopoulos K, Melvin A, Hazra R, Farley J (2011). Clinical management and follow-up of hypercholesterolemia among perinatally HIV-infected children enrolled in the PACTG 219C study. J Acquir Immune Defic Syndr.

[215] Gonzalez-Tome MI, Amador JT, Pena MJ, Gomez ML, Conejo PR, Fontelos PM (2008). Outcome of protease inhibitor substitution with nevirapine in HIV-1 infected children. BMC Infect Dis.

[216] McComsey G, Bhumbra N, Ma JF, Rathore M, Alvarez A (2003). First Pediatric Switch Study. Impact of protease inhibitor substitution with efavirenz in HIV-infected children: results of the First Pediatric Switch Study. Pediatrics.

[217] Strehlau R, Coovadia A, Abrams EJ, Martens L, Arpadi S, Meyers T (2012). Lipid profiles in young HIV-infected children initiating and changing antiretroviral therapy. J Acquir Immune Defic Syndr.

[218] Vigano A, Zuccotti GV, Puzzovio M, Pivetti V, Zamproni I, Cerini C (2010). Tenofovir disoproxil fumarate and bone mineral density: a 60-month longitudinal study in a cohort of HIV-infected youths. Antivir Ther.

[219] Daniels SR, Greer FR, Committee on Nutrition (2008). Lipid screening and cardiovascular health in childhood. Pediatrics.

[220] Ten S, Maclaren N (2004). Insulin resistance syndrome in children. J Clin Endocrinol Metab.

[221] Alves C, Oliveira AC, Brites C (2008). Lipodystrophic syndrome in children and adolescents infected with the human immunodeficiency virus. Braz J Infect Dis.

[222] Bockhorst JL, Ksseiry I, Toye M, Chipkin SR, Stechenberg BW, Fisher DJ (2003). Evidence of human immunodeficiency virus-associated lipodystrophy syndrome in children treated with protease inhibitors. Pediatr Infect Dis J.

[223] Aboud M, Elgalib A, Kulasegaram R, Peters B (2007). Insulin resistance and HIV infection: a review. Int J Clin Pract.

[224] Wierzbicki AS, Purdon SD, Hardman TC, Kulasegaram R, Peters BS (2008). HIV lipodystrophy and its metabolic consequences: implications for clinical practice. Curr Med Res Opin.

[225] Young F, Critchley JA, Johnstone LK, Unwin NC (2009). A review of co-morbidity between infectious and chronic disease in sub Saharan Africa: TB and diabetes mellitus, HIV and metabolic syndrome, and the impact of globalization. Global Health.

[226] Hazra R, Hance LF, Monteiro JP, Ruz NP, Machado DM, Saavedra M (2013). Insulin resistance and glucose and lipid concentrations in a cohort of perinatally HIV-infected Latin American children. Pediatr Infect Dis J.

[227] Lee B, Aurpibul L, Sirisanthana V, Mangklabruks A, Sirisanthana T, Puthanakit T (2009). Low prevalence of insulin resistance among HIV-infected children receiving nonnucleoside reverse transcriptase inhibitor-based highly active antiretroviral therapy in Thailand. HIV Med.

[228] Geffner ME, Patel K, Miller TL, Hazra R, Silio M, Van Dyke RB (2011). Factors associated with insulin resistance among children and adolescents perinatally infected with HIV-1 in the pediatric HIV/AIDS cohort study. Horm Res Paediatr.

[229] Vigano A, Brambilla P, Cafarelli L, Giacomet V, Borgonovo S, Zamproni I (2007). Normalization of fat accrual in lipoatrophic, HIV-infected children switched from stavudine to tenofovir and from protease inhibitor to efavirenz. Antivir Ther.

[230] Vigano A, Brambilla P, Pattarino G, Stucchi S, Fasan S, Raimondi C (2009). Long-term evaluation of glucose homeostasis in a cohort of HAART-treated HIV-infected children: a longitudinal, observational cohort study. Clin Drug Investig.

[231] Vigano A, Cerini C, Pattarino G, Fasan S, Zuccotti GV (2010). Metabolic complications associated with antiretroviral therapy in HIV-infected and HIV-exposed uninfected paediatric patients. Expert Opin Drug Saf.

[232] Barlow-Mosha L, Eckard AR, McComsey GA, Musoke PM (2013). Metabolic complications and treatment of perinatally HIV-infected children and adolescents. J Int AIDS Soc.

[233] Hadigan C, Meigs JB, Corcoran C, Rietschel P, Piecuch S, Basgoz N (2001). Metabolic abnormalities and cardiovascular disease risk factors in adults with human immunodeficiency virus infection and lipodystrophy. Clin Infect Dis.

[234] Friis-Moller N, Sabin CA, Weber R, d'Arminio Monforte A, El-Sadr WM, Reiss P (2003). Combination antiretroviral therapy and the risk of myocardial infarction. N Engl J Med.

[235] Stagi S, Bindi G, Galluzzi F, Galli L, Salti R, de Martino M (2004). Changed bone status in human immunodeficiency virus type 1 (HIV-1) perinatally infected children is related to low serum free IGF-I. Clin Endocrinol.

[236] Zamboni G, Antoniazzi F, Bertoldo F, Lauriola S, Antozzi L, Tato L (2003). Altered bone metabolism in children infected with human immunodeficiency virus. Acta Paediatr.

[237] Mora S, Sala N, Bricalli D, Zuin G, Chiumello G, Vigano A (2001). Bone mineral loss through increased bone turnover in HIV-infected children treated with highly active antiretroviral therapy. AIDS.

[238] Mora S, Zamproni I, Beccio S, Bianchi R, Giacomet V, Vigano A (2004). Longitudinal changes of bone mineral density and metabolism in antiretroviral-treated human immunodeficiency virus-infected children. J Clin Endocrinol Metab.

[239] Hazra R, Gafni RI, Maldarelli F, Balis FM, Tullio AN, DeCarlo E (2005). Tenofovir disoproxil fumarate and an optimized background regimen of antiretroviral agents as salvage therapy for pediatric HIV infection. Pediatrics.

[240] Gafni RI, Hazra R, Reynolds JC, Maldarelli F, Tullio AN, DeCarlo E (2006). Tenofovir disoproxil fumarate and an optimized background regimen of antiretroviral agents as salvage therapy: impact on bone mineral density in HIV-infected children. Pediatrics.

[241] Purdy JB, Gafni RI, Reynolds JC, Zeichner S, Hazra R (2008). Decreased bone mineral density with off-label use of tenofovir in children and adolescents infected with human immunodeficiency virus. J Pediatr.

[242] Giacomet V, Mora S, Martelli L, Merlo M, Sciannamblo M, Vigano A (2005). A 12-month treatment with tenofovir does not impair bone mineral accrual in HIV-infected children. J Acquir Immune Defic Syndr.

[243] Vigano A, Aldrovandi GM, Giacomet V, Merlo M, Martelli L, Beretta S (2005). Improvement in dyslipidaemia after switching stavudine to tenofovir and replacing protease inhibitors with efavirenz in HIV-infected children. Antivir Ther.

[244] Giacomet V, Nannini P, Vigano A, Erba P, Benincaso A, Bedogni G (2015). Long-term renal effects of tenofovir-disoproxil-fumarate in vertically HIV-infected children, adolescents, and young adults: a 132-month follow-up study. Clin Drug Investig.

[245] Gallant JE, Staszewski S, Pozniak AL, DeJesus E, Suleiman JM, Miller MD (2004). Efficacy and safety of tenofovir DF vs stavudine in combination therapy in antiretroviral-naive patients: a 3-year randomized trial. JAMA.

[246] Thomas V, Purdy JB, Reynolds JC, Hadigan C, Hazra R (2009). Bone mineral density in adolescents infected with HIV perinatally or childhood: data from the NIH Intramural Program.

[247] Puthanakit T, Siberry GK (2013). Bone health in children and adolescents with perinatal HIV infection. J Int AIDS Soc.

[248] Buve A, Jespers V, Crucitti T, Fichorova RN (2014). The vaginal microbiota and susceptibility to HIV. AIDS.

[249] Cone RA (2014). Vaginal microbiota and sexually transmitted infections that may influence transmission of cell-associated HIV. J Infect Dis.

[250] Venkatesh KK, van der Straten A, Mayer KH, Blanchard K, Ramjee G, Lurie MN (2011). African women recently infected with HIV-1 and HSV-2 have increased risk of acquiring Neisseria gonorrhoeae and Chlamydia trachomatis in the methods for improving reproductive health in Africa trial. Sex Transm Dis.

[251] Marschalko M, Ponyai K, Karpati S (2015). Sexually transmitted coinfections. HIV coinfections. Orv Hetil.

[252] Belongia EA, Danila RN, Angamuthu V, Hickman CD, DeBoer JM, MacDonald KL (1997). A population-based study of sexually transmitted disease incidence and risk factors in human immunodeficiency virus-infected people. Sex Transm Dis.

[253] Hankins C, Coutlee F, Lapointe N, Simard P, Tran T, Samson J (1999). Prevalence of risk factors associated with human papillomavirus infection in women living with HIV. Canadian Women's HIV Study Group. CMAJ.

[254] Maiman M, Fruchter RG, Serur E, Remy JC, Feuer G, Boyce J (1990). Human immunodeficiency virus infection and cervical neoplasia. Gynecol Oncol.

[255] Moscicki AB, Ellenberg JH, Vermund SH, Holland CA, Darragh T, Crowley-Nowick PA (2000). Prevalence of and risks for cervical human papillomavirus infection and squamous intraepithelial lesions in adolescent girls: impact of infection with human immunodeficiency virus. Arch Pediatr Adolesc Med.

[256] Mullins TL, Rudy BJ, Wilson CM, Sucharew H, Kahn JA (2013). Incidence of sexually transmitted infections in HIV-infected and HIV-uninfected adolescents in the USA. Int J STD AIDS.

[257] Brogly SB, Watts DH, Ylitalo N, Franco EL, Seage GR, Oleske J (2007). Reproductive health of adolescent girls perinatally infected with HIV. Am J Public Health.

[258] Agwu AL, Jang SS, Korthuis PT, Araneta MR, Gebo KA (2011). Pregnancy incidence and outcomes in vertically and behaviorally HIV-infected youth. JAMA.

[259] Jao J, Agwu A, Mhango G, Kim A, Park K, Posada R (2015). Growth patterns in the first year of life differ in infants born to perinatally vs. nonperinatally HIV-infected women. AIDS.

[260] Kennedy CE, Spaulding AB, Brickley DB, Almers L, Mirjahangir J, Packel L (2010). Linking sexual and reproductive health and HIV interventions: a systematic review. J Int AIDS Soc.

[261] Davis AM, Brown RF, Taylor JL, Epstein RA, McPheeters ML (2014). Transition care for children with special health care needs. Pediatrics.

[262] Dowshen N, D'Angelo L (2011). Health care transition for youth living with HIV/AIDS. Pediatrics.

[263] Mellins CA, Brackis-Cott E, Leu CS, Elkington KS, Dolezal C, Wiznia A (2009). Rates and types of psychiatric disorders in perinatally human immunodeficiency virus-infected youth and seroreverters. J Child Psychol Psychiatry.

[264] Gaughan DM, Hughes MD, Oleske JM, Malee K, Gore CA, Nachman S (2004). Psychiatric hospitalizations among children and youths with human immunodeficiency virus infection. Pediatrics.

[265] Scharko AM (2006). DSM psychiatric disorders in the context of pediatric HIV/AIDS. AIDS Care.

[266] Benton TD (2011). Psychiatric considerations in children and adolescents with HIV/AIDS. Pediatr Clin North Am.

[267] Mellins CA, Malee KM (2013). Understanding the mental health of youth living with perinatal HIV infection: lessons learned and current challenges. J Int AIDS Soc.

[268] Chernoff M, Nachman S, Williams P, Brouwers P, Heston J, Hodge J (2009). Mental health treatment patterns in perinatally HIV-infected youth and controls. Pediatrics.

[269] Gadow KD, Chernoff M, Williams PL, Brouwers P, Morse E, Heston J (2010). Co-occuring psychiatric symptoms in children perinatally infected with HIV and peer comparison sample. J Dev Behav Pediatr.

[270] Mellins CA, Elkington KS, Leu CS, Santamaria EK, Dolezal C, Wiznia A (2012). Prevalence and change in psychiatric disorders among perinatally HIV-infected and HIV-exposed youth. AIDS Care.

[271] Malee KM, Tassiopoulos K, Huo Y, Siberry G, Williams PL, Hazra R (2011). Mental health functioning among children and adolescents with perinatal HIV infection and perinatal HIV exposure. AIDS Care.

[272] Nachman S, Chernoff M, Williams P, Hodge J, Heston J, Gadow KD (2012). Human immunodeficiency virus disease severity, psychiatric symptoms, and functional outcomes in perinatally infected youth. Arch Pediatr Adolesc Med.

[273] Wood SM, Shah SS, Steenhoff AP, Rutstein RM (2009). The impact of AIDS diagnoses on long-term neurocognitive and psychiatric outcomes of surviving adolescents with perinatally acquired HIV. AIDS.

[274] Bomba M, Nacinovich R, Oggiano S, Cassani M, Baushi L, Bertulli C (2010). Poor health-related quality of life and abnormal psychosocial adjustment in Italian children with perinatal HIV infection receiving highly active antiretroviral treatment. AIDS Care.

[275] Gadow KD, Angelidou K, Chernoff M, Williams PL, Heston J, Hodge J (2012). Longitudinal study of emerging mental health concerns in youth perinatally infected with HIV and peer comparisons. J Dev Behav Pediatr.

[276] Elkington KS, Robbins RN, Bauermeister JA, Abrams EJ, McKay M, Mellins CA (2011). Mental health in youth infected with and affected by HIV: the role of caregiver HIV. J Pediatr Psychol.

[277] Mellins CA, Brackis-Cott E, Dolezal C, Abrams EJ (2006). Psychiatric disorders in youth with perinatally acquired human immunodeficiency virus infection. Pediatr Infect Dis J.

[278] Betancourt T, Scorza P, Kanyanganzi F, Fawzi MC, Sezibera V, Cyamatare F (2014). HIV and child mental health: a case–control study in Rwanda. Pediatrics.

[279] Zeegers I, Rabie H, Swanevelder S, Edson C, Cotton M, van Toorn R (2010). Attention deficit hyperactivity and oppositional defiance disorder in HIV-infected South African children. J Trop Pediatr.

[280] Lee B, Chhabra M, Oberdorfer P (2011). Depression among vertically HIV-infected adolescents in Northern Thailand. J Int Assoc Physicians AIDS Care.

[281] Havens JF, Mellins CA, Rutter M, Taylor E (2008). Psychiatric aspects of HIV/AIDS in childhood and adolescence. Child and adolescent psychiatry.

[282] Rutter M (1966). Children of sick parents.

[283] Dowdney L (2000). Childhood bereavement following parental death. J Child Psychol Psychiatry.

[284] Tremblay GC, Israel AC (1998). Children's adjustment to parental death. Clin Psychol.

[285] Mabugu T, Revill P, van den Berg B (2013). The methodological challenges for the estimation of quality of life in children for use in economic evaluation in low-income countries. Value Health Reg Issues.

[286] Stevens M, Kirsh B, Nixon SA (2014). Rehabilitation interventions for children living with HIV: a scoping review. Disab Rehabil.

[287] Worthington C, Myers T, O'Brien K, Nixon S, Cockerill R (2005). Rehabilitation in HIV/AIDS: development of an expanded conceptual framework. AIDS Patient Care STDs.

[288] Sohn AH, Hazra R (2013). The changing epidemiology of the global paediatric HIV epidemic: keeping track of perinatally HIV-infected adolescents. J Int AIDS Soc.

[289] Wettstein C, Mugglin C, Egger M, Blaser N, Vizcaya LS, Estill J (2012). Missed opportunities to prevent mother-to-child-transmission: systematic review and meta-analysis. AIDS.

[290] Ciaranello AL, Park JE, Ramirez-Avila L, Freedberg KA, Walensky RP, Leroy V (2011). Early infant HIV-1 diagnosis programs in resource-limited settings: opportunities for improved outcomes and more cost-effective interventions. BMC Med.

[291] Mugglin C, Wandeler G, Estill J, Egger M, Bender N, Davies MA (2013). Retention in care of HIV-infected children from HIV test to start of antiretroviral therapy: systematic review. PLoS One.

# Sub article: Achieving 90–90–90 in paediatric HIV: adolescence as the touchstone for transition success

**DOI:** 10.7448/IAS.18.7.20257

**Published:** 2015-12-02

**Authors:** Sonia Lee, Rohan Hazra

**Affiliations:** Eunice Kennedy Shriver National Institute on Child Health and Human Development, Maternal and Pediatric Infectious Disease Branch, Bethesda, MD, USA

**Keywords:** children, adolescents, HIV, transition, guidelines

## Abstract

**Introduction:**

The number of children less than 15 years estimated to be living with HIV globally approximated 3.2 million in 2013. Young people aged 15 to 24 years living with HIV approximated 4 million. The survival of these children and adolescents into adulthood poses new and urgent challenges of transition from the paediatric to adolescent to adult healthcare settings due to emerging developmental, psychosocial and comorbid issues. In order to achieve treatment targets of 90–90–90 across the continuum of care for paediatric HIV by 2020, focused efforts on the implementation of appropriate healthcare transition plans across the lifespan, with a focus on adolescence, should be prioritized.

**Discussion:**

Published data or empirical evidence examining implementation of transition models and association with clinical outcomes are limited. While some guidelines do exist that offer recommendations about how to promote seamless transitions, very few data are available to assess the adequacy of these guidelines and whether they are effectively adhered to in clinical care settings globally. Furthermore, paediatric and adolescent HIV infection, either acquired perinatally or behaviourally, is set apart from other chronic illnesses as a highly stigmatizing disease that disproportionately affects poor, minority and often marginalized populations. Focused efforts on adolescence as the touchstone for transition practices and policies need to be implemented.

**Conclusions:**

Optimal healthcare for these vulnerable populations, particularly in resource-limited settings, will require HIV-specific transitional care services and programmes that are coordinated, collaborative, integrated and, importantly, evidence-based.

## Introduction

Survival of perinatally HIV-infected (PHIV) children into youth, and continued survival of PHIV youth and behaviourally HIV-infected youth into adulthood, poses new challenges of transitions from the paediatric and adolescent healthcare setting to the adult healthcare setting. These transitions across the early lifespan encompass a dynamic developmental stage, namely adolescence, in which youth are establishing their identity and autonomy, mastering abstract thought, negotiating independent decision-making, managing educational and employment challenges and having intimate relationships [1]. For these HIV-positive youth, this complex developmental stage also occurs in the context of their HIV infection and often economic, social and familial stress. Additional issues such as disclosure of HIV illness and stigma also need to be navigated. Therefore, optimal healthcare for these children and youth must consider healthcare transition as a continuum and not separate, discrete moves from paediatric to adolescent to adult clinic settings. Such consideration will include the development of a formal plan among the child, family and medical providers with a focus on the adolescence transition period.

It is estimated that in the United States over 90% of children with chronic conditions will survive beyond their twenties [2]. Due to medical advances and the increased availability of antiretroviral medications, the overall number of children and youth with HIV surviving into adulthood is increasing. The reduction in AIDS mortalities in infants in South Africa has shifted the burden of paediatric HIV to older children with a growing proportion of AIDS deaths in older children [3]. Globally, improved care and treatment options have increased the lifespan of people living with HIV, with rapid decreases in AIDS-related deaths between 2001 and 2013 for all age groups, with one notable exception – adolescents (aged 10 to 19 years) [4]. In response, the 90–90–90 targets, developed by UNAIDS and partners for all people living with HIV, have been adopted: by 2020, 90% of all people living with HIV will know their HIV status; 90% of all people with diagnosed HIV infection will receive sustained antiretroviral therapy (ART); 90% of all people receiving ART will have viral suppression [5]. The 90–90–90 treatment goals must include children and adolescents. They can only be achieved if appropriate transition plans are methodically incorporated into the healthcare of children and youth with HIV.

Over the past 20 years the Society for Adolescent Medicine [6,7], the US Department of Health and Human Services [8], the American Academy of Pediatrics [9] and others [10] have made efforts to promulgate the critical need to study and evaluate transition processes. Despite these efforts, transitional care services and programmes that are coordinated, collaborative, integrated and, most importantly, evidence-based are lacking [11]. Indeed, the immense burden of transitioning patients across various healthcare clinics needs to be removed from medical care providers and even the patients themselves. Instead, the HIV community of researchers, policy-makers and broader healthcare service programme advocates need to take the lead. The effectiveness of integrating implementation science research efforts with current policies, guidelines and recommendations for effective transitioning needs to be assessed. The number of children with HIV ageing into adolescents, in addition to the increasing number of new HIV infections in adolescents, is developing into an enormous burden on current healthcare services and programmes worldwide [12,13]. Indeed, disparities and trends in AIDS mortality among adolescents with HIV in low- and middle-income countries call for immediate action [14]. It is imperative to conduct research studies to evaluate not only the transition process, but also the outcomes of transition, including the 90–90–90 targets, in order to implement findings and determine the optimal healthcare approach to manage this burgeoning change in the landscape of paediatric HIV.

## Discussion

### Emerging health challenges

Paediatric HIV poses a specialized challenge from other chronic illnesses in that treatment, and thus transition, plans should be mindful of emerging health challenges such as cardiovascular disease risk and bone health [15–18]. In addition, biomedical comorbidities represent a particularly important and relevant concern to manage over the course of HIV infection and thus to address in any transition plan concerns psychiatric illnesses. Children and youth with HIV are at increased risk for psychiatric hospitalizations, compared with the general paediatric population [19]. A systematic review of patients with HIV infection and psychiatric diagnoses and symptoms also found an increased risk of psychiatric illness among HIV-positive individuals than among the general population [20]. With improved survival among children and youth with HIV, the manifestations of neuropsychiatric complications during HIV disease, including HIV-associated neurocognitive disorders, and the use of psychotropic medications in conjunction with antiretroviral treatments, are additional outcomes to screen for and manage in an effective and adaptable treatment and transition plan.

### Emphasis on adolescent-friendly services

Adolescents who acquired HIV infection horizontally through risk behaviours (such as consensual or non-consensual sex and injection drug use) are a distinct population from adolescents with perinatally acquired HIV. However, the many challenges of moving to adult healthcare, including disclosure of HIV status to others and emerging independence in managing a chronic illness, will need to be integrated into a purposeful and planned transition. Adolescence poses a developmental and high-risk period where physical, emotional and social stressors may impact how youth cope with their HIV and manage their treatment. Other factors for adolescents to tackle during this time include stigma, feelings of loss and death and lack of social support [21]. Overall, adolescents are significantly underserved by HIV services and thus have poor access to treatment options and lower adherence to ART and medical appointments than adults [22]. Indeed, the HIV cascade of care from HIV diagnosis to viral suppression among adolescents and young adults indicates larger declines at all steps than older adults with HIV [23]. Therefore, it is important for transition plans to bridge these gaps by adapting and tailoring HIV services to retain adolescents in care, improve adherence to ART and manage the developmental issues in parallel to their chronic illness. A comprehensive approach to transition to assist healthcare providers as well as children, adolescents and their families will ensure a seamless and successful integration into a new, adult care setting. Complexities inherent with successful transition outcomes and the ultimate goal of viral suppression become most apparent for adolescents with HIV, including awareness that many paediatric, adolescent (if available) and adult HIV care models have fundamental differences [24]. For example, most adolescent HIV clinics are multidisciplinary and cater to more than HIV treatment with an integrated approach to address sexual/reproductive health, mental health-related issues and adherence counselling. On the other hand, many adult clinics may prefer to refer the young adults to separate subspecialty care settings. This change in medical care provision practice places additional burdens on the newly transitioned young adult to take ownership of and integrate their care, practice autonomy and independent decision-making and provide informed consent for new procedures and doctors. The pressure for young adults to independently navigate a fractionated healthcare system may potentially increase the lack of treatment engagement altogether [25]. As such, transition plans need to fill in the treatment gaps along the continuum of care trajectory by specifically targeting and focusing on adolescence by integrating “adolescent-friendly” healthcare services into a comprehensive care model [26]. This step is particularly important for those practices that directly transition patients from a paediatric care setting to an adult care facility, without separate consideration for adolescent-specific services. To date, too much focus for transition success has been placed on establishing guidelines for medical providers and for the patients themselves. Instead, the focus needs to shift on implementation science research efforts as the context of HIV care varies incredibly due to resources, healthcare provision and policies and geographical settings.

### Striving for healthy outcomes

In addition to encompassing the complex developmental, psychosocial and medical issues facing children and adolescents with HIV as they transition to adulthood, a comprehensive care transition programme should be guided by further research on the predictors of healthy outcomes. Especially with respect to strategies for achieving the 90–90–90 treatment targets, more needs to be known regarding how these vulnerable HIV populations fare with respect to their health, physical and mental, in adulthood. Unfortunately, preliminary findings point to the transitions from paediatric to adult care as yet another risk factor for poor clinical outcomes [27]. A study conducted in England highlights poor consequences for young people with perinatally acquired HIV infections following their transfer to adult care [28]. In this study, the estimated minimum mortality rate for those PHIV patients aged greater than or equal to 21 years in adult care was nearly five times greater than those PHIV patients aged 13 to 15 years in paediatric care. Better methods for continued surveillance of PHIV populations to reliably and systematically track outcomes will greatly inform the current guidelines and recommendations for transition to adult care settings [29–31]. In addition, published data and/or empirical evidence examining implementation of transition models and association with clinical outcomes are critical. Despite previous recommendations for this type of research, published data on healthcare transition outcomes for HIV populations are limited and, thus, evidence-based transition recommendations are currently lacking.

A crucial research area to improve transition models encompasses the voices of youth actively going through the transition process as well as young adults who have just experienced the transition process. Qualitative interview responses, demographic information and physical health status outcomes were collected from a study to explore the experiences of 42 recently transitioned youth (mean age 22 years) [32]. Unfortunately, the health outcomes (measured by CD4 counts) trended in the downward direction with 45% reporting a more difficult transition process than anticipated. Several barriers to successful transition were cited: economic, logistical, lack of communication between paediatric and adult care providers, lag in developmental readiness, difficult access to psychosocial services and even reluctance by paediatric providers to transition their patients who had been under their care for most of their lives. Separate qualitative studies conducted with small samples of transitioned young adults with behaviourally acquired HIV (ages 24 to 29 years) emphasized how they felt unprepared for transition, resulting in increased anxiety about expectations for their future [33–35]. Important concepts of readiness [36–38] and stigma [20] should also be continually assessed prior to and during the transition process. Additional needs for successful transition plans should also include an assessment of the young adult's educational aspirations, with appropriate vocational and life skills training. As the prognosis for children and adolescents with HIV has changed, paediatric care providers will need to work with adult care providers to better prepare their patients for independent and healthy living.

### Integrated management of illness

With HIV as the second leading cause of death among adolescents in 2012 [39], international efforts to improve surveillance of the global adolescent health epidemiology are needed, as are efforts to define public health priorities for adolescents. Indeed, as many adolescent health problems start from childhood and continue across the life course into early adulthood, transition programmes need to emphasize the impact of developmental processes on the care continuum. Previously mentioned guidelines and recommendations for transition have emanated from the United States and tend to focus on medical providers. Instead, their applicability to international settings most affected by HIV needs to be carefully evaluated and the contexts under which transition occurs (or does not successfully occur) need to be considered. An integrated management of adult, adolescent and childhood illness [40] has been developed to support a public-health approach in resource-limited, developing countries, where the HIV pandemic is more severe. This poses a different model from the tailored approaches recommended in the United States, where resources to modify healthcare services are more readily available. Since 2001, the WHO has promoted a conceptual shift from an individual-based to a population-based approach to ensure access to ART treatment, outside the specialized management approach of high-resource settings [41]. For example, the Thai HIV programme provides free, comprehensive HIV treatment for children, delivered by their Ministry of Public Health through a holistic model, including clinical and psychosocial care; this model is evolving to encompass the entire care continuum from infection with HIV through the transition to adult services [42]. Individual-based approaches to HIV care pose challenges, including the need for numerous providers with separate, specialized expertise. One unique and important aspect of this public health approach to HIV is through the integration of volunteer “co-providers” at clinics and in the community as support systems for patients across developmental stages. Further evaluations and evidence for the success of a public-health approach to scale up HIV services and facilitate access to ART are becoming more widespread. Parallel efforts should also be implemented globally to address the evolving needs of those living with HIV and to ensure the smooth transition of children to adolescents to adults with simple, standardized, decentralized and equitable healthcare.

## Conclusions

While some guidelines do exist to offer recommendations about how to manage the transitions across different healthcare settings, very few data are available to assess the adequacy of these guidelines and whether they are routinely implemented in clinical care settings, both domestically and globally. In order to attain the 2020 targets of 90% of all people living with HIV aware of their HIV status, 90% of all people diagnosed with HIV on sustained ART and 90% of all people living with HIV achieving viral suppression, HIV research priorities need to emphasize empirical investigations of healthcare setting transition plans (see Table 1).

**Table 1:** Future priorities for transition research and practice

•	Evidence-based transition models specifically for HIV populations, with a focus on adolescent development care and needs
•	Longitudinal studies, including registries and/or cohorts of children and youth with HIV to effectively track and monitor physical and mental health outcomes across the lifespan
•	Adolescent-friendly HIV healthcare services with comprehensive care to incorporate emerging co-morbidities
•	Broader health system reform and policies to approach HIV care from a dynamic, developmental perspective and to consider social and contextual factors such as stigma, resources and access

Focused transition efforts within the field of HIV have the potential to address the broader issues of chronic illness during adolescence, especially in low- and middle-income countries, where adolescent health services are quite limited. Optimally, with adequate preparation, children and youth with HIV will have appropriately transitioned to adult care upon successful implementation of a well-prepared plan, developed over the entire life course of their HIV illness. They will feel clinically and psychosocially able to manage their HIV illness, seek and attend appropriate medical appointments and will become goal-oriented toward healthy and long-term living. Targeting the developmental period of adolescence for vulnerable populations of children with HIV and focusing our efforts of improving HIV care services [43], in conjunction with initiating an overarching umbrella of broader health system reform and policies [17], will facilitate transitioning children to adolescents, and adolescents to adults.

## References

[1] http://www.cdc.gov/ncbddd/childdevelopment/positiveparenting/adolescence.html.

[2] Scal P, Evans T, Blozis S, Okinow N, Bloom R (1999). Trends in transition from pediatric to adult health care services for young adults with chronic conditions. J Adolesc Health.

[3] Johnson LF, Davies MA, Moultrie H, Harry S, Gayle G, Bland RM (2012). The effect of early initiation of antiretroviral treatment in infants on pediatric AIDS mortality in South Africa. Pediatr Infect Dis J.

[4] United Nations Children's Fund (UNICEF) (2015). Progress for Children: Beyond Averages. Learning from the MDGS. Number 11.

[5] Joint United Nations Programme on HIV/AIDS (UNAIDS) (2014). 90-90-90 An ambitious treatment target to help end the AIDS epidemic.

[6] Blum RW, Garell D, Hodgman CH, Jorissen TW, Okinow NA, Orr DP (1993). Transition from child-centered to adult health-care systems for adolescents with chronic conditions. J Adolesc Health.

[7] Rosen DS, Blum RWM, Britto M, Sawyer SM, Siegel DM, Society for Adolescent Medicine (2003). Transition from child-centered to adult health-care systems for adolescents with chronic conditions: a position paper of the Society for Adolescent Medicine. J Adolesc Health.

[8] Panel on Antiretroviral Guidelines for Adults and Adolescents Guidelines for the use of antiretroviral agents in HIV-1-infected adults and adolescents. Department of Health and Human Services [Internet].

[9] American Academy of Pediatrics Policy Statement (2013). Transitioning HIV-infected youth into adult health care. Pediatrics.

[10] Cervia JS (2013). Easing the transition of HIV-infected adolescents to adult care. AIDS Patient Care STDs.

[11] Andiman WA (2011). Transition from pediatric to adult healthcare services for young adults with chronic illnesses: the special case of human immunodeficiency virus infection. J Pediatr.

[12] Idele P, Gillespie A, Porth T, Suzuki C, Mahy M, Kasedde S (2014). Epidemiology of HIV and AIDS among adolescents: current status, inequities, and data gaps. J Acquir Immune Defic Syndr.

[13] Lowenthal ED, Bakeera-Kitaka S, Marukutira T, Chapman J, Goldrath K, Ferrand RA (2014). Perinatally acquired HIV infection in adolescents from sub-Saharan Africa: a review of emerging challenges. Lancet.

[14] Porth T, Suzuki C, Gillespie A, Kasedde S, Idele P (2014). Disparities and trends in AIDS mortality among adolescents living with HIV in low- and middle-income countries.

[15] Jacobson DL, Williams P, Tassiopoulus K, Melvin A, Hazra R, Farley J (2011). Clinical management and follow-up of hypercholesterolemia among perinatally HIV-infected children enrolled in the PACTG 219C Study. J Acquir Immune Defic Syndr.

[16] Carter RJ, Wiener J, Abrams EJ, Farley J, Nesheim S, Palumbo P (2006). Dyslipidemia among perinatally HIV-infected children enrolled in the PACTS-HOPE cohort, 1999–2004: a longitudinal analyses. J Acquir Immune Defic Syndr.

[17] Patel K, Wang J, Jaconson DL, Lipshultz SE, Landy DC, Geffner ME (2014). Aggregate risk of cardiovascular disease among adolescents perinatally infected with the human immunodeficiency virus. Circulation.

[18] Dimeglio LA, Wang J, Siberry GK, Miller TL, Geffner ME, Hazra R (2013). Bone mineral density in children and adolescents with perinatal HIV infection. AIDS.

[19] Gaughan DM, Hughes MD, Oleske JM, Malee K, Gore CA, Nachman S (2004). Psychiatric hospitalizations among children and youths with human immunodeficiency virus infection. Pediatrics.

[20] Owe-Larsson B, Sall L, Salamon E, Allqulander C (2009). HIV infection and psychiatric illness. Afr J Psychiatry.

[21] http://apps.who.int/adolescent/hiv-testing-treatment/page/Psychosocial_well_being.

[22] World Health Organization (2014). Report of the consultation on the treatment of HIV among adolescents. Meeting Report.

[23] Zanoni BC, Mayer KH (2014). The adolescent and young adult HIV cascade of care in the United States: exaggerated health disparities. AIDS Patient Care STDs.

[24] Tanner AE, Philbin MM, Duval A, Ellen J, Kapogiannis B, Fortenberry JD (2014). “Youth friendly” clinics: considerations for linking and engaging HIV-infected adolescents into care. AIDS Care.

[25] Agwu A, Lee L, Fleishman JA, Voss C, Yehia BR, Althoff KN (2015). Aging and loss to follow-up among youth living with human immunodeficiency virus in the HIV Research Network. J Adolesc Health.

[26] Bertrand R, Reif L, Pape JW (2014). Impact of implementing “adolescent-focused” services in Haiti.

[27] Hepburn CM, Cohen E, Bhawra J, Weiser N, Hayeems RZ, Guttmann A (2015). Health system strategies supporting transition to adult care. Arch Dis Child.

[28] Fish R, Judd A, Jungmann E, O'Leary E, Foster C, HIV Young Persons Network (HYPNet) (2014). Mortality in perinatally HIV-infected young people in England following transition to adult care: an HIV Young Persons Network (HYPNet) audit. HIV Med.

[29] Foster C, Judd A, Tookey P, Tudor-Williams G, Dunn D, Shingadia D (2009). Young people in the United Kingdom and Ireland with perinatally acquired HIV: the pediatric legacy for adult services. AIDS Patient Care STDS.

[30] https://clinicaltrials.gov/ct2/show/NCT01269632.

[31] https://phacsstudy.org/About-Us/Active-Studies.

[32] Wiener LS, Kohrt BA, Battles HB, Pao M (2011). The HIV experience: youth identified barriers for transitioning from pediatric to adult care. J Pediatr Psychol.

[33] Valenzuela JM, Buchanan CL, Radcliffe J, Ambrose C, Hawkins LA, Tanney M (2011). Transition to adult services among behaviorally infected adolescents with HIV – a qualitative study. J Pediatr Psychol.

[34] Katusiime C, Parkes-Ratanshi R, Kambugu A (2013). Transitioning behaviourally infected HIV-positive young people into adult care: experiences from the young person's point of view. S Afr J HIV Med.

[35] Bundock H, Fidler S, Clarke S, Holmes-Walker DJ, Farrell K, McDonald S (2011). Crossing the divide: transition care services for young people with HIV – their views. AIDS Patient Care STDS.

[36] Wiener L, Zobel M, Battles H, Ryder C (2007). Transition from a pediatric HIV intramural clinical research program to adolescent and adult community-based care services: assessing transition readiness. Soc Work Health Care.

[37] Schwartz LA, Tuchman LK, Hobbie WL, Ginsberg JP (2011). A socio-ecological model of readiness for transition to adult-oriented care for adolescents and young adults with chronic health conditions. Child Care Health Dev.

[38] New York State Department of Health AIDS Institute (2012). Transitioning HIV-Infected adolescents into adult care.

[39] http://www.who.int/maternal_child_adolescent/epidemiology/adolescence/en/.

[40] World Health Organization (2007). Chronic HIV care with ART and prevention: Integrated Management of Adolescent and Adult Illness, Integrated Management of Childhood Illness Interim Guidelines for health workers at health centre or district hospital outpatient clinic.

[41] Gilks CF, Crowley S, Ekpini R, Gove S, Perriens J, Souteyrand Y (2006). The WHO public-health approach to antiretroviral treatment against HIV in resource-limited settings. Lancet.

[42] Tullock O, Theobald S, Ananworanich J, Chasombat S, Kosalaraksa P, Jirawattanapisal T (2014). From transmission to transition: lessons learnt from the Thai Paediatric Antiretroviral Programme. PLOS One.

[43] Bekker L-G, Hosek S (2015). HIV and adolescents: focus on young key populations. J Int AIDS Soc.

# Sub article: Social protection: potential for improving HIV outcomes among adolescents

**DOI:** 10.7448/IAS.18.7.20260

**Published:** 2015-12-02

**Authors:** Lucie D Cluver, Rebecca J Hodes, Lorraine Sherr, F Mark Orkin, Franziska Meinck, Patricia Lim Ah Ken, Natalia E Winder-Rossi, Jason Wolfe, Marissa Vicari

**Affiliations:** 1 Centre for Evidence-Based Intervention, Department of Social Policy & Intervention, University of Oxford, Oxford, UK; 2 Department of Psychiatry and Mental Health, University of Cape Town, Cape Town, South Africa; 3 AIDS and Society Research Unit, Centre for Social Science Research, University of Cape Town, Cape Town, South Africa; 4 Department of Historical Studies, University of Cape Town, Cape Town, South Africa; 5 Health Psychology Unit, Department of Infection & Population Health, University College London, London, UK; 6 School of Clinical Medicine and DST-NRF Centre of Excellence in Human Development, University of the Witwatersrand, Johannesburg, South Africa; 7 HIV and AIDS Section, UNICEF, New York, USA; 8 UNICEF Regional Office for Eastern and Southern Africa, Nairobi, Kenya; 9 Office of HIV/AIDS, Bureau for Global Health, US Agency for International Development, Washington, DC, USA; 10 Collaborative Initiative for Paediatric HIV Education and Research (CIPHER), International AIDS Society, Geneva, Switzerland

**Keywords:** social protection, HIV/AIDS, adolescents, HIV prevention, adherence

## Abstract

**Introduction:**

Advances in biomedical technologies provide potential for adolescent HIV prevention and HIV-positive survival. The UNAIDS 90–90–90 treatment targets provide a new roadmap for ending the HIV epidemic, principally through antiretroviral treatment, HIV testing and viral suppression among people with HIV. However, while imperative, HIV treatment and testing will not be sufficient to address the epidemic among adolescents in Southern and Eastern Africa. In particular, use of condoms and adherence to antiretroviral therapy (ART) remain haphazard, with evidence that social and structural deprivation is negatively impacting adolescents’ capacity to protect themselves and others. This paper examines the evidence for and potential of interventions addressing these structural deprivations.

**Discussion:**

New evidence is emerging around social protection interventions, including cash transfers, parenting support and educational support (“cash, care and classroom”). These interventions have the potential to reduce the social and economic drivers of HIV risk, improve utilization of prevention technologies and improve adherence to ART for adolescent populations in the hyper-endemic settings of Southern and Eastern Africa. Studies show that the integration of social and economic interventions has high acceptability and reach and that it holds powerful potential for improved HIV, health and development outcomes.

**Conclusions:**

Social protection is a largely untapped means of reducing HIV-risk behaviours and increasing uptake of and adherence to biomedical prevention and treatment technologies. There is now sufficient evidence to include social protection programming as a key strategy not only to mitigate the negative impacts of the HIV epidemic among families, but also to contribute to HIV prevention among adolescents and potentially to remove social and economic barriers to accessing treatment. We urge a further research and programming agenda: to actively combine programmes that increase availability of biomedical solutions with social protection policies that can boost their utilization.

## Introduction

Adolescent HIV support has misfired throughout the epidemic, caught in a wrinkle of under-provision and misunderstanding, with avoidable HIV infection and death as the result. Antiretroviral therapy (ART) and condoms provide the means for adolescents to live with HIV and to prevent transmission. However even where these technologies are available, high rates of HIV infection, morbidity and mortality persist, particularly in Southern and Eastern Africa. HIV infections among adolescents in the region remain at 440 per day [1] with approximately 2.1 million adolescents (aged 10 to 19) living with HIV, the majority in Southern Africa [2]. Worldwide, adolescents are the only age group in which AIDS-related deaths are not decreasing [1].

Why is this? Evidence shows that technologies, despite their efficacy, are not being accessed or used consistently. Biomedical responses are imperative but not sufficient, and they remain obstructed by weak procurement and supply management systems, human resources shortages and the high cost of particular treatment regimens and diagnostics [3, p. 10]. The UNAIDS 90–90–90 commitment highlights the importance of achieving better coverage of HIV testing, ART initiation and retention in effective care. However, its focus is principally on the provision of medical technologies within the health sector, rather than on the expansion of other services and interventions from within the overlapping spheres of social development and education [3]. Similar efforts and commitments related to increasing access to and uptake of prevention services among adolescents are needed if the goals of HIV prevention and treatment programs are to be achieved [3].

Reported rates of girls’ condom use at last high-risk sex was less than one-third in nine countries in Southern and Eastern Africa [4] and even lower in poor households and rural areas [5]. ART adherence also remains a major challenge: in a nine-country regional study, adolescent adherence was between 7 and 20% [6,7]. A recent trial of pre-exposure prophylaxis (PrEP) in Zimbabwe, South Africa and Uganda was halted early due to adherence rates of 25 to 30%, which were even lower among younger, single women [8].

Consistent condom and PrEP use and ART adherence are global challenges. For adolescents in resource-limited settings, individual barriers are exacerbated by socio-economic, environmental and structural constraints. Adolescence is a period of emotional-social development, growing independence and changing relationships with families, peers and romantic partners [9]. Associations between HIV infection, poverty and inequality are complex [10–12], but a growing body of research has established that social and structural deprivation, often with gendered aspects, are key drivers of adolescent HIV infection and mortality [13]. These deprivations include poverty and exclusion [14], income shocks [15,16], mental health distress, stigma [17], harsh parenting and abuse [18,19]. Exposure to multiple stressors can have cumulative effects, maximizing HIV-related risks [19]. In addition, HIV risk behaviours are primarily extra-clinical, occurring in the social spaces beyond the health system where adolescents live, have fun and take risks. HIV prevention programmes that focus on individual behaviours but do not account for their socio-ecological basis are likely to have limited efficacy [20]. This paper examines a set of interventions that aim to address socio-economic vulnerabilities among young people, assessing the quality and extent of the evidence base for social protection in contributing to our goals of reaching the 90–90–90 targets.

## Discussion

Interventions that function beyond the clinic, incorporating broader populations than patients alone, have begun to capture the attention of policymakers [21,22]. There is increasing interest in social protection as a potential intervention to improve HIV prevention and treatment outcomes in adolescence, by ameliorating the socio-economic deprivations that increase risks. Moreover, interventions that combine clinical and social care may have wider benefits for populations beyond HIV effects [23].

### Social protection and adolescent HIV prevention

*Social protection* is often understood as the transfer of cash to poor and vulnerable populations [24], but it is conceptually more expansive. UNICEF's definition for social protection incorporates “the set of public and private policies and programmes aimed at preventing, reducing and eliminating economic and social vulnerabilities to poverty and deprivation.” [25]. This can include a range of provisions. For example Devereux operationalizes social protection as emergency food aid, public works projects and agricultural subsidies [26]; de Haan highlights non-contributory schemes targeting chronic poverty [27]; and Haushofer notes increasing interest in health insurance and savings support [28]. Social protection programmes can include economic, social and psychosocial provisions administered by governments, NGOs or communities, or combinations of these modalities.

A new body of evidence shows that social protection can reduce risks of HIV infection in sub-Saharan Africa. While extensive evidence from Latin America and Africa links social protection to wider facets of wellbeing [28], research with HIV outcomes has largely focused on specific social protections of cash transfers, food provision and psychosocial care. In a randomized trial in Malawi, both unconditional and educational-conditioned cash transfers reduced HIV prevalence among girls [29]. A cluster randomized trial in Kenya and two propensity-matched studies in South Africa (one on a national cross-sectional dataset and another on a two-province longitudinal study, *n*=3500) all demonstrated associations between national unconditional cash transfer programmes and reduced HIV-infection risks among adolescents, particularly girls. These include reductions in sexual debut, pregnancy, age-disparate sex and transactional sex [30–32].

In the next year, further findings will emerge from the Transfer Project's [33] cluster randomized trials of national social cash transfers in Malawi, Zambia and Zimbabwe. Critiques of this research note that some findings are limited by a lack of HIV-incidence biomarkers, instead using proxies of self-reported behaviour, pregnancy and HIV prevalence. The evidence from South Africa uses propensity score matching rather than randomized trials (due to existing national cash transfer programmes) and there are ongoing debates concerning whether we should prioritize the real-world applicability of testing impacts of existing programmes or the causal reliability of randomized trials.

Research shows that cash transfers are not the only form of social protection with HIV-prevention effects. In South Africa, the combination of social welfare grants or school feeding with positive parenting or teacher support (“cash plus care”) was shown as more effective than cash transfers alone in reducing adolescent risk behaviour (see Figure 1) [34].

**Figure 1:** Impacts of cash and care provision on HIV-risk behaviour among adolescents in South Africa (marginal effects models, controlling for covariates) [34].

Indeed, new data demonstrate that *combination social protection* (providing specific combinations of cash transfers, school feeding, free schools, parental supervision and teacher support) shows cumulative risk-reduction effects among adolescents [35]. Such combinations appear to be most effective among the most difficult to reach groups. In Uganda, a combination programme of economic support (matched savings), educational support and mentoring improved mental health and educational attainment and reduced sexual risk-taking intentions among younger children [36,37]. Indeed, the strong prevention effects of keeping adolescents in school and of providing free schooling, school feeding and supportive teachers [38] suggest that social protection for adolescents should be expanded beyond cash plus care to include “cash, care and classroom.”

### Social protection and adolescent treatment

HIV-positive adolescents include two co-existing groups with shared and unique risks [39]. Survivors of vertical infection are growing up with lifelong exposure to HIV, high risk of cognitive impairment [40] and parenting environments often characterized by illness and death. Adolescents who acquire HIV through sexual transmission may differ in behavioural profiles, but both groups are strongly influenced by family-level and structural factors [41]. Recent systematic reviews [7,42] identify risk factors for ART non-adherence among adolescents, including social and structural deprivations of poverty, disorganized families, caregiver–child conflict and mental health problems [43]. A recent situation analysis in 23 sub-Saharan African countries found that socio-economic challenges including transportation costs and food insecurity were the greatest barriers to adolescent treatment and care [44]. Research has identified protective impacts of early disclosure to adolescents of their HIV status [45], but overall there is very limited research on how to improve adolescent adherence.

Evidence from the region suggests that social protection has the potential to be an effective intervention for HIV-positive adolescents and thus could contribute directly to achieving the UNAIDS 90–90–90 commitments. In South Africa, adolescent ART adherence was improved by social protection interventions of school feeding programmes, parental monitoring and social support [2]. A study of cash transfers for clinic transportation among adults in Uganda showed improved adherence and retention in care [46]. Several studies of mentorship mother programmes have shown improved PMTCT adherence [47–50], suggesting both a potential impact for adolescent mothers and prevention of child infection. In Tanzania, an ongoing study among adults is testing whether combinations of nutrition counselling, food assistance and unconditional cash transfers can promote ART adherence [51]. In Uganda an adolescent-focused randomized trial is currently testing effects on adherence of family savings with evidence-based family-support programming [52]. The recent WHO consultation on the upcoming 2015 treatment guideline update identified social protection (including food vouchers) as a key priority area for adolescents [53].

### Clearing confusion: cash incentives, conditional cash transfers and social protection

Cash incentive programmes are often conflated with social protection, but some differ fundamentally in their conceptual approach. Unconditional social protection premises that sexual decisions in low-resource contexts such as sub-Saharan Africa are constrained by structural deprivations such as poverty and violence, which need to be mitigated to allow healthy decision-making. In contrast, cash incentives premise that adolescent HIV risks are driven primarily by behavioural choice and can therefore be changed by provision of alternative (cash) rewards [54]. Pettifor distinguishes cash incentive programmes that are dependent on specific sexual behavioural adaptations as “downstream programmes” [55], tethered to a more individualist orientation for health promotion.

The evidence from trials of cash incentives is mixed. No studies have examined cash incentives among adolescents under the age of 18. No known studies have examined cash incentives for ART adherence in adolescents. In Tanzania among adults aged 18 to 30, a high cash amount (but not a lower amount), which was conditional on negative results of quarterly testing for sexually transmitted infections, reduced sexually transmitted infection (STI) prevalence compared to no-cash controls [54]. In Lesotho, a lottery-based system for 18 to 32 year olds, also based on negative results of STI testing, showed reductions in HIV incidence, but there remain questions about introducing gambling as a behavioural mechanism. In the United States, studies with adult drug-using HIV-positive populations have shown improvements [56] but a recent RCT showed no effects [57]. All evidence to date on cash incentives is from researcher-run programmes in randomized trials. Such trials provide greater confidence in causal relationships but may represent proof of concept rather than scalability and require further effectiveness testing as well as testing with adolescent populations.

A major concern with incentives is the complexity and costs associated with implementation, policing conditions and applying sanctions, which have been questioned as potentially unfeasible in resource-constrained countries with limited administrative capacity. In addition, critics raise ethical concerns about removing sources of income from poor adolescents in periods of acute vulnerability, such as HIV diagnosis. There are further questions about the validity of assumptions that STI infection represents a behavioural choice, when evidence shows high rates of rape and coerced sex among adolescent girls [58,59].

Somewhere between the two approaches of social protection and cash incentives is a third option of conditional cash transfers, focused on incentivizing behaviour to utilize services that will reduce structural drivers of HIV risk [55]. This approach is based on an understanding that the cash will serve to ensure the adoption of well-established beneficial behaviours such as school attendance [38]. Such cash transfers have been used for non-HIV behaviour change in national programmes in Latin America. These are based on robust evidence that schooling is protective against HIV infection, particularly for girls [60]. Among adults in Kenya, incentives for voluntary counselling and testing showed increased uptake [61]. New findings from two randomized trials in South Africa were reported in July 2015, both with promising findings. An individually randomized trial of cash transfers conditional on girls’ school attendance (ages 13 to 20) found reduced self-reported HIV-risk behaviours [62]. A cluster randomized trial of cash transfers for adolescents in early high school (grades 9 to 10, ages around 15 to 17), conditional on a range of behaviours including attendance at extracurricular activities, school exam success and HIV testing, showed reductions in HSV-2 incidence [63]. However, both trials had lower than expected overall HIV-incidence rates in the adolescent age group and may have had power thus reduced for this biomarker, reflecting challenges of testing HIV incidence as a primary outcome for adolescent programmes.

It is important to consider supply issues if conditionalities were to be taken to scale. In Tanzania, schooling conditions for secondary-age adolescents had to be removed due to insufficient schools to absorb the increased demand. These programmes show potential, but we also need to assess outcomes where conditionality may remove support from those at greatest risk for HIV transmission as well as for non-compliance to conditions. Further research is required to determine whether conditionality improves outcomes in comparison to unconditional cash transfers. Only one study date compares them: in Malawi, cash transfers reduced HIV prevalence and HSV prevalence among girls, but showed no differences between unconditional and conditional transfers [29].

### Is social protection affordable, acceptable and scalable?

The introduction of social grant systems has risen dramatically in Southern and Eastern Africa. Today, most countries in the region have implemented social protection programmes, although many remain small-scale. South Africa leads this trend, with over 16,640,000 grants disbursed monthly as of March 2015 – an economic lifeline for many [64]. The new DREAMS partnership, an initiative of PEPFAR, the Gates Foundation and the Nike Foundation, aims to reduce HIV infections among adolescent girls and young women in the region. Based on evidence synthesis, DREAMS includes a “combination prevention” package of biomedical and behavioural interventions, such as condom provision, HIV testing and PrEP in selected contexts, together with social protection interventions such as parenting programmes, school-based violence prevention, cash transfers and educational subsidies [65]. UNAIDS has identified social protection as a critical enabler in HIV prevention and is developing tools to assess country-level social protection programming and its potential for HIV synergies. There is remarkable potential for research, sustainability and expansion of social protection programmes.

However is social protection scalable and sustainable in the region? Seekings identifies that barriers to national social protection programmes are often neither administrative nor fiscal: both the ILO and World Bank show that most African countries can afford to expand their social protection floors [66]. Instead, barriers are often social and political attitudes, based on mutable perceptions of who is deserving of support [67]. The views of political elites, electorates, advocacy groups and international agencies may all have influence [66], although studies show high levels of acceptability at a population level [68]. Thus, programmes must intersect with the needs of a society in ways that enhance their acceptability and efficacy. It is also argued that social protection must be seen by governments of emerging economies as a developmentalist tool [27] and that it should be based on the availability and sustainability of local services [69] in order to be acceptable and feasible. Studies in Southern and Eastern Africa have found cost-effectiveness of national cash transfers and school support for adolescents in generalized epidemics [70], with long-term savings on avoidance of future negative outcomes. As the sustainable development goals move away from an HIV focus towards broader aims and integrated responses, there may be increasing rationale for programming that demonstrates evidence of multiple health and development outcomes [71]. The STRIVE consortium demonstrated that co-financing from multiple government departments that benefit from social transfers can result in manageable budgetary commitments [72,73], and this is reflected in trends towards domestically funded social protection programmes in the region.

### The next research agenda: combination prevention and protection

There are pockets of vulnerable populations across the globe, but this must not obscure the reality that adolescents in Southern Africa and in particular girls and young women bear the heaviest burden of HIV risk.

However, research and interventions among these populations have been conducted primarily in the global north. Delany-Moretlwe's review of health services for young key populations points to the lack of research on barriers and facilitators of comprehensive health care among adolescents in Africa, with less than 10% of studies cited located in an African setting. Lall *et al*. highlight the urgent need for research on ART adherence among HIV-positive adolescents and youth, with most studies and programming on ART adherence and retention in care among adolescents conducted in Northern America [74,75]. It will be important to understand how social protection policies interact with availability and acceptability of sexual and reproductive health services in high-epidemic contexts and how that may affect adolescent girls and young women in particular. We must also be cautious of assuming transferability of findings across contexts, both between and across high and low resource settings. Research must grapple with the situation-dependent and context-specific nature of behaviour and identity and how these are negotiated, produced, gendered and constructed.

It is increasingly clear that public health interventions must be adapted to local contexts if they are to succeed on a sustained basis [76]. Evidence suggests that social protection may be an effective facilitator of biomedical HIV prevention and treatment programming. Research to date suggests good evidence on cash transfers, emerging evidence on other forms of social protection and demonstrable increased effects of combining specific forms of social protection on reducing HIV risks for adolescents.

However no known research to date has examined effects of actively joining biomedical and social protection programming, “combination prevention and protection.” This presents an urgent research agenda, as highlighted by Celum and colleagues in a recent JIAS commentary [77]. HIV programming must be acceptable and effective for adolescents, both male and female [78]. It must also take into account the particularities of HIV risk and resilience in the unique contexts of Southern and Eastern Africa. Without access to condoms, circumcision, ART and potentially PrEP, there is little opportunity for adolescents to protect themselves and others. Social protection holds the potential to allow adolescents to use those opportunities.

## Conclusions

Despite the momentous progress that has been made in HIV treatment and prevention and the ambitious commitments to serving the needs of most at-risk populations, programmes remain inadequate to the major challenges adolescents face. While the global north has begun to speak of the “end of HIV,” those in the global south continue to grapple with endemic weaknesses in health systems, high incidence and the challenges of providing equitable, sustained access to HIV treatment and prevention. However even where there is access, severe structural and social deprivations experienced by the most vulnerable adolescents create barriers to uptake of and adherence to HIV prevention and treatment programmes. The UNAIDS publication on its 90–90–90 goals describes how the HIV epidemic galvanized global efforts to achieve greater equity, but emphasizes that, in order for these goals to be realized, the powerful momentum to provide programmatic responses must be sustained. Moreover, it highlights intolerable gaps in HIV treatment and service provision for adolescents in sub-Saharan Africa and the persistence of social and systemic challenges [3, pp. 7, 13].

Social protection that combines cash, care and classroom and other provisions can potentially reduce adolescent HIV-risk behaviour in the Southern and Eastern African region. Early evidence suggests that social protection may also increase adherence to PMTCT and ART for adolescents and youth. The next step in research and programming is to actively combine biomedical with social protection programmes (and potentially with efficacious behavioural programmes). With these combinations, access to biomedical technologies, social support and sufficient economic stability, we may be able to stem the adolescent epidemic.

## References

[1] UNICEF (2013). Towards an AIDS-free generation – children and AIDS: sixth stocktaking report.

[2] Cluver L, Orkin FM, Toska E, Hodes R, Sherr L Cash plus care, prevention and adherence: is social protection a Southern African answer?.

[3] UNAIDS (2014). 90-90-90: an ambitious treatment target to help end the AIDS epidemic.

[4] World Health Organization (2014). Adolescence: a second chance in the second decade.

[5] Idele P, Gillespie A, Porth T, Suzuki C, Mahy M, Kasedde S (2014). Epidemiology of HIV and AIDS among adolescents: current status, inequities, and data gaps. J Acquir Immune Defic Syndr.

[6] Nachega JB, Hislop M, Nguyen H, Dowdy DW, Chaisson RE, Regensberg L (2009). Antiretroviral therapy adherence, virologic and immunologic outcomes in adolescents compared with adults in southern Africa. J Acquir Immune Defic Syndr.

[7] Hudelson C, Cluver L (2015). Factors associated with adherence to antiretroviral therapy among adolescents living with HIV/AIDS in low- and middle-income countries: a systematic review. AIDS Care.

[8] Marrazzo JM, Ramjee G, Richardson BA, Gomez K, Mgodi N, Nair G (2015). Tenofovir-based preexposure prophylaxis for HIV infection among African women. N Engl J Med.

[9] Nglazi MD, Kranzer K, Holele P, Kaplan R, Mark D, Jaspan H (2012). Treatment outcomes in HIV-infected adolescents attending a community-based antiretroviral therapy clinic in South Africa. BMC Infec Dis.

[10] Nattrass N (2009). Poverty, sex and HIV. AIDS Behav.

[11] Mishra, Vinod (2007). HIV infection does not disproportionately affect the poorer in sub-Saharan Africa. AIDS.

[12] Gillespie S, Kadiyala S, Greener R (2007). Is poverty or wealth driving HIV transmission?. AIDS.

[13] Seeley J, Watts CH, Kippax S, Russell S, Heise L, Whiteside A (2012). Addressing the structural drivers of HIV: a luxury or necessity for programmes?. J Int AIDS Soc.

[14] Piot P, Greener R, Russell S (2007). Squaring the circle: AIDS, poverty and human development. PLoS Med.

[15] Robinson J, Yeh E (2011). Transactional sex as a response to risk in Western Kenya. Am Econ J Appl Econ.

[16] Dinkelman T, Lam D, Leibbrandt M (2008). Linking poverty and income shocks to risky sexual behaviour: evidence from a panel study of young adults in Cape Town. S Afr J Econ.

[17] UNAIDS (2013). Report on the global AIDS epidemic, 2013.

[18] Watts C, Seeley J (2014). Addressing gender inequality and intimate partner violence as critical barriers to an effective HIV response in sub-Saharan Africa. J Int AIDS Soc.

[19] Cluver L, Orkin M, Boyes M, Gardner F, Meinck F (2011). Transactional sex amongst AIDS-orphaned and AIDS-affected adolescents predicted by abuse and extreme poverty. J Acquir Immune Defic Syndr.

[20] Sawyer SM, Afifi RA, Bearinger LH, Blakemore SJ, Dick B, Ezeh AC (2012). Adolescence: a foundation for future health. Lancet.

[21] UNDP (2014). Discussion paper: cash transfers and HIV-prevention.

[22] UNICEF (2015). Social Protection programmes contribute to HIV prevention.

[23] Miller E, Samson M (2012). HIV-sensitive social protection: state of the evidence 2012 in sub-Saharan Africa.

[24] Devereux S, Sabates-Wheeler R (2004). Transformative social protection. IDS working paper 232.

[25] UNICEF (2012). UNICEF social protection strategic framework.

[26] Devereux S (2012). Social protection for enhanced food security in sub-Saharan Africa. Food Policy.

[27] de Haan A (2014). The rise of social protection in development: progress, pitfalls and politics. Eur J Dev Res.

[28] Haushofer J, Fehr E (2014). On the psychology of poverty. Science.

[29] Baird SJ, Garfein RS, McIntosh CT, Ozler B (2012). Effect of a cash transfer programme for schooling on prevalence of HIV and herpes simplex type 2 in Malawi: a cluster randomised trial. Lancet.

[30] Department of Social Development, SASSA, UNICEF (2012). The South African child support grant impact assessment: evidence from a survey of children, adolescents and their households.

[31] Handa S, Halpern CT, Pettifor A, Thirumurthy H (2014). The government of Kenya's cash transfer program reduces the risk of sexual debut among young people age 15–25. PLoS One.

[32] Cluver L, Boyes M, Orkin M, Molwena T, Sherr L (2013). Child-focused state cash transfers and adolescent risk of HIV infection in South Africa: a propensity-score-matched case-control study. Lancet Glob Health.

[33] UNC Population Center, UNICEF, FAO (2015). The transfer project [Internet].

[34] Cluver L, Orkin FM, Boyes ME, Sherr L (2014). Cash plus care: social protection cumulatively mitigates HIV-risk behaviour among adolescents in South Africa. AIDS.

[35] Cluver L (2015). Cash, care, prevention and adherence: is social protection the South African answer?.

[36] Ssewamala FM, Han CK, Neilands TB, Ismayilova L, Sperber E (2010). Effect of economic assets on sexual risk-taking intentions among orphaned adolescents in Uganda. Am J Public Health.

[37] Ssewamala FM, Nabunya P, Mukasa NM, Ilic V, Nattabi J (2014). Integrating a mentorship component in programming for care and support of AIDS-orphaned and vulnerable children: lessons from the Suubi and Bridges Programs in Sub-Saharan Africa. Glob Soc Welf.

[38] Pettifor A, Bekker LG, Hosek S, DiClemente R, Rosenberg M, Bull SS (2013). Preventing HIV among young people: research priorities for the future. J Acquir Immune Defic Syndr.

[39] Mofenson L, Cotton M (2013). The challenge of success: adolescents with perinatal HIV infection. J Int AIDS Soc.

[40] Sherr L, Croome N, Bradshaw K, Parra Castaneda K (2014). A systematic review examining whether interventions are effective in reducing cognitive delay in children infected and affected with HIV. AIDS Care.

[41] Sohn, Hazra (2013). The changing epidemiology of the global paediatric HIV epidemic: keeping track of perinatally HIV-infected adolescents. J Int AIDS Soc.

[42] Vreeman RC, Wiehe SE, Pearce EC, Nyandiko WM (2008). A systematic review of pediatric adherence to antiretroviral therapy in low- and middle-income countries. Pediatric Infect Dis J.

[43] Lowenthal E, Lawler K, Harari N, Moamogwe L, Masunge J, Masedi M (2012). Rapid psychosocial function screening test identified treatment failure in HIV+ African youth. AIDS Care.

[44] Soeters H, Pediatric AIDS Treatment for Africa, World Health Organization (2015). Adolescent Africa: a situational analysis of adolescent HIV-treatment and care in sub-Saharan Africa.

[45] Cluver L, Hodes R, Toska E, Kidia KK, Orkin FM, Sherr L (2015). ‘HIV is like a tsotsi. ARVs are your guns’: associations between HIV-disclosure and adherence to antiretroviral treatment among adolescents in South Africa. AIDS.

[46] Emenyonu N, Muyindike W, Habayarimana J, Pops-Eleches C, Thirumurthy N, Ragland K (2012). Cash transfers to cover clinic transportation costs improve adherence and retention in care in a HIV treatment program in rural Uganda.

[47] Fatti G, Shaikh N, Eley B, Grimwood A (2014). Improved virological suppression in children on antiretroviral treatment receiving community-based adherence support: a multicentre cohort study from South Africa. AIDS Care.

[48] Grimwood A, Fatti G, Mothibi E, Malahlela M, Shea J, Eley B (2012). Community adherence support improves programme retention in children on antiretroviral treatment: a multicentre cohort study in South Africa. J Int AIDS Soc.

[49] Lewycka S, Mwansambo C, Rosato M, Kazembe P, Phiri T, Mganga A (2013). Effect of women's groups and volunteer peer counselling on rates of mortality, morbidity, and health behaviours in mothers and children in rural Malawi (MaiMwana): a factorial, cluster-randomised controlled trial. Lancet.

[50] Richter L, Rotheram-Borus MJ, Van Heerden A, Stein A, Tomlinson M, Harwood JM (2014). Pregnant women living with HIV (WLH) supported at clinics by peer WLH: a cluster randomized controlled trial. AIDS Behav.

[51] McCoy S (2015). Comparing food and cash assistance for HIV-positive men and women on antiretroviral therapy in Tanzania [Internet]. Clinical Trials Registry.

[52] Ssewamala FM (2013). Evaluating a youth-focused economic empowerment approach to HIV treatment adherence [Internet].

[53] World Health Organization (2015). Report of the consultation on the treatment of HIV among adolescents.

[54] de Walque D, Dow WH, Nathan R, Abdul R, Abilahi F, Gong E (2012). Incentivising safe sex: a randomised trial of conditional cash transfers for HIV and sexually transmitted infection prevention in rural Tanzania. BMJ Open.

[55] Pettifor A, MacPhail C, Nguyen A, Rosenberg M (2012). Can money prevent the spread of HIV? A review of cash payments for HIV prevention. AIDS Behav.

[56] Galarraga O, Genberg BL, Martin RA, Barton Laws M, Wilson IB (2013). Conditional economic incentives to improve HIV treatment adherence: literature review and theoretical considerations. AIDS Behav.

[57] El-Sadr WM (2015). Effect of financial incentives on linkage to care and viral suppression: HPTN 065.

[58] Dartnall E, Jewkes R (2013). Sexual violence against women: the scope of the problem. Best Pract Res Clin Obstet Gynaecol.

[59] Jewkes R, Dunkle K, Nduna M, Shai N (2010). Intimate partner violence, relationship power inequity, and incidence of HIV infection in young women in South Africa: a cohort study. Lancet.

[60] Hargreaves J, Glynn J (2002). Educational attainment and HIV-1 infection in developing countries: a systematic review. Trop Med Int Health.

[61] Thornton R (2008). The demand for, and impact of, learning HIV status. Am Econ Rev.

[62] Pettifor A (2013). Effects of cash transfer and community mobilization for prevention of HIV in young South African women. HPTN 068.

[63] Abdool Karim Q (2015). Impact of conditional cash incentives on HSV-2 and HIV prevention in rural high school students in South Africa: the CAPRISA 007 cluster randomized controlled trial.

[64] SASSA A statistical summary of social grants in South Africa: fact sheet issue no. 3 of 2015 [Internet].

[65] PEFPAR (2015). Preventing HIV in adolescent girls and young women: guidance for PEPFAR country teams on the DREAMS partnership.

[66] Garcia M, Moore C (2012). The cash dividend: the rise of cash transfer programs in sub-Saharan Africa.

[67] Seekings J (2007). The mutability of distributive justice attitudes in South Africa. S Afr Rev Sociol.

[68] Adato M, Bassett L (2009). Social protection to support vulnerable children and families: the potential of cash transfers to protect education, health and nutrition. AIDS Care.

[69] Delany-Moretlwe S, Cowan FM, Busza J, Bolton-Moore C, Kelley K, Fairlie L (2015). Providing comprehensive health services for young key populations: needs, barriers and gaps. J Int AIDS Soc.

[70] Remme M, Siapka M, Vassall A, Heise L, Jacobi J, Ahumada C (2014). The cost and cost-effectiveness of gender-responsive interventions for HIV: a systematic review. J Int AIDS Soc.

[71] UNICEF LEAP briefing paper [Internet].

[72] Kim JC, Julia C (2011). The ‘AIDS and MDGs’ approach: what is it, why does it matter, and how do we take it forward?. Third World Q.

[73] Remme M, Vassall A, Lutz B, Luna J, Watts C (2014). Financing structural interventions: going beyond HIV-only value for money assessments. AIDS.

[74] Mutumba M, Harper G (2015). Mental health and support among young key populations: an ecological approach to understanding and intervention. J Int AIDS Soc.

[75] Lall P, How Lim S, Khairuddin, Norliana, Kamarulzaman, Adeeba (2015). Review: an urgent need for research on factors impacting adherence to and retention in care among HIV-positive youth and adolescents from key populations. J Int AIDS Soc.

[76] Pettifor A, Nguyen NL, Celum C, Cowan FM, Go V, Hightow-Weidman L (2015). Tailored combination prevention packages and PrEP for young key populations. J Int AIDS Soc.

[77] Celum CL, Delany-Moretlwe S, McConnell M, van Rooyen H, Bekker LG, Kurth A (2015). Rethinking HIV prevention to prepare for oral PrEP implementation for young African women. J Int AIDS Soc.

[78] Bekker LG (2015). Building our youth for the future. J Int AIDS Soc.

# Sub article: Integration of HIV in child survival platforms: a novel programmatic pathway towards the 90–90–90 targets

**DOI:** 10.7448/IAS.18.7.20250

**Published:** 2015-12-02

**Authors:** Dick D Chamla, Shaffiq Essajee, Mark Young, Scott Kellerman, Ronnie Lovich, Nandita Sugandhi, Anouk Amzel, Chewe Luo

**Affiliations:** 1 Health Section, UNICEF, New York, NY, USA; 2 HIV Department, World Health Organization, Geneva, Switzerland; 3 Global HIV Program, Management Sciences for Health, Arlington, VA, USA; 4 MCH Department, Elizabeth Glaser Pediatric AIDS Foundation, Washington, DC, USA; 5 HIV Department, Clinton Health Access Initiative, New York, NY, USA; 6 HIV Office, USAID, Washington, DC, USA; 7 HIV Section, UNICEF, New York, NY, USA

**Keywords:** integration, HIV, child survival, double dividend, maternal-child health, PITC

## Abstract

**Introduction:**

Integration of HIV into child survival platforms is an evolving territory with multiple connotations. Most literature on integration of HIV into other health services focuses on adults; however promising practices for children are emerging. These include the Double Dividend (DD) framework, a new programming approach with dual goal of improving paediatric HIV care and child survival. In this commentary, the authors discuss why integrating HIV testing, treatment and care into child survival platforms is important, as well as its potential to advance progress towards global targets that call for, by 2020, 90% of children living with HIV to know their status, 90% of those diagnosed to be on treatment and 90% of those on treatment to be virally suppressed (90–90–90).

**Discussion:**

Integration is critical in improving health outcomes and efficiency gains. In children, integration of HIV in programmes such as immunization and nutrition has been associated with an increased uptake of HIV infant testing. Integration is increasingly recognized as a case-finding strategy for children missed from prevention of mother-to-child transmission programmes and as a platform for diffusing emerging technologies such as point-of-care diagnostics. These support progress towards the 90–90–90 targets by providing a pathway for early identification of HIV-infected children with co-morbidities, prompt initiation of treatment and improved survival. There are various promising practices that have demonstrated HIV outcomes; however, few have documented the benefits of integration on child survival interventions. The DD framework is well positioned to address the bidirectional impacts for both programmes.

**Conclusions:**

Integration provides an important programmatic pathway for accelerated progress towards the 90–90–90 targets. Despite this encouraging information, there are still challenges to be addressed in order to maximize the benefits of integration.

## Introduction

Integration of health services for children that incorporate aspects of HIV care and treatment with other childhood illnesses is high on the global health and development agenda. Yet defining and measuring integration remains elusive. In the health sector, integration of services in primary health care (PHC) can be traced back to the Alma Ata declaration adopted in 1978, which recognized the importance of primary health care as key to achieving the goal of “Health For All” [1]. The Paris Declaration on Aid Effectiveness in 2005 further reaffirmed the importance of integration as a country-owned process built upon existing programmes [2]. For decades, debates on the comparative impact of *vertical* versus *integration* systems have had polarizing views mainly due to lack of hard evidence [3].

The World Health Organization defines *integration* as the management and delivery of health services, where clients receive a continuum of preventive and curative services according to their needs over time and across different levels of the health system [4]. In practice, integration has many forms, including co-location of services at one facility, co-delivery of multiple services by one programme or combined services for every client in one encounter to ensure multiple needs are met. More often, the idea of comprehensiveness overlaps with that of integration [5]. There is also a range of descriptions of integration, such as a “supermarket” approach for service delivery, “one-stop shop” or delivery of services “under the same roof.” A more recent concept that has emerged in paediatric HIV is that of “smart integration,” which underlines the importance of data-driven, targeted and feasible choices in models of integrated services aimed at maximizing child survival outcomes. Smart integration calls for rigorous analysis of gaps in service delivery and coverage, to guide the design of feasible and targeted integration of services. Additionally, it calls for robust monitoring strategies to be in place to track outcomes and potential negative consequences for both new and existing services. In the HIV field, there is growing literature on improved outcomes following integration of HIV with programmes such as tuberculosis (TB) and family planning among adult populations [6–8]; however, such evidence among children remains rudimentary.

This paper examines why integrating HIV services into child survival platforms is important and discusses promising strategies, opportunities and challenges of integrating HIV services into selected child survival programmes to improve both HIV and child survival outcomes. This includes the Double Dividend (DD) framework, a new programming approach that describes the bidirectional benefits of integrating HIV services with child survival platforms [9]. The article aims to show that integration is a key strategy needed for survival of children living with HIV and for the delivery of services and uptake of new technologies critical to reaching the new global target that calls for, by 2020, 90% of children living with HIV to know their status; 90% of those diagnosed to be on treatment; and 90% of those on treatment to be virally suppressed (90–90–90).

## Discussion

The rationale for integration dwells on its potential to improve service delivery, health outcomes and efficiencies [10]. For example, in Uganda, the introduction of Integrated Management of Childhood Illness (IMCI) led to improved performance of trained health workers and superior quality of care delivered to children aged under five years [11]. Similar findings on improved outcomes following IMCI implementation were reported in Bangladesh, South Africa and Tanzania [12–14]. A newborn mortality reduction of 34 to 62% was also demonstrated through the delivery of a package of interventions shortly after birth [15,16]. For HIV-exposed children, there are indications of improved uptake of HIV testing following integration with programmes such as immunization and nutrition [17–19]. However, there is a lack of evidence of potential impact on child survival programmes following their integration with HIV.

Integration of HIV testing in child survival programmes is increasingly recognized as a case-finding strategy for children who were missed in prevention of mother-to-child transmission of HIV (PMTCT) programmes or who were infected late during the breastfeeding period. This issue is crucial, as global coverage of early infant diagnosis remains low at 39% among the 22 priority countries that contribute to over 90% of new paediatric HIV infections globally [20]. In spite of growing literature on HIV co-morbidities [21,22], the contribution of HIV in major causes of under-five mortality rates (U5MR), such as pneumonia or diarrhoea, remains largely unknown. Given the potential for case finding within service delivery platforms for sick, malnourished and well-child care, HIV testing at various child survival entry points offers significant potential for accelerated case finding, clear pathways to HIV treatment initiation and may contribute to retention in care. However, decades of vertical programming of paediatric HIV care has missed these opportunities, resulting in poor survival outcomes for children with co-morbidities [22].

Recent successes of the PMTCT programmes in reducing HIV transmission in the prenatal and postnatal periods provide another reason for integration of services for children. There are fewer HIV-infected children being born and identified through successful PMTCT programmes [23]. Therefore, the majority of yet-unidentified HIV-infected children are those born to women that were missed or lost from PMTCT programmes. Most of these unidentified HIV-infected children could be identified through HIV testing in other facility-based programmes that care for children, such as immunization, nutrition or inpatient services. Last, the importance of integration could also be viewed as a platform for diffusing technological innovations such as point-of-care diagnostics (PoC) for viral load assays [24] in order to maximize their impacts in child survival programmes.

There are two approaches for integrating HIV testing into child survival platforms that can be considered: general integrated platforms, such as in immunization sites, where integration would enable universal HIV testing or screening of all children, versus targeted integration, where the focus for testing would be on a subset of high risk children (such as sick or malnourished children). The choice of these approaches is likely determined by each country's HIV epidemic context and health system characteristics. For instance, integration of HIV testing into routine immunization sessions may be ideal in high HIV prevalence countries [25], whereas screening of all infants for risk of HIV exposure may be a more effective practice in low level epidemics.

Beyond integration of HIV testing, there is a need to establish feasible approaches to improving retention of HIV-exposed infants and HIV-infected infants and children in HIV care and treatment services within maternal and child health (MCH) settings. What is seen in the literature is that most of the focus of integration is on HIV testing and it does not examine delivery of paediatric HIV treatment through MCH service delivery points. In Mozambique, care of HIV-exposed and -infected children is being delivered together with care for other at-risk children through “Child at Risk” clinics providing an integrated model of service delivery for all paediatric health issues.

### Promising integrated approaches

Many countries have integrated HIV testing into one or more child survival platforms; however few have documented the specific outcomes due to lack of robust evaluation designs. There are a few instances, such as integration of HIV testing in immunization and nutrition programmes, paediatric inpatient units and community or home-based programmes [26–36], that have demonstrated excellent outcomes in HIV case finding (Box 1). Fewer studies, however, have shown the effect on child health platforms when HIV testing is incorporated. The need for maximizing the outcomes for both child survival and paediatric HIV interventions has been the guiding mantra for the DD framework.

**Box 1. Promising approaches for integrating HIV into child survival platforms**

HIV and immunization integration

Increased uptake of HIV testing through immunization services has been well described [18]. In some settings, more than 90% of mothers accepted the offer to test their infants during immunization sessions [26]. Other studies showed a sevenfold greater proportion of infants receiving HIV testing in immunization clinics than in under-five clinics [19]. There is growing consensus on the effectiveness of this approach in increasing coverage of HIV infant testing and thereby helping to reach the UNAIDS target of 90% of infected children knowing their HIV status. However, further study of the effects of that integration on basic immunization platforms as well as the yield from case findings in different epidemic contexts would be valuable. There remains the challenge of stigma reported by other studies [29], which needs careful attention during design and monitoring of new integration initiatives.

HIV and nutrition integration

Malnourished children infected with HIV have an increased risk of mortality [22]. Routine HIV testing for early identification and prompt initiation of antiretroviral therapy (ART) have been associated with improved nutritional recovery and overall survival of infected children [17]. Uptake of HIV testing of more than 94% for children in nutrition programmes has been reported [30]. As such, both facility- and community-based nutrition therapeutic centres have been valuable entry points for HIV testing in most countries [30]. There are now studies confirming the association between food insecurity and unsuppressed viral load among ART patients [27,45].

HIV testing in children’s clinics and wards

There is mounting experience in providing HIV testing to hospitalized or out-patient paediatric populations in high HIV prevalence settings. In Zambia over 87% of paediatric patients admitted to one hospital received HIV testing [28]. Of those, 29% were identified as HIV positive. In one meta-analysis, up to one-fifth of pneumonia cases and 60% of pneumonia deaths were shown to occur in HIV-infected children [21]. As the efficiency and reach of prevention of mother-to-child transmission of HIV (PMTCT) programmes increase, a focus on children sick enough to be admitted to hospital may prove effective in early identification of HIV-infected children that “fall through the cracks” of PMTCT services.

HIV integration in community-based programmes

For decades, a community component of paediatric HIV response has been overlooked. Promising community-based paediatric HIV practices are now evident. The “Tingathe” community project in Malawi successfully used community health workers, with increased uptake of maternal and infant HIV testing [32]. Home-based HIV testing for children has been piloted in countries such as Swaziland [36]. In 2013, UNICEF and WHO revised their community health care training packages and materials for integrated community case management for pneumonia, diarrhoea and malaria, caring for newborns in the community and care of the well child to include HIV and tuberculosis [31]. Upcoming pilot implementation of these adapted materials remains critical.

### Double Dividend

The DD is a framework intended to catalyze actions toward the dual goals of accelerating paediatric HIV prevention, care and treatment, while contributing to improvements in child survival [9]. It calls for a systematic approach from both a child health and paediatric HIV perspective, to strengthen linkages and targeted integration across the health system based on analysis of gaps in coverage and needs for strengthening service delivery platforms. It also acknowledges the need to improve the response for HIV-exposed children, who face additional vulnerabilities as the risk of death among HIV-exposed uninfected children continues to rise [37]. However most programmes have yet to incorporate this emerging evidence in their HIV response.

Through preliminary analysis undertaken during a think tank exercise in Harare, the architects of DD identified five key areas where integration of HIV and child survival platforms can be mutually beneficial and therefore improve child outcomes – in postnatal care settings, immunization clinics, nutrition points, IMCI and integrated community case management for pneumonia, diarrhoea and malaria. The DD framework proposes four operational steps that will enable countries to 1) identify major causes of U5MR, paediatric HIV infections and the unmet care needs in these areas; 2) design integrated approaches for programming in child survival and paediatric HIV based on the needs assessment; 3) deliver and monitor the package of integrated services developed; and 4) work to ensure sustainability.

There are several novel dimensions to the framework: an expanded definition of the paediatric HIV-affected population to include HIV-exposed uninfected children; targeted integration of paediatric HIV in broader maternal, newborn and child health (MNCH) platforms; and greater understanding of the role that paediatric HIV infection plays in relation to common childhood diseases such as pneumonia, malaria and diarrhoea. The DD calls for a major shift in paediatric HIV programming, away from its current vertical delivery model, with the overall aim of identifying areas where investments of effort and resources will maximize prevention, care and treatment outcomes while contributing to strengthened child survival platforms.

The DD comes during dialogues of post-millennium development goals and new calls to action for child and newborn health, with new targets and timelines for achieving goals [38]. New global targets for paediatric HIV, 90–90–90, have also been endorsed [39]. Convergence of these initiatives to achieve the 90–90–90 targets in countries should be an obvious next step. Among the 11 countries endorsing the DD in 2013, Zimbabwe has begun taking steps to ensure that accelerated action to find HIV-infected children and initiate treatment can be undertaken through strengthened child survival platforms. There are critical lessons that can be adapted in other countries.

### Opportunities

As the world moves towards sustainable development goals, we have an unprecedented opportunity to reduce the silos existing in the implementation of paediatric HIV programmes. In 2011, in the United Nations General Assembly High Level Meeting on HIV, heads of state committed to eliminating parallel systems and integrating HIV services into health and broader development programming. Efforts are needed to ensure HIV-infected children are included in the Universal Health Coverage Initiative [40], health system strengthening and equity agendas and when addressing unmet needs for newborn and child survival [38]. The momentum for childhood TB efforts also provides an important opportunity for child survival to be further optimized [41]. Other opportunities for inclusion of HIV into child survival platforms include its integration in the already scaled-up PMTCT/antiretroviral therapy (ART) programming for women in MCH settings (Option B+) [42,43]. Likewise, the community health worker (CHW) movements such as One Million CHW by 2015 [44] have been silent on the unmet needs in paediatric HIV – missing a clear opportunity to improve survival of children exposed to or infected with HIV.

### Challenges for integration

The majority of critical gaps that hamper HIV service delivery also threaten basic health services. In many countries, MNCH, particularly post-natal platforms, is underdeveloped, under-utilized and in most cases under-funded. The extent and monitoring of outcomes following integration remains a challenge. For instance, most literature has focused on integration of HIV and child survival platforms at the service delivery level (Box 1), with little information on full integration across the critical elements of health systems such as governance, human resources, information or financing. As previously discussed, the outcomes on child survival platforms following their integration with HIV are rarely reported. For example, understanding how HIV integration into vaccination sites may impact completion of the full immunization schedule could be beneficial to the immunization community. As the impact of integration of HIV services into child health platforms is not yet widely known, robust studies are needed to evaluate the effects.

### Integration and the 90–90–90 goals

Addressing persistent gaps in the provision of paediatric HIV services and ultimately the global targets of 90–90–90 by 2020 requires innovative approaches. Integration, as one of the programmatic innovations, has the potential to accelerate this goal through intensified case finding in child survival platforms, timely treatment initiation, improved retention and ultimately long-term survival of children exposed to or infected with HIV. Implementing the approaches that integrate HIV testing into child survival platforms (as presented in Box 1), would greatly accelerate progress towards the first 90 goal – 90% of HIV-infected children knowing their status. Delivery of paediatric ART in the MCH facilities alongside PMTCT programming (Option B+) remains a critical strategy to advance the second 90 goal – ensuring that 90% of those who test positive receive treatment. Synergies between HIV and other programmes such as nutrition enhance viral suppression for those on ART [27,45] and improve the achievement of the third 90 goal – 90% of children on ART to maintain viral suppression. These benefits are further amplified by the potential for efficiency gains and dissemination of new technologies necessary to accelerate progress towards the 90–90–90 goals. Strategies such as DD provide new momentum for programming approaches to reach the 90–90–90 goals. There are, however, challenges that need to be addressed and joint investments are needed to ensure that integration achieves maximum impacts for both platforms.

## Conclusions

The field of integration of HIV into child survival platforms is evolving with remarkable prospects. Its potential role in case identification, treatment initiation and dissemination of new technologies should form a critical part of strategies towards the achievement of the 90–90–90 goals. The potential for the field of integration could be additionally enhanced by combining with emerging technological advances such as PoC diagnostics and optimized paediatric antiretroviral drug formulations. Defining the bidirectional dividends, including the burden of HIV in other major child health diseases, will enhance optimal care and joint investments. Adequate investments and implementation research are needed to ensure that optimal integrated models are identified and scaled up, both to achieve the goals of 90–90–90 in paediatric HIV and to improve child survival outcomes.

## References

[1] World Health Organization (1978). Declaration of Alma-Ata.

[2] Organization for Economic Cooperation and Development (2005). The Paris declaration on Aid effectiveness.

[3] Atun R, de Jongh T, Secci F, Ohiri K, Adeyi O (2010). A systematic review of the evidence on integration of targeted health interventions into health systems. Health Policy Plan.

[4] World Health Organization (2008). Integrated health services – what and why. Technical brief no. 1.

[5] Kodner DL, Spreeuwenberg C (2002). Integrated care: meaning, logic, applications, and implications – a discussion paper. Int J Integr Care.

[6] Onono M, Guze MA, Grossman D, Steinfeld R, Bukusi EA, Shade S (2015). Integrating family planning and HIV services in western Kenya: the impact on HIV-infected patients’ knowledge of family planning and male attitudes toward family planning. AIDS Care.

[7] Kendall T, Barnighausen T, Fawzi WW, Langer A (2014). Towards comprehensive women's healthcare in sub-Saharan Africa: addressing intersections between HIV, reproductive and maternal health. J Acquir Immune Defic Syndr.

[8] Ferroussier O, Dlodlo RA, Capo-Chichi D, Boillot F, Gninafon M, Trebucq A (2013). Results of rapid and successful integration of HIV diagnosis and care into tuberculosis services in Benin. Int J Tuberc Lung Dis.

[9] United Nations Children's Fund (2013). Double dividend.

[10] Integra (2013). Making sense of complexity: key findings from the Integra Initiative. Integra Newsletter.

[11] Pariyo GW, Gouws E, Bryce J, Burnham G (2005). Uganda IMCI Impact Study Team. Improving facility-based care for sick children in Uganda: training is not enough. Health Policy Plan.

[12] Masanja H, Schellenberg JA, de Savigny D, Mshinda H, Victora CG (2005). Impact of integrated management of childhood illness on inequalities in child health in rural Tanzania. Health Policy Plan.

[13] Chopra M, Patel S, Cloete K, Sanders D, Peterson S (2005). Effect of an IMCI intervention on quality of care across four districts in Cape Town, South Africa. Arch Dis Child.

[14] Arifeen SE, Bryce J, Gouws E, Baqui AH, Black RE, Hoque DM (2005). Quality of care for under-fives in first-level health facilities in one district of Bangladesh. Bull World Health Organ.

[15] Darmstadt GL, Bhutta ZA, Cousens S, Adam T, Walker N, de Bernis L (2005). Evidence-based, cost-effective interventions: how many newborn babies can we save?. Lancet.

[16] Baqui A, Williams EK, Rosecrans AM, Agrawal PK, Ahmed S, Darmstadt GL (2008). Impact of an integrated nutrition and health programme on neonatal mortality in rural northern India. Bull World Health Organ.

[17] Kim MH, Cox C, Dave A, Draper HR, Kabue M, Schutze GE (2012). Prompt initiation of ART with therapeutic food is associated with improved outcomes in HIV-infected Malawian children with malnutrition. J Acquir Immune Defic Syndr.

[18] Chamla D, Luo C, Adjorlolo-Johnson G, Vandelaer J, Young M, Costales MO (2014). Integration of HIV infant testing into immunization programmes: a systematic review. Paediatr Int Child Health.

[19] McCollum ED, Johnson DC, Chasela CS, Siwande LD, Kazembe PN, Olson D (2012). Superior uptake and outcomes of early infant diagnosis of HIV services at an immunization clinic versus an “under-five” general pediatric clinic in Malawi. J Acquir Immune Defic Syndr.

[20] United Nations AIDS Programme (2014). 2014 Progress report on the global plan towards the elimination of new HIV infections among children by 2015 and keeping their mothers alive.

[21] Theodoratou E, McAllister DA, Reed C, Adeloye DO, Rudan I, Muhe LM (2014). Global, regional, and national estimates of pneumonia burden in HIV-infected children in 2010: a meta-analysis and modelling study. Lancet Infect Dis.

[22] Fergusson P, Tomkins A (2009). HIV prevalence and mortality among children undergoing treatment for severe acute malnutrition in sub-Saharan Africa: a systematic review and meta-analysis. Trans R Soc Trop Med Hyg.

[23] Chamla D, Mbori-Ngacha D, Newman M, Kellerman S, Sugandhi N, Rwebembera A (2013). Evidence from the field: missed opportunities for identifying and linking HIV-infected children for early initiation of ART. AIDS.

[24] Ritchie AV, Ushiro-Lumb I, Edemaga D, Joshi HA, De Ruiter A, Szumilin E (2014). SAMBA HIV semiquantitative test, a new point-of-care viral-load-monitoring assay for resource-limited settings. J Clin Microbiol.

[25] Ahmed S, Kim MH, Sugandhi N, Phelps BR, Sabelli R, Diallo MO (2013). Beyond early infant diagnosis: case finding strategies for identification of HIV-infected infants and children. AIDS.

[26] Rollins N, Mzolo S, Moodley T, Esterhuizen T, van Rooyen H (2009). Universal HIV testing of infants at immunization clinics: an acceptable and feasible approach for early infant diagnosis in high HIV prevalence settings. AIDS.

[27] Mendoza JA, Matshaba M, Makhanda J, Liu Y, Boitshwarelo M, Anabwani GM (2014). Food insecurity and CD4% among HIV+ children in Gaborone, Botswana. J Acquir Immune Defic Syndr.

[28] Kankasa C, Carter RJ, Briggs N, Bulterys M, Chama E, Cooper ER (2009). Routine offering of HIV testing to hospitalized pediatric patients at university teaching hospital, Lusaka, Zambia: acceptability and feasibility. J Acquir Immune Defic Syndr.

[29] Goodson JL, Finkbeiner T, Davis NL, Lyimo D, Rwebembera A, Swartzendruber AL (2013). Evaluation of using routine infant immunization visits to identify and follow-up HIV-exposed infants and their mothers in Tanzania. J Acquir Immune Defic Syndr.

[30] Bahwere P, Piwoz E, Joshua MC, Sadler K, Grobler-Tanner CH, Guerrero S (2008). Uptake of HIV testing and outcomes within a Community-based Therapeutic Care (CTC) programme to treat severe acute malnutrition in Malawi: a descriptive study. BMC Infect Dis.

[31] World Health Organization, United Nations Children's Fund (2014). Caring for newborns and children in the community, adaptation for high HIV or TB settings.

[32] Kim MH, Ahmed S, Buck WC, Preidis GA, Hosseinipour MC, Bhalakia A (2012). The Tingathe programme: a pilot intervention using community health workers to create a continuum of care in the prevention of mother to child transmission of HIV (PMTCT) cascade of services in Malawi. J Int AIDS Soc.

[33] Fergusson P, Chinkhumba J, Grijalva-Eternod C, Banda T, Mkangama C, Tomkins A (2009). Nutritional recovery in HIV-infected and HIV-uninfected children with severe acute malnutrition. Arch Dis Child.

[34] Chamie G, Kwarisiima D, Clark TD, Kabami J, Jain V, Geng E (2014). Uptake of community-based HIV testing during a multi-disease health campaign in rural Uganda. PLoS One.

[35] Mutanga JN, Raymond J, Towle MS, Mutembo S, Fubisha RC, Lule F (2012). Institutionalizing provider-initiated HIV testing and counselling for children: an observational case study from Zambia. PLoS One.

[36] Parker LA, Jobanputra K, Rusike L, Mazibuko S, Okello V, Kerschberger B (2015). Feasibility and effectiveness of two community-based HIV testing models in rural Swaziland. Trop Med Int Health.

[37] Filteau S (2009). The HIV-exposed, uninfected African child. Trop Med Int Health.

[38] United Nations Children's Fund (2012). Committing to child survival: a promise renewed.

[39] United Nations AIDS Programme (2014). 90–90–90: an ambitious treatment target to help end the AIDS epidemic.

[40] O'Connell T, Rasanathan K, Chopra M (2014). What does universal health coverage mean?. Lancet.

[41] Marais BJ, Graham SM (2014). Childhood tuberculosis: a roadmap towards zero deaths. J Paediatr Child Health.

[42] Thyssen A, Lange JH, Thyssen E, Reddi A (2013). Toward an AIDS-free generation with option B+: reconceptualizing and integrating prevention of mother to child transmission (PMTCT) with pediatric antiretroviral therapy initiatives. J Acquir Immune Defic Syndr.

[43] Kellerman S, Jay JS, Quick JD (2013). Is Option B+ the best choice?. Lancet.

[44] Singh P, Sachs JD (2013). 1 million community health workers in sub-Saharan Africa by 2015. Lancet.

[45] Mendoza JA, Paul ME, Schwarzwald H, Liu Y, Martinez R, Nicklas TA (2013). Food insecurity, CD4 counts, and incomplete viral suppression among HIV+ patients from Texas Children's Hospital: a pilot study. AIDS Behav.
